# Late‐Stage Cross‐Electrophile Coupling of Arylthianthrenium Salts with (Hetero)aryl (Pseudo)halides via Palladium Catalysis

**DOI:** 10.1002/anie.202502441

**Published:** 2025-04-14

**Authors:** Yuanhao Xie, Li Zhang, Tobias Ritter

**Affiliations:** ^1^ Max‐Planck‐Institut für Kohlenforschung Kaiser‐Wilhelm‐Platz 1 45470 Mülheim an der Ruhr Germany; ^2^ Institute of Organic Chemistry RWTH Aachen University Landoltweg 1 52074 Aachen Germany

**Keywords:** Arylthianthrenium salts, Late‐stage functionalization, Palladium catalysis, Reductive cross‐coupling reaction

## Abstract

Herein, we present a cross‐coupling reaction of arylthianthrenium salts at a late stage with diverse (hetero)aryl (pseudo)halides under reductive conditions, in which a palladium(0) catalyst differentiates between two aryl electrophiles based on the different rates of oxidative addition of arylthianthrenium salts and aryl halides for selective umpolung. A measured near‐zero Hammett rho value is consistent with oxidative addition of the arylthianthrenium salts to palladium(0) being insensitive to substituent effects, which enables reaction with structurally and electronically diverse arylthianthrenium salts. In addition, we show the robustness of this method by coupling of two complex fragments that would otherwise be difficult to access in a single step.

## Graphical abstract

By employing complex arylthianthrenium salts and readily available (hetero)aryl (pseudo)halides as starting materials, in conjunction with hypoboric acid as a reductant, structurally diverse (hetero)biaryl motifs can be rapidly synthesized via palladium‐catalyzed cross‐coupling reaction. Key to this advance is the selective and mild borylation of arylthianthrenium salts, followed by a conventional SMC (Suzuki–Miyaura cross‐coupling) reaction with aryl halides.

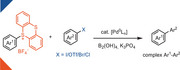

In 2023, approximately 10% of the top 200 small molecule drugs ranked by retail sales feature a (hetero)biaryl scaffold.^[^ ^1^ ^]^ Transition metal–catalyzed Csp^2^–Csp^2^ cross‐coupling reactions are robust, yet require aryl nucleophiles.^[^ ^2^, ^3^, ^4^, ^5^ ^]^ Csp^2^–Csp^2^ cross‐electrophile coupling (CEC) has emerged as a popular approach to (hetero)biaryl synthesis and require two aryl electrophiles.^[^ ^6^, ^7^, ^8^, ^9^ ^]^ Current methods for CEC have shown excellent chemoselectivity but are limited to simple aryl electrophiles thus far; CEC reactions at a late stage have not yet been reported with conventional leaving groups (Scheme 1a). Formation of organometallic reagents from an aryl electrophile in situ with concurrent cross‐coupling is challenging, and typically requires a two‐step approach.^[^ ^10^ ^]^ Here, we present the first late‐stage Csp^2^–Csp^2^ cross‐coupling reaction with functionally complex arenes, between arylthianthrenium salts and readily available (hetero)aryl iodides, bromides, chlorides and triflates (Scheme 1b), which offers a modular route for the production of (hetero)biaryl‐based complex chemical entities. Our strategy is distinguished by the selective umpolung of complex arylthianthrenium salts in the presence of aryl (pseudo)halides under mild condition while preserving robustness and excellent functional group tolerance. The facile oxidative addition of arylthianthrenium salts to palladium(0) is fast due to the distinct electronic properties of arylthianthrenium salts compared to other (pseudo)halides, which results in a chemoselective mild borylation, followed by in situ cross‐coupling. Because aryl thianthrenium salts are more readily accessible selectively at a late stage than halides,^[^ ^13^ ^]^ structurally diverse (hetero)biaryl motifs can be rapidly constructed, as exemplified by the construction of complex (hetero)biaryl **1**, which are currently difficult to achieve by other Csp^2^–Csp^2^ reductive cross‐coupling methods (Scheme 1c).

**Scheme 1:**
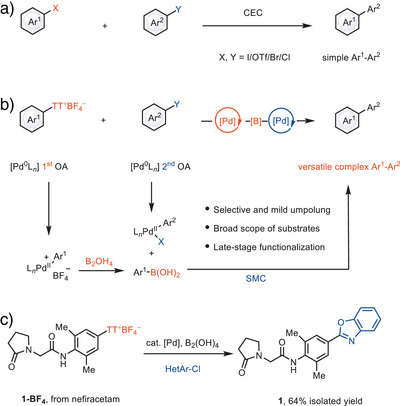
a) Transition metal–catalyzed CEC. b) Late‐stage cross‐coupling between arylthianthrenium salts and (hetero)aryl halides via palladium catalysis. c) Specific example (reaction condition see Supporting Information for details). CEC, cross‐electrophile coupling; L, ligand; TT, thianthrene; OA, oxidative addition; SMC, Suzuki–Miyaura coupling; [B], arylboron species.

The cross‐coupling reaction under reductive conditions proceeds selectively if the two aryl electrophiles exhibit different reactivity as a consequence of different electronic properties. For examples, Gosmini,^[^ ^14^, ^15^, ^16^ ^]^ Léonel,^[^ ^17^, ^18^ ^]^ Dubreuil,^[^ ^19^, ^20^ ^]^ Gong,^[^ ^21^ ^]^ Duan,^[^ ^22^ ^]^ Lautens,^[^ ^23^ ^]^ Lee,^[^ ^24^ ^]^ and Stahl^[^ ^25^ ^]^ independently have achieved considerable progress in Csp^2^–Csp^2^ CEC reactions via nickel catalysis. Shen reported CEC reactions of arylsulfonium salts via the in situ generation of Grignard reagents from aryl bromides, with the limitations of functional group tolerance expected from Grignard reagents.^[^ ^26^, ^27^ ^]^ Weix developed a fundamentally different approach involving bimetallic catalysis with palladium and nickel to use a variety of aryl (pseudo)halides. The cross‐selectivity relies on the difference in relative rates for oxidative addition of two aryl electrophiles with palladium(0) and nickel(0), respectively.^[^ ^11^, ^28^, ^29^, ^30^ ^]^ Maiti reported a light induced, monometallic CEC approach. The synergistic, dual palladium catalytic cycle can differentiate two aryl halides based on the bond dissociation energy.^[^ ^12^ ^]^ Toste and Ye have developed a zirconaaziridine mediated palladium‐catalyzed CEC between (hetero)aryl iodides and (hetero)bromides, in which the cross‐selectivity is controlled by relative rate difference of oxidative addition to palladium(0).^[^ ^31^ ^]^ Krische reported a palladium(I)‐catalyzed CEC reaction, where the facile reductive elimination from a heterodiarylpalladium intermediate leads to high cross‐selectivity.^[^ ^32^ ^]^ All of these modern advances have substantially broadened the scope of simple aryl electrophiles cross‐coupling reactions. Nevertheless, despite significant progress so far, no general method appears to be available that can achieve a cross‐coupling reaction between a complex aryl electrophile with a variety of (hetero)aryl halides; here we fill this void.

Due to the frequent occurrence of complex (hetero)biaryls in small molecule drugs,^[^ ^1^ ^]^ we focused our attention on developing a late stage Csp^2^–Csp^2^ cross‐coupling reaction for incorporation of (hetero)aryl cores on structurally complex molecules. The use of arylthianthrenium salts offers two key advantages in our design. First, the thianthrenation proceeds chemo‐ and regioselectively at a late stage,^[^ ^13^ ^]^ enabling the modification of complex molecules. Second, owing to the small BDE (bond dissociation energy)^[^ ^33^ ^]^ and low lying LUMO (lowest unoccupied molecular orbital),^[^ ^34^ ^]^ arylthianthrenium salts can proceed in a rapid oxidative addition to palladium(0), which can be applied to the design of a general and selective cross‐coupling reaction between arylthianthrenium salts and other aryl halides.^[^ ^13^, ^35^, ^36^ ^]^ In situ mild borylation, in the presence of both aryl electrophiles, affords aryl nucleophiles with broad functional groups tolerance that can subsequently undergo cross‐coupling with aryl (pseudo)halides. The faster rate for transmetallation of diboron compared to arylboron^[^ ^37^ ^]^ compounds provides chemoselectivity to minimize the formation of homo‐coupling by‐product. The soluble diboron reagent as reductant avoids the use of insoluble metal reductants, with potential advantages to scale‐up and flow reactors.^[^ ^38^ ^]^

To evaluate the potential of our reaction design, we explored the cross‐coupling reaction of the arylthianthrenium salt **2‐BF_4_** with 4‐bromofluorobenzene **2‐Br** (Scheme 2a). The cross‐product **2a** was obtained in 84% yield, with the product ratio **2a** to homocoupling dimer **2b** as 14:1. The generality of the method was explored by varying the (hetero)aryl halides. The cross‐coupling reaction of **2‐BF_4_** with phenyl chloride and phenyl triflate showed less than 10% in yield of desired products with Pd(*^t^* Bu_3_P)_2_ as a catalyst due to the poor reactivity of aryl chloride and triflate in the cross‐coupling step (see Table S3), and the major byproduct observed were the hydrodefunctionalized compound and the homocoupling dimer derived from **2‐BF_4_**. A palladium catalyst featuring electron‐rich ligand SPhos‐Pd‐G3 offers a broader scope with 2‐bromopyridine, phenyl iodide, phenyl bromide, phenyl triflate, and phenyl chloride (Scheme 2b).

**Scheme 2:**
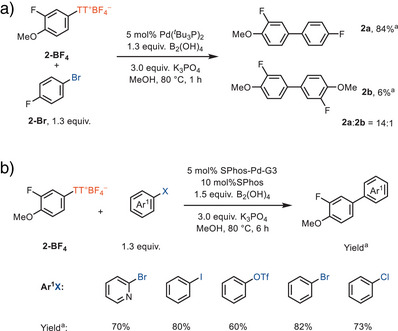
a) Product distribution. b) Palladium‐catalyzed cross‐coupling reaction of **2‐BF_4_** with different aryl(pseudo)halides. ^a^Yield determined by ^19^F NMR spectroscopic analysis with 2‐fluorotoluene as an internal standard.

A plausible catalytic cycle for this transformation is shown in Scheme 3a. In the borylation cycle, the catalytically active, monoligated palladium(0) catalyst could react with arylthianthrenium salt to form π complex intermediate **A**,^[^ ^39^ ^]^ which undergoes oxidative addition to produce the aryl palladium cation species **B**.^[^ ^36^, ^40^ ^]^ Transmetallation occurs between **B** and the diboron reagent, followed by a fast reductive elimination to generate the arylboron species **D**.^[^ ^10^, ^41^ ^]^ The borylation product **D** enters the SMC (Suzuki–Miyaura cross‐coupling) cycle and undergoes transmetallation with oxidative addition complex **F** in the presence of a base via the pre‐transmetallation complex **G** ^[^ ^42^ ^]^ to form complex **H**, which undergoes reductive elimination to form the desired biaryl product. The control experiment showed that the palladium(0)‐catalyzed borylation exhibits high selectivity toward **2‐BF_4_** in the presence of conventional aryl halides (see Figures S3−S5), which is consistent with the selective oxidative addition of arylthianthrenium salt with palladium(0). Monitoring of the cross‐coupling reaction showed that the borylation of the arylthianthrenium salt **2‐BF_4_** proceeded rapidly within 10 s, accompanied by the formation of the homocoupling dimer **2b** during this period. The subsequent SMC reaction with the residual aryl bromide **2‐Br** proceeded at a slower rate, occurring only after the arylthianthrenium salt **2‐BF_4_** was mostly consumed (Scheme 3b). Compared with bimetallic CEC reactions,^[^ ^11^ ^]^ where the cross‐selectivity is controlled by the relative rate difference for oxidative addition of aryl electrophiles with two distinct catalysts, respectively, the cross‐selectivity in our approach is determined in the first borylation cycle by a single palladium catalyst. The selective and rapid borylation facilitated by arylthianthrenium salts allows for efficient cross‐coupling with various (hetero)aryl electrophiles.

**Scheme 3:**
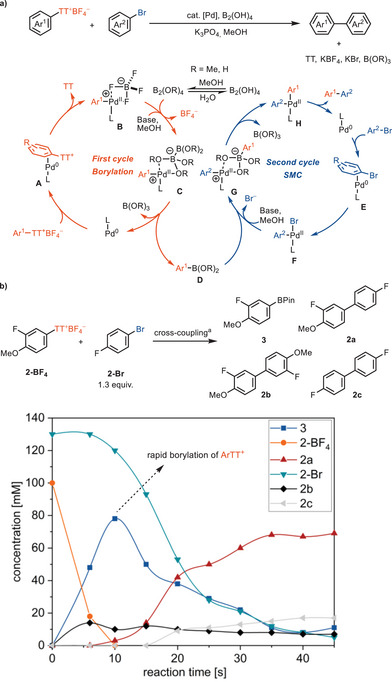
a) Proposed catalytic cycle. b) Reaction profile of cross‐coupling reaction between **2‐BF_4_** and **2‐Br**. ^a^ **2‐BF_4_** (0.05 mmol), *p*‐bromofluorobenzene **2‐Br** (1.3 equiv), B_2_(OH)_4_ (1.5 equiv), K_3_PO_4_ (3.0 equiv), Pd(*^t^* Bu_3_P)_2_ (5 mol%), MeOH (0.1 M), 80 °C. After the indicated time, pinacol (5.0 equiv) was added. Yield determined by ^19^F NMR spectroscopic analysis with 2‐fluorotoluene as an internal standard.

Investigation into the substituent effects on the oxidative addition of arylthianthrenium salts with palladium(0) via Hammett analysis shows an almost zero rho value (Scheme 4a). Given that the oxidative addition of arylthianthrenium salts by palladium(0) is irreversible,^[^ ^36^ ^]^ it can be inferred that the rate of the oxidative addition is virtually independent on the electronic properties of the substituents on the arenes, and hence the oxidative addition is sufficiently fast even for arenes with electron‐releasing groups. A radical clock experiment was performed with arylthianthrenium salt **4‐BF_4_** as a mechanistic probe to distinguish between a concerted oxidative addition and SET pathway with palladium(0) catalyst. The reaction produced noncyclized cross‐coupled products **4a** in 19% yield (Scheme 4b). The low mass balance observed is due to the formation of homocoupled by‐product **4b** derived from arylthianthrenium salt **4‐BF_4_**. No cyclized product from the trapping of an aryl radical was observed. The preliminary mechanistic data presented above is consistent with concerted oxidative addition of the arylthianthrenium salt to palladium(0).^[^ ^43^ ^]^ Given the unique electronic structure of arylthianthrenium salts, our approach enables the cross‐coupling of electron‐rich and electron‐neutral arylthianthrenium salts with a broad range of (hetero)aryl (pseudo)halides.

**Scheme 4:**
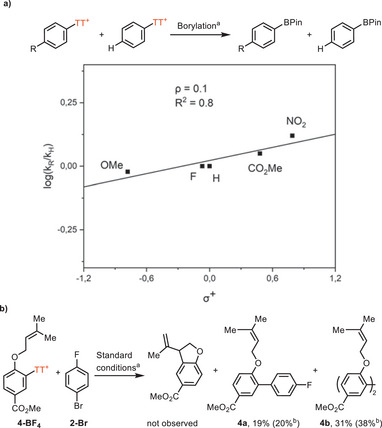
a) Hammett analysis via palladium catalyzed borylation. ^a^Arylthianthrenium salts (0.05 mmol), B_2_(OH)_4_ (0.4 equiv), Pd(*^t^* Bu_3_P)_2_ (5 mol%), MeOH (25 mM), 25 °C, 10 min, then pinacol (5.0 equiv). b) Radical clock cyclization experiment. ^a^ **4‐BF_4_** (0.05 mmol), *p*‐bromofluorobenzene **2‐Br** (1.3 equiv), B_2_(OH)_4_ (1.3 equiv), K_3_PO_4_ (3.0 equiv), Pd(*^t^* Bu_3_P)_2_ (5 mol%), MeOH (0.1 M), 80 °C, 30 min. ^b^Yield determined by ^1^H NMR spectroscopic analysis with CH_2_Br_2_ as an internal standard.

Structurally and electronically diverse aryl electrophiles can now participate in our approach as coupling partners (Scheme 5). We initially focused on the synthesis of aryl–heteroaryl structures due to the challenges by the installation of heteroaryl cores via traditional cross‐coupling reactions.^[^ ^44^ ^]^ A wide range of heteroaryl halides were successfully coupled with complex arylthianthrenium salts to provide the aryl–heteroaryl structures. For example, halopyridines featuring halides in different positions (**5**, **6**, **10**) participate well. The scope also includes other heteroaryl cores, such as 2‐thiazolyl (**7**), 5‐pyrimidyl (**8**), 2‐benzothiazolyl (**9**), 2‐quinolinyl (**11**), 8‐xanthinyl (**12**), 2‐benzofuranyl (**13**), 8‐quinolinyl (**14**), and unprotected, nitrogen‐rich 3‐chloroindazole (**16**). A weaker base Na_2_CO_3_ can avoid the competing S_N_Ar side reaction with solvent of reactive 2‐heteroaryl halide (**13**). The reaction can be used to cross‐couple heteroaryl thianthrenium salt with heteroaryl halides to afford heterobiaryl (**15**). The high functional‐group tolerance is relevant for late‐stage functionalization. Amide, ethers, nitriles, amines, halogens, and a range of basic heterocycles are well tolerated. Our approach is also successful for coupling with aryl (pseudo)halides, such as aryl chlorides (**17**, **18**, **21**, **22**), aryl bromides (**19, 20, 23**), aryl iodides (**24, 28**), and aryl triflates (**25**–**27**). We also aimed to utilize both structurally complex arylthianthrenium salts and aryl halides, which enable the linkage of two complex building blocks (**17**, **21**, **23**). The low mass balance can be explained by the formation of the homodimers derived from arylthianthrenium salts, as illustrated by substrates **24**, **25**, and **27**. Proto‐defunctionalization byproducts and residual arylboron species also accounted for some of the mass balance. Although the reported sequential C–H borylation followed by SMC reaction involves the same number of steps as our approach with C–H thianthrenation followed by a cross‐coupling reaction under reductive condition, the high selectivity for the most electron‐rich position in C–H thianthrenation results in a different constitutional isomer compared to those obtained from C–H borylation reactions, which is predominantly controlled by steric factors.^[^ ^13^, ^45^ ^]^

**Scheme 5:**
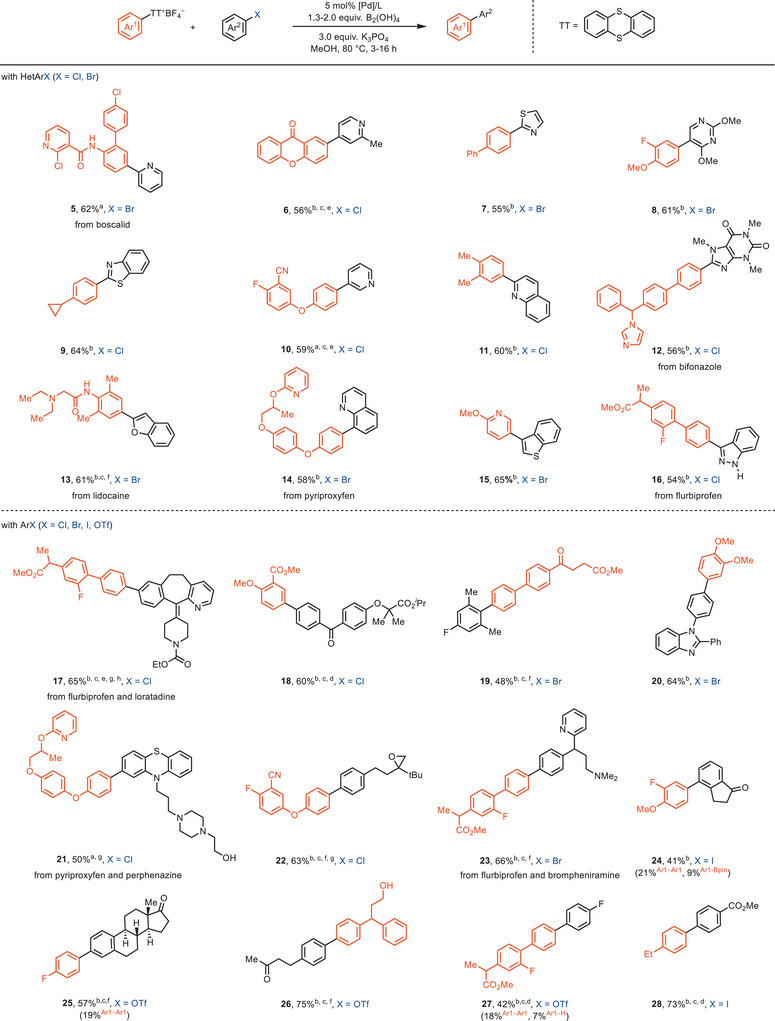
Substrate scope. ^a^Arylthianthrenium salt (0.1 mmol), aryl halide (1.0–1.3 equiv), B_2_(OH)_4_ (1.3–2.0 equiv), Pd(*^t^* Bu_3_P)_2_ (5 mol%), K_3_PO_4_ (3.0 equiv), MeOH (0.1 M), 80 °C, 3–16 h. ^b^Arylthianthrenium salt (0.1 mmol), aryl halide (1.0–1.3 equiv), B_2_(OH)_4_ (1.3–2.0 equiv), SPhos‐Pd‐G3 (5 mol%), SPhos (10 mol%), K_3_PO_4_ (3.0 equiv), MeOH (0.1 M), 80 °C, 3–16 h. ^c^Na_2_CO_3_ (3.0 equiv) instead of K_3_PO_4_ (3.0 equiv). ^d^MeOH/*n*BuOH/H_2_O = 2:3:1 instead of MeOH. ^e^MeOH/*n*BuOH = 2:3 instead of MeOH. ^f^MeOH/*n*BuOH/H_2_O = 2:3:0.5 instead of MeOH. ^g^90 °C instead of 80 °C. ^h^Reaction was performed at 1.0 mmol scale. Mass balance: yield determined by ^19^F NMR spectroscopic analysis with 2‐fluorotoluene as internal standard. ^Ar1−Ar1^Yield of the homo dimer derived from arylthianthrenium salt. Highest theoretical yield for homocoupling: 50%. ^Ar1−H^Yield of the proto‐defunctionalization byproduct derived from arylthianthrenium salt. ^Ar1−Bpin^Yield of the arylboron species derived from arylthianthrenium salt, 4.0 equiv pinacol was added to the reaction mixture after the indicated reaction time.

In conclusion, we report a robust reductive cross‐coupling reaction between complex arylthianthrenium salts and (hetero)aryl (pseudo)halides. The selective umpolung of arylthianthrenium salts into arylboronic acid with concurrent cross‐coupling enables the reaction to proceed under mild conditions. We anticipate that the late‐stage modification of arylthianthrenium salts with versatile aryl halides can serve as a valuable method for biaryl synthesis by a formal CEC reaction.

## Conflict of Interests

The authors declare no conflict of interest.

## Supporting information



Supporting Information

## Data Availability

The data that support the findings of this study are available in the Supporting Information of this article.
