# Supplementary material for: Late‐Stage Cross‐Electrophile Coupling of Arylthianthrenium Salts with (Hetero)aryl (Pseudo)halides via Palladium Catalysis
Source: Angew Chem Int Ed Engl. 2025 Apr 14;64(24):e202502441. doi: 10.1002/anie.202502441 (PMC12144872; doi:10.1002/anie.202502441)
Supplement: Supplementary file 1 — Supporting Information [file ANIE-64-e202502441-s001.pdf]

SUPPLEMENTARY INFORMATION

Late-stage cross-electrophile coupling of arylthianthrenium salts with (hetero)aryl (pseudo)halides via palladium catalysis

Yuanhao Xie[1,2], Li Zhang[1], Tobias Ritter[1]*

[1] Max-Planck-Institute für Kohlenforschung, Kaiser-Wilhelm Platz 1, D-45470 Mülheim an der Ruhr, Germany.

[2] Institute of Organic Chemistry, RWTH Aachen University, Landoltweg 1, 52074 Aachen, Germany.

*E-mail: ritter@kofo.mpg.de

TABLE OF CONTENTS

TABLE OF CONTENTS 2

MATERIALS AND METHODS 9

EXPERIMENTAL DATA 10

General procedure and reaction optimization for the formal cross-electrophile coupling (CEC) 10

General procedure for the formal CEC of arylthianthrenium salts 10

Table S1. Reaction condition optimization for the formal CEC with aryl or heteroaryl halides 11

Table S2. Reaction condition optimization for the formal CEC with 2-chlorobenzoxazole 13

Table S3. Formal cross electrophile coupling of arylthianthrenium salts with different aryl halides 14

Substrates incompatible with our formal CEC reaction 14

Formal cross-electrophile coupling of arylthianthrenium salts 15

Nefiracetam derivative **1** 15

2-(3-Fluoro-4-methoxyphenyl)pyridine (**2**) 15

3,4'-Difluoro-4-methoxy-1,1'-biphenyl (**2a**) 16

3-Fluoro-4-methoxy-1,1'-biphenyl (**2d**) 18

Boscalid derivative **5** 19

Xanthone derivative **6** 19

2-(4-Phenylphenyl)thiazole (**7**) 20

5-(3-Fluoro-4-methoxyphenyl)-2,4-dimethoxypyrimidine (**8**) 21

2-(4-Cyclopropylphenyl)-2-benzothiazole (**9**) 22

2-Fluoro-6-phenoxybenzonitrile derivative **10** 22

2-(3,4-Dimethylphenyl)quinoline (**11**) 23

Bifonazole derivative **12** 24

Lidocaine derivative **13** 25

Pyriproxyfen derivative **14** 25

2-Methoxypyridine derivative **15** 26

Flurbiprofen methyl ester derivative **16** 27

Flurbiprofen methyl ester derivative **17** (1 mmol scale) 28

Fenofibrate derivative **18** 29

Fenbufen methyl ester derivative **19** 29

1,2-Dimethoxybenzene derivative **20** 30

Pyriproxyfen derivative **21** 31

2-Fluoro-6-phenoxybenzonitrile derivative **22** 32

Flurbiprofen methyl ester derivative **23** 33

4-(3-Fluoro-4-methoxyphenyl)-2,3-dihydro-1H-inden-1-one (**24**) 34

Estrone derivative **25** 34

3,3-Diphenylpropan-1-ol derivative **26** 35
Flurbiprofen methyl ester derivative **27** 36
Methyl-4´-ethyl-(1,1´-biphenyl)-4-carboxylate (**28**) 37

Palladium catalyzed borylation of aryl thianthrenium salts.....37

2-(3-Fluoro-4-methoxyphenyl)-4,4,5,5-tetramethyl-1,3,2-dioxaborolane (**3**) 37
4-(4,4,5,5-Tetramethyl-1,3,2-dioxaborolan-2-yl)anisole (**3a**) 38
2-(4-Fluorophenyl)-4,4,5,5-tetramethyl-1,3,2-dioxaborolane (**3b**) 39
2-(Phenyl)-4,4,5,5-tetramethyl-1,3,2-dioxaborolane (**3c**) 40
Methyl 4-(4,4,5,5-tetramethyl-1,3,2-dioxaborolan-2-yl)benzoate (**3d**) 40
4,4,5,5-tetramethyl-2-(4-nitrophenyl)-1,3,2-dioxaborolane (**3e**).....41

Synthesis of arylthianthrenium salt 42

Nefiracetam-derived thianthrenium salt **1-BF$_4$**.....42
2-Fluoroanisole-derived thianthrenium salt **2-BF$_4$** 42
Anisole-derived thianthrenium salt **3a-BF$_4$**.....43
Benzene-derived thianthrenium salt **3c-BF$_4$**.....44
Methylbenzoate-derived thianthrenium salt **3d-BF$_4$**.....45
Nitrobenzene-derived thianthrenium salt **3e-BF$_4$**.....46
Radical-clock thianthrenium salt **4-BF$_4$** 46
Boscalid-derived thianthrenium salt **5-BF$_4$** 48
Xanthon-derived thianthrenium salt **6-BF$_4$** 48
Biphenyl-derived thianthrenium salt **7-BF$_4$**.....49
Cyclopropylbenzene-derived thianthrenium salt **9-BF$_4$**.....50
2-Fluoro-6-phenoxybenzonitrile-derived thianthrenium salt **10-BF$_4$** 51
o-Xylene-derived thianthrenium salt **11-BF$_4$**.....52
Bifonazole-derived thianthrenium salt **12-BF$_4$** 52
Lidocaine-derived thianthrenium salt **13-BF$_4$** 53
Pyriproxyfen-derived thianthrenium salt **14-BF$_4$**.....54
2-Methoxypyridine-derived thianthrenium salt **15-BF$_4$**.....55
Flurbiprofen methyl ester-derived thianthrenium salt **16-BF$_4$**.....56
Methyl-2-methoxybenzoate-derived thianthrenium salt **18-BF$_4$** 57
Fenbufen methyl ester-derived thianthrenium salt **19-BF$_4$**.....58
1,2-Dimethoxybenzene-derived thianthrenium salt **20-BF$_4$**.....59
Fluorobenzene-derived thianthrenium salt **25-BF$_4$** 59
3,3-Diphenylpropan-1-ol -derived thianthrenium salt **26-BF$_4$** 60
Ethylbenzene-derived thianthrenium salt **28-BF$_4$** 61

Synthesis of aryl triflates 62

Estrone-derived aryl triflate **25a**.....62

4-(3-Oxobutyl)phenyl trifluoromethanesulfonate **26a**......63

MECHANISTIC STUDIES......64

NMR Monitoring......64

Competitive borylation of arylthianthrenium salt and aryl bromide......64

Competitive borylation of arylthianthrenium salt and aryl iodide......65

Competitive borylation of arylthianthrenium salt and aryl triflate......66

Reaction profile of Pd-catalyzed formal cross electrophile coupling......67

Competition Kinetics......68

Table S4. Reaction rate ratio for competition reactions (X: yield for borylation of para-substituted arylthianthrenium salt; H: yield for borylation of benzene-derived arylthianthrenium salt)......68

Radical clock cyclization experiment......69

SPECTROSCOPIC DATA......71

^{1}H NMR of **1**......71

^{13}C NMR of **1**......72

^{1}H NMR of **2**......73

^{13}C NMR of **2**......74

^{19}F NMR of **2**......75

^{1}H NMR of **2a**......76

^{13}C NMR of **2a**......77

^{19}F NMR of **2a**......78

^{1}H NMR of **2b**......79

^{13}C NMR of **2b**......80

^{19}F NMR of **2b**......81

^{1}H NMR of **2d**......82

^{13}C NMR of **2d**......83

^{19}F NMR of **2d**......84

^{1}H NMR of **4a**......85

^{13}C NMR of **4a**......86

^{19}F NMR of **4a**......87

^{1}H NMR of **4b**......88

^{13}C NMR of **4b** 89
^{1}H NMR of **5** 90
^{13}C NMR of **5** 91
^{1}H NMR of **6** 92
^{13}C NMR of **6** 93
^{1}H NMR of **7** 94
^{13}C NMR of **7** 95
^{1}H NMR of **8** 96
^{13}C NMR of **8** 97
^{19}F NMR of **8** 98
^{1}H NMR of **9** 99
^{13}C NMR of **9** 100
^{1}H NMR of **10** 101
^{13}C NMR of **10** 102
^{19}F NMR of **10** 103
^{1}H NMR of **11** 104
^{13}C NMR of **11** 105
^{1}H NMR of **12** 106
^{13}C NMR of **12** 107
^{1}H NMR of **13** 108
^{13}C NMR of **13** 109
^{1}H NMR of **14** 110
^{13}C NMR of **14** 111
^{1}H NMR of **15** 112
^{13}C NMR of **15** 113
^{1}H NMR of **16** 114
^{13}C NMR of **16** 115
^{19}F NMR of **16** 116
^{1}H NMR of **17** 117

^{13}C NMR of **17** 118

^{19}F NMR of **17** 119

^{1}H NMR of **18** 120

^{13}C NMR of **18** 121

^{1}H NMR of **19** 122

^{13}C NMR of **19** 123

^{19}F NMR of **19** 124

^{1}H NMR of **20** 125

^{13}C NMR of **20** 126

^{1}H NMR of **21** 127

^{13}C NMR of **21** 128

^{1}H NMR of **22** 129

^{13}C NMR of **22** 130

^{19}F NMR of **22** 131

^{1}H NMR of **23** 132

^{13}C NMR of **23** 133

^{19}F NMR of **23** 134

^{1}H NMR of **24** 135

^{13}C NMR of **24** 136

^{19}F NMR of **24** 137

^{1}H NMR of **25** 138

^{13}C NMR of **25** 139

^{19}F NMR of **25** 140

^{1}H NMR of **26** 141

^{13}C NMR of **26** 142

^{1}H NMR of **27** 143

^{13}C NMR of **27** 144

^{19}F NMR of **27** 145

^{1}H NMR of **28** 146

^{13}C NMR of **28** 147

^{1}H NMR of **3** 148

^{13}C NMR of **3** 149

^{19}F NMR of **3** 150

^{11}B NMR of **3** 151

^{1}H NMR of **3a** 152

^{13}C NMR of **3a** 153

^{11}B NMR of **3a** 154

^{1}H NMR of **3b** 155

^{13}C NMR of **3b** 156

^{19}F NMR of **3b** 157

^{11}B NMR of **3b** 158

^{1}H NMR of **3c** 159

^{13}C NMR of **3c** 160

^{11}B NMR of **3c** 161

^{1}H NMR of **3d** 162

^{13}C NMR of **3d** 163

^{11}B NMR of **3d** 164

^{1}H NMR of **3e** 165

^{13}C NMR of **3e** 166

^{11}B NMR of **3e** 167

^{1}H NMR of **1-BF$_4$** 168

^{13}C NMR of **1-BF$_4$** 169

^{19}F NMR of **1-BF$_4$** 170

^{1}H NMR of **3d-BF$_4$** 171

^{13}C NMR of **3d-BF$_4$** 172

^{19}F NMR of **3d-BF$_4$** 173

^{1}H NMR of **5-BF$_4$** 174

^{13}C NMR of **5-BF$_4$** 175

^{19}F NMR of **5-BF$_4$**....176

^{1}H NMR of **25-BF$_4$**....177

^{13}C NMR of **25-BF$_4$**....178

^{19}F NMR of **25-BF$_4$**....179

REFERENCES....180

MATERIALS AND METHODS

All reactions were carried out under ambient atmosphere unless otherwise stated and monitored by thin-layer chromatography (TLC). High-resolution mass spectra were obtained using *Q Exactive Plus* from *Thermo*. Concentration under reduced pressure was performed by rotary evaporation at 25–40 °C at an appropriate pressure. Purified compounds were further dried under vacuum (10^{-6} – 10^{-3} bar). Yields refer to purified and spectroscopically pure compounds, unless otherwise stated.

Solvents

Anhydrous solvents were obtained from Phoenix Solvent Drying Systems. All deuterated solvents were purchased from Euriso-Top®.

Chromatography

Thin layer chromatography (TLC) was performed using EMD TLC plates pre-coated with 250 μm thickness silica gel 60 F_{254} plates and visualized by fluorescence quenching under UV light and $KMnO_4$ stain. Flash column chromatography was performed using silica gel (40–63 μm particle size) purchased from Geduran®.

Spectroscopy and Instruments

NMR spectra were recorded on a Bruker *Ascend*^{TM} 500 spectrometer operating at 500 MHz, 471 MHz, 126 MHz, and 126 MHz, for ^{1}H, ^{19}F, and ^{13}C acquisitions, respectively. Chemical shifts are reported in ppm with the solvent residual peak as the internal standard. For ^{1}H NMR: $CDCl_3$, δ 7.26, CD_3CN, δ 1.96, CD_2Cl_2, δ 5.32. For ^{13}C NMR: $CDCl_3$, δ 77.16, CD_3CN, δ 1.79, CD_2Cl_2, δ 53.5.[1] ^{19}F NMR spectra were referenced using a unified chemical shift scale based on the ^{1}H resonance of tetramethylsilane (1% (v/v) solution in the respective solvent). Data is reported as follows: s = singlet, d = doublet, t = triplet, q = quartet, m = multiplet, br = broad; coupling constants in Hz; integration.

Starting materials

All substrates were used as received from commercial suppliers, unless otherwise stated. Chemicals were purchased from *Sigma-Aldrich*, *TCI*, *Alfa Aesar, Abcr, or Fluorochem*.

EXPERIMENTAL DATA

General procedure and reaction optimization for the formal cross-electrophile coupling (CEC)

General procedure for the formal CEC of arylthianthrenium salts

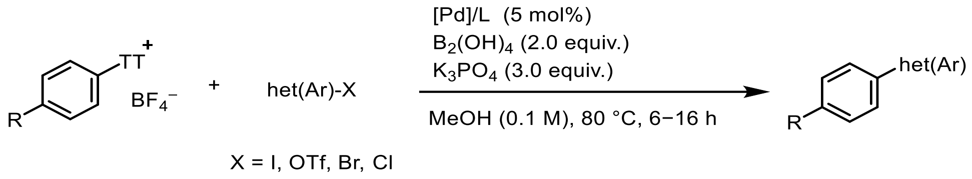

Under ambient atmosphere, a 4-mL borosilicate vial equipped with a magnetic stir bar and a Schlenk-line adapter was charged with arylthianthrenium salt (0.100 mmol, 1.00 equiv.), (het)aryl halide (if solid) (0.130 mmol, 1.30 equiv.), $B_2(OH)_4$ (17.9 mg, 0.200 mmol, 2.00 equiv.), K_3PO_4 (63.7 mg, 0.300 mmol, 3.00 equiv.). Subsequently, either the catalyst Pd(^{t}Bu$_3$P)$_2$ (2.5 mg, 5.0 μmol, 5.0 mol%) or the catalyst combination containing SPhos-Pd-G3 (3.9 mg, 5.0 μmol, 5.0 mol%) and SPhos (4.1 mg, 10 μmol, 10 mol%) was added into the vial in one portion. The vial was evacuated and backfilled with argon three times using a Schlenk line. MeOH (1 mL, *c* = 0.1 M) and (het)aryl halide (if liquid) (0.130 mmol, 1.30 equiv.) were added via syringe. The vial was sealed and then the reaction mixture was stirred vigorously at 80 °C for 6−16 h on a heating block. After the indicated time, the reaction mixture was allowed to cool to 23 °C. Then, the reaction vial was opened to air, and the resulting mixture was concentrated by rotary evaporation. The residue was purified by chromatography on silica gel to obtain the pure product.

Caution: Because the formal CEC reaction was carried out in a sealed vial at the temperatures above the boiling point of the solvent (MeOH), a blast shield must be used to minimize personal damage in case of an accident.

Table S1. Reaction condition optimization for the formal CEC with aryl or heteroaryl halides

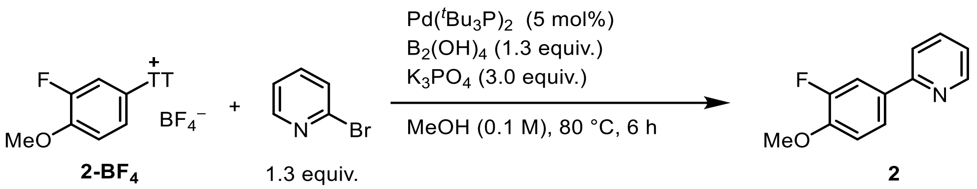

Entry	Change of reaction conditions	Yield of product[b]
1	None	82%(80%[c])
2	Change concentration to 0.05 M	81%
3	Change K_3PO_4 to KOAc	38%
4	Change K_3PO_4 to KOPiv	10%
5	Change K_3PO_4 to KF	31%
6	Change K_3PO_4 to K_2CO_3	70%
7	Change K_3PO_4 to Na_2CO_3	75%
8	Change K_3PO_4 to $NaHCO_3$	48%
9	Change MeOH to MeOH:H_2O = 9:1 (0.1M)	80%
10	Change MeOH to MeOH:*n*BuOH = 2:3 (0.1M)	72%
11	Change MeOH to MeCN	< 5%
12	Change MeOH to 2-methoxyethanol	35%
13	Change MeOH to 1,4-dioxane	< 5%
14	Change MeOH to THF	< 5%
15	Change MeOH to DMSO	< 5%
16	Change to 60 °C	75%
17	Change to 70 °C	82%
18	Change to 90 °C	80%
19	Change $B_2(OH)_4$ (1.3 equiv.) to $B_2(OH)_4$ (2.0 equiv.)	79%
20	Change $B_2(OH)_4$ (2.0 equiv.) to $B_2(OH)_4$ (3.0 equiv.)	36%
21	Change $ArTT^+BF_4^-$ to $ArTT^+NTf_2^-$	82%
22	Change $ArTT^+BF_4^-$ to $ArTT^+OTf^-$	80%
23	Change 1.3 equiv. to 1.0 equiv. 2-bromopyridine	50%
24	Change 1.3 equiv. to 1.5 equiv. 2-bromopyridine	79%
25	Change $B_2(OH)_4$ to $B_2(pin)_2$	77%
26	Change $B_2(OH)_4$ to $B_2(npg)_2$	79%
27	Change $B_2(OH)_4$ to $B_2(cat)_2$	59%

28	Change $B_2(OH)_4$ to $B_2(oct)_2$	56%

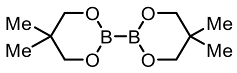

$B_2(npg)_2$

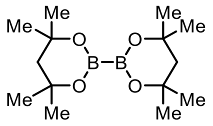

$B_2(oct)_2$

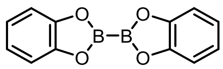

$B_2(cat)_2$

[a]Arylthianthrenim salt (0.05 mmol), 2-bromopyridine (1.3 equiv.), $B_2(OH)_4$ (1.3 equiv.), K_3PO_4 (3.0 equiv.), Pd(^{t}Bu$_3$P)$_2$ (5 mol%), MeOH (0.1 M), 80 °C, 6 h. [b]Yield determined by ^{19}F NMR. [c]Isolated yield.

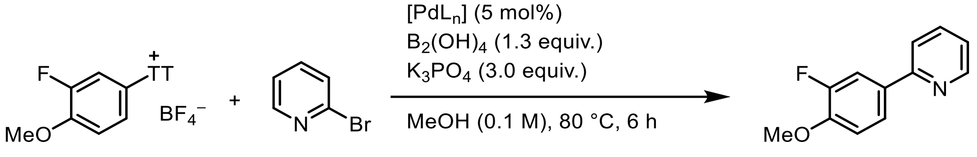

2-BF$_4$ **2**

Entry	[Pd]/L	Yield of product[b]
1	Pd(^{t}Bu$_3$P)$_2$	82%(80%[c])
2	SPhos-Pd-G$_3$/SPhos	70%
3	Pd(OAc)$_2$ (5 mol%) / PPh$_3$ (10 mol%)	33%
4	Pd(OAc)$_2$ / P(*o*-tol)$_3$	20%
5	Pd(OAc)$_2$ / dppf	59%
6	Pd(OAc)$_2$ / SPhos	68%
7	Pd(OAc)$_2$ / XPhos	38%
8	Pd(OAc)$_2$ / BrettPhos	25%
9	NiCl$_2$(PCy$_3$)$_2$	< 5%
10	NiCl$_2$(dppp)$_2$	< 5%

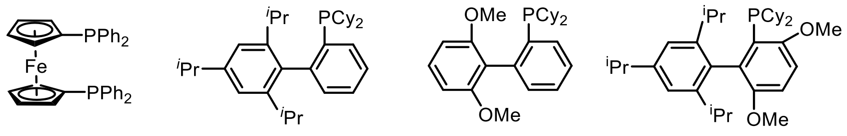

dppf XPhos SPhos BrettPhos

[a]Arylthianthrenim salt (0.05 mmol), 2-bromopyridine (1.3 equiv.), $B_2(OH)_4$ (1.3 equiv.), K_3PO_4 (3.0 equiv.), PdL$_n$ (5 mol%), MeOH (0.1 M), 80 °C, 6 h. [b]Yield determined by ^{19}F NMR. [c]Isolated yield.

Table S2. Reaction condition optimization for the formal CEC with 2-chlorobenzoxazole

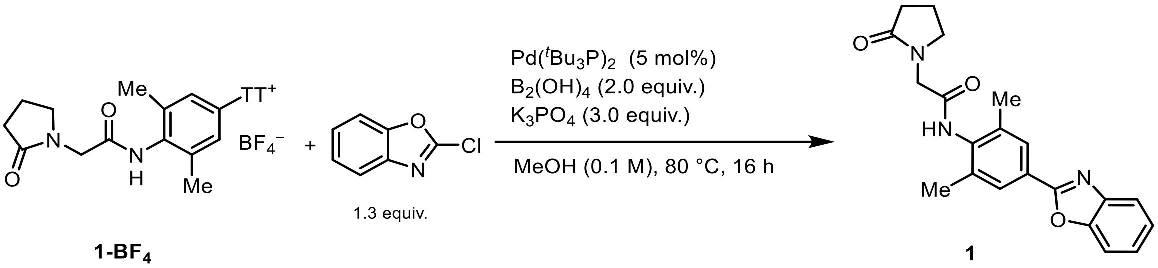

1-BF$_4$ **1**

Entry	Change of reaction conditions	Yield of product[b]
1	None	<5%
2	Na_2CO_3	31%
3	Na_2CO_3, MeOH:*n*-Butanol (2:3)	35%
4	Na_2CO_3, MeOH:*n*-Butanol (2:3), 100 °C	44%
5	Na_2CO_3, MeOH:*n*-Butanol:H_2O (2:3:1), 100 °C	67% (64%)[c]

[a]Arylthianthrenim salt (0.05 mmol), 2-chlorobenzoxazole (1.3 equiv.), $B_2(OH)_4$ (2.0 equiv.), base (3.0 equiv.), Pd(^{t}Bu$_3$P)$_2$

(5 mol%), solvent (0.1 M), 80–100 °C, 16 h. [b]Yield determined by ^{1}H NMR. [c]Isolated yield.

Table S3. Formal cross electrophile coupling of arylthianthrenium salts with different aryl halides

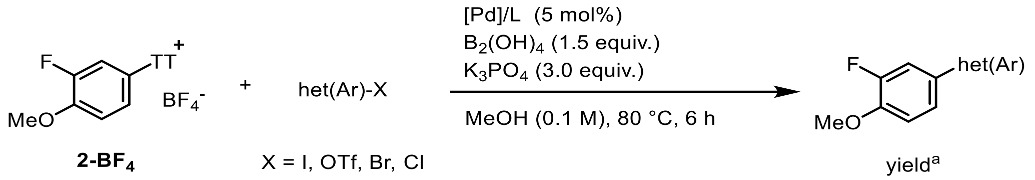

het(Ar)-X:

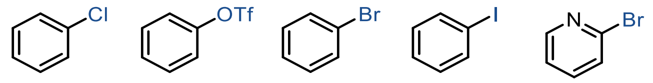

Conditions:	Cl	OTf	Br	I	2-Br-pyridine
**A**: Pd(^{t}Bu$_3$P)$_2$	9%[a]	10%[a]	84%[a]	67%[a]	82%[a]
**B**: SPhos-Pd-G3/SPhos	73%[a]	60%[a]	82%[a]	80%[a]	70%[a]

Arylthianthrenim salt (0.05 mmol), (het)aryl halides (1.3 equiv.), $B_2(OH)_4$ (1.5 equiv.), K_3PO_4 (3.0 equiv.), PdL_n (5 mol%), MeOH (0.1 M), 80 °C, 6 h. [a]Yield of cross electrophile coupling product, determined by ^{19}F NMR.

Substrates incompatible with our formal CEC reaction

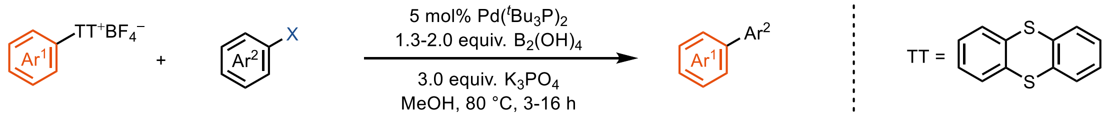

unsuccessful combinations

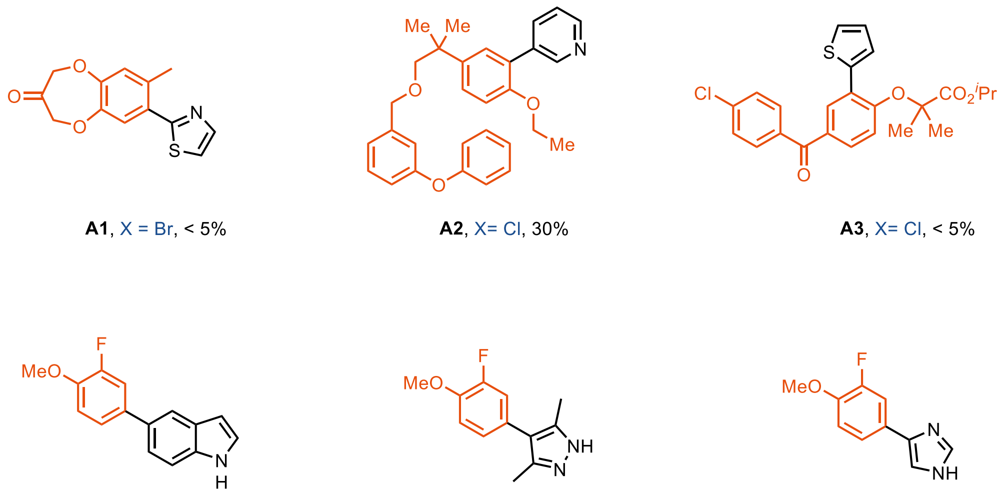

A1, X = Br, < 5%

A2, X= Cl, 30%

A3, X= Cl, < 5%

A4, X=Cl, < 5%

A5, X= Br, < 5%

A6, X=Br, < 5%

Figure S1. Substrates incompatible with our formal CEC reaction

Discussion: The formal CEC reaction with sterically encumbered *o*-substituted arylthianthrenium salts either completely shut down the reactivity (**A1**, **A3**) or results in low yield (**A2**). The CEC reaction with unprotected, nitrogen-rich heterocycles (**A4**, **A5**, **A6**) suppresses the reactivity and leads to the formation of hydrodefunctionalized byproducts from arylthianthrenium salts.

Formal cross-electrophile coupling of arylthianthrenium salts

Nefiracetam derivative 1

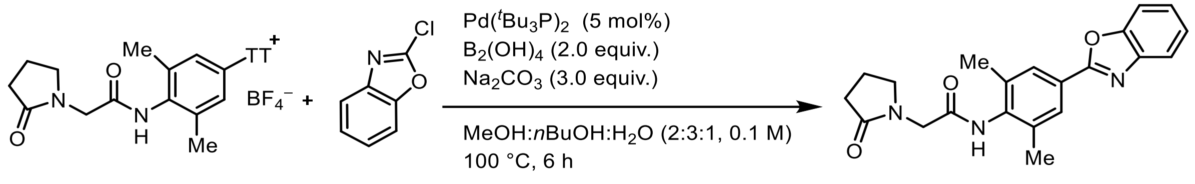

1-BF4 **1**, 64%

Under ambient atmosphere, a 4-mL borosilicate vial equipped with a magnetic stir bar and Schlenk-line adapter was charged with nefiracetam-derived thianthrenium salt **1-BF4** (54.8 mg, 0.100 mmol, 1.00 equiv.), $B_2(OH)_4$ (17.9 mg, 0.200 mmol, 2.00 equiv.), Na_2CO_3 (31.8 mg, 0.300 mmol, 3.00 equiv.), and Pd(^{t}Bu$_3$P)$_2$ (2.5 mg, 5.0 µmol, 5.0 mol%). The vial was evacuated and backfilled with argon three times using a Schlenk line. A solvent mixture of MeOH:*n*BuOH:H_2O (2:3:1, 1 mL, *c* = 0.1 M) and 2-chlorobenzoxazole (15 µL, 20 mg, 0.13 mmol, 1.3 equiv.) were added via syringe. The vial was sealed and then the reaction mixture was stirred vigorously at 100 °C for 6 h on a heating block. After the indicated time, the reaction mixture was allowed to cool to 23 °C. Then, the reaction vial was opened to air, and the resulting mixture was concentrated by rotary evaporation. The residue was purified by chromatography on silica gel eluting with DCM/MeOH (20:1, v/v) to afford 23.2 mg (64% yield) of the title compound as a pale-yellow oil.

R*f* = 0.20 (DCM/MeOH, 20:1, v/v (UV))

NMR Spectroscopy:

^{1}H NMR (500 MHz, $CDCl_3$, 25 °C, δ): 7.98 (s, 2H), 7.92 (s, 1H), 7.76 – 7.74 (m, 1H), 7.58 – 7.58 (m, 1H), 7.37 – 7.31 (m, 2H), 4.13 (s, 2H), 3.62 (t, *J* = 7.1 Hz, 2H), 2.48 (t, *J* = 7.6 Hz, 2H), 2.29 (s, 6H), 2.20 – 2.12 (m, 2H).

^{13}C NMR (126 MHz, $CDCl_3$, 25 °C, δ): 176.6, 167.1, 162.8, 150.9, 142.2, 136.8, 136.0, 127.6, 125.9, 125.2, 124.7, 120.1, 110.7, 48.9, 48.2, 30.5, 18.7, 18.4.

HRMS ESIpos (m/z) calc'd for $C_{21}H_{21}N_3O_3Na^+$ [M+Na]$^+$, 386.1475; found, 386.1472. Deviation: +0.7 ppm.

2-(3-Fluoro-4-methoxyphenyl)pyridine (2)

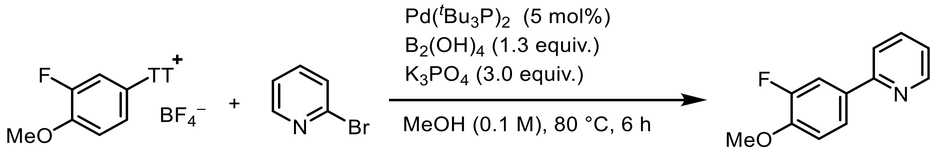

2-BF4 **2**, 80%

Under ambient atmosphere, a 4-mL borosilicate vial equipped with a magnetic stir bar and Schlenk-line adapter was charged with 2-fluoroanisole-derived thianthrenium salt **2-BF4** (42.8 mg, 0.100 mmol, 1.00 equiv.), $B_2(OH)_4$ (11.7 mg, 0.130 mmol, 1.30 equiv.), K_3PO_4 (63.7 mg, 0.300 mmol, 3.00 equiv.), and Pd(^{t}Bu$_3$P)$_2$ (2.5 mg, 5.0 µmol, 5.0 mol%). The vial was evacuated and backfilled with argon three times using

a Schlenk line. MeOH (1 mL, *c* = 0.1 M) and 2-bromopyridine (13 µL, 21 mg, 0.13 mmol, 1.3 equiv.) were added via syringe. The vial was sealed and then the reaction mixture was stirred vigorously at 80 °C for 6 h on a heating block. After the indicated time, the reaction mixture was allowed to cool to 23 °C. Then, the reaction vial was opened to air, and the resulting mixture was concentrated by rotary evaporation. The residue was purified by chromatography on silica gel eluting with hexanes/ethyl acetate (100:1 to 10:1, v/v) to afford 16.3 mg (80% yield) of the title compound as a colorless oil.

R*f* = 0.50 (hexanes/EtOAc, 5:1, v/v (UV))

NMR Spectroscopy:

^{1}H NMR (500 MHz, $CDCl_3$, 25 °C, δ): 8.64 (d, *J* = 3.9 Hz, 1H), 7.78 (dd, *J* = 12.7, 2.2 Hz, 1H), 7.75 – 7.69 (m, 2H), 7.66 (d, *J* = 8.0 Hz, 1H), 7.20 – 7.18 (m, 1H), 7.03 (t, *J* = 8.6 Hz, 1H), 3.93 (s, 3H).

^{13}C NMR (126 MHz, $CDCl_3$, 25 °C, δ): 156.0 (d, *J* = 2.4 Hz), 152.8 (d, *J* = 245.7 Hz), 149.6, 148.6 (d, *J* = 10.8 Hz), 137.0, 132.6 (d, *J* = 6.5 Hz), 122.8 (d, *J* = 3.4 Hz), 122.0, 120.0, 114.8 (d, *J* = 19.7 Hz), 113.4 (d, *J* = 2.3 Hz), 56.4.

^{19}F NMR (471 MHz, $CDCl_3$, 25 °C, δ): –135.0.

HRMS GC-EI (m/z) calc'd for $C_{12}H_{10}NOF^+$ $[M]^+$, 203.0741; found, 203.0740. Deviation: +0.5 ppm.

3,4'-Difluoro-4-methoxy-1,1'-biphenyl (2a)

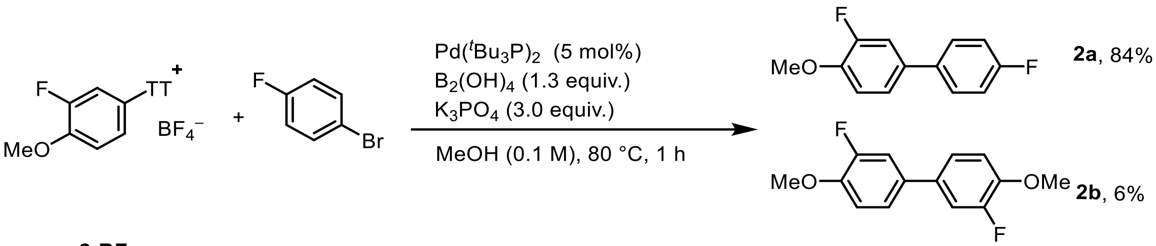

2-BF$_4$ **2a**:**2b** = 14:1

Under ambient atmosphere, a 4-mL borosilicate vial equipped with a magnetic stir bar and Schlenk-line adapter was charged with 2-fluoroanisole-derived thianthrenium salt **2-BF$_4$** (42.8 mg, 0.100 mmol, 1.00 equiv.), $B_2(OH)_4$ (11.7 mg, 0.130 mmol, 1.30 equiv.), K_3PO_4 (63.7 mg, 0.300 mmol, 3.00 equiv.). The vial was evacuated and backfilled with argon three times using a Schlenk line. MeOH (1 mL, *c* = 0.1 M) and *p*-bromofluorobenzene (14 µL, 23 mg, 0.13 mmol, 1.3 equiv.) were added via syringe. Subsequently, Pd(^{t}Bu$_3$P)$_2$ (2.5 mg, 5.0 µmol, 5.0 mol%) in MeOH (0.1 mL) was added to the reaction mixture. The vial was sealed and then the reaction mixture was stirred vigorously at 80 °C for 1 h on a heating block. After the indicated time, the reaction mixture was allowed to cool to 23 °C. Then, the reaction vial was opened to air, and the resulting mixture was concentrated by rotary evaporation. The reaction mixture was diluted with $CDCl_3$ followed by addition of 2-fluorotoluenene (22 mg, 22 µL, 0.20 mmol, 2.0 equiv.) as an internal standard. The ^{19}F NMR spectrum was measured. The yield of the product **2a** (84% yield) and the yield of the homo-coupling dimer **2b** (6% yield) were determined by ^{19}F NMR integration relative to the internal standard. Due to the excess of *p*-bromofluorobenzene used in the CEC reaction, the homo-coupling dimer of

p-bromofluorobenzene **2c** was formed with a yield of 23% as deterimined by ^{19}F NMR integration relative to the internal standard. The NMR samples were recycled and recombined with the remaining reaction mixture. The resulting mixture was concentrated by rotary evaporation. The residue was purified by chromatography on silica gel eluting with hexanes/ethyl acetate (100:1 to 20:1, v/v) to afford **2a** (18.1 mg, 82% yield) as colorless solid and **2b** (1.6 mg, 6% yield) as colorless oil.

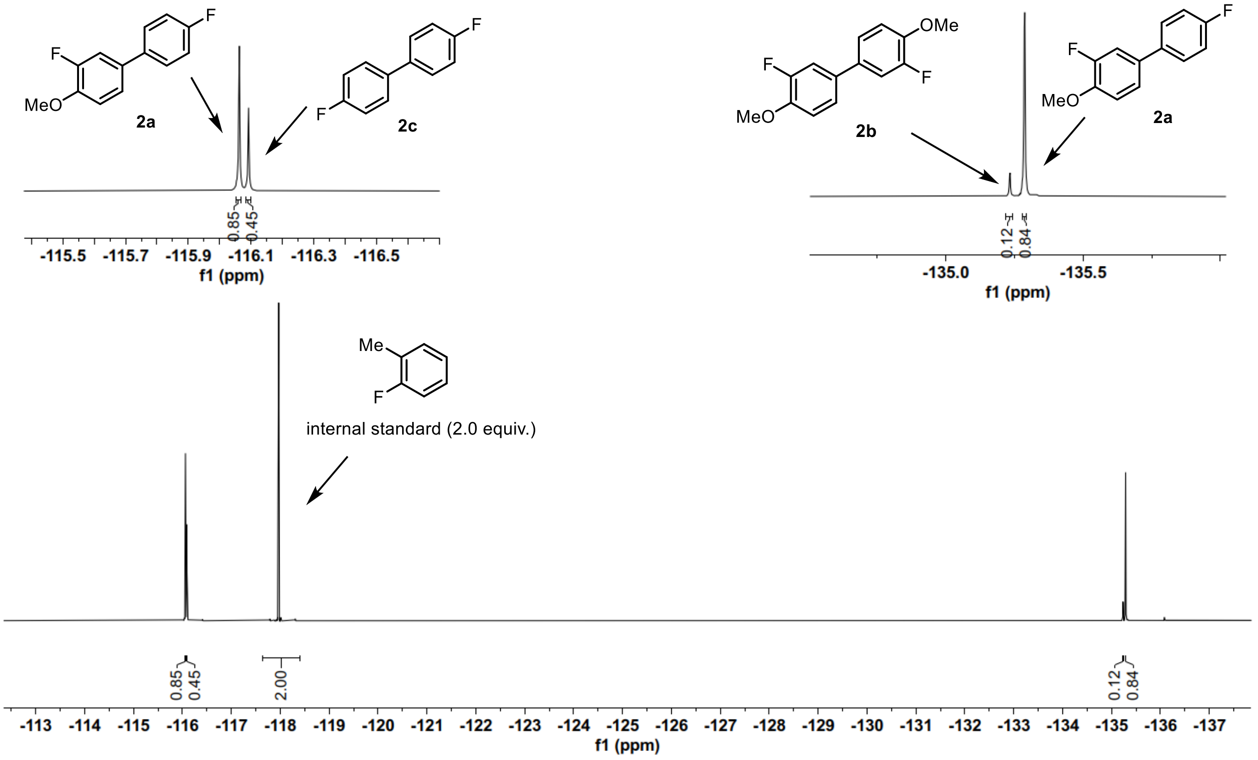

Figure S2. The ^{19}F NMR spectrum in $CDCl_3$

Compound 2a R*f* = 0.40 (hexanes/EtOAc, 20:1, v/v (UV))

Compound 2b R*f* = 0.20 (hexanes/EtOAc, 20:1, v/v (UV))

NMR Spectroscopy for 2a:

^{1}**H NMR** (500 MHz, $CDCl_3$, 25 °C, δ): 7.50 – 7.45 (m, 2H), 7.29 – 7.23 (m, 2H), 7.13 – 7.09 (m, 2H), 7.02 (t, *J* = 8.5 Hz, 1H), 3.93 (s, 3H).

^{13}**C NMR** (126 MHz, $CDCl_3$, 25 °C, δ): 162.3 (d, *J* = 251.8 Hz), 152.6 (d, *J* = 247.3 Hz), 147.0 (d, *J* = 10.9 Hz), 135.9, 133.5 (d, *J* = 6.3 Hz), 128.3 (d, *J* = 8.1 Hz), 122.5 (d, *J* = 3.4 Hz), 115.7 (d, *J* = 21.9 Hz), 114.7 (d, *J* = 19.6z Hz), 113.7 (d, *J* = 2.2 Hz), 56.3.

^{19}**F NMR** (471 MHz, $CDCl_3$, 25 °C, δ): –115.7, –134.9.

HRMS GC-EI (m/z) calc'd for $C_{13}H_{10}OF_2^+$ [M]$^+$, 220.0694; found, 220.0695. Deviation: –0.4 ppm.

NMR Spectroscopy for 2b:

^{1}**H NMR** (500 MHz, $CDCl_3$, 25 °C, δ): 7.28 – 7.21 (m, 4H), 7.01 (t, *J* = 8.8 Hz, 2H), 3.93 (s, 6H).

^{13}C NMR (126 MHz, $CDCl_3$, 25 °C, δ): 152.8 (d, *J* = 246.4 Hz), 147.1 (d, *J* = 11.0 Hz), 133.2 (dd, *J* = 1.6, 6.5 Hz), 122.4 (d, *J* = 3.2 Hz), 114.6 (d, *J* = 19.9 Hz), 113.8 (d, *J* = 2.2 Hz), 56.5.

^{19}F NMR (471 MHz, $CDCl_3$, 25 °C, δ): –134.8.

HRMS GC-EI (m/z) calc'd for $C_{14}H_{12}O_2F_2^+$ $[M]^+$, 250.0799; found, 250.0802. Deviation: –0.8 ppm.

3-Fluoro-4-methoxy-1,1'-biphenyl (2d)

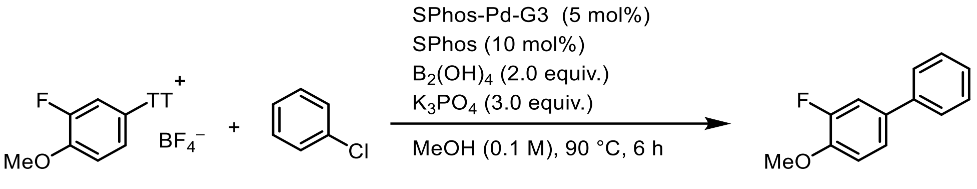

2-BF₄ **2d**, 73%

Under ambient atmosphere, a 4-mL borosilicate vial equipped with a magnetic stir bar and Schlenk-line adapter was charged with 2-fluoroanisole-derived thianthrenium salt **2-BF$_4$** (42.9 mg, 0.100 mmol, 1.00 equiv.), $B_2(OH)_4$ (17.9 mg, 0.200 mmol, 2.00 equiv.), K_3PO_4 (63.5 mg, 0.300 mmol, 3.00 equiv.), SPhos (4.1 mg, 10 µmol, 10 mol%) and SPhos-Pd-G3 (3.9 mg, 5.0 µmol, 5.0 mol%). The vial was evacuated and backfilled with argon three times using a Schlenk line. MeOH (1 mL, *c* = 0.1 M) and chlorobenzene (13 µL, 15 mg, 0.13 mmol, 1.3 equiv.) were added via syringe. The vial was sealed and then the reaction mixture was stirred vigorously at 90 °C for 6 h on a heating block. After the indicated time, the reaction mixture was allowed to cool to 23 °C. Then, the reaction vial was opened to air, and the resulting mixture was concentrated by rotary evaporation. The residue was purified by chromatography on silica gel eluting with hexanes/ethyl acetate (100:1 to 20:1, v/v) to afford 15.0 mg (73% yield) of the title compound as a colorless solid.

R_f = 0.50 (hexanes/EtOAc, 10:1, v/v (UV))

NMR Spectroscopy:

^{1}H NMR (500 MHz, $CDCl_3$, 25 °C, δ): 7.56 – 7.54 (m, 2H), 7.45 – 7.43 (m, 2H), 7.38 – 7.30 (m, 3H), 7.04 (t, *J* = 8.8 Hz, 1H), 3.94 (s, 3H).

^{13}C NMR (126 MHz, $CDCl_3$, 25 °C, δ): 152.7 (d, *J* = 245.1 Hz), 147.2 (d, *J* = 10.8 Hz), 139.8 (d, *J* = 1.8 Hz), 134.6 (d, *J* = 6.5 Hz), 129.0, 127.3, 126.8, 122.8 (d, *J* = 3.7 Hz), 114.9 (d, *J* = 19.3 Hz), 113.8 (d, *J* = 2.2 Hz), 56.5.

^{19}F NMR (471 MHz, $CDCl_3$, 25 °C, δ): –135.1.

HRMS GC-EI (m/z) calc'd for $C_{13}H_{11}OF^+$ $[M]^+$, 202.0788; found, 202.0790. Deviation: –1.0 ppm.

Boscalid derivative 5

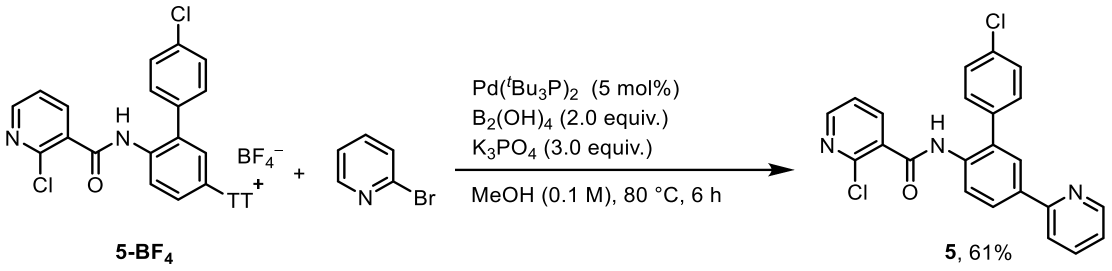

Under ambient atmosphere, a 4-mL borosilicate vial equipped with a magnetic stir bar and Schlenk-line adapter was charged with boscalid-derived thianthrenium salt **5-BF$_4$** (64.5 mg, 0.100 mmol, 1.00 equiv.), $B_2(OH)_4$ (17.9 mg, 0.200 mmol, 2.00 equiv.), K_3PO_4 (63.7 mg, 0.300 mmol, 3.00 equiv.),and Pd(^{t}Bu$_3$P)$_2$ (2.5 mg, 5.0 µmol, 5.0 mol%). The vial was evacuated and backfilled with argon three times using a Schlenk line. MeOH (1 mL, *c* = 0.1 M) and 2-bromopyridine (13 µL, 21 mg, 0.13 mmol, 1.3 equiv.) were added via syringe. The vial was sealed and then the reaction mixture was stirred vigorously at 80 °C for 6 h on a heating block. After the indicated time, the reaction mixture was allowed to cool to 23 °C. Then, the reaction vial was opened to air, and the resulting mixture was concentrated by rotary evaporation. The residue was purified by chromatography on silica gel eluting with hexanes/ethyl acetate (20:1 to 1:1, v/v) to afford 26.1 mg (61% yield) of the title compound as a colorless solid.

R*f* = 0.25 (hexanes/EtOAc, 1:1, v/v (UV))

NMR Spectroscopy:

^{1}H NMR (500 MHz, $CDCl_3$, 25 °C, δ): 8.64 (d, *J* = 4.9 Hz, 1H), 8.61(d, *J* = 8.5 Hz, 1H), 8.46 (dd, *J* = 4.9, 1.9 Hz, 1H), 8.30 (s, 1H), 8.19 (d, *J* = 7.3 Hz, 1H), 8.05 (d, *J* = 8.7 Hz, 1H), 7.98 (d, *J* = 1.7 Hz, 1H), 7.76 (d, *J* = 4.1 Hz, 2H), 7.48 – 7.35 (m, 5H), 7.25 – 7.22 (m, 1H).

^{13}C NMR (126 MHz, $CDCl_3$, 25 °C, δ): 162.5, 156.3, 151.3, 149.9, 146.8, 140.4, 137.0, 136.2, 136.1, 135.3, 134.8, 132.4, 131.1, 131.0, 129.5, 128.9, 127.3, 123.1, 122.4, 121.9, 120.5.

HRMS ESIpso (m/z) calc'd for $C_{23}H_{16}Cl_2N_3O^+$ $[M+H]^+$, 420.0664; found, 420.0666. Deviation: –0.5 ppm.

Xanthone derivative 6

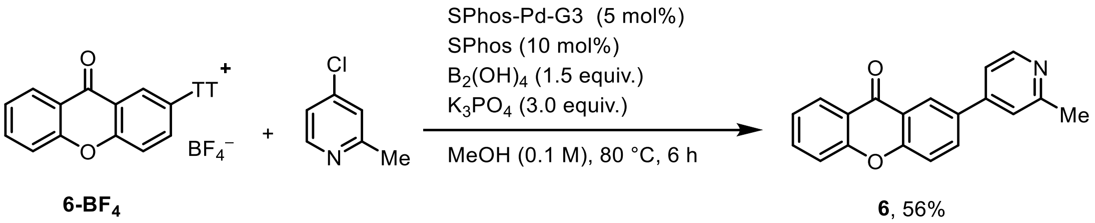

Under ambient atmosphere, a 4-mL borosilicate vial equipped with a magnetic stir bar and Schlenk-line adapter was charged with xanthone-derived thianthrenium salt **6-BF$_4$** (49.3 mg, 0.100 mmol, 1.00 equiv.), $B_2(OH)_4$ (13.4 mg, 0.150 mmol, 1.50 equiv.), K_3PO_4 (63.7 mg, 0.300 mmol, 3.00 equiv.), SPhos (4.1 mg,

10 µmol, 10 mol%) and SPhos-Pd-G3 (3.9 mg, 5.0 µmol, 5.0 mol%). The vial was evacuated and backfilled with argon three times using a Schlenk line. MeOH (1 mL, *c* = 0.1 M) and 4-chloro-2-picoline (15 µL, 17 mg, 0.13 mmol, 1.3 equiv.) was added via syringe. The vial was sealed and the then reaction mixture was stirred vigorously at 80 °C for 6 h on a heating block. After the indicated time, the reaction mixture was allowed to cool to 23 °C. Then, the reaction vial was opened to air, and the resulting mixture was concentrated by rotary evaporation. The residue was purified by chromatography on silica gel eluting with hexanes/ethyl acetate (10:1 to 1:1, v/v) to afford 16.1 mg (56% yield) of the title compound as a colorless solid.

R*f* = 0.15 (hexanes/EtOAc, 1:1, v/v (UV))

NMR Spectroscopy:

^{1}H NMR (500 MHz, $CDCl_3$, 25 °C, δ): 8.60 (d, *J* = 2.6 Hz, 1H), 8.58 (d, *J* = 5.1 Hz, 1H), 8.36 (dd, *J* = 7.97, 1.70 Hz, 1H), 7.75 (ddd, *J* = 8.7, 6.9, 1.7 Hz, 1H), 7.60 (d, *J* = 8.8 Hz, 1H), 7.52 (d, *J* = 8.6 Hz, 1H), 7.47 (s, 1H),), 7.43 – 7.38 (m, 2H), 2.65 (s, 3H).

^{13}C NMR (126 MHz, $CDCl_3$, 25 °C, δ): 176.9, 159.2, 156.4, 156.1, 149.9, 146.8, 135.1, 134.2, 133.2, 126.9, 125.1, 124.3, 122.1, 121.8, 121.1, 118.9, 118.6, 118.1, 24.6.

HRMS ESIpos (m/z) calc'd for $C_{19}H_{14}NO_2^+$ [M+H]$^+$, 288.1019; found, 288.1015. Deviation: +1.3 ppm.

2-(4-Phenylphenyl)thiazole (7)

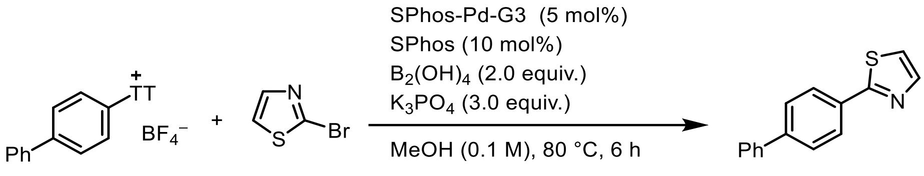

7-BF4 **7**, 55%

Under ambient atmosphere, a 4-mL borosilicate vial equipped with a magnetic stir bar and Schlenk-line adapter was charged with biphenyl-derived thianthrenium salt (45.6 mg, 0.100 mmol, 1.00 equiv.), $B_2(OH)_4$ (17.9 mg, 0.200 mmol, 2.00 equiv.), K_3PO_4 (63.7 mg, 0.300 mmol, 3.00 equiv.), SPhos (4.1 mg, 10 µmol, 10 mol%) and SPhos-Pd-G3 (3.9 mg, 5.0 µmol, 5.0 mol%). The vial was evacuated and backfilled with argon three times using a Schlenk line. MeOH (1 mL, *c* = 0.1 M) and 2-bromothiazole (12 µL, 21 mg, 0.13 mmol, 1.3 equiv.) were added via syringe. The vial was sealed and then the reaction mixture was stirred vigorously at 80 °C for 6 h on a heating block. After the indicated time, the reaction mixture was allowed to cool to 23 °C. Then, the reaction vial was opened to air, and the resulting mixture was concentrated by rotary evaporation. The residue was purified by chromatography on silica gel eluting with hexanes/ethyl acetate (50:1 to 10:1, v/v) to afford 13.2 mg (55% yield) of the title compound as a colorless solid.

R*f* = 0.40 (hexanes/EtOAc, 10:1, v/v (UV))

NMR Spectroscopy:

^{1}H NMR (500 MHz, $CDCl_3$, 25 °C, δ): 8.05 (d, *J* = 8.4 Hz, 2H), 7.89 (d, *J* = 3.4 Hz, 1H), 7.71 – 7.67 (m,

2H), 7.66 – 7.63 (m, 2H), 7.47 (t, J = 7.9 Hz, 2H), 7.41 – 7.36 (m, 1H), 7.35 (d, J = 3.4 Hz, 1H).

^{13}C NMR (126 MHz, $CDCl_3$, 25 °C, δ): 168.2, 143.9, 142.9, 140.3, 132.7, 129.0, 127.9, 127.8, 127.2, 127.1, 118.9.

HRMS GC-EI (m/z) calc'd for $C_{15}H_{11}NS^+$ $[M]^+$, 237.0606; found, 237.0609. Deviation: –1.0 ppm.

5-(3-Fluoro-4-methoxyphenyl)-2,4-dimethoxypyrimidine (8)

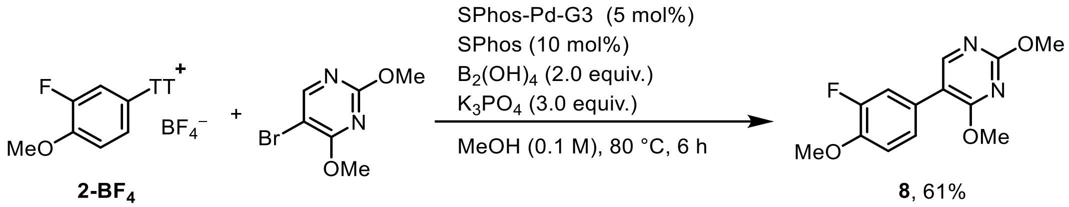

2-BF$_4$ **8**, 61%

Under ambient atmosphere, a 4-mL borosilicate vial equipped with a magnetic stir bar and Schlenk-line adapter was charged with 2-fluoroanisole-derived thianthrenium salt **2-BF$_4$** (42.8 mg, 0.100 mmol, 1.00 equiv.), 5-bromo-2,4-dimethoxypyrimidine (28.5 mg, 0.130 mmol, 1.30 equiv.), $B_2(OH)_4$ (17.9 mg, 0.200 mmol, 2.00 equiv.), K_3PO_4 (63.7 mg, 0.300 mmol, 3.00 equiv.), SPhos (4.1 mg, 10 µmol, 10 mol%) and SPhos-Pd-G3 (3.9 mg, 5.0 µmol, 5.0 mol%). The vial was evacuated and backfilled with argon three times using a Schlenk line. MeOH (1 mL, c = 0.1 M) was added via syringe. The vial was sealed and then the reaction mixture was stirred vigorously at 80 °C for 6 h on a heating block. After the indicated time, the reaction mixture was allowed to cool to 23 °C. Then, the reaction vial was opened to air, and the resulting mixture was concentrated by rotary evaporation. The residue was purified by chromatography on silica gel eluting with hexanes/ethyl acetate (10:1 to 1:1, v/v) to afford 16.2 mg (61% yield) of the title compound as a brown solid.

R$_f$ = 0.20 (hexanes/EtOAc, 10:1, v/v (UV))

NMR Spectroscopy:

^{1}H NMR (500 MHz, $CDCl_3$, 25 °C, δ): 8.22 (s, 1H), 7.27 (dd, J = 12.4, 2.3 Hz, 1H), 7.21 – 7.17 (m, 1H), 7.01 (t, J = 8.4 Hz, 1H), 4.04 – 4.01 (m, 6H), 3.92 (s, 3H).

^{13}C NMR (126 MHz, $CDCl_3$, 25 °C, δ): 168.1, 164.6, 157.4, 152.2 (d, J = 242.2 Hz), 147.4 (d, J = 11.5 Hz), 126.3 (d, J = 6.7 Hz), 124.6 (d, J = 4.3 Hz), 116.8 (d, J = 18.9 Hz), 115, 113.5 (d, J = 2.3 Hz), 56.4, 55.0, 54.3.

^{19}F NMR (471 MHz, $CDCl_3$, 25 °C, δ): –135.0.

HRMS GC-EI (m/z) calc'd for $C_{13}H_{13}N_2O_3F^+$ $[M]^+$, 264.0904; found, 264.0903. Deviation: +0.5 ppm.

2-(4-Cyclopropylphenyl)-2-benzothiazole (9)

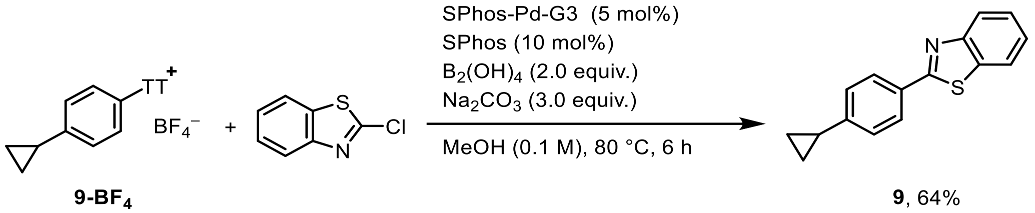

Under ambient atmosphere, a 4-mL borosilicate vial equipped with a magnetic stir bar and Schlenk-line adapter was charged with cyclopropylbenzene-derived thianthrenium salt **9-BF₄** (42.0 mg, 0.100 mmol, 1.00 equiv.), $B_2(OH)_4$ (17.9 mg, 0.200 mmol, 2.00 equiv.), Na_2CO_3 (31.8 mg, 0.300 mmol, 3.00 equiv.), SPhos (4.1 mg, 10 µmol, 10 mol%) and SPhos-Pd-G3 (3.9 mg, 5.0 µmol, 5.0 mol%). The vial was evacuated and backfilled with argon three times using a Schlenk line. MeOH (1 mL, *c* = 0.1 M) and 2-chlorobenzothiazole (16 µL, 22 mg, 0.13 mmol, 1.3 equiv.) were added via syringe. The vial was sealed and then the reaction mixture was stirred vigorously at 80 °C for 6 h on a heating block. After the indicated time, the reaction mixture was allowed to cool to 23 °C. Then, the reaction vial was opened to air, and the resulting mixture was concentrated by rotary evaporation. The residue was purified by chromatography on silica gel eluting with hexanes/ethyl acetate (100:1 to 10:1, v/v) to afford 16.1 mg (64% yield) of the title compound as a colorless solid.

$\mathbf{R}_f$ = 0.30 (hexanes/EtOAc, 10:1, v/v (UV))

NMR Spectroscopy:

^{1}H NMR (500 MHz, $CDCl_3$, 25 °C, δ): 8.05 (d, *J* = 8.4 Hz, 1H), 7.98 (d, *J* = 8.2 Hz, 2H), 7.89 (d, *J* = 8.1 Hz, 1H), 7.48 (t, *J* = 7.7 Hz, 1H), 7.37 (t, *J* = 7.1 Hz, 1H), 7.17 (d, *J* = 8.8 Hz, 2H), 1.96 (tt, *J* = 8.5, 5.3 Hz, 1H), 1.08 – 1.02 (m, 2H), 0.82 – 0.77 (m, 2H).

^{13}C NMR (126 MHz, $CDCl_3$, 25 °C, δ): 168.3, 154.3, 147.9, 135.1, 131.0, 127.7, 126.4, 126.2, 125.1, 123.2, 121.7, 15.8, 10.2.

HRMS ESIpos (m/z) calc'd for $C_{16}H_{14}NS^+$ $[M+H]^+$, 252.0841; found, 252.0842. Deviation: –0.4 ppm.

2-Fluoro-6-phenoxybenzonitrile derivative 10

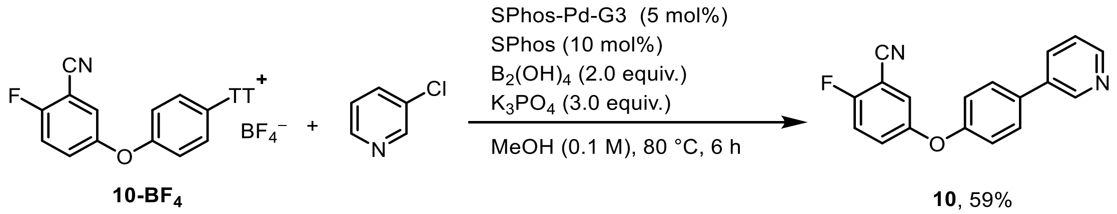

Under ambient atmosphere, a 4-mL borosilicate vial equipped with a magnetic stir bar and Schlenk-line adapter was charged with 2-fluoro-5-phenoxybenzonitrile-derived thianthrenium salt **10-BF₄** (51.6 mg, 0.100 mmol, 1.00 equiv.), $B_2(OH)_4$ (17.9 mg, 0.200 mmol, 2.00 equiv.), K_3PO_4 (63.7 mg, 0.300 mmol, 3.00 equiv.), SPhos (4.1 mg, 10 µmol, 10 mol%) and SPhos-Pd-G3 (3.9 mg, 5.0 µmol, 5.0 mol%). The vial was

evacuated and backfilled with argon three times using a Schlenk line. MeOH (1 mL, *c* = 0.1 M) and 3-chloropyridine (12 µL, 15 mg, 0.13 mmol, 1.3 equiv.) were added via syringe. The vial was sealed and then the reaction mixture was stirred vigorously at 80 °C for 6 h on a heating block. After the indicated time, the reaction mixture was allowed to cool to 23 °C. Then, the reaction vial was opened to air, and the resulting mixture was concentrated by rotary evaporation. The residue was purified by chromatography on silica gel eluting with hexanes/ethyl acetate (10:1 to 1:1, v/v) to afford 17.0 mg (59% yield) of the title compound as a colorless solid.

R*f* = 0.20 (hexanes/EtOAc, 1:1, v/v (UV))

NMR Spectroscopy:

^{1}H NMR (500 MHz, $CDCl_3$, 25 °C, δ): 8.84 (d, *J* = 2.0 Hz, 1H), 8.62 (dd, *J* = 5.2, 1.5 Hz, 1H), 7.87 (dt, *J* = 7.9, 1.8 Hz, 1H), 7.66 – 7.61 (m, 2H), 7.46 (td, *J* = 8.8, 6.2 Hz, 1H), 7.39 (dd, *J* = 7.9, 4.9 Hz, 1H), 7.24 – 7.20 (m, 2H), 6.93 (t, *J* = 7.9 Hz, 1H), 6.71 (d, *J* = 9.2 Hz, 1H).

^{13}C NMR (126 MHz, $CDCl_3$, 25 °C, δ): 164.4 (d, *J* = 264.1 Hz), 160.8 (d, *J* = 4.2 Hz), 154.8, 148.6 (d, *J* = 72.3 Hz), 135.6, 135.5, 135.1 (d, *J* = 10.5 Hz), 134.4, 129.2, 213.8, 121.0, 112.3 (d, *J* = 3.2 Hz), 111.1, 110.2 (d, *J* = 19.5 Hz), 94.1 (d, *J* = 18.6 Hz),

^{19}F NMR (471 MHz, $CDCl_3$, 25 °C, δ): –104.3.

HRMS ESIpos (m/z) calc'd for $C_{18}H_{12}N_2OF^+$ $[M+H]^+$, 291.0928; found, 291.0924. Deviation: +1.2 ppm.

2-(3,4-Dimethylphenyl)quinoline (11)

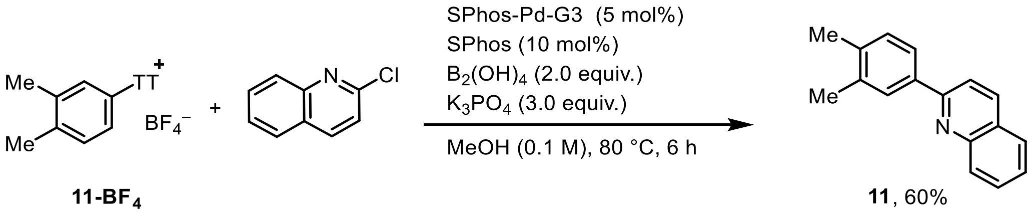

11-BF$_4$ **11**, 60%

Under ambient atmosphere, a 4-mL borosilicate vial equipped with a magnetic stir bar and Schlenk-line adapter was charged with *o*-xylene-derived thianthrenium salt **11-BF$_4$** (40.8 mg, 0.100 mmol, 1.00 equiv.), $B_2(OH)_4$ (17.9 mg, 0.200 mmol, 2.00 equiv.), K_3PO_4 (63.7 mg, 0.300 mmol, 3.00 equiv.), SPhos (4.1 mg, 10 µmol, 10 mol%) and SPhos-Pd-G3 (3.9 mg, 5.0 µmol, 5.0 mol%). The vial was evacuated and backfilled with argon three times using a Schlenk line. MeOH (1 mL, *c* = 0.1 M) and 2-chloroquinoline (17 µL, 21 mg, 0.13 mmol, 1.3 equiv.) were added via syringe. The vial was sealed and then the reaction mixture was stirred vigorously at 80 °C for 6 h on a heating block. After the indicated time, the reaction mixture was allowed to cool to 23 °C. Then, the reaction vial was opened to air, and the resulting mixture was concentrated by rotary evaporation. The residue was purified by chromatography on silica gel eluting with hexanes/ethyl acetate (100:1 to 50:1, v/v) to afford 14.0 mg (60% yield) of the title compound as a colorless solid.

R*f* = 0.36 (hexanes/EtOAc, 10:1, v/v (UV))

NMR Spectroscopy:

^{1}H NMR (500 MHz, $CDCl_3$, 25 °C, δ): 8.19 (d, *J* = 8.9 Hz, 1H), 8.16 (d, *J* = 8.4 Hz, 1H), 7.99 (s, 1H), 7.90 – 7.85 (m, 2H), 7.82 (d, *J* = 8.9 Hz, 1H), 7.72 (ddd, *J* = 8.2, 7.3, 1.5 Hz, 1H), 7.51 (td, *J* = 7.2, 1.1 Hz, 1H), 7.29 (d, *J* = 7.6 Hz, 1H), 2.39 (s, 3H), 2.35 (s, 3H).

^{13}C NMR (126 MHz, $CDCl_3$, 25 °C, δ): 157.7, 148.5, 138.3, 137.4, 137.2, 136.7, 130.3, 129.8, 129.7, 128.8, 127.6, 127.2, 126.2, 125.1, 119.1, 20.1, 19.8.

HRMS GC-EI (m/z) calc'd for $C_{17}H_{15}N^{+}$ $[M]^{+}$, 233.1198; found, 233.1197. Deviation: +0.8 ppm.

Bifonazole derivative 12

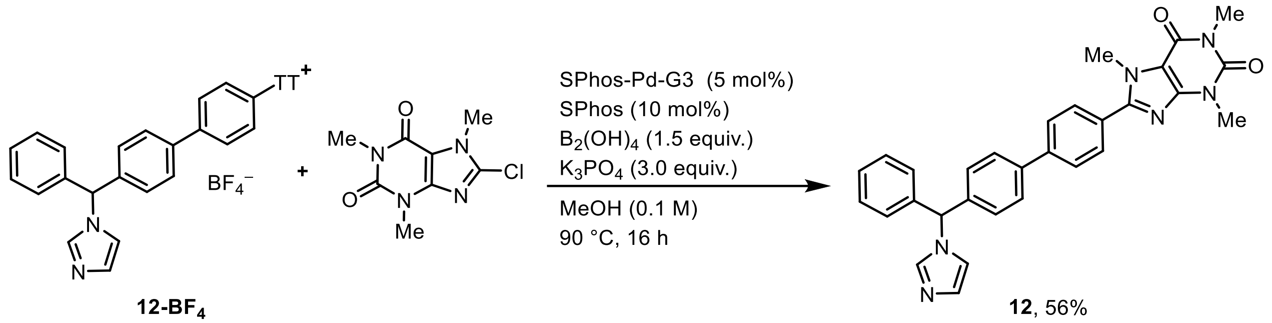

Under ambient atmosphere, a 4-mL borosilicate vial equipped with a magnetic stir bar and Schlenk-line adapter was charged with bifonazole-derived thianthrenium salt **12-BF₄** (61.2 mg, 0.100 mmol, 1.00 equiv.), 8-chloro-1,3,7-trimethyl-3,7-dihydro-1*H*-purine-2,6-dione (29.7 mg, 0.130 mmol, 1.30 equiv.), $B_2(OH)_4$ (13.4 mg, 0.150 mmol, 1.50 equiv.), K_3PO_4 (63.7 mg, 0.300 mmol, 3.00 equiv.), SPhos (4.1 mg, 10 µmol, 10 mol%) and SPhos-Pd-G3 (3.9 mg, 5.0 µmol, 5.0 mol%). The vial was evacuated and backfilled with argon three times using a Schlenk line. MeOH (1 mL, *c* = 0.1 M) was added via syringe. The vial was sealed and then the reaction mixture was stirred vigorously at 90 °C for 16 h on a heating block. After the indicated time, the reaction mixture was allowed to cool to 23 °C. Then, the reaction vial was opened to air, and the resulting mixture was concentrated by rotary evaporation. The residue was purified by chromatography on silica gel eluting with DCM/MeOH (50:1 to 30:1, v/v) to afford 28.1 mg (56% yield) of the title compound as a colorless oil.

R*f* = 0.40 (DCM/MeOH, 10:1, v/v (UV))

NMR Spectroscopy:

^{1}H NMR (500 MHz, $CDCl_3$, 25 °C, δ): 7.79 (d, *J* = 8.1 Hz, 2H), 7.73 (d, *J* = 8.7 Hz, 2H), 7.43 – 7.37 (m, 3H), 7.23 (d, *J* = 7.7 Hz, 2H), 7.20 – 7.15 (m, 3H), 6.94 (s, 1H), 6.63 (s, 1H), 4.11 (s, 3H), 3.64 (s, 3H), 3.44 (s, 3H).

^{13}C NMR (126 MHz, $CDCl_3$, 25 °C, δ): 155.7, 151.8, 151.7, 148.4, 142.0, 140.6, 137.9, 137.8, 129.8, 129.4, 129.2, 128.8, 128.2, 128.0, 127.8, 127.7, 126.0, 120.1, 108.8, 65.7, 34.1, 26.9, 28.1.

HRMS ESIpos (m/z) calc'd for $C_{30}H_{25}N_6O_2^+$ $[M+H]^+$, 503.2190; found, 503.2187. Deviation: +0.7 ppm.

Lidocaine derivative 13

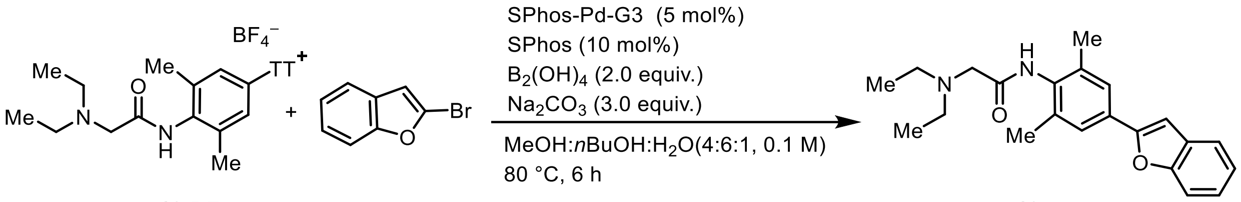

13-BF₄ **13**, 61%

Under ambient atmosphere, a 4-mL borosilicate vial equipped with a magnetic stir bar and Schlenk-line adapter was charged with lidocaine-derived thianthrenium salt **13-BF₄** (53.6 mg, 0.100 mmol, 1.00 equiv.), 2-bromobenzofuran (25.6 mg, 0.130 mmol, 1.30 equiv.), $B_2(OH)_4$ (17.9 mg, 0.200 mmol, 2.00 equiv.), Na_2CO_3 (31.8 mg, 0.300 mmol, 3.00 equiv.), SPhos (4.1 mg, 10 μmol, 10 mol%) and SPhos-Pd-G3 (3.9 mg, 5.0 μmol, 5.0 mol%). The vial was evacuated and backfilled with argon three times using a Schlenk line. A solvent mixture of MeOH:*n*BuOH:H_2O (4:6:1, 1 mL, *c* = 0.1 M) was added via syringe. The vial was sealed and then the reaction mixture was stirred vigorously at 80 °C for 6 h on a heating block. After the indicated time, the reaction mixture was allowed to cool to 23 °C. Then, the reaction vial was opened to air, and the resulting mixture was concentrated by rotary evaporation. The residue was purified by chromatography on silica gel eluting with hexanes/ethyl acetate (10:1 to 1:1, v/v) to afford 22.0 mg (61% yield) of the title compound as a yellow oil.

R_f = 0.20 (hexanes/EtOAc, 1:1, v/v (UV))

NMR Spectroscopy:

^{1}H NMR (500 MHz, $CDCl_3$, 25 °C, δ): 8.99 (s, 1H), 7.60 (s, 2H), 7.57 (d, *J* = 7.6 Hz, 1H), 7.51 (d, *J* = 8.5 Hz, 1H), 7.29 – 7.25 (m, 1H), 7.24 – 7.20 (m, 1H), 6.98 (s, 1H), 3.24 (s, 2H), 2.71 (q, *J* = 7.2 Hz, 4H), 2.31 (s, 6H), 1.16 (t, *J* = 6.9 Hz, 6H).

^{13}C NMR (126 MHz, $CDCl_3$, 25 °C, δ): 170.5, 155.8, 154.9, 135.7, 134.6, 129.4, 129.1, 124.9, 124.3, 123.1, 120.9, 111.2, 101.4, 54.7, 49.1, 18.9, 12.8.

HRMS ESIpos (m/z) calc'd for $C_{22}H_{27}N_2O_2^+$ $[M+H]^+$, 351.2067; found, 351.2062. Deviation: +1.4 ppm.

Pyriproxyfen derivative 14

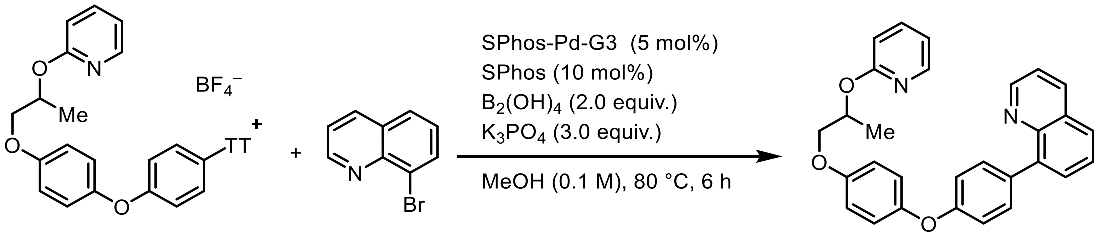

14-BF₄ **14**, 58%

Under ambient atmosphere, a 4-mL borosilicate vial equipped with a magnetic stir bar and Schlenk-line

adapter was charged with pyriproxyfen-derived thianthrenium salt **14-BF$_4$** (62.1 mg, 0.100 mmol, 1.00 equiv.), $B_2(OH)_4$ (17.9 mg, 0.200 mmol, 2.00 equiv.), K_3PO_4 (63.7 mg, 0.300 mmol, 3.00 equiv.), SPhos (4.1 mg, 10 µmol, 10 mol%) and SPhos-Pd-G3 (3.9 mg, 5.0 µmol, 5.0 mol%). The vial was evacuated and backfilled with argon three times using a Schlenk line. MeOH (1 mL, *c* = 0.1 M) and 8-bromoquinoline (17 µL, 27 mg, 0.13 mmol, 1.3 equiv.), were added via syringe. The vial was sealed and then the reaction mixture was stirred vigorously at 80 °C for 6 h on a heating block. After the indicated time, the reaction mixture was allowed to cool to 23 °C. Then, the reaction vial was opened to air, and the resulting mixture was concentrated by rotary evaporation. The residue was purified by chromatography on silica gel eluting with hexanes/ethyl acetate (10:1 to 5:1, v/v) to afford 26.0 mg (58% yield) of the title compound as a yellow solid.

R_f = 0.20 (hexanes/EtOAc, 5:1, v/v (UV))

NMR Spectroscopy:

^{1}H NMR (500 MHz, $CDCl_3$, 25 °C, δ): 8.97 (dd, *J* = 4.3, 1.8 Hz, 1H), 8.20 (dd, *J* = 8.2, 1.6 Hz, 1H), 8.16 (dd, *J* = 5.3, 1.4 Hz, 1H), 7.81 (dd, *J* = 8.3, 1.4 Hz, 1H), 7.72 (dd, *J* = 7.1, 1.5 Hz, 1H), 7.67 – 7.62 (m, 2H), 7.62 – 7.54 (m, 2H), 7.42 (dd, *J* = 8.3, 4.1 Hz, 1H), 7.09 – 7.03 (m, 4H), 6.98 – 6.94 (m, 2H), 6.88 – 6.84 (m, 1H), 6.75 (d, *J* = 8.3 Hz, 1H), 5.64 – 5.55 (m, 1H), 4.21 (dd, *J* = 9.9, 5.3 Hz, 1H), 4.09 (dd, *J* = 9.7, 5.1 Hz, 1H), 1.49 (d, *J* = 6.2 Hz, 3H).

^{13}C NMR (126 MHz, $CDCl_3$, 25 °C, δ): 163.3, 158.3, 155.5, 150.3, 150.2, 146.9, 146.2, 140.4, 138.8, 136.4, 133.7, 131.9, 130.3, 128.9, 127.4, 126.4, 121.4, 121.1, 116.9, 116.8, 115.9, 118.2, 71.2, 69.4, 17.2.

HRMS ESIpos (m/z) calc'd for $C_{29}H_{25}N_2O_3^+$ $[M+H]^+$, 449.1859; found, 449.1857. Deviation: +0.6 ppm.

2-Methoxypyridine derivative 15

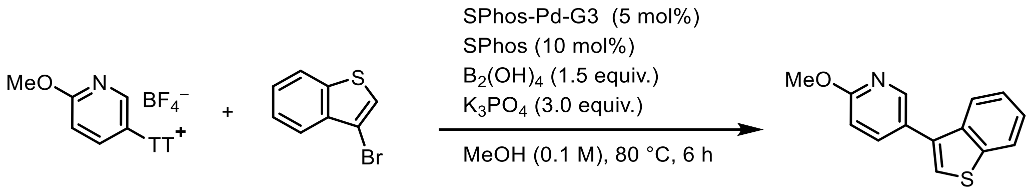

15-BF$_4$ **15**, 65%

Under ambient atmosphere, a 4-mL borosilicate vial equipped with a magnetic stir bar and Schlenk-line adapter was charged with 2-methoxypyridine-derived thianthrenium salt **15-BF$_4$** (41.1 mg, 0.100 mmol, 1.00 equiv.), $B_2(OH)_4$ (13.4 mg, 0.150 mmol, 1.50 equiv.), K_3PO_4 (63.7 mg, 0.300 mmol, 3.00 equiv.), SPhos (4.1 mg, 10 µmol, 10 mol%) and SPhos-Pd-G3 (3.9 mg, 5.0 µmol, 5.0 mol%). The vial was evacuated and backfilled with argon three times using a Schlenk line. MeOH (1 mL, *c* = 0.1 M) and 3-bromo-1-benzothiophene (17 µL, 28 mg, 0.13 mmol, 1.3 equiv.), were added via syringe. The vial was sealed and then the reaction mixture was stirred vigorously at 80 °C for 6 h on a heating block. After the indicated time, the reaction mixture was allowed to cool to 23 °C. Then, the reaction vial was opened to air, and the resulting mixture was concentrated by rotary evaporation. The residue was purified by chromatography on silica gel

eluting with hexanes/ethyl acetate (100:1, v/v) to afford 15.6 mg (65% yield) of the title compound as a colorless solid.

R*f* = 0.4 (hexanes/EtOAc, 10:1, v/v (UV))

NMR Spectroscopy:

^{1}H NMR (500 MHz, $CDCl_3$, 25 °C, δ): 8.39 (d, *J* = 2.4 Hz, 1H), 7.95 – 7.90 (m, 1H), 7.86 – 7.82 (m, 1H), 7.80 (dd, *J* = 8.7, 2.6 Hz, 1H), 7.43 – 7.39 (m, 2H), 7.39 (s, 1H), 6.88 (d, *J* = 8.7 Hz, 1H), 4.02 (s, 3H).

^{13}C NMR (126 MHz, $CDCl_3$, 25 °C, δ): 163.7, 146.3, 140.8, 139.2, 138.0, 134.4, 125.3, 124.7, 124.6, 123.7, 123.2, 122.6, 111.0, 53.8.

HRMS GC-EI (m/z) calc'd for $C_{14}H_{11}N_1O_1S_1^+$ $[M]^+$, 241.0556; found, 241.0556. Deviation: –0.2 ppm.

Flurbiprofen methyl ester derivative 16

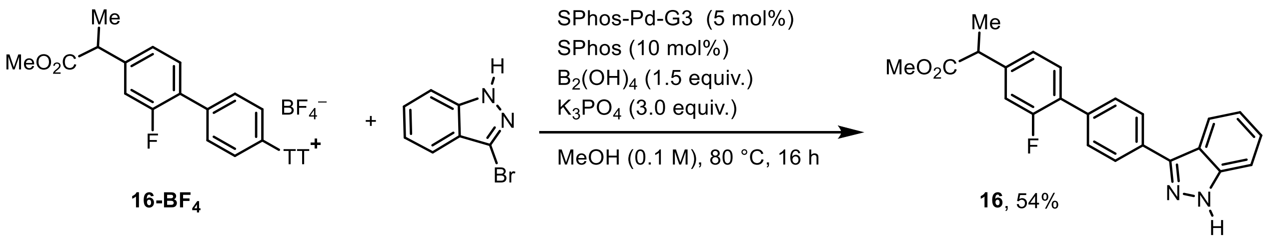

Under ambient atmosphere, a 4-mL borosilicate vial equipped with a magnetic stir bar and Schlenk-line adapter was charged with flurbiprofen methyl ester-derived thianthrenium salt **16-BF$_4$** (56.1 mg, 0.100 mmol, 1.00 equiv.), 3-bromoindazole (25.6 mg, 0.13 mmol, 1.3 equiv.), $B_2(OH)_4$ (13.4 mg, 0.150 mmol, 1.50 equiv.), K_3PO_4 (63.7 mg, 0.300 mmol, 3.00 equiv.), SPhos (4.1 mg, 10 µmol, 10 mol%) and SPhos-Pd-G3 (3.9 mg, 5.0 µmol, 5.0 mol%). The vial was evacuated and backfilled with argon three times using a Schlenk line. MeOH (1 mL, *c* = 0.1 M) was added via syringe. The vial was sealed and then the reaction mixture was stirred vigorously at 80 °C for 16 h on a heating block. After the indicated time, the reaction mixture was allowed to cool to 23 °C. Then, the reaction vial was opened to air, and the resulting mixture was concentrated by rotary evaporation. The residue was purified by chromatography on silica gel eluting with hexanes/ethyl acetate (10:1 to 2:1, v/v) to afford 20.2 mg (54% yield) of the title compound as a yellow oil.

R*f* = 0.2 (hexanes/EtOAc, 5:1, v/v (UV))

NMR Spectroscopy:

^{1}H NMR (500 MHz, $CDCl_3$, 25 °C, δ): 8.10 – 8.04 (m, 3H), 7.71 (d, *J* = 7.1 Hz, 2H), 7.49 – 7.44 (m, 1H), 7.44 – 7.41 (m, 2H), 7.28 – 7.24 (m, 1H), 7.20 – 7.14 (m, 2H), 3.79 (q, *J* = 7.2 Hz, 1H), 3.72 (s, 3H), 1.53 (d, *J* = 6.8 Hz, 3H).

^{13}C NMR (126 MHz, $CDCl_3$, 25 °C, δ): 174.6, 159.9 (d, *J* = 249.1 Hz), 144.7, 142.3 (d, *J* = 7.3 Hz), 141.6, 135.8, 132.0, 130.8 (d, *J* = 4.0 Hz), 129.7 (d, *J* = 3.2 Hz), 128.0, 127.8, 127.5 (d, *J* = 13.0 Hz), 123.8 (d, *J* = 3.6 Hz), 122.1, 121.4, 120.8, 115.5 (d, *J* = 25.2 Hz), 110.8, 52.4, 45.1, 18.6.

^{19}F NMR (471 MHz, $CDCl_3$, 25 °C, δ): –117.2.

HRMS GC-EI (m/z) calc'd for $C_{23}H_{19}N_2O_2F_1^+$ $[M]^+$, 374.1425; found, 374.1427. Deviation: –0.6 ppm.

Flurbiprofen methyl ester derivative 17 (1 mmol scale)

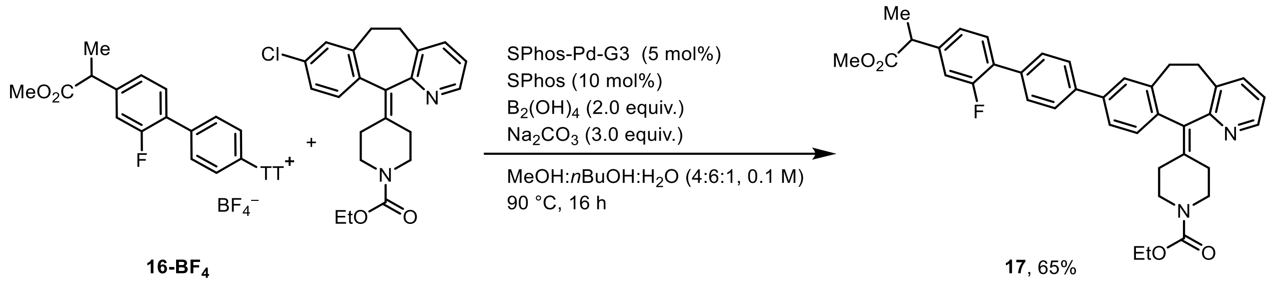

16-BF$_4$ **17**, 65%

Under ambient atmosphere, a 20-mL borosilicate vial equipped with a magnetic stir bar and Schlenk-line adapter was charged with flurbiprofen methyl ester-derived thianthrenium salt **16-BF$_4$** (561 mg, 1.00 mmol, 1.00 equiv.), loratadine (460 mg, 1.20 mmol, 1.20 equiv.), $B_2(OH)_4$ (179 mg, 2.00 mmol, 2.00 equiv.), Na_2CO_3 (318 mg, 3.00 mmol, 3.00 equiv.), SPhos (41.0 mg, 0.100 mmol, 10.0mol%) and SPhos-Pd-G3 (39.0 mg, 50.0 µmol, 5.00 mol%). A solvent mixture of MeOH:*n*BuOH:H_2O (4:6:1, 10 mL, *c* = 0.1 M) was added via syringe. The vial was sealed and then the reaction mixture was stirred vigorously at 90 °C for 16 h on a heating block. After the indicated time, the reaction mixture was allowed to cool to 23 °C. Then, the reaction vial was opened to air, and the resulting mixture was concentrated by rotary evaporation. The residue was purified by chromatography on silica gel eluting with hexanes/ethyl acetate (10:1 to 1:2, v/v) to afford 391 mg (65% yield) of the title compound as a brown oil.

R_f = 0.20 (hexanes/EtOAc, 1:1, v/v (UV))

NMR Spectroscopy:

^{1}H NMR (500 MHz, $CDCl_3$, 25 °C, δ): 8.41 (dd, *J* = 4.8, 1.6 Hz, 1H), 7.65 – 7.57 (m, 4H), 7.48 – 7.40 (m, 4H), 7.30 – 7.27 (m, 1H), 7.15 (td, *J* = 9.5, 1.7 Hz, 2H), 7.09 (dd, *J* = 7.7, 4.6 Hz, 1H), 4.14 (q, *J* = 7.3 Hz, 2H), 3.91 – 3.80 (m, 2H), 3.76 (q, *J* = 7.9 Hz, 1H), 3.70 (s, 3H), 3.54 – 3.36 (m, 2H), 3.24 – 3.10 (m, 2H), 2.96 – 2.86 (m, 2H), 2.56 – 2.29 (m, 4H), 1.53 (d, *J* = 7.4 Hz, 3H), 1.25 (t, *J* = 6.6 Hz, 3H).

^{13}C NMR (126 MHz, $CDCl_3$, 25 °C, δ): 174.5, 159.8 (d, *J* = 247.5 Hz), 157.7, 155.7, 146.7, 142.0 (d, *J* = 7.7 Hz), 140.2, 139.8, 138.4, 138.2, 137.5, 137.1, 135.1, 134.5, 133.8, 130.8 (d, *J* = 4.4 Hz), 130.0, 129.4 (d, *J* = 3.3 Hz), 127.9, 127.5 (d, *J* = 12.7 Hz), 127.1, 124.8, 123.7 (d, *J* = 3.2 Hz), 122.3, 115.4 (d, *J* = 22.2 Hz), 61.4, 52.3, 45.1, 45.0, 44.9, 32.2, 31.8, 30.9, 30.7, 18.5, 14.8.

^{19}F NMR (471 MHz, $CDCl_3$, 25 °C, δ): –117.3.

HRMS ESIpos (m/z) calc'd for $C_{38}H_{38}N_2O_4F^+$ $[M+H]^+$, 605.2810; found, 605.2815. Deviation: –0.9 ppm.

Fenofibrate derivative 18

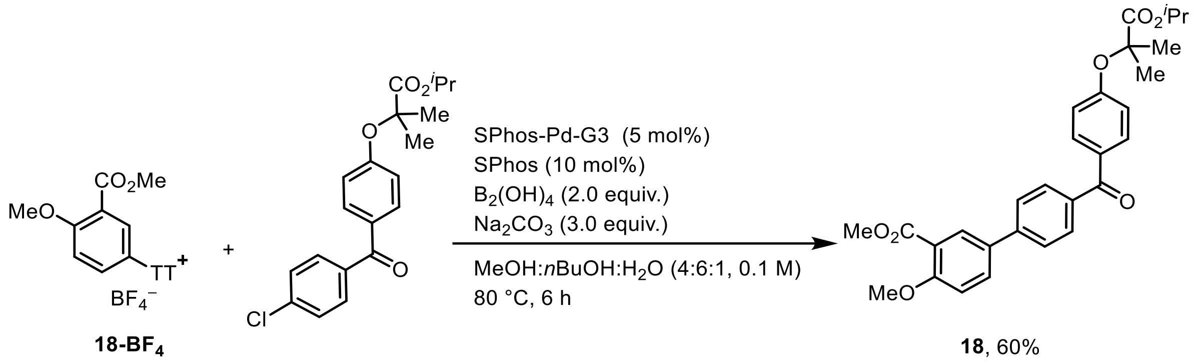

Under ambient atmosphere, a 4-mL borosilicate vial equipped with a magnetic stir bar and Schlenk-line adapter was charged with methyl-2methoxybenzoate-derived thianthrenium salt **18-BF4** (46.8 mg, 0.100 mmol, 1.00 equiv.), fenofibrate (43.3 mg, 0.120 mmol, 1.20 equiv.), $B_2(OH)_4$ (17.9 mg, 0.200 mmol, 2.00 equiv.), Na_2CO_3 (31.8 mg, 0.300 mmol, 3.00 equiv.), SPhos (4.1 mg, 10 µmol, 10 mol%) and SPhos-Pd-G3 (3.9 mg, 5.0 µmol, 5.0 mol%). A solvent mixture of MeOH:*n*BuOH:H_2O (4:6:1, 1 mL, *c* = 0.1 M) was added via syringe. The vial was sealed and then the reaction mixture was stirred vigorously at 80 °C for 6 h on a heating block. After the indicated time, the reaction mixture was allowed to cool to 23 °C. Then, the reaction vial was opened to air, and the resulting mixture was concentrated by rotary evaporation. The residue was purified by chromatography on silica gel eluting with hexanes/ethyl acetate (10:1 to 2:1, v/v) to afford 29.2 mg (60% yield) of the title compound as a brown solid.

R_f = 0.50 (hexanes/EtOAc, 5:1, v/v (UV))

NMR Spectroscopy:

^{1}H NMR (500 MHz, $CDCl_3$, 25 °C, δ): 8.09 (d, *J* = 2.4 Hz, 1H), 7.84 – 7.77 (m, 4H), 7.75 (dd, *J* = 8.7, 2.2 Hz, 1H), 7.68 – 7.64 (m, 2H), 7.08 (d, *J* = 8.7 Hz, 1H), 6.88 (d, *J* = 9.2 Hz, 2H), 5.08 (p, *J* = 6.7 Hz, 1H), 3.95 (s, 3H), 3.93 (s, 3H), 1.66 (s, 6H), 1.20 (d, *J* = 6.2 Hz, 6H).

^{13}C NMR (126 MHz, $CDCl_3$, 25 °C, δ): 194.9, 173.0, 166.3, 159.4, 159.0, 143.1, 136.5, 132.0, 131.9, 131.8, 130.6, 130.4, 130.3, 126.2, 120.4, 117.1, 112.5, 79.3, 69.2, 56.1, 52.1, 25.3, 21.4.

HRMS ESIpos (m/z) calc'd for $C_{29}H_{31}O_7^+$ $[M+H]^+$, 491.2064; found, 491.2067. Deviation: –0.7 ppm.

Fenbufen methyl ester derivative 19

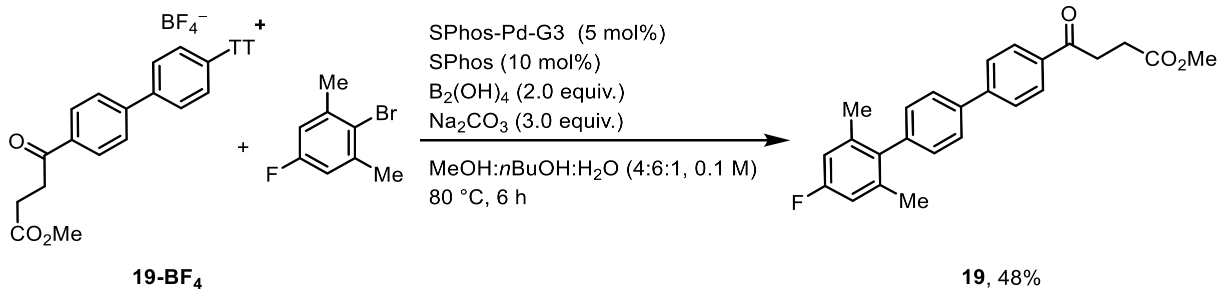

Under ambient atmosphere, a 4-mL borosilicate vial equipped with a magnetic stir bar and Schlenk-line adapter was charged with fenbufen methyl ester-derived thianthrenium salt **19-BF$_4$** (57.0 mg, 0.100 mmol, 1.00 equiv.), 2-bromo-5-fluoro-1,3-dimethylbenzene (26.4 mg, 0.130 mmol, 1.30 equiv.), $B_2(OH)_4$ (17.9 mg, 0.200 mmol, 2.00 equiv.), Na_2CO_3 (31.8 mg, 0.300 mmol, 3.00 equiv.), SPhos (4.1 mg, 10 µmol, 10 mol%) and SPhos-Pd-G3 (3.9 mg, 5.0 µmol, 5.0 mol%). A solvent mixture of MeOH:*n*BuOH:H_2O (4:6:1, 1 mL, *c* = 0.1 M) was added via syringe. The vial was sealed and then the reaction mixture was stirred vigorously at 80 °C for 6 h on a heating block. After the indicated time, the reaction mixture was allowed to cool to 23 °C. Then, the reaction vial was opened to air, and the resulting mixture was concentrated by rotary evaporation. The residue was purified by chromatography on silica gel eluting with hexanes/ethyl acetate (10:1, v/v) to afford 18.9 mg (48% yield) of the title compound as a yellow solid.

R*f* = 0.17 (hexanes/EtOAc, 10:1, v/v (UV))

NMR Spectroscopy:

^{1}H NMR (500 MHz, $CDCl_3$, 25 °C, δ): 8.09 (d, *J* = 8.6 Hz, 2H), 7.76 (d, *J* = 8.9 Hz, 2H), 7.70 (d, *J* = 8.1 Hz, 2H), 7.22 (d, *J* = 8.1 Hz, 2H), 6.84 (d, *J* = 9.4 Hz, 2H), 3.72 (s, 3H), 3.37 (t, *J* = 7.1 Hz, 2H), 2.81 (t, *J* = 6.1 Hz, 2H), 2.06 (s, 6H).

^{13}C NMR (126 MHz, $CDCl_3$, 25 °C, δ): 197.7, 173.6, 161.7 (d, *J* = 246.6 Hz), 145.7, 140.6, 138.5, 138.4 (d, *J* = 5.1 Hz), 137.1 (d, *J* = 2.9 Hz), 135.4, 130.2, 128.8, 127.5, 127.3, 113.9 (d, *J* = 21.0 Hz), 52.0, 33.6, 28.2, 21.2.

^{19}F NMR (471 MHz, $CDCl_3$, 25 °C, δ): –116.6.

HRMS ESIpos (m/z) calc'd for $C_{25}H_{23}O_3FNa^+$ $[M+Na]^+$, 413.1523; found, 413.1523. Deviation: –0.1 ppm.

1,2-Dimethoxybenzene derivative 20

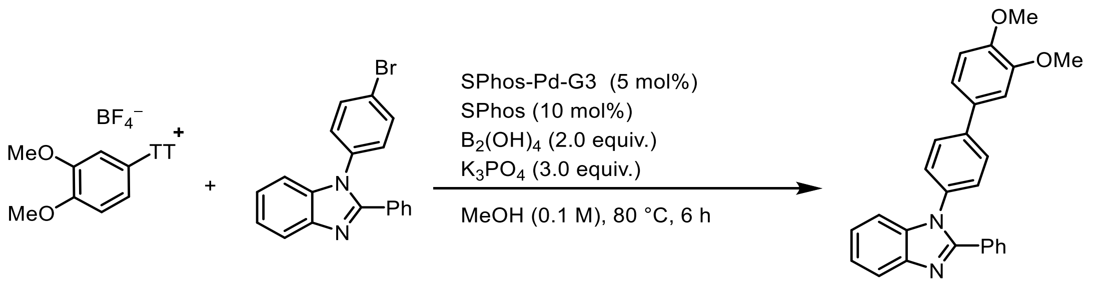

20-BF$_4$ **20**, 64%

Under ambient atmosphere, a 4-mL borosilicate vial equipped with a magnetic stir bar and Schlenk-line adapter was charged with 1,2-dimethoxybenzene-derived thianthrenium salt **20-BF$_4$** (44.0 mg, 0.100 mmol, 1.00 equiv.), 1-(4-bromophenyl)-2-phenylbenzimidazole (34.9 mg, 0.100 mmol, 1.00 equiv.), $B_2(OH)_4$ (17.9 mg, 0.200 mmol, 2.00 equiv.), K_3PO_4 (63.7 mg, 0.300 mmol, 3.00 equiv.), SPhos (4.1 mg, 10 µmol, 10 mol%) and SPhos-Pd-G3 (3.9 mg, 5.0 µmol, 5.0 mol%). The vial was evacuated and backfilled with argon three times using a Schlenk line. MeOH (1 mL, *c* = 0.1 M) was added via syringe. The vial was sealed and then the reaction mixture was stirred vigorously at 80 °C for 6 h on a heating block. After the indicated time,

the reaction mixture was allowed to cool to 23 °C. Then, the reaction vial was opened to air, and the resulting mixture was concentrated by rotary evaporation. The residue was purified by chromatography on silica gel eluting with hexanes/ethyl acetate (10:1 to 2:1, v/v) to afford 26.3 mg (64% yield) of the title compound as a colorless solid.

R*f* = 0.33 (hexanes/EtOAc, 2:1, v/v (UV))

NMR Spectroscopy:

^{1}H NMR (500 MHz, $CDCl_3$, 25 °C, δ): 7.90 (d, *J* = 8.1 Hz, 1H), 7.68 (d, *J* = 8.7 Hz, 2H), 7.63 (d, *J* = 7.1 Hz, 2H), 7.38 – 7.27 (m, 8H), 7.21 (dd, *J* = 8.4, 1.9 Hz, 1H), 7.16 (d, *J* = 2.1 Hz, 1H), 6.98 (d, *J* = 8.1 Hz, 1H), 3.98 (s, 3H), 3.95 (s, 3H).

^{13}C NMR (126 MHz, $CDCl_3$, 25 °C, δ): 152.4, 149.4, 149.2, 143.1, 141.2, 137.3, 135.6, 132.6, 130.0, 129.5, 129.4, 128.4, 128.0, 127.7, 123.4, 123.0, 119.9, 119.6, 111.6, 110.5, 110.3, 56.1.

HRMS ESIpos (m/z) calc'd for $C_{27}H_{23}N_2O_2^+$ [M+H]$^+$, 407.1754; found, 407.1754. Deviation: –0.1 ppm.

Pyriproxyfen derivative 21

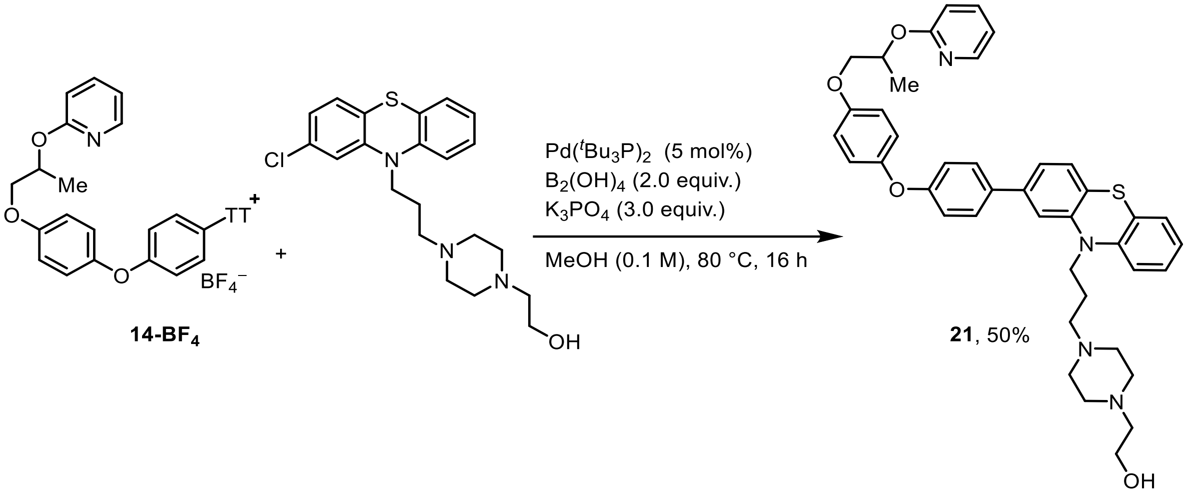

Under ambient atmosphere, a 4-mL borosilicate vial equipped with a magnetic stir bar and Schlenk-line adapter was charged with pyriproxyfen-derived thianthrenium salt **14-BF$_4$** (62.1 mg, 0.100 mmol, 1.00 equiv.), perphenazine (40.4 mg, 0.100 mmol, 1.00 equiv.), $B_2(OH)_4$ (17.9 mg, 0.200 mmol, 2.00 equiv.), K_3PO_4 (63.7 mg, 0.300 mmol, 3.00 equiv.), and Pd(^{t}Bu$_3$P)$_2$ (2.5 mg, 5.0 µmol, 5.0 mol%). MeOH (1 mL, *c* = 0.1 M) was added via syringe. The vial was sealed and then the reaction mixture was stirred vigorously at 80 °C for 16 h on a heating block. After the indicated time, the reaction mixture was allowed to cool to 23 °C. Then, the reaction vial was opened to air, and the resulting mixture was concentrated by rotary evaporation. The residue was purified by chromatography on silica gel eluting with DCM/MeOH (50:1 to 20:1, v/v) to afford 35.2 mg (50% yield) of the title compound as a brown oil.

R*f* = 0.30 (DCM/MeOH, 10:1, v/v (UV))

NMR Spectroscopy:

^{1}H NMR (500 MHz, $CDCl_3$, 25 °C, δ): 8.15 (dd, *J* = 5.1, 1.6 Hz, 1H), 7.57 (ddd, *J* = 8.7, 6.7, 2.2 Hz, 1H), 7.47 – 7.43 (m, 2H), 7.17 – 7.13 (m, 3H), 7.08 (dd, *J* = 7.8, 1.7 Hz, 1H), 7.02 – 6.89 (m, 9H), 6.86 (dd, *J* = 6.9, 5.3 Hz, 1H), 6.75 (d, *J* = 8.9 Hz, 1H), 5.59 (td, *J* = 11.9, 6.5 Hz, 1H), 4.20 (dd, *J* = 9.5, 5.3 Hz, 1H), 4.08 (dd, *J* = 9.9, 4.7 Hz, 1H), 3.99 (t, *J* = 6.6 Hz, 2H), 3.58 (t, *J* = 5.5 Hz, 2H), 2.58 – 2.38 (m, 12H), 1.98 (m, 2H), (tt, *J* = 9.9, 4.7 Hz, 1H), 1.48 (d, *J* = 6.6 Hz, 3H).

^{13}C NMR (126 MHz, $CDCl_3$, 25 °C, δ): 163.3, 158.3, 155.5, 150.2, 146.9, 145.8, 145.3, 140.2, 138.8, 135.4, 128.3, 127.7, 127.6, 127.4, 125.3, 124.1, 122.6, 121.2, 120.9, 117.9, 116.9, 115.9, 115.8, 114.4, 111.8, 71.2, 69.4, 59.3, 57.8, 55.8, 53.4, 52.9, 45.4, 24.6, 17.1.

HRMS ESIpos (m/z) calc'd for $C_{41}H_{45}N_4O_4S^+$ $[M+H]^+$, 689.3156; found, 689.3162. Deviation: –0.9 ppm.

2-Fluoro-6-phenoxybenzonitrile derivative 22

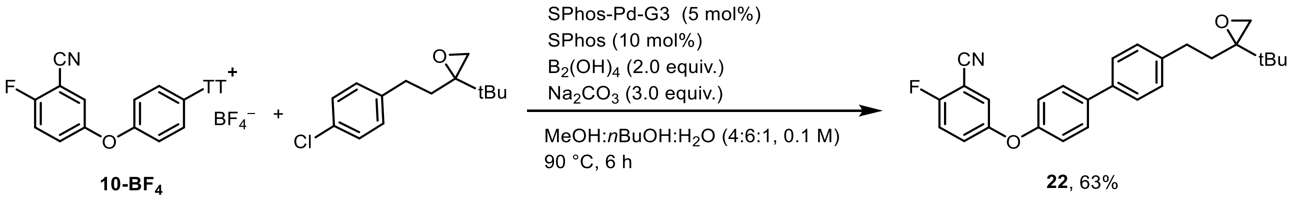

Under ambient atmosphere, a 4-mL borosilicate vial equipped with a magnetic stir bar and Schlenk-line adapter was charged with 2-fluoro-5-phenoxybenzonitrile-derived thianthrenium salt **10-BF₄** (51.6 mg, 0.100 mmol, 1.00 equiv.), 2-(*tert*-butyl)-2-(4-chlorophenyl)oxirane (31.0 mg, 0.130 mmol, 1.30 equiv.), $B_2(OH)_4$ (17.9 mg, 0.200 mmol, 2.00 equiv.), Na_2CO_3 (31.8 mg, 0.300 mmol, 3.00 equiv.), SPhos (4.1 mg, 10 µmol, 10 mol%) and SPhos-Pd-G3 (3.9 mg, 5.0 µmol, 5.0 mol%). The vial was evacuated and backfilled with argon three times using a Schlenk line. MeOH (1 mL, *c* = 0.1 M) was added via syringe. The vial was sealed and then the reaction mixture was stirred vigorously at 90 °C for 6 h on a heating block. After the indicated time, the reaction mixture was allowed to cool to 23 °C. Then, the reaction vial was opened to air, and the resulting mixture was concentrated by rotary evaporation. The residue was purified by chromatography on silica gel eluting with hexanes/ethyl acetate (50:1 to 5:1, v/v) to afford 26.1 mg (63% yield) of the title compound as a yellow oil.

R*f* = 0.30 (hexanes/EtOAc, 10:1, v/v (UV))

NMR Spectroscopy:

^{1}H NMR (500 MHz, $CDCl_3$, 25 °C, δ): 7.63 – 7.59 (m, 2H), 7.52 – 7.48 (m, 2H), 7.43 (td, *J* = 8.2, 6.6 Hz, 1H), 7.29 – 7.26 (m, 2H), 7.18 – 7.14 (m, 2H), 6.89 (t, *J* = 8.7 Hz, 1H), 6.68 (d, *J* = 8.1 Hz, 1H), 2.80 (d, *J* = 4.4 Hz, 1H), 2.70 (d, *J* = 4.4 Hz, 1H), 2.66 – 2.47 (m, 2H), 2.21 – 2.02 (m, 2H), 0.99 (s, 9H).

^{13}C NMR (126 MHz, $CDCl_3$, 25 °C, δ): 164.2 (d, *J* = 263.1 Hz), 161.2 (d, *J* = 4.3 Hz), 153.8, 141.8, 138.9, 137.8, 134.9 (d, *J* = 10.6 Hz), 129.0, 128.9, 127.2, 120.8, 112.0 (d, *J* = 3.4 Hz), 111.3, 109.8 (d,

J = 18.5 Hz), 93.8 (d, J = 17.6 Hz), 63.5, 48.1, 34.1, 31.5, 30.3, 26.2.

^{19}F NMR (471 MHz, $CDCl_3$, 25 °C, δ): –104.6.

HRMS ESIpos (m/z) calc'd for $C_{27}H_{26}NO_2FNa^+$ $[M+Na]^+$, 438.1840; found, 438.1836. Deviation: +0.8 ppm.

Flurbiprofen methyl ester derivative 23

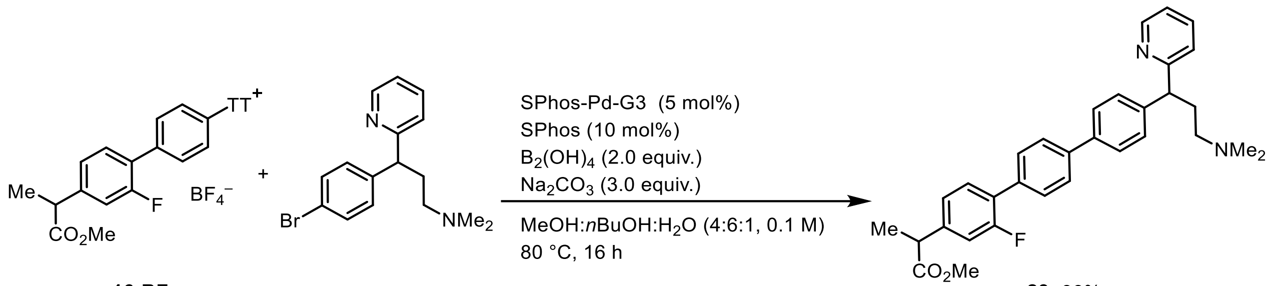

Under ambient atmosphere, a 4-mL borosilicate vial equipped with a magnetic stir bar and Schlenk-line adapter was charged with flurbiprofen methyl ester-derived thianthrenium salt **16-BF$_4$** (56.1 mg, 0.100 mmol, 1.00 equiv.), brompheniramine (31.9 mg, 0.100 mmol, 1.00 equiv.), $B_2(OH)_4$ (17.9 mg, 0.200 mmol, 2.00 equiv.), Na_2CO_3 (31.8 mg, 0.300 mmol, 3.00 equiv.), SPhos (4.1 mg, 10 µmol, 10 mol%) and SPhos-Pd-G3 (3.9 mg, 5.0 µmol, 5.0 mol%). The vial was evacuated and backfilled with argon three times using a Schlenk line. A solvent mixture of MeOH:*n*BuOH:H_2O (4:6:1, 1 mL, c = 0.1 M) was added via syringe. The vial was sealed and then the reaction mixture was stirred vigorously at 80 °C for 16 h on a heating block. After the indicated time, the reaction mixture was allowed to cool to 23 °C. Then, the reaction vial was opened to air, and the resulting mixture was concentrated by rotary evaporation. The residue was purified by chromatography on silica gel eluting with DCM/MeOH (20:1 to 10:1, v/v) to afford 33.4 mg (66% yield) of the title compound as a colorless oil.

R_f = 0.20 (DCM/MeOH, 10:1, v/v (UV))

NMR Spectroscopy:

^{1}H NMR (500 MHz, $CDCl_3$, 25 °C, δ): 8.59 (d, J = 5.4 Hz, 1H), 7.65 – 7.60 (m, 2H), 7.59 – 7.54 (m, 5H), 7.45 – 7.40 (m, 3H), 7.22 (d, J = 7.7 Hz, 1H), 7.18 – 7.10 (m, 3H), 4.22 – 4.18 (m, 1H), 3.76 (q, J = 7.8 Hz, 1H), 3.69 (s, 3H), 2.52 – 2.44 (m, 1H), 2.31 – 2.23 (m, 3H), 2.21 (s, 6H), 1.53 (d, J = 7.0 Hz, 3H).

^{13}C NMR (126 MHz, $CDCl_3$, 25 °C, δ): 174.6, 163.5, 159.8 (d, J = 236.2 Hz),149.5, 142.9, 141.9 (d, J = 7.3 Hz), 140.3, 138.9, 136.6, 134.4, 130.8 (d, J = 3.9 Hz), 129.4 (d, J = 3.2 Hz), 128.6, 127.5 (d, J = 13.6 Hz), 127.3, 127.2, 123.7 (d, J = 3.3 Hz), 123.1, 121.6, 115.4 (d, J = 23.0 Hz), 57.9, 52.4, 51.1, 45.4, 45.1, 32.8, 18.6.

^{19}F NMR (471 MHz, $CDCl_3$, 25 °C, δ): –117.3.

HRMS ESIpos (m/z) calc'd for $C_{32}H_{34}N_2O_2F^+$ $[M+H]^+$,497.2598; found, 497.2597. Deviation: +0.2 ppm.

4-(3-Fluoro-4-methoxyphenyl)-2,3-dihydro-1H-inden-1-one (24)

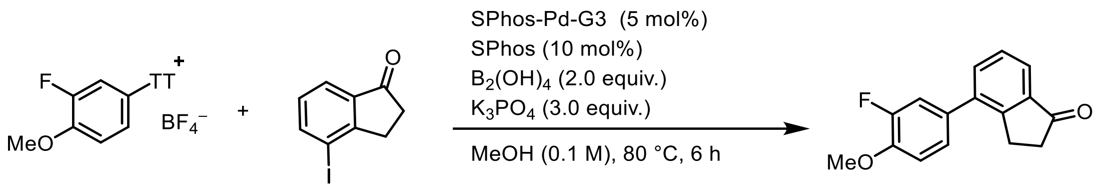

2-BF$_4$ **24**, 41%

Under ambient atmosphere, a 4-mL borosilicate vial equipped with a magnetic stir bar and Schlenk-line adapter was charged with 2-fluoroanisole-derived thianthrenium salt **2-BF$_4$** (42.8 mg, 0.100 mmol, 1.00 equiv.), 4-iodo-1-indanone (33.5 mg, 0.130 mmol, 1.30 equiv.), $B_2(OH)_4$ (17.9 mg, 0.200 mmol, 2.00 equiv.), K_3PO_4 (63.7 mg, 0.300 mmol, 3.00 equiv.), SPhos (4.1 mg, 10 µmol, 10 mol%) and SPhos-Pd-G3 (3.9 mg, 5.0 µmol, 5.0 mol%). MeOH (1 mL, *c* = 0.1 M) was added via syringe. The vial was sealed and then the reaction mixture was stirred vigorously at 80 °C for 6 h on a heating block. After the indicated time, the reaction mixture was allowed to cool to 23 °C. Then, the reaction vial was opened to air, and the resulting mixture was concentrated by rotary evaporation. The residue was purified by chromatography on silica gel eluting with hexanes/ethyl acetate (10:1 to 5:1, v/v) to afford 10.4 mg (41% yield) of the title compound as a brown solid.

R*f* = 0.60 (hexanes/EtOAc, 1:1, v/v (UV))

NMR Spectroscopy:

^{1}H NMR (500 MHz, $CDCl_3$, 25 °C, δ): 7.76 (d, *J* = 7.7 Hz, 1H), 7.55 (dd, *J* = 7.6, 1.1 Hz, 1H), 7.47 – 7.43 (m, 1H), 7.21 (dd, *J* = 11.7, 2.0 Hz, 1H), 7.19 – 7.16 (m, 1H), 7.06 (t, *J* = 8.9 Hz, 1H), 3.95 (s, 3H), 3.16 (t, *J* = 5.7 Hz, 2H), 2.72 – 2.68 (m, 2H).

^{13}C NMR (126 MHz, $CDCl_3$, 25 °C, δ): 207.1, 152.6, 152.3 (d, *J* = 245.7 Hz), 147.4 (d, *J* = 10.8 Hz), 139.2, 137.8, 134.7, 132.2 (d, *J* = 6.5 Hz), 128.2, 124.5 (d, *J* = 3.4 Hz), 122.9, 116.4 (d, *J* = 17.9 Hz), 113.6 (d, *J* = 2.3 Hz), 56.5, 36.5, 25.9.

^{19}F NMR (471 MHz, $CDCl_3$, 25 °C, δ): –134.7.

HRMS GC-EI (m/z) calc'd for $C_{16}H_{13}O_2F^+$ $[M]^+$, 256.0894; found, 256.0896. Deviation: –1.0 ppm.

Estrone derivative 25

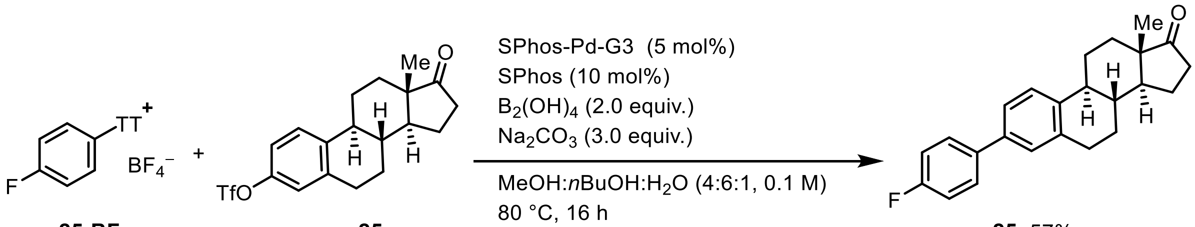

25-BF$_4$ **25a** **25**, 57%

Under ambient atmosphere, a 4-mL borosilicate vial equipped with a magnetic stir bar and Schlenk-line adapter was charged with fluorobenzene-derived thianthrenium salt **25-BF$_4$** (39.8 mg, 0.100 mmol, 1.00

equiv.), estrone triflate **25a** (40.2 mg, 0.100 mmol, 1.00 equiv.), $B_2(OH)_4$ (17.9 mg, 0.200 mmol, 2.00 equiv.), Na_2CO_3 (31.8 mg, 0.300 mmol, 3.00 equiv.), SPhos (4.1 mg, 10 µmol, 10 mol%) and SPhos-Pd-G3 (3.9 mg, 5.0 µmol, 5.0 mol%). The vial was evacuated and backfilled with argon three times using a Schlenk line. A solvent mixture of MeOH:*n*BuOH:H_2O (4:6:1, 1 mL, *c* = 0.1 M) .) was added via syringe. The vial was sealed and then the reaction mixture was stirred vigorously at 80 °C for 16 h on a heating block. After the indicated time, the reaction mixture was allowed to cool to 23 °C. Then, the reaction vial was opened to air, and the resulting mixture was concentrated by rotary evaporation. The residue was purified by chromatography on silica gel eluting with hexanes/ethyl acetate (20:1 to 10:1, v/v) to afford 20.1 mg (57% yield) of the title compound as a brown solid.

R*f* = 0.24 (hexanes/EtOAc, 10:1, v/v (UV))

NMR Spectroscopy:

^{1}H NMR (500 MHz, $CDCl_3$, 25 °C, δ): 7.57 – 7.49 (m, 2H), 7.40 – 7.31 (m, 2H), 7.31 – 7.28 (m, 1H), 7.16 – 7.06 (m, 2H), 2.99 (dd, *J* = 9.3, 4.5 Hz, 2H), 2.58 – 2.43 (m, 2H), 2.41 – 2.30 (m, 1H), 2.23 – 1.95 (m, 4H), 1.72 – 1.44 (m, 6H), 0.93 (s, 3H).

^{13}C NMR (126 MHz, $CDCl_3$, 25 °C, δ): 220.9, 162.4 (d, *J* = 243.3 Hz), 139.1, 137.9, 137.2 (d, *J* = 3.3 Hz), 137.1, 128.6 (d, *J* = 7.9 Hz), 127.7, 126.0, 124.6, 115.7 (d, *J* = 21.7 Hz), 50.7, 48.1, 44.5, 38.3, 35.9, 31.8, 29.7, 26.7, 25.9, 21.7, 14.0.

^{19}F NMR (471 MHz, $CDCl_3$, 25 °C, δ): –116.1.

HRMS GC-EI (m/z) calc'd for $C_{24}H_{25}OF^+$ $[M]^+$, 348.1883; found, 348.1889. Deviation: –1.6 ppm.

3,3-Diphenylpropan-1-ol derivative 26

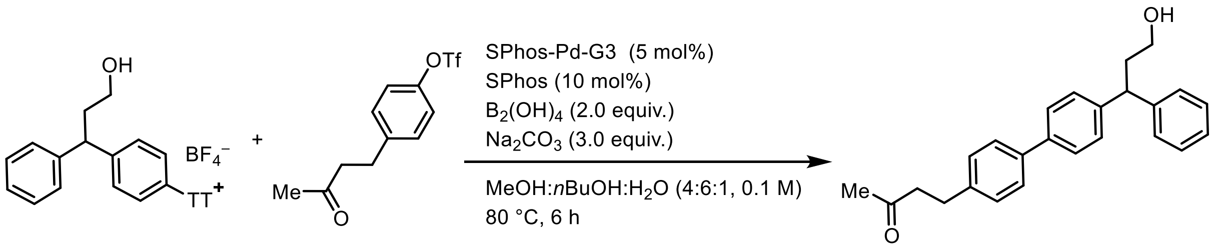

26-BF$_4$ **26a** **26,** 75%

Under ambient atmosphere, a 4-mL borosilicate vial equipped with a magnetic stir bar and Schlenk-line adapter was charged with 3,3-diphenylpropan-1-ol-derived thianthrenium salt **26-BF$_4$** (51.4 mg, 0.100 mmol, 1.00 equiv.), 4-(3-oxobutyl)phenyl triflate **26a** (29.6 mg, 0.100 mmol, 1.00 equiv.), $B_2(OH)_4$ (17.9 mg, 0.200 mmol, 2.00 equiv.), Na_2CO_3 (31.8 mg, 0.300 mmol, 3.00 equiv.), SPhos (4.1 mg, 10 µmol, 10 mol%) and SPhos-Pd-G3 (3.9 mg, 5.0 µmol, 5.0 mol%). The vial was evacuated and backfilled with argon three times using a Schlenk line. A solvent mixture of MeOH:*n*BuOH:H_2O (4:6:1, 1 mL, *c* = 0.1 M) was added via syringe. The vial was sealed and then the reaction mixture was stirred vigorously at 80 °C for 6 h on a heating block. After the indicated time, the reaction mixture was allowed to cool to 23 °C. Then, the reaction vial was opened to air, and the resulting mixture was concentrated by rotary evaporation. The residue was

purified by chromatography on silica gel eluting with hexanes/ethyl acetate (10:1 to 1:1, v/v) to afford 27.3 mg (75% yield) of the title compound as a brown oil.

R*f* = 0.30 (hexanes/EtOAc, 1:1, v/v (UV))

NMR Spectroscopy:

^{1}**H NMR** (500 MHz, $CDCl_3$, 25 °C, δ): 7.48 – 7.42 (m, 4H), 7.30 – 7.25 (m, 6H), 7.22 – 7.16 (m, 3H), 4.15 (t, *J* = 7.6 Hz, 1H), 3.60 (t, *J* = 6.8 Hz, 2H), 2.89 (t, *J* = 7.7 Hz, 2H), 2.75 (t, *J* = 7.8 Hz, 2H), 2.35 – 2.28 (m, 2H), 2.11 (s, 3H).

^{13}**C NMR** (126 MHz, $CDCl_3$, 25 °C, δ): 208.1, 144.5, 143.6, 140.0, 138.9, 138.8, 128.8, 128.7, 128.3, 127.9, 127.2, 127.1, 126.5, 61.1, 47.1, 45.2, 38.3, 30.2, 29.4.

HRMS GC-EI (m/z) calc'd for $C_{25}H_{26}O_2^+$ $[M]^+$, 358.1927; found, 358.1925. Deviation: +0.5 ppm.

Flurbiprofen methyl ester derivative 27

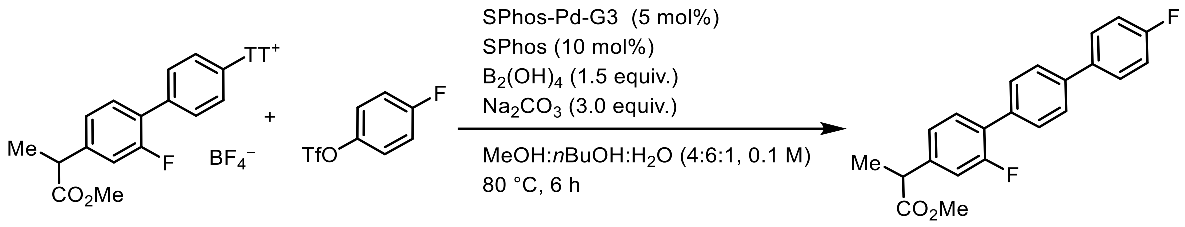

16-BF$_4$ **27**, 42%

Under ambient atmosphere, a 4-mL borosilicate vial equipped with a magnetic stir bar and Schlenk-line adapter was charged with flurbiprofen methyl ester-derived thianthrenium salt **16-BF$_4$** (56.1 mg, 0.100 mmol, 1.00 equiv.), 4-fluorophenyl triflate (31.7 mg, 0.130 mmol, 1.30 equiv.), $B_2(OH)_4$ (13.4 mg, 0.150 mmol, 1.50 equiv.), Na_2CO_3 (31.8 mg, 0.300 mmol, 3.00 equiv.), SPhos (4.1 mg, 10 μmol, 10 mol%) and SPhos-Pd-G3 (3.9 mg, 5.0 μmol, 5.0 mol%). The vial was evacuated and backfilled with argon three times using a Schlenk line. A solvent mixture of MeOH:*n*BuOH:H_2O (4:6:1, 1 mL, *c* = 0.1 M) was added via syringe. The vial was sealed and then the reaction mixture was stirred vigorously at 80 °C for 6 h on a heating block. After the indicated time, the reaction mixture was allowed to cool to 23 °C. Then, the reaction vial was opened to air, and the resulting mixture was concentrated by rotary evaporation. The residue was purified by chromatography on silica gel eluting with hexanes/ethyl acetate (10:1, v/v) to afford 14.7 mg (42% yield) of the title compound as a colorless solid.

R*f* = 0.25 (hexanes/EtOAc, 10:1, v/v (UV))

NMR Spectroscopy:

^{1}**H NMR** (500 MHz, $CDCl_3$, 25 °C, δ): 7.64 – 7.57 (m, 6H), 7.44 (t, *J* = 7.6 Hz, 1H), 7.19 – 7.13 (m, 4H), 3.78 (q, *J* = 7.6 Hz, 1H), 3.72 (s, 3H), 1.55 (d, *J* = 6.6 Hz, 3H).

^{13}**C NMR** (126 MHz, $CDCl_3$, 25 °C, δ): 174.6, 162.6 (d, *J* = 245.5 Hz), 159.8 (d, *J* = 248.8 Hz), 142.1 (d, *J* = 8.1 Hz), 139.7, 136.9 (d, *J* = 3.0 Hz), 134.6, 130.8 (d, *J* = 3.9 Hz), 129.5 (d, *J* = 3.0 Hz), 128.8 (d,

J = 8.0 Hz), 127.4 (d, *J* = 13.2 Hz), 127.2, 123.8 (d, *J* = 3.0 Hz), 115.8 (d, *J* = 21.1 Hz), 115.5 (d, *J* = 23.8 Hz), 52.4, 45.1, 18.6.

^{19}F NMR (471 MHz, $CDCl_3$, 25 °C, δ): –115.5, -117.4.

HRMS ESIpos (m/z) calc'd for $C_{22}H_{18}O_2F_2Na^+$ $[M+Na]^+$, 375.1167; found, 375.1165. Deviation: +0.6 ppm.

Methyl-4´-ethyl-(1,1´-biphenyl)-4-carboxylate (28)

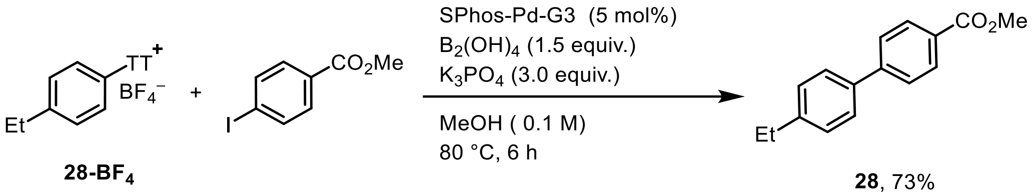

Under ambient atmosphere, a 4-mL borosilicate vial equipped with a magnetic stir bar and Schlenk-line adapter was charged with ethylbenzene-derived thianthrenium salt **28-BF₄** (40.8 mg, 0.100 mmol, 1.00 equiv.), methyl 4-iodobenzoate (34.1 mg, 0.130 mmol, 1.30 equiv.), $B_2(OH)_4$ (13.4 mg, 0.150 mmol, 1.50 equiv.), K_3PO_4 (63.7 mg, 0.300 mmol, 3.00 equiv.) and SPhos-Pd-G3 (3.9 mg, 5.0 μmol, 5.0 mol%). The vial was evacuated and backfilled with argon three times using a Schlenk line. MeOH (1 mL, *c* = 0.1 M) was added via syringe. The vial was sealed and then the reaction mixture was stirred vigorously at 80 °C for 6 h on a heating block. After the indicated time, the reaction mixture was allowed to cool to 23 °C. Then, the reaction vial was opened to air, and the resulting mixture was concentrated by rotary evaporation. The residue was purified by chromatography on silica gel eluting with hexanes/ethyl acetate (50:1, v/v) to afford 17.5 mg (73% yield) of the title compound as a colorless solid.

R_f = 0.21 (hexanes/EtOAc, 20:1, v/v (UV))

NMR Spectroscopy:

^{1}H NMR (500 MHz, $CDCl_3$, 25 °C, δ): 8.09 (d, *J* = 7.3 Hz, 2H), 7.65 (d, *J* = 7.3 Hz, 2H), 7.56 (d, *J* = 7.7 Hz, 2H), 7.30 (d, *J* = 7.9 Hz, 2H), 3.94 (s, 3H), 2.71 (q, *J* = 7.7 Hz, 2H), 1.28 (t, *J* = 8.1 Hz, 3H).

^{13}C NMR (126 MHz, $CDCl_3$, 25 °C, δ): 167.2, 145.8, 144.6, 137.5, 130.2, 128.7, 128.6, 127.3, 126.9, 52.2, 28.7, 15.7.

HRMS GC-EI (m/z) calc'd for $C_{16}H_{16}O_2^+$ $[M]^+$, 240.1144; found, 240.1146. Deviation: –0.6 ppm.

Palladium catalyzed borylation of aryl thianthrenium salts

2-(3-Fluoro-4-methoxyphenyl)-4,4,5,5-tetramethyl-1,3,2-dioxaborolane (3)

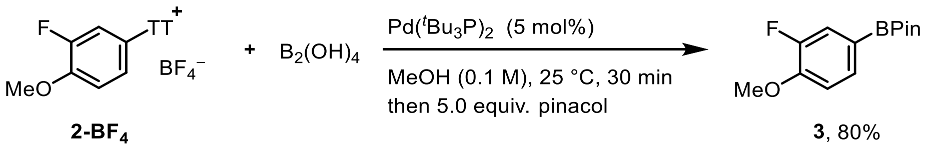

Under ambient atmosphere, a 4-mL borosilicate vial equipped with a magnetic stir bar was charged with 2-

fluoroanisole-derived thianthrenium salt **2-BF$_4$** (42.8 mg, 0.100 mmol, 1.00 equiv.), $B_2(OH)_4$ (13.4 mg, 0.150 mmol, 1.50 equiv.) and Pd(^{t}Bu$_3$P)$_2$ (2.5 mg, 5.0 μmol, 5.0 mol%). MeOH (1 mL, *c* = 0.1 M) was added via syringe into the reaction mixture. The vial was sealed and then the reaction mixture was stirred vigorously at 25 °C for 30 min. Subsequently, pinacol (59.1 mg, 0.500 mmol, 5.00 equiv.) was added, and the reaction mixture was stirred at 25 °C for 10 min. After that, the reaction vessel was opened to air, and the resulting mixture was concentrated by rotary evaporation. The residue was purified by chromatography on silica gel eluting with hexanes/ethyl acetate (50:1, v/v) to afford 20.2 mg (80% yield) of the title compound as a colorless oil.

R*f* = 0.05 (hexanes/EtOAc, 50:1, v/v (UV))

NMR Spectroscopy:

^{1}H NMR (500 MHz, $CDCl_3$, 25 °C, δ): 7.52 (d, *J* = 8.4 Hz, 1H), 7.48 (dd, *J* = 12.4 Hz, 1H), 6.84 (t, *J* = 8.2 Hz, 1H), 3.90 (s, 3H), 1.33 (s, 12H).

^{13}C NMR (126 MHz, $CDCl_3$, 25 °C, δ): 152.1 (d, *J* = 246.9 Hz), 150.4 (d, *J* = 10.6 Hz), 131.6 (d, *J* = 3.7 Hz), 121.8 (d, *J* = 16.4 Hz), 112.7, 84.0, 56.2, 25.0. The signal for the carbon directly attached to the boron atom was not detected due to the quadrupolar broadening.

^{19}F NMR (471 MHz, $CDCl_3$, 25 °C, δ): –137.1.

^{11}B NMR (96 MHz, $CDCl_3$, 25 °C, δ): 30.6.

HRMS GC-EI (m/z) calc'd for $C_{13}H_{18}O_3BF^+$ $[M]^+$, 252.1328; found, 252.1330. Deviation: –1.2 ppm.

4-(4,4,5,5-Tetramethyl-1,3,2-dioxaborolan-2-yl)anisole (3a)

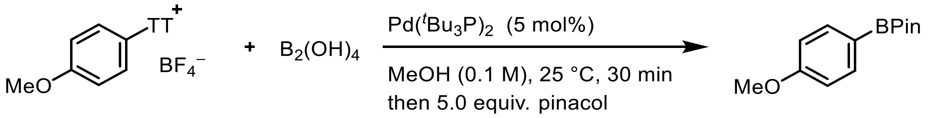

3a-BF$_4$ **3a**, 80%

Under ambient atmosphere, a 4-mL borosilicate vial equipped with a magnetic stir bar was charged with anisole-derived thianthrenium salt **3a-BF$_4$** (41.0 mg, 0.100 mmol, 1.00 equiv.), $B_2(OH)_4$ (13.4 mg, 0.150 mmol, 1.50 equiv.) and Pd(^{t}Bu$_3$P)$_2$ (2.5 mg, 5.0 μmol, 5.0 mol%). MeOH (1 mL, *c* = 0.1 M) was added via syringe into the reaction mixture. The vial was sealed and then the reaction mixture was stirred vigorously at 25 °C for 30 min. Subsequently, pinacol (59.1 mg, 0.500 mmol, 5.00 equiv.) was added, and the reaction mixture was stirred at 25 °C for 10 min. After that, the reaction vessel was opened to air, and the resulting mixture was concentrated by rotary evaporation. The residue was purified by chromatography on silica gel eluting with hexanes/ethyl acetate (50:1, v/v) to afford 18.7 mg (80% yield) of the title compound as a colorless oil.

R$_f$ = 0.20 (hexanes/EtOAc, 20:1, v/v (UV))

NMR Spectroscopy:

^{1}**H NMR** (500 MHz, $CDCl_3$, 25 °C, δ): 7.75 (d, *J* = 8.8 Hz, 2H), 6.90 (d, *J* = 8.8 Hz, 2H), 3.83 (s, 3H), 1.34 (s, 12H).

^{13}**C NMR** (126 MHz, $CDCl_3$, 25 °C, δ): 162.3, 136.7, 113.4, 83.7, 55.2, 25.0. The signal for the carbon directly attached to the boron atom was not detected due to the quadrupolar broadening.

^{11}**B NMR** (96 MHz, $CDCl_3$, 25 °C, δ): 30.9.

HRMS GC-EI (m/z) calc'd for $C_{13}H_{19}O_3B^+$ $[M]^+$, 234.1422; found, 234.1423. Deviation: –0.8 ppm

2-(4-Fluorophenyl)-4,4,5,5-tetramethyl-1,3,2-dioxaborolane (3b)

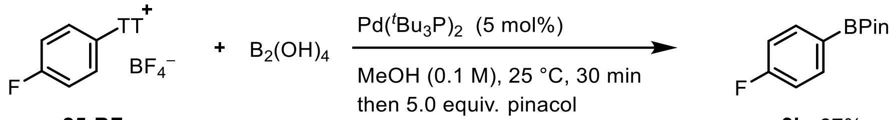

25-BF$_4$ **3b**, 67%

Under ambient atmosphere, a 4-mL borosilicate vial equipped with a magnetic stir bar was charged with fluorobenzene-derived thianthrenium salt **25-BF$_4$** (39.8 mg, 0.100 mmol, 1.00 equiv.), $B_2(OH)_4$ (13.4 mg, 0.150 mmol, 1.50 equiv.) and Pd(^{t}Bu$_3$P)$_2$ (2.5 mg, 5.0 µmol, 5.0 mol%). MeOH (1 mL, *c* = 0.1 M) was added via syringe into the reaction mixture. The vial was sealed and then the reaction mixture was stirred vigorously at 25 °C for 30 min. Subsequently, pinacol (59.1 mg, 0.500 mmol, 5.00 equiv.) was added, and the reaction mixture was stirred at 25 °C for 10 min. After that, the reaction vessel was opened to air, and the resulting mixture was concentrated by rotary evaporation. The residue was purified by chromatography on silica gel eluting with hexanes/ethyl acetate (100:1, v/v) to afford 14.9 mg (67% yield) of the title compound as a colorless oil.

R$_f$ = 0.43 (hexanes/EtOAc, 20:1, v/v (UV))

NMR Spectroscopy:

^{1}**H NMR** (500 MHz, $CDCl_3$, 25 °C, δ): 7.82 – 7.78 (m, 2H), 7.05 (t, *J* = 9.3 Hz, 2H), 1.34 (s, 12H).

^{13}**C NMR** (126 MHz, $CDCl_3$, 25 °C, δ): 165.2 (d, *J* = 246.6 Hz), 137.1 (d, *J* = 8.4 Hz), 115.0 (d, *J* = 20.8 Hz), 84.1, 25.0. The signal for the carbon directly attached to the boron atom was not detected due to the quadrupolar broadening.

^{19}**F NMR** (471 MHz, $CDCl_3$, 25 °C, δ): –108.5.

^{11}**B NMR** (96 MHz, $CDCl_3$, 25 °C, δ): 30.8.

HRMS GC-EI (m/z) calc'd for $C_{12}H_{16}O_2FB^+$ $[M]^+$, 222.1222; found, 222.1221. Deviation: +0.2 ppm.

2-(Phenyl)-4,4,5,5-tetramethyl-1,3,2-dioxaborolane (3c)

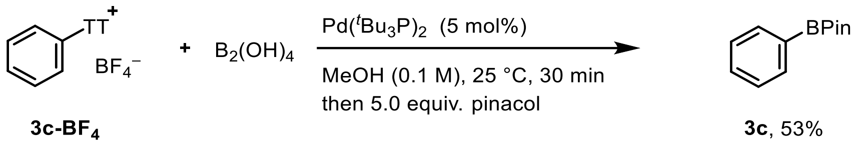

Under ambient atmosphere, a 4-mL borosilicate vial equipped with a magnetic stir bar was charged with benzene-derived thianthrenium salt **3c-BF$_4$** (38.0 mg, 0.100 mmol, 1.00 equiv.), $B_2(OH)_4$ (13.4 mg, 0.150 mmol, 1.50 equiv.) and Pd(^{t}Bu$_3$P)$_2$ (2.5 mg, 5.0 µmol, 5.0 mol%). MeOH (1 mL, *c* = 0.1 M) was added via syringe into the reaction mixture. The vial was sealed and then the reaction mixture was stirred vigorously at 25 °C for 30 min. Subsequently, pinacol (59.1 mg, 0.500 mmol, 5.00 equiv.) was added, and the reaction mixture was stirred at 25 °C for 10 min. After that, the reaction vessel was opened to air, and the resulting mixture was concentrated by rotary evaporation. The residue was purified by chromatography on silica gel eluting with hexanes/ethyl acetate (100:1, v/v) to afford 10.9 mg (53% yield) of the title compound as a colorless oil.

R*f* = 0.43 (hexanes/EtOAc, 20:1, v/v (UV))

NMR Spectroscopy:

^{1}H NMR (500 MHz, $CDCl_3$, 25 °C, δ): 7.82 (d, *J* = 7.5 Hz, 2H), 7.48 – 7.44 (m, 1H), 7.37 (t, *J* = 7.5 Hz, 2H), 1.35 (s, 12H).

^{13}C NMR (126 MHz, $CDCl_3$, 25 °C, δ): 134.9, 131.4, 127.9, 83.9, 25.0. The signal for the carbon directly attached to the boron atom was not detected due to the quadrupolar broadening.

^{11}B NMR (96 MHz, $CDCl_3$, 25 °C, δ): 31.1.

HRMS GC-EI (m/z) calc'd for $C_{12}H_{17}O_2B^+$ $[M]^+$, 204.1316; found, 204.1316. Deviation: –0.1 ppm.

Methyl 4-(4,4,5,5-tetramethyl-1,3,2-dioxaborolan-2-yl)benzoate (3d)

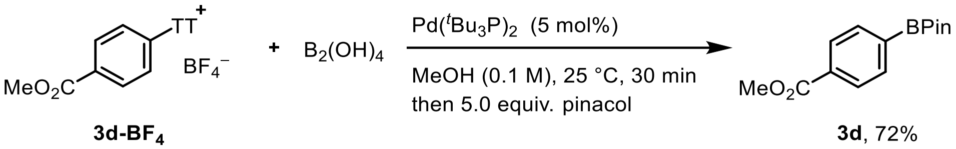

Under ambient atmosphere, a 4-mL borosilicate vial equipped with a magnetic stir bar was charged with methyl benzoate-derived thianthrenium salt **3d-BF$_4$** (43.8 mg, 0.100 mmol, 1.00 equiv.), $B_2(OH)_4$ (13.4 mg, 0.150 mmol, 1.50 equiv.) and Pd(^{t}Bu$_3$P)$_2$ (2.5 mg, 5.0 µmol, 5.0 mol%). MeOH (1 mL, *c* = 0.1 M) was added via syringe into the reaction mixture. The vial was sealed and then the reaction mixture was stirred vigorously at 25 °C for 30 min. Subsequently, pinacol (59.1 mg, 0.500 mmol, 5.00 equiv.) was added, and the reaction mixture was stirred at 25 °C for 10 min. After that, the reaction vessel was opened to air, and the resulting mixture was concentrated by rotary evaporation. The residue was purified by chromatography on silica gel eluting with hexanes/ethyl acetate (20:1, v/v) to afford 18.8 mg (72% yield) of the title compound as a colorless solid.

R*f* = 0.35 (hexanes/EtOAc, 20:1, v/v (UV))

NMR Spectroscopy:

^{1}H NMR (500 MHz, $CDCl_3$, 25 °C, δ): 8.01 (d, *J* = 8.4 Hz, 2H), 7.87 (d, *J* = 8.0 Hz, 2H), 3.92 (s, 3H).1.35 (s, 12H).

^{13}C NMR (126 MHz, $CDCl_3$, 25 °C, δ): 167.3, 134.8, 132.4, 128.7, 84.3, 52.3, 25.0. The signal for the carbon directly attached to the boron atom was not detected due to the quadrupolar broadening.

^{11}B NMR (96 MHz, $CDCl_3$, 25 °C, δ): 30.9.

HRMS GC-EI (m/z) calc'd for $C_{14}H_{19}O_4B^+$ $[M]^+$, 262.1371; found, 262.1370. Deviation: +0.3 ppm.

4,4,5,5-tetramethyl-2-(4-nitrophenyl)-1,3,2-dioxaborolane (3e)

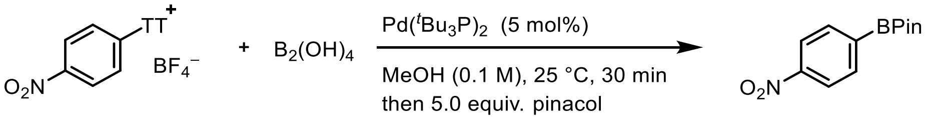

3e-BF$_4$ **3e**, 68%

Under ambient atmosphere, a 4-mL borosilicate vial equipped with a magnetic stir bar was charged with nitrobenzene-derived thianthrenium salt 3e-BF_4 (42.5 mg, 0.100 mmol, 1.00 equiv.), $B_2(OH)_4$ (13.4 mg, 0.150 mmol, 1.50 equiv.) and Pd(^{t}Bu$_3$P)$_2$ (2.5 mg, 5.0 μmol, 5.0 mol%). MeOH (1 mL, *c* = 0.1 M) was added via syringe into the reaction mixture. The vial was sealed and then the reaction mixture was stirred vigorously at 25 °C for 30 min. Subsequently, pinacol (59.1 mg, 0.500 mmol, 5.00 equiv.) was added, and the reaction mixture was stirred at 25 °C for 10 min. After that, the reaction vessel was opened to air, and the resulting mixture was concentrated by rotary evaporation. The residue was purified by chromatography on silica gel eluting with hexanes/ethyl acetate (20:1, v/v) to afford 17.0 mg (68% yield) of the title compound as a colorless oil.

R*f* = 0.24 (hexanes/EtOAc, 20:1, v/v (UV))

NMR Spectroscopy:

^{1}H NMR (500 MHz, $CDCl_3$, 25 °C, δ): 8.19 (d, *J* = 8.2 Hz, 2H), 7.96 (d, *J* = 8.3 Hz, 2H), 1.36 (s, 12H).

^{13}C NMR (126 MHz, $CDCl_3$, 25 °C, δ): 150.0, 135.8, 122.6, 84.8, 25.0. The signal for the carbon directly attached to the boron atom was not detected due to the quadrupolar broadening.

^{11}B NMR (96 MHz, $CDCl_3$, 25 °C, δ): 30.5.

HRMS APPIpos (m/z) calc'd for $C_{14}H_{19}O_4B^+$ $[M+H]^+$, 250.1245; found, 250.1243. Deviation: –0.7ppm.

Synthesis of arylthianthrenium salt

Nefiracetam-derived thianthrenium salt 1-BF_4

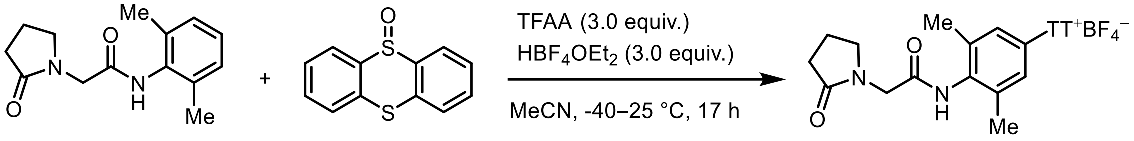

1-BF_4, 70%

The compound **1-BF_4** was prepared according to the modified versions of the reported procedures.[8] Under an ambient atmosphere, a 25 mL round-bottom flask equipped with a stir bar was charged with thianthrene-S-oxide (232 mg, 1.00 mmol, 1.0 equiv.), MeCN (10 mL, *c* = 0.1 M), and nefiracetam (246 mg, 1.0 mmol, 1.0 equiv.). After cooling to –40 °C, TFAA (417 µL, 630 mg, 3.00 mmol, 3.00 equiv.) and then $HBF_4 \cdot OEt_2$ (408 µL, 486 mg, 3.00 mmol, 3.00 equiv.) were subsequently added to the vial while stirring the reaction mixture. The reaction mixture was stirred at –40 °C for 1 hour, and then stirred at 25 °C for 16 hours. The solvent was removed under reduced pressure by rotary evaporation and the residue was dissolved in DCM (10 mL). The resulting solution was washed with saturated aqueous $NaHCO_3$ solution (10 mL) and aqueous $NaBF_4$ solution (2 × 10 mL, 10% w/w). The organic phase was dried over Na_2SO_4, filtered, and the solvent was removed under reduced pressure. The residue was purified by chromatography on silica gel eluting with DCM/MeOH (50:1 to 20:1, v/v) to afford **1-BF_4** (386 mg, 70%) as a colorless solid.

R_f = 0.20 (DCM/MeOH, 20:1, v/v (UV))

NMR Spectroscopy:

^{1}H NMR (500 MHz, DMSO-d_6, 25 °C, δ): 9.56 (s, 1H), 8.55 (dd, *J* = 7.5, 1.1 Hz, 2H), 8.07 (dd, *J* =8.3, 0.9 Hz, 2H), 7.93 (td, *J* = 7.7, 1.4 Hz, 2H), 7.84 (td, *J* = 7.9, 1.3 Hz, 2H), 6.98 (s, 2H), 4.03 (s, 2H), 3.41 (t, *J* = 7.4 Hz, 2H), 2.23 (t, *J* = 8.3 Hz, 2H), 2.08 (s, 6H), 1.95 (p, *J* = 7.7 Hz, 2H).

^{13}C {^{1}H} NMR (126 MHz, DMSO-d_6, 25 °C, δ): 174.7, 166.5, 139.3, 138.2, 135.4, 135.3, 134.8, 130.2, 129.7, 126.9, 121.9, 119.1, 47.4, 45.1, 29.9, 18.3, 17.4.

^{19}F NMR (471 MHz, DMSO-d_6, 25 °C, δ): –148.2 (bs), –148.3 (bs).

HRMS ESI (m/z) calc'd for $C_{26}H_{25}O_2N_2S_2^+$ [M-BF4]$^+$, 461.1352; found, 461.1353. Deviation: –0.11 ppm.

2-Fluoroanisole-derived thianthrenium salt 2-BF_4

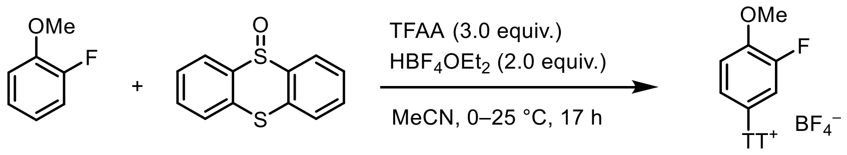

2-BF_4, 85%

The compound **2-BF$_4$** was prepared according to the modified versions of the reported procedures and the NMR spectra are in good accordance with reported data.[9] Under an ambient atmosphere, a 25 mL round-bottom flask equipped with a stir bar was charged with thianthrene-S-oxide (232 mg, 1.00 mmol, 1.00 equiv.), MeCN (10 mL, *c* = 0.1 M), and 2-fluoroanisole (112 μL, 1.00 mmol, 1.00 equiv.). After cooling to 0 °C, TFAA (417 μL, 630 mg, 3.00 mmol, 3.00 equiv.) and then HBF_4·OEt_2 (272 μL, 324 mg, 2.00 mmol, 2.00 equiv.) were subsequently added to the vial while stirring the reaction mixture. The reaction mixture was stirred at 0 °C for 1 hour, and then stirred at 25 °C for 16 hours. The solvent was removed under reduced pressure by rotary evaporation and the residue was dissolved in DCM (10 mL). The resulting solution was washed with saturated aqueous $NaHCO_3$ solution (10 mL) and aqueous $NaBF_4$ solution (2 × 10 mL, 10% w/w). The organic phase was dried over Na_2SO_4, filtered, and the solvent was removed under reduced pressure. The residue was purified by chromatography on silica gel eluting with DCM/MeOH (50:1 to 20:1, v/v) to afford **2-BF$_4$** (362 mg, 85%) as a colorless solid.

R*f* = 0.30 (DCM/MeOH, 50:1, v/v (UV))

NMR Spectroscopy:

^{1}H NMR (500 MHz, DMSO-d$_6$, 25 °C, δ): 8.51 (d, *J* = 7.5 Hz, 2H), 8.07 (d, *J* = 7.0 Hz, 2H), 7.92 (d, *J* = 7.0 Hz, 2H), 7.84 (d, *J* = 7.8 Hz, 2H), 3.86 (s, 3H).

^{13}C {^{1}H} NMR (126 MHz, DMSO-d$_6$, 25 °C, δ): 151.4 (d, *J* = 251.5 Hz), 151.0 (d, *J* = 10.3 Hz), 135.2, 135.0, 134.7, 130.2, 129.6, 126.3 (d, *J* = 3.7 Hz), 119.6, 116.3 (d, *J* = 22.8 Hz), 115.3 (d, *J* = 2.1 Hz), 114.8 (d, *J* = 7.2 Hz), 56.7.

^{19}F NMR (471 MHz, DMSO-d$_6$, 25 °C, δ): –134.5, –148.2, –148.3.

HRMS-ESI (m/z) calc'd for $C_{19}H_{14}O_1F_1S_2$ [M–BF_4]$^+$, 341.0465; found, 341.0464; deviation: 0.1 ppm.

Anisole-derived thianthrenium salt 3a-BF$_4$

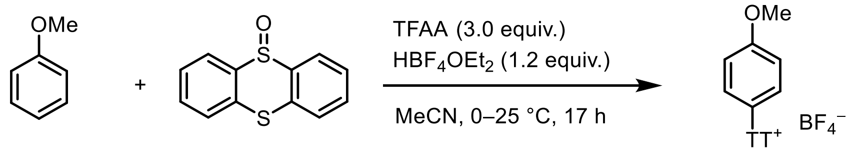

3a-BF$_4$, 91%

The compound **3a-BF$_4$** was prepared according to the modified versions of the reported procedures and the NMR spectra are in good accordance with reported data.[3] Under an ambient atmosphere, a 25 mL round-bottom flask equipped with a stir bar was charged with thianthrene-S-oxide (232 mg, 1.00 mmol, 1.00 equiv.), MeCN (10 mL, *c* = 0.1 M), and anisole (109 μL, 108 mg, 1.0 mmol, 1.0 equiv.). After cooling to 0 °C, TFAA (417 μL, 630 mg, 3.00 mmol, 3.00 equiv.) and then HBF_4·OEt_2 (163 μL, 194 mg, 1.20 mmol, 1.20 equiv.) were subsequently added to the vial while stirring the reaction mixture. The reaction mixture was stirred at 0 °C for 1 hour, and then stirred at 25 °C for 16 hours. The solvent was removed under reduced pressure by rotary evaporation and the residue was dissolved in DCM (10 mL). The resulting solution was washed with

saturated aqueous $NaHCO_3$ solution (10 mL) and aqueous $NaBF_4$ solution (2 × 10 mL, 10% w/w). The organic phase was dried over Na_2SO_4, filtered, and the solvent was removed under reduced pressure. The residue was purified by chromatography on silica gel eluting with DCM/MeOH (50:1 to 20:1, v/v) to afford **3a-BF$_4$** (370 mg, 90%) as a colorless solid.

R*f* = 0.30 (DCM/MeOH, 50:1, v/v (UV))

NMR Spectroscopy:

^{1}H NMR (500 MHz, DMSO-d$_6$, 25 °C, δ): 8.50 (d, *J* = 8.0 Hz, 2H), 8.06 (d, *J* = 8.0 Hz, 2H), 7.90 (t, *J* = 8.1 Hz, 2H), 7.83 (t, *J* = 7.4 Hz, 2H), 7.25 (d, *J* = 10.2 Hz, 2H), 7.12 (d, *J* = 8.7 Hz, 2H), 3.77 (s, 3H).

^{13}C {^{1}H} NMR (126 MHz, DMSO-d$_6$, 25 °C, δ): 162.7, 134.9, 134.5, 130.4, 130.1, 129.6, 127.8, 120.0, 116.2, 114.3, 55.9.

^{19}F NMR (471 MHz, DMSO-d$_6$, 25 °C, δ): –148.2 (bs), –148.3 (bs).

HRMS-ESI (m/z) calc'd for $C_{19}H_{15}O_1S_2$ $[M–BF_4]^+$, 323.0559; found, 323.0557; deviation: 0.6 ppm.

Benzene-derived thianthrenium salt 3c-BF$_4$

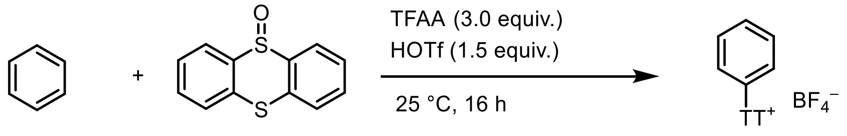

3c-BF$_4$, 63%

The compound **3c-BF$_4$** was prepared according to the modified versions of the reported procedures and the NMR spectra are in good accordance with reported data.[3] Under an ambient atmosphere, a 10 mL round-bottom flask equipped with a stir bar was charged with thianthrene-S-oxide (232 mg, 1.00 mmol, 1.00 equiv.), benzene (1.00 mL, 875 mg, 11.2 mmol, 11.2 equiv.). Trifluoromethanesulfonic acid (133 µL, 225 mg, 1.50 mmol, 1.50 equiv.) was added to the vial while stirring the reaction mixture, followed by TFAA (417 µL, 630 mg, 3.00 mmol, 3.00 equiv.). The reaction mixture was stirred at 25 °C for 16 hour. The solvent was removed under reduced pressure by rotary evaporation and the residue was dissolved in DCM (10 mL). The resulting solution was washed with saturated aqueous $NaHCO_3$ solution (10 mL) and aqueous $NaBF_4$ solution (2 × 10 mL, 10% w/w). The organic phase was dried over Na_2SO_4, filtered, and the solvent was removed under reduced pressure. The residue was purified by chromatography on silica gel eluting with DCM/MeOH (50:1 to 20:1, v/v) to afford **3c-BF$_4$** (240 mg, 63%) as a colorless solid.

R*f* = 0.35 (DCM/MeOH, 15:1, v/v (UV))

NMR Spectroscopy:

^{1}H NMR (500 MHz, CD_3CN, 25 °C, δ): 8.37 (d, *J* = 7.7 Hz, 2H), 7.96 (d, *J* = 8.0 Hz, 2H), 7.90 (t, *J* = 7.1 Hz, 2H), 7.83 (t, *J* = 7.7 Hz, 2H), 7.61 (t, *J* = 7.8 Hz, 1H), 7.48 (t, *J* = 7.3 Hz, 2H), 7.11 (t, *J* = 7.7 Hz, 2H).

^{13}C {^{1}H} NMR (126 MHz, CD_3CN, 25 °C, δ): 137.7, 136.1, 136.0, 133.9, 131.7, 131.5, 130.9, 128.9, 124.9, 119.5.

^{19}F NMR (471 MHz, CD_3CN, 25 °C, δ): –151.7 (bs), –151.8 (bs).

HRMS-ESI (m/z) calc'd for $C_{18}H_{13}S_2$ [M–BF_4]$^+$, 293.0453; found,293.0452; deviation: 0.6 ppm.

Methylbenzoate-derived thianthrenium salt 3d-BF_4

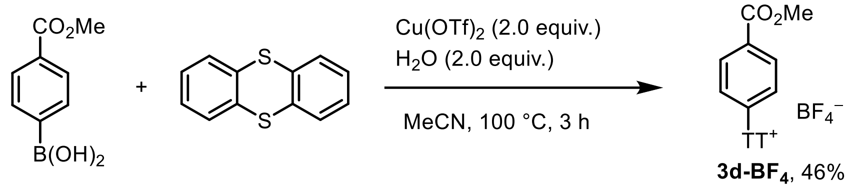

3d-BF$_4$, 46%

The compound **3d-BF4** was prepared according to the modified versions of the reported procedures.[14] Under an argon atmosphere, a 10 mL Schlenk tube equipped with a stir bar was charged with thianthrene (324 mg, 1.50 mmol, 1.50 equiv.) and 4-(methoxycarbonyl)phenylboronic acid (178 mg, 1.00 mmol, 1.00 equiv.). The Schlenk tube was transferred into a nitrogen-filled glovebox. $Cu(OTf)_2$ (723 mg, 2.00 mmol, 2.00 equiv.) was added and the Schlenk tube was sealed and removed from the glovebox. Subsequently, water (36 µL, 36 mg, 2.0 mmol, 2.0 equiv.) and MeCN (1.0 mL) were added into the tube. The reaction mixture was stirred at 100 °C for 3 hour. The reaction mixture was added into NH_3•H_2O (30 wt% in H_2O, 20 mL) after cooling to 25 °C. Then, the reaction mixture was extracted with DCM (20 mL). The resulting organic layer was collected and washed with aqueous $NaBF_4$ solution (2 × 20 mL, 10% w/w). The organic phase was dried over Na_2SO_4, filtered, and the solvent was removed under reduced pressure. The residue was purified by chromatography on silica gel eluting with DCM/MeOH (50:1 to 20:1, v/v) to afford **3d-BF$_4$** (201 mg, 46%) as a colorless solid.

R*f* = 0.35 (DCM/MeOH, 15:1, v/v (UV))

NMR Spectroscopy:

^{1}H NMR (500 MHz, CD_3CN, 25 °C, δ): 8.45 (dd, *J* = 7.3 Hz, 2H), 8.01 (t, *J* = 7.6 Hz, 4H), 7.95 (t, *J* = 8.3 Hz, 2H), 7.87 (t, *J* = 8.8 Hz, 2H), 7.21 (t, *J* = 8.8 Hz, 2H), 3.89 (s, 3H).

^{13}C {^{1}H} NMR (126 MHz, CD_3CN, 25 °C, δ): 165.9, 137.9, 136.4, 136.3, 135.2, 131.8, 131.7, 131.0, 129.7, 129.3, 119.2, 53.4.

^{19}F NMR (471 MHz, CD_3CN, 25 °C, δ): –151.6 (bs), –151.7 (bs).

HRMS ESI (m/z) calc'd for $C_{20}H_{15}O_2S_2^+$ [M–BF_4]$^+$, 351.0508; found, 351.0508. Deviation: 0.03 ppm.

Nitrobenzene-derived thianthrenium salt 3e-BF_4

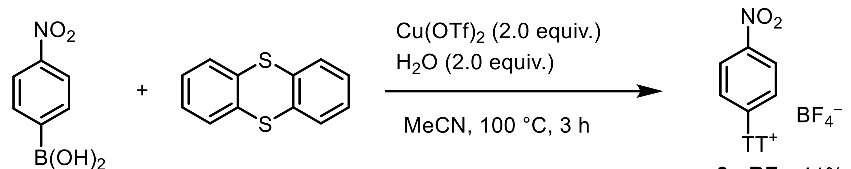

3e-BF4, 44%

The compound **3e-BF$_4$** was prepared according to the modified versions of the reported procedures[14] and the NMR spectra are in good accordance with reported data.[3] Under an argon atmosphere, a 10 mL Schlenk tube equipped with a stir bar was charged with thianthrene (324 mg, 1.50 mmol, 1.50 equiv.), 4-nitrobenzeneboronic acid (167 mg, 1.00 mmol, 1.00 equiv.). The Schlenk tube was transferred into a nitrogen-filled glovebox. $Cu(OTf)_2$ (723 mg, 2.00 mmol, 2.00 equiv.) was added and the Schlenk tube was sealed and removed from the glovebox. Subsequently, water (36 μL, 36 mg, 2.0 mmol, 2.0 equiv.) and MeCN (1.0 mL) were added into the tube. The reaction mixture was stirred at 100 °C for 3 hour. The reaction mixture was added into NH_3•H_2O (30 wt% in H_2O, 20 mL) after cooling to 25 °C. Then, the reaction mixture was extracted with DCM (20 mL). The resulting organic layer was collected and washed with aqueous $NaBF_4$ solution (2 × 20 mL, 10% w/w). The organic phase was dried over Na_2SO_4, filtered, and the solvent was removed under reduced pressure. The residue was purified by chromatography on silica gel eluting with DCM/MeOH (50:1 to 20:1, v/v) to afford **3e-BF$_4$** (187 mg, 44%) as a colorless solid.

R*f* = 0.35 (DCM/MeOH, 15:1, v/v (UV))

NMR Spectroscopy:

^{1}H NMR (500 MHz, $CDCl_3$, 25 °C, δ): 8.69 (d, *J* = 6.7 Hz, 2H), 8.21 (d, *J* = 8.2 Hz, 2H), 7.93 – 7.83 (m, 6H), 7.36 (d, *J* = 8.5 Hz, 2H).

^{13}C {^{1}H} NMR (126 MHz, $CDCl_3$, 25 °C, δ): 150.2, 136.9, 136.3, 135.6, 131.3, 130.8, 130.7, 129.6, 125.2, 118.4.

^{19}F NMR (471 MHz, $CDCl_3$, 25 °C, δ): –149.8 (bs), –149.9 (bs).

HRMS ESI (m/z) calc'd for $C_{18}H_{12}O_2N_1S_2^+$ $[M–BF_4]^+$, 338.0304; found, 338.0302. Deviation: 0.5 ppm.

Radical-clock thianthrenium salt 4-BF_4

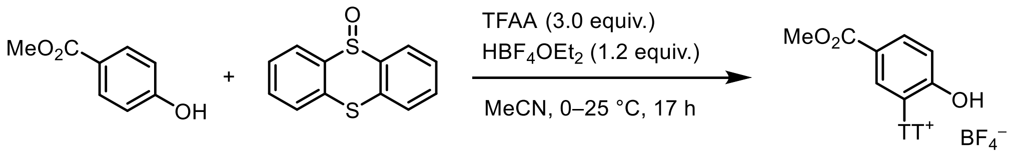

4-1

The compound **4-1** and **4-BF4** were prepared according to the reported procedures and the NMR spectra are in good accordance with reported data.[3] Under an ambient atmosphere, a 25 mL vial equipped with a stir bar

was charged with thianthrene-S-oxide (232 mg, 1.00 mmol, 1.00 equiv.), 4-hydroxybenzoate (152 mg, 1.00 mmol, 1.00 equiv.) and MeCN (4 mL, *c* = 0.25 M). After cooling to 0 °C, TFAA (417 µL, 640 mg, 3.00 mmol, 3.00 equiv.) and then $HBF_4{\cdot}OEt_2$ (174 µL, 1.20 mmol, 1.20 equiv.) were subsequently added to the vial while stirring the reaction mixture. The reaction mixture was stirred at 0 °C for 1 hour, and then stirred at 25 °C for 16 hours. The solvent was removed under reduced pressure by rotary evaporation and the residue was dissolved in DCM (10 mL). The resulting solution was washed with saturated aqueous $NaHCO_3$ solution (10 mL) and aqueous $NaBF_4$ solution (2 × 10 mL, 10% w/w). The organic phase was dried over Na_2SO_4, filtered, and the solvent was removed under reduced pressure. The residue was rinsed with 2 mL DCM, and the resulting colorless solid was filtered off, collected and dried in vacuo to afford **4-1** (404 mg, 0.889 mmol, 89%) as a colorless solid. The solid was used for the next step without purification.

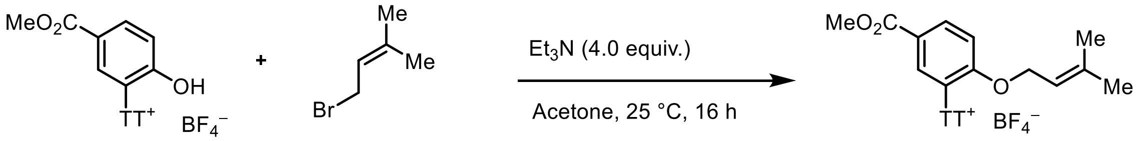

4-1

4-BF$_4$, 54%

Under ambient atmosphere, a 25 mL vial equipped with a stir bar was charged with **4-1** (227 mg, 0.500 mmol, 1.00 equiv.) and acetone (5 mL, *c* = 0.1 M). After addition of trimethylamine (279 µL, 202 mg, 2.00 mmol, 4.00 equiv) was subsequently added into the reaction mixture at 25 °C, followed by the addition of prenylbromide (231 µL, 298 mg, 2.00 mmol, 4.00 equiv.). The reaction mixture was stirred at 25 °C for 16 hours. Then the solvent was removed under reduced pressure by rotary evaporation and the residue was dissolved in DCM (10 mL). The resulting solution was washed with water (10 mL). The DCM layer was collected, and the aqueous layer was further extracted with DCM (2 × 10 mL). The combined DCM solution was washed with aqueous $NaBF_4$ solution (2 × 10 mL, 10% w/w). The organic phase was dried over Na_2SO_4, filtered, and the solvent was removed under reduced pressure. The residue was purified by chromatography on silica gel eluting with DCM/MeOH (50:1 to 20:1, v/v) to afford **4-BF$_4$** (142 mg, 54%) as a colorless solid.

R*f* = 0.35 (DCM/MeOH, 15:1, v/v (UV))

NMR Spectroscopy:

^{1}H NMR (300 MHz, CD_2Cl_2, 25 °C, δ): 8.29 (d, *J* = 8.1 Hz, 2H), 8.22 (dd, *J* = 8.7, 2.1 Hz, 1H), 7.94 (d, *J* = 7.1 Hz, 2H), 7.86 (t, *J* = 7.4 Hz, 2H), 7.76 (t, *J* = 7.1 Hz, 2H), 7.23 (d, *J* = 1.7 Hz, 1H), 7.17 (d, *J* = 8.7 Hz, 1H), 5.48 (t, *J* = 7.5 Hz, 1H), 4.76 (d, *J* = 7.8 Hz, 2H), 3.79 (s, 3H), 1.90 (s, 3H), 1.77 (s, 3H).

^{13}C {^{1}H} NMR (76 MHz, CD_2Cl_2, 25 °C, δ): 164.8, 161.0, 142.8, 138.2, 137.4, 135.6, 135.4, 131.1, 130.8, 130.4, 124.2, 117.1, 116.9, 114.7, 109.1, 68.1, 52.9, 26.0, 18.5.

^{19}F NMR (282 MHz, CD_2Cl_2, 25 °C, δ): –152.4 (bs), –152.5 (bs).

HRMS ESI (m/z) calc'd for $C_{25}H_{23}O_3S_2^+$ $[M–BF_4]^+$, 435.1083; found, 435.1082. Deviation: 0.4 ppm.

Boscalid-derived thianthrenium salt 5-BF_4

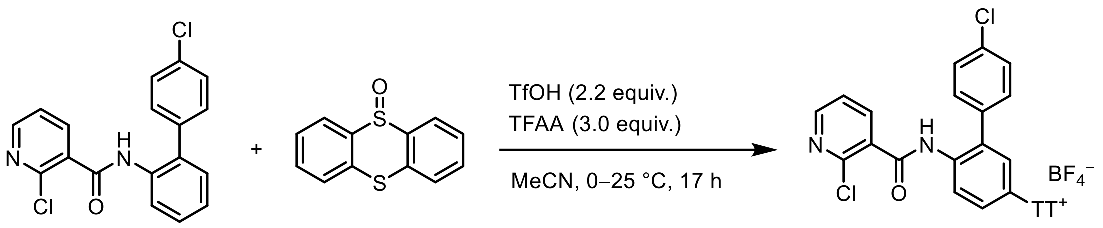

5-BF$_4$, 82%

Under an ambient atmosphere, a 25 mL round-bottom flask equipped with a stir bar was charged with thianthrene-S-oxide (232 mg, 1.00 mmol, 1.00 equiv.), boscalid (343 mg, 1.00 mmol, 1.00 equiv.) and MeCN (10 mL, *c* = 0.1 M). After cooling to 0 °C, TfOH (195 µL, 2.20 mmol, 2.20 equiv.) and then TFAA (417 µL, 3.00 mmol, 3.00 equiv.) were subsequently added to the vial while stirring the reaction mixture. The reaction mixture was stirred at 0 °C for 1 hour, and then stirred at 25 °C for 16 hours. The solvent was removed under reduced pressure by rotary evaporation and the residue was dissolved in DCM (10 mL). The resulting solution was washed with saturated aqueous $NaHCO_3$ solution (10 mL) and aqueous $NaBF_4$ solution (2 × 10 mL, 10% w/w). The organic phase was dried over Na_2SO_4, filtered, and the solvent was removed under reduced pressure. The residue was purified by chromatography on silica gel eluting with DCM/MeOH (50:1 to 20:1, v/v) to afford **5-BF$_4$** (532 mg, 82%) as a colorless solid.

R_f = 0.23 (DCM/MeOH, 15:1, v/v (UV))

NMR Spectroscopy:

^{1}H NMR (300 MHz, CD_3CN, 23 °C, δ): 8.44 (s, 1H), 8.41 (dd, *J* = 4.8, 1.8 Hz, 1H), 8.35 (dd, *J* = 7.9, 1.2 Hz, 2H), 8.27 (d, *J* = 9.2 Hz, 1H), 7.99 (dd, *J* = 7.8, 1.3 Hz, 2H), 7.89 (td, *J* = 7.3, 1.2 Hz, 2H), 7.85 – 7.77 (m, 3H), .7.47 – 7.41 (m, 2H), 7.38 (dd, *J* = 7.5, 4.7 Hz, 1H), 7.29 – 7.24 (m, 2H), 7.19 (dd, *J* = 9.1, 2.6 Hz, 1H), 7.07 (d, *J* = 2.5 Hz, 1H).

^{13}C NMR (126 MHz, CD_3CN, 23 °C, δ): 165.1, 152.0, 147.6, 140.0, 139.1, 137.5, 136.3, 136.1, 135.9, 135.44, 135.42, 132.8, 131.9, 131.7, 131.0, 130.9, 130.1, 129.4, 125.7, 123.8, 120.1, 119.6.

^{19}F NMR (282 MHz, CD_3CN, 23 °C, δ): –151.7 (bs), –151.8 (bs).

HRMS ESI (m/z) calc'd for $C_{30}H_{19}O_1N_2S_2Cl_2^+$ [M–BF_4]$^+$, 557.0310; found, 557.0310. Deviation: 0.1 ppm.

Xanthon-derived thianthrenium salt 6-BF_4

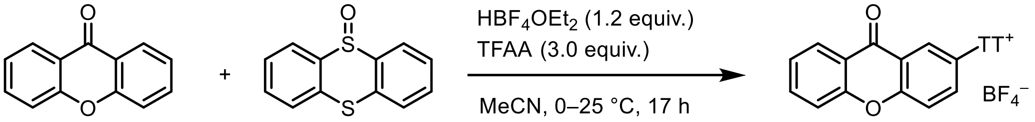

6-BF$_4$, 62%

The compound **6-BF$_4$** was prepared according to the modified versions of the reported procedures and the

NMR spectra are in good accordance with reported data.[15] Under an ambient atmosphere, a 25 mL round-bottom flask equipped with a stir bar was charged with thianthrene-S-oxide (232 mg, 1.00 mmol, 1.00 equiv.), xanthon (196 mg, 1.00 mmol, 1.00 equiv.) and MeCN (10 mL, *c* = 0.1 M). After cooling to 0 °C, TFAA (417 µL, 630 mg, 3.00 mmol, 3.00 equiv.) and then $HBF_4{\cdot}OEt$ (163 µL, 194 mg, 1.20 mmol, 1.20 equiv.) were subsequently added to the vial while stirring the reaction mixture. The reaction mixture was stirred at 0 °C for 1 hour, and then stirred at 25 °C for 16 hours. The solvent was removed under reduced pressure by rotary evaporation and the residue was dissolved in DCM (10 mL). The resulting solution was washed with saturated aqueous $NaHCO_3$ solution (10 mL) and aqueous $NaBF_4$ solution (2 × 10 mL, 10% w/w). The organic phase was dried over Na_2SO_4, filtered, and the solvent was removed under reduced pressure. The residue was purified by chromatography on silica gel eluting with DCM/MeOH (50:1 to 20:1, v/v) to afford **6-BF₄** (308 mg, 62%) as a yellow solid.

R*f* = 0.30 (DCM/MeOH, 50:1, v/v (UV))

NMR Spectroscopy:

^{1}H NMR (500 MHz, CD_3CN, 23 °C, δ): 8.46 (dd, *J* = 8.1, 1.1 Hz, 2H), 8.12 (dd, *J* = 8.0, 1.3 Hz, 1H), 7.98 (dd, *J* = 7.8, 1.3 Hz, 2H), 7.93 (td, *J* = 7.4, 1.2 Hz, 2H), 7.89 – 7.81 (m, 4H), 7.62 (d, *J* = 8.8 Hz, 1H), 7.53 (d, *J* = 8.4 Hz, 1H), 7.47 (dd, *J* = 9.2, 2.7 Hz, 1H), 7.44 (t, *J* = 7.2 Hz, 1H).

^{13}C NMR (126 MHz, CD_3CN, 23 °C, δ): 175.8, 159.0, 156.8, 137.5, 137.2, 136.3, 136.1, 134.2, 131.7, 131.0, 128.2, 127.1, 126.2, 123.4, 122.2, 120.1, 119.4, 119.2.

^{19}F NMR (565 MHz, CD_3CN, 23 °C, δ): –151.4 (bs), –151.7 (bs).

HRMS ESI (m/z) calc'd for $C_{25}H_{15}O_2S_2^+$ $[M–BF_4]^+$, 411.0508; found, 411.0506. Deviation: 0.4 ppm.

Biphenyl-derived thianthrenium salt 7-BF₄

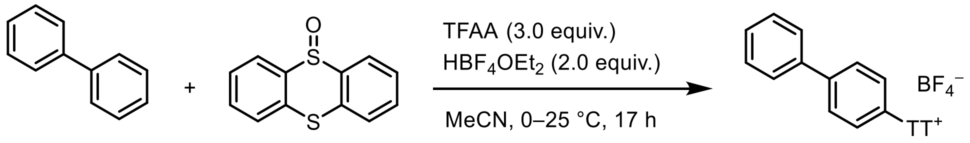

7-BF₄, 80%

The compound **7-BF₄** was prepared according to the modified versions of the reported procedures and the NMR spectra are in good accordance with reported data.[3] Under an ambient atmosphere, a 25 mL round-bottom flask equipped with a stir bar was charged with thianthrene-S-oxide (232 mg, 1.00 mmol, 1.00 equiv.), biphenyl (154 mg, 1.00 mmol, 1.00 equiv.) and MeCN (10 mL, *c* = 0.1 M). After cooling to 0 °C, TFAA (417 µL, 630 mg, 3.00 mmol, 3.00 equiv.) and then $HBF_4{\cdot}OEt_2$ (272 µL, 323 mg, 2.00 mmol, 2.00 equiv.) were subsequently added to the vial while stirring the reaction mixture. The reaction mixture was stirred at 0 °C for 1 hour, and then stirred at 25 °C for 16 hours. The solvent was removed under reduced pressure by rotary evaporation and the residue was dissolved in DCM (10 mL). The resulting solution was washed with saturated aqueous $NaHCO_3$ solution (10 mL) and aqueous $NaBF_4$ solution (2 × 10 mL, 10% w/w). The

organic phase was dried over Na_2SO_4, filtered, and the solvent was removed under reduced pressure. The residue was purified by chromatography on silica gel eluting with DCM/MeOH (50:1 to 20:1, v/v) to afford **7-BF$_4$** (367 mg, 80%) as a colorless solid.

R*f* = 0.35 (DCM/MeOH, 15:1, v/v (UV))

NMR Spectroscopy:

^{1}H NMR (300 MHz, CD_3CN, 25 °C, δ): 8.40 (d, *J* = 7.7 Hz, 2H), 7.99 (dd, *J* = 7.7, 1.4 Hz, 2H), 7.91 (td, *J* = 7.1, 1.1 Hz, 2H), 7.84 (td, *J* = 7.9, 1.5 Hz, 2H), 7.76 – 7.69 (m, 2H), 7.62 – 7.57 (m, 2H), 7.51 – 7.42 (m, 3H), 7.23 – 7.17 (m, 2H).

^{13}C {^{1}H} NMR (76 MHz, CD_3CN, 25 °C, δ): 146.5, 139.0, 137.6, 136.1, 136.0, 131.7, 130.9, 130.1, 130.0, 129.8, 129.5, 128.2, 123.3, 119.5.

^{19}F NMR (282 MHz, CD_3CN, 25 °C, δ): –151.7 (bs), –151.8 (bs).

HRMS ESI (m/z) calc'd for $C_{24}H_{17}S_2^+$ $[M–BF_4]^+$, 369.0766; found, 369.0764. Deviation: 0.7 ppm.

Cyclopropylbenzene-derived thianthrenium salt 9-BF$_4$

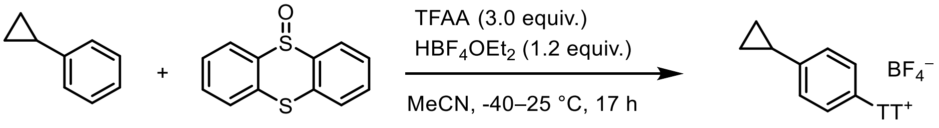

9-BF$_4$, 83%

The compound **9-BF$_4$** was prepared according to the modified versions of the reported procedures and the NMR spectra are in good accordance with reported data.[3] Under an ambient atmosphere, a 25 mL round-bottom flask equipped with a stir bar was charged with thianthrene-S-oxide (232 mg, 1.00 mmol, 1.00 equiv.), cyclopropylbenzene (126 µL, 118 mg, 1.00 mmol, 1.00 equiv.) and MeCN (10 mL, *c* = 0.1 M). After cooling to −40 C, TFAA (417 µL, 630 mg, 3.00 mmol, 3.00 equiv.) and then $HBF_4·OEt_2$ (163 µL, 194 mg, 1.20 mmol, 1.20 equiv.) were subsequently added to the vial while stirring the reaction mixture. The reaction mixture was stirred at −40 C for 1 hour, and then stirred at 25 °C for 16 hours. The solvent was removed under reduced pressure by rotary evaporation and the residue was dissolved in DCM (10 mL). The resulting solution was washed with saturated aqueous $NaHCO_3$ solution (10 mL) and aqueous $NaBF_4$ solution (2 × 10 mL, 10% w/w). The organic phase was dried over Na_2SO_4, filtered, and the solvent was removed under reduced pressure. The residue was purified by chromatography on silica gel eluting with DCM/MeOH (50:1 to 20:1, v/v) to afford **9-BF$_4$** (350 mg, 83%) as a colorless solid.

R*f* = 0.40 (DCM/MeOH, 20:1, v/v (UV))

NMR Spectroscopy:

^{1}H NMR (300 MHz, CD_2Cl_2, 25 °C, δ): 8.37 (d, *J* = 8.0 Hz, 2H), 7.93 – 7.74 (m, 6H), 7.16 – 7.10 (m, 2H), 7.06 – 7.00 (m, 2H), 1.90 (ddd, *J* = 13.0, 8.5, 5.2 Hz, 1H), 1.12 – 1.01 (m, 2H), 0.75 – 0.67 (m, 2H).

^{13}C {^{1}H} NMR (76 MHz, CD_2Cl_2, 25 °C, δ): 152.0, 137.1, 135.4, 134.9, 131.0, 130.6, 128.3, 128.2, 119.4, 119.1, 15.8, 11.2.

^{19}F NMR (282 MHz, CD_2Cl_2, 25 °C, δ): –151.6 (bs), –151.7 (bs).

HRMS ESI (m/z) calc'd for $C_{21}H_{17}S_2^+$ $[M–BF_4]^+$, 333.0766; found, 333.0763. Deviation: 1.1 ppm.

2-Fluoro-6-phenoxybenzonitrile-derived thianthrenium salt 10-BF$_4$

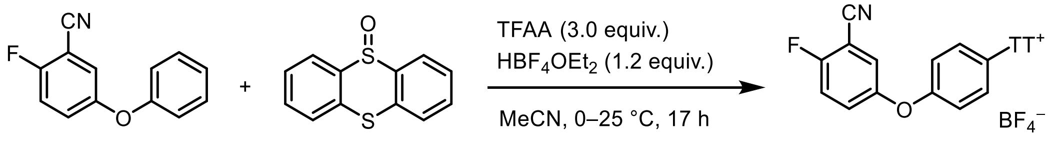

10-BF$_4$, 80%

The compound **10-BF$_4$** was prepared according to the modified versions of the reported procedures and the NMR spectra are in good accordance with reported data.[10] Under an ambient atmosphere, a 25 mL round-bottom flask equipped with a stir bar was charged with thianthrene-S-oxide (232 mg, 1.00 mmol, 1.00 equiv.), 2-fluoro-6-phenoxybenzonitrile (213 mg, 1.0 mmol, 1.0 equiv.) and MeCN (10 mL, *c* = 0.1 M). After cooling to 0 C, TFAA (417 μL, 630 mg, 3.00 mmol, 3.00 equiv.) and then $HBF_4·OEt_2$ (163 μL,194 mg, 1.20 mmol, 1.20 equiv.) were subsequently added to the vial while stirring the reaction mixture. The reaction mixture was stirred at 0 C for 1 hour, and then stirred at 25 °C for 16 hours. The solvent was removed under reduced pressure by rotary evaporation and the residue was dissolved in DCM (10 mL). The resulting solution was washed with saturated aqueous $NaHCO_3$ solution (10 mL) and aqueous $NaBF_4$ solution (2 × 10 mL, 10% w/w). The organic phase was dried over Na_2SO_4, filtered, and the solvent was removed under reduced pressure. The residue was purified by chromatography on silica gel eluting with DCM/MeOH (50:1 to 20:1, v/v) to afford **10-BF$_4$** (413 mg, 80%) as a colorless solid.

R_f = 0.30 (DCM/MeOH, 15:1, v/v (UV))

NMR Spectroscopy:

^{1}H NMR (300 MHz, CD_2Cl_2, 25 °C, δ): 8.45 (d, *J* = 7.6 Hz, 2H), 7.97 – 7.77 (m, 6H), 7.58 (q, *J* = 7.0 Hz, 1H), 7.25 (d, *J* = 8.3 Hz, 2H), 7.15 (d, *J* = 8.1 Hz, 2H), 7.06 (t, *J* = 8.1 Hz, 1H), 6.82 (d, *J* = 8.3 Hz, 1H).

^{13}C {^{1}H} NMR (76 MHz, CD_2Cl_2, 25 °C, δ): 164.4 (d, *J* = 269.7 Hz), 159.7, 158.2 (d, *J* = 3.8 Hz), 137.2, 136.2 (d, *J* = 9.9 Hz), 135.6, 135.2, 131.1, 131.0, 130.7, 121.2, 119.1, 118.9, 115.1 (d, *J* = 3.8 Hz), 112.5 (d, *J* = 20.4 Hz), 110.8, 95.4.

^{19}F NMR (282 MHz, CD_2Cl_2, 25 °C, δ): -104.6, –151.1 (bs), –151.2 (bs).

HRMS ESI (m/z) calc'd for $C_{25}H_{15}O_1N_1F_1S_2^+$ $[M–BF_4]^+$, 428.0574; found, 428.0571. Deviation: 0.7 ppm.

o-Xylene-derived thianthrenium salt 11-BF_4

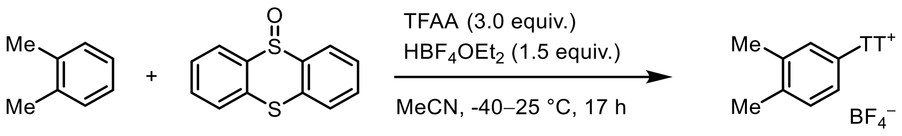

11-BF_4, 85%

The compound **11-BF_4** was prepared according to the modified versions of the reported procedures and the NMR spectra are in good accordance with reported data.[11] Under an ambient atmosphere, a 25 mL round-bottom flask equipped with a stir bar was charged with thianthrene-S-oxide (232 mg, 1.00 mmol, 1.00 equiv.), o-xylene (121 µL, 106 mg, 1.0 mmol, 1.0 equiv.) and MeCN (10 mL, *c* = 0.1 M). After cooling to –40 C, TFAA (417 µL, 630 mg, 3.00 mmol, 3.00 equiv.) and then $HBF_4 \cdot OEt_2$ (204 µL, 242 mg, 1.50 mmol, 1.50 equiv.) were subsequently added to the vial while stirring the reaction mixture. The reaction mixture was stirred at –40 C for 1 hour, and then stirred at 25 °C for 16 hours. The solvent was removed under reduced pressure by rotary evaporation and the residue was dissolved in DCM (10 mL). The resulting solution was washed with saturated aqueous $NaHCO_3$ solution (10 mL) and aqueous $NaBF_4$ solution (2 × 10 mL, 10% w/w). The organic phase was dried over Na_2SO_4, filtered, and the solvent was removed under reduced pressure. The residue was purified by chromatography on silica gel eluting with DCM/MeOH (50:1 to 30:1, v/v) to afford **11-BF_4** (348 mg, 85%) as a colorless solid.

R_f = 0.40 (DCM/MeOH, 10:1, v/v (UV))

NMR Spectroscopy:

^{1}H NMR (300 MHz, CD_3CN, 25 °C, δ): 8.33 (dd, *J* = 7.7, 1.2 Hz, 2H), 7.93 (dd, *J* = 7.7, 1.5 Hz, 2H), 7.87 (td, *J* = 7.6, 1.4 Hz, 2H), 7.80 (td, *J* = 7.5, 1.8 Hz, 2H), 7.23 (d, *J* = 8.2 Hz, 1H), 6.96 (d, *J* = 2.0 Hz, 1H), 6.83 (dd, *J* = 8.4, 2.3 Hz, 1H), 2.23 (s, 3H), 2.16 (s, 3H).

^{13}C {^{1}H} NMR (76 MHz, CD_3CN, 25 °C, δ): 144.3, 141.3, 137.3, 135.9, 135.7, 132.4, 131.5, 130.8, 129.2, 126.4, 121.1, 119.6, 19.8, 19.7.

^{19}F NMR (282 MHz, CD_3CN, 25 °C, δ): –151.3 (bs), –151.4 (bs).

HRMS ESI (m/z) calc'd for $C_{20}H_{17}S_2^+$ $[M–BF_4]^+$, 321.0766; found, 321.0765. Deviation: 0.5 ppm.

Bifonazole-derived thianthrenium salt 12-BF_4

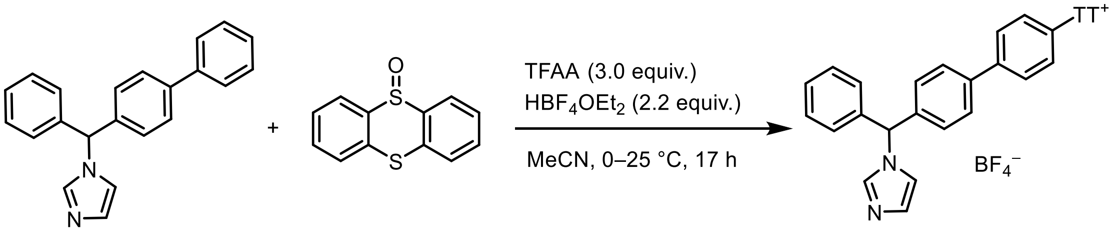

12-BF_4, 90%

The compound **12-BF$_4$** was prepared according to the modified versions of the reported procedures and the NMR spectra are in good accordance with reported data.[8] Under an ambient atmosphere, a 25 mL round-bottom flask equipped with a stir bar was charged with thianthrene-S-oxide (232 mg, 1.00 mmol, 1.00 equiv.), bifonazole (310 mg, 1.0 mmol, 1.0 equiv.) and MeCN (10 mL, *c* = 0.1 M). After cooling to 0 C, TFAA (417 µL, 630 mg, 3.00 mmol, 3.00 equiv.) and then $HBF_4{\cdot}OEt_2$ (299 µL, 356 mg, 2.20 mmol, 2.20 equiv.) were subsequently added to the vial while stirring the reaction mixture. The reaction mixture was stirred at 0 C for 1 hour, and then stirred at 25 °C for 16 hours. The solvent was removed under reduced pressure by rotary evaporation and the residue was dissolved in DCM (10 mL). The resulting solution was washed with saturated aqueous $NaHCO_3$ solution (10 mL) and aqueous $NaBF_4$ solution (2 × 10 mL, 10% w/w). The organic phase was dried over Na_2SO_4, filtered, and the solvent was removed under reduced pressure. The residue was purified by chromatography on silica gel eluting with DCM/MeOH (50:1 to 10:1, v/v) to afford **12-BF$_4$** (550 mg, 90%) as a colorless solid.

R$_f$ = 0.30 (DCM/MeOH, 20:1, v/v (UV))

NMR Spectroscopy:

^{1}H NMR (300 MHz, DMSO-d$_6$, 25 °C, δ): 8.62 (dd, *J* = 7.7, 1.0 Hz, 2H), 8.09 (dd, *J* = 7.8, 1.3 Hz, 2H), 7.94 (td, *J* = 7.5, 1.5 Hz, 2H), 7.89 (dd, *J* = 7.6, 1.6 Hz, 2H), 7.85 (d, *J* = 8.8 Hz, 2H), 7.69 (d, *J* = 8.5 Hz, 2H), 7.46 – 7.36 (m, 3H), 7.34 – 7.24 (m, 4H), 7.20 (d, *J* = 6.3 Hz, 2H), 7.01 (s, 1H). The three protons of the imidazolyl group did not give detectable nmr signals.

^{13}C {^{1}H} NMR (76 MHz, DMSO-d$_6$, 25 °C, δ): 143.5, 139.9, 138.9, 137.5, 135.7, 135.4, 134.8, 130.3, 129.7, 128.9, 128.7, 128.6, 128.5, 128.3, 127.9, 127.6, 123.8, 119.3, 63.5. The three carbon atoms of the imidazolyl group did not give detectable nmr signals.

^{19}F NMR (282 MHz, DMSO-d$_6$, 25 °C, δ): –148.2 (bs), –148.3 (bs).

HRMS ESI (m/z) calc'd for $C_{34}H_{25}N_2S_2^+$ $[M–BF_4]^+$, 525.1454; found, 525.1453. Deviation: 0.2 ppm.

Lidocaine-derived thianthrenium salt 13-BF$_4$

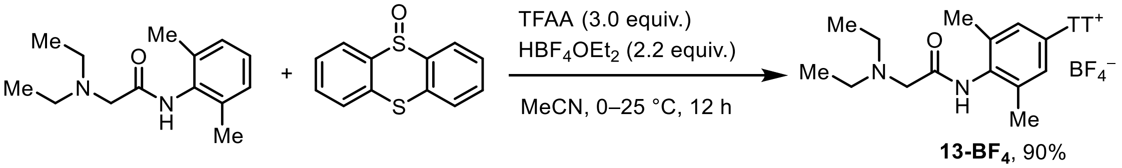

13-BF$_4$, 90%

The compound **13-BF$_4$** was prepared according to the modified versions of the reported procedures and the NMR spectra are in good accordance with reported data.[12] Under an ambient atmosphere, a 25 mL round-bottom flask equipped with a stir bar was charged with thianthrene-S-oxide (232 mg, 1.00 mmol, 1.00 equiv.), dry MeCN (3 mL, *c* = 0.33 M), and lidocaine (234 mg, 1.00 mmol, 1.00 equiv.). After cooling to 0 °C, $HBF_4{\cdot}OEt_2$ (0.14 mL, 0.16 g, 1.0 mmol, 1.0 equiv.) was added into the vial and the mixture was stirred for 5 minutes. Then TFAA (0.42 mL, 0.63 g, 0.30 mmol, 3.0 equiv.) and $HBF_4{\cdot}OEt_2$ (0.17 mL, 0.20 g, 1.2 mmol, 1.2 equiv.) were added to the vial while stirring the reaction mixture. The reaction mixture was stirred at 0 C

for 1 hour, and then stirred at 25 °C for 16 hours. The solvent was removed under reduced pressure by rotary evaporation and the residue was dissolved in DCM (10 mL). The resulting solution was washed with saturated aqueous NaHCO3 solution (10 mL) and aqueous NaBF4 solution (2 × 10 mL, 10% w/w). The organic phase was dried over Na_2SO_4, filtered, and the solvent was removed under reduced pressure. The residue was dissolved in ca. 4 mL DCM, and precipitated with ca. 20 mL Et_2O. The suspension was decanted, and the solid was dried in vacuo to afford **13-BF$_4$** (480 mg, 90%) as a colorless solid.

R*f* = 0.35 (DCM/MeOH, 20:1, v/v (UV))

NMR Spectroscopy:

^{1}H NMR (300 MHz, CD_3CN, δ) 8.58 (s, 1H), 8.36 (dd, *J* = 7.8, 1.4 Hz, 2H), 7.99 (dd, *J* = 8.0, 1.4 Hz, 2H), 7.91 (td, *J* = 7.8, 1.3 Hz, 2H), 7.83 (td, *J* = 7.8, 1.9 Hz, 2H), 6.87 (s, 2H), 4.08 (s, 2H), 3.26 (q, *J* = 7.1 Hz, 4H), 2.11 (s, 6H), 1.26 (t, *J* = 6.6 Hz, 6H).

^{13}C {^{1}H} NMR (76 MHz, CD_3CN, δ): 164.4, 140.0, 138.7, 137.6, 136.1, 136.0, 131.8, 131.0, 128.1, 123.2, 119.3, 54.4, 51.4, 18.7, 9.6.

^{19}F NMR (282 MHz, CD_3CN, 25 °C, δ): –151.4 (bs), –151.5 (bs).

HRMS ESI (m/z) calc'd for $C_{26}H_{29}O_1N_2S_2^+$ [M–BF$_4$]$^+$, 449.1716; found, 449.1714. Deviation: 0.3 ppm.

Pyriproxyfen-derived thianthrenium salt 14-BF$_4$

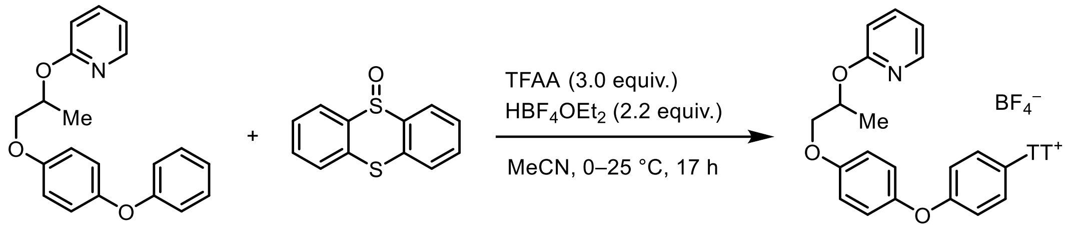

14-BF$_4$, 91%

The compound **14-BF$_4$** was prepared according to the modified versions of the reported procedures and the NMR spectra are in good accordance with reported data.[7] Under an ambient atmosphere, a 25 mL round-bottom flask equipped with a stir bar was charged with thianthrene-S-oxide (232 mg, 1.00 mmol, 1.00 equiv.), pyriproxyfen (321mg, 1.00 mmol, 1.00 equiv.) and MeCN (10 mL, *c* = 0.1 M). After cooling to 0 C, TFAA (417 µL, 630 mg, 3.00 mmol, 3.00 equiv.) and then $HBF_4 \cdot OEt_2$ (300 µL, 356 mg, 2.20 mmol, 2.20 equiv.) were subsequently added to the vial while stirring the reaction mixture. The reaction mixture was stirred at 0 C for 1 hour, and then stirred at 25 °C for 16 hours. The solvent was removed under reduced pressure by rotary evaporation and the residue was dissolved in DCM (10 mL). The resulting solution was washed with saturated aqueous $NaHCO_3$ solution (10 mL) and aqueous $NaBF_4$ solution (2 × 10 mL, 10% w/w). The organic phase was dried over Na_2SO_4, filtered, and the solvent was removed under reduced pressure. The residue was purified by chromatography on silica gel eluting with DCM/MeOH (50:1 to 20:1, v/v) to afford **14-BF$_4$** (570 mg, 91%) as a colorless solid.

R*f* = 0.40 (DCM/MeOH, 10:1, v/v (UV))

NMR Spectroscopy:

^{1}H NMR (300 MHz, DMSO-d$_6$, 25 °C, δ): 8.51 (d, *J* = 6.9 Hz, 2H), 8.15 (d, *J* = 5.2 Hz, 1H), 8.07 (d, *J* = 7.2 Hz, 2H), 7.91 (t, *J* = 6.9 Hz, 2H), 7.83 (t, *J* = 7.3 Hz, 2H), 7.69 (td, *J* = 7.4, 1.8 Hz, 1H), 7.27 (d, *J* = 8.9 Hz, 2H), 7.06 – 6.99 (m, 6H), 6.93 (dd, *J* = 6.9, 5.3 Hz, 1H), 6.77 (d, *J* = 8.2 Hz, 1H), 5.56 – 5.43 (m, 1H), 4.13 (qd, *J* = 10.1, 6.1 Hz, 2H), 1.34 (d, *J* = 6.4 Hz, 3H).

^{13}C {^{1}H} NMR (76 MHz, DMSO-d$_6$, 25 °C, δ): 162.6, 161.9, 155.8, 147.5, 146.8, 139.4, 135.1, 135.0, 134.6, 130.7, 130.2, 129.6, 121.7, 119.7, 118.2, 117.1, 116.4, 116.1, 111.2, 70.5, 69.0, 16.5.

^{19}F NMR (282 MHz, DMSO-d$_6$, 25 °C, δ): –148.2 (bs), –148.3 (bs).

HRMS ESI (m/z) calc'd for $C_{32}H_{26}O_3N_1S_2^+$ [M–BF$_4$]$^+$, 536.1349; found, 536.1349. Deviation: 0.03 ppm.

2-Methoxypyridine-derived thianthrenium salt 15-BF$_4$

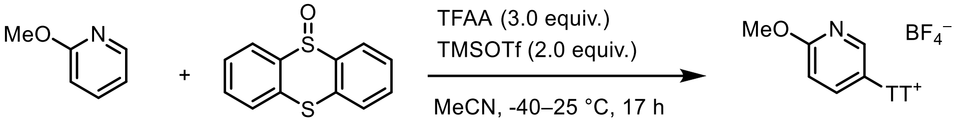

15-BF$_4$, 59%

The compound **15-BF$_4$** was prepared according to the modified versions of the reported procedures and the NMR spectra are in good accordance with reported data.[8] Under nitrogen ambient atmosphere, a 25 mL Schlenk-tube equipped with a stir bar was charged with thianthrene-S-oxide (232 mg, 1.00 mmol, 1.00 equiv.), 2-methoxypyridine (105 µL, 109 mg, 1.00 mmol, 1.00 equiv.) and dry MeCN (10 mL, *c* = 0.1 M). After cooling to –40 C, TFAA (417 µL, 630 mg, 3.00 mmol, 3.00 equiv.) was added while stirring. A solution of trimethylsilyl trifluoromethanesulfonate (362 µL, 445 mg, 2.00 mmol, 2.00 equiv.) in 1 mL of dry MeCN was added dropwise over 5 min. The reaction mixture was stirred at –40 C for 1 hour, and then stirred at 25 °C for 16 hours. The solvent was removed under reduced pressure by rotary evaporation and the residue was dissolved in DCM (10 mL). The resulting solution was washed with aqueous NaBF$_4$ solution (2 × 10 mL, 10% w/w), and with water (10 mL). The organic phase was dried over Na$_2$SO$_4$, filtered, and the solvent was removed under reduced pressure. The residue was purified by chromatography on silica gel eluting with DCM/MeOH (50:1 to 20:1, v/v) to afford **15-BF$_4$** (241 mg, 59%) as a yellow solid.

R*f* = 0.20 (DCM/MeOH, 10:1, v/v (UV))

NMR Spectroscopy:

^{1}H NMR (300 MHz, CDCl$_3$, 25 °C, δ): 8.52 (d, *J* = 7.8 Hz, 2H), 7.93 (d, *J* = 2.5 Hz, 1H), 7.87 – 7.78 (m, 4H), 7.77 – 7.71 (m, 2H), 7.61 (dd, *J* = 8.8, 2.9 Hz, 1H), 6.79 (d, *J* = 9.1 Hz, 1H), 3.87 (s, 3H).

^{13}C {^{1}H} NMR (76 MHz, CDCl$_3$, 25 °C, δ): 166.9, 147.6, 138.4, 136.4, 135.2, 135.1, 130.6, 130.4, 118.5, 113.9, 112.8, 54.7.

^{19}**F NMR** (282 MHz, $CDCl_3$, 25 °C, δ): –150.5 (bs), –150.6 (bs).

HRMS ESI (m/z) calc'd for $C_{18}H_{14}O_1N_1S_2^+$ $[M–BF_4]^+$, 324.0511; found, 324.0510. Deviation: 0.5 ppm.

Flurbiprofen methyl ester-derived thianthrenium salt 16-BF$_4$

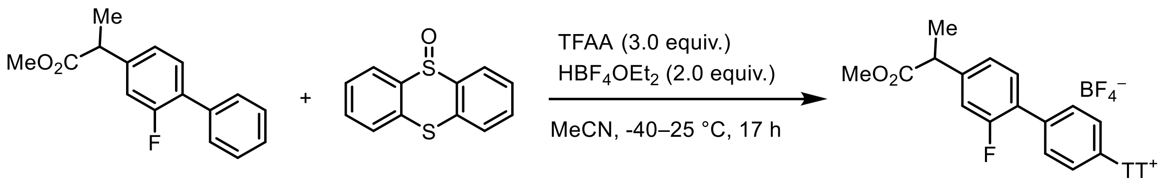

16-BF$_4$, 80%

The compound **16-BF$_4$** was prepared according to the modified versions of the reported procedures and the NMR spectra are in good accordance with reported data.[8] Under an ambient atmosphere, a 25 mL round-bottom flask equipped with a stir bar was charged with thianthrene-S-oxide (232 mg, 1.00 mmol, 1.00 equiv.), flurbiprofen methyl ester (258 mg, 1.00 mmol, 1.00 equiv.) and MeCN (10 mL, *c* = 0.1 M). After cooling to −40 C, TFAA (417 μL, 630 mg, 3.00 mmol, 3.00 equiv.) and then $HBF_4·OEt_2$ (272 μL, 324 mg, 2.00 mmol, 2.00 equiv.) were subsequently added to the vial while stirring the reaction mixture. The reaction mixture was stirred at −40 C for 1 hour, and then stirred at 25 °C for 16 hours. The solvent was removed under reduced pressure by rotary evaporation and the residue was dissolved in DCM (10 mL). The resulting solution was washed with saturated aqueous $NaHCO_3$ solution (10 mL) and aqueous $NaBF_4$ solution (2 × 10 mL, 10% w/w). The organic phase was dried over Na_2SO_4, filtered, and the solvent was removed under reduced pressure. The residue was purified by chromatography on silica gel eluting with DCM/MeOH (50:1 to 10:1, v/v) to afford **16-BF$_4$** (450 mg, 80%) as a colorless solid.

R*f* = 0.35 (DCM/MeOH, 15:1, v/v (UV))

NMR Spectroscopy:

^{1}**H NMR** (300 MHz, DMSO-d$_6$, 25 °C, δ): 8.62 (d, *J* = 8.2 Hz, 2H), 8.10 (d, *J* = 7.3 Hz, 2H), 7.95 (t, *J* = 7.3 Hz, 2H), 7.88 (t, *J* = 7.1 Hz, 2H), 7.73 (d, *J* = 8.3 Hz, 2H), 7.47 (t, *J* = 8.0 Hz, 1H), 7.32 (d, *J* = 8.8 Hz, 2H), 7.24 (t, *J* = 8.6 Hz, 2H), 3.91 (q, *J* = 6.9 Hz, 1H), 3.60 (s, 3H), 1.41 (q, *J* = 6.7 Hz, 3H).

^{13}**C {^{1}H} NMR** (76 MHz, DMSO-d$_6$, 25 °C, δ): 173.6, 158.8 (d, *J* = 245 Hz), 144.0 (d, *J* = 7.7 Hz), 139.0, 135.7, 135.5, 134.8, 130.9 (d, *J* = 3.2 Hz), 130.6 (d, *J* = 2.9 Hz), 130.3, 129.7, 128.4, 124.6 (d, *J* = 12.7 Hz), 124.3 (d, *J* = 3.1 Hz), 124.2, 119.1, 115.4 (d, *J* = 23.1 Hz), 52.0, 43.8, 18.2.

^{19}**F NMR** (282 MHz, DMSO-d$_6$, 25 °C, δ): –117.9, –148.2, –148.3.

HRMS ESI (m/z) calc'd for $C_{28}H_{22}O_2F_1S_2^+$ $[M–BF_4]^+$, 473.1040; found, 473.1039. Deviation: 0.2 ppm.

Methyl-2-methoxybenzoate-derived thianthrenium salt 18-BF_4

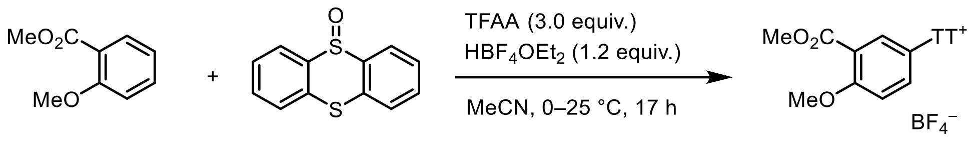

18-BF_4, 75%

The compound **18-BF_4** was prepared according to the modified versions of the reported procedures and the NMR spectra are in good accordance with reported data.[8] Under an ambient atmosphere, a 25 mL round-bottom flask equipped with a stir bar was charged with thianthrene-S-oxide (230 mg, 1.00 mmol, 1.00 equiv.), methyl-2-methoxybenzoate (166 mg, 1.00 mmol, 1.00 equiv.) and MeCN (10 mL, *c* = 0.1 M). After cooling to 0 C, TFAA (417 µL, 630 mg, 3.00 mmol, 3.00 equiv.) and then HBF_4·OEt_2 (163 µL, 194 mg, 1.20 mmol, 1.20 equiv.) were subsequently added to the vial while stirring the reaction mixture. The reaction mixture was stirred at 0 C for 1 hour, and then stirred at 25 °C for 16 hours. The solvent was removed under reduced pressure by rotary evaporation and the residue was dissolved in DCM (10 mL). The resulting solution was washed with saturated aqueous $NaHCO_3$ solution (10 mL) and aqueous $NaBF_4$ solution (2 × 10 mL, 10% w/w). The organic phase was dried over Na_2SO_4, filtered, and the solvent was removed under reduced pressure. The residue was purified by chromatography on silica gel eluting with DCM/MeOH (50:1 to 20:1, v/v) to afford **18-BF_4** (353 mg, 75%) as a colorless solid.

R*f* = 0.40 (DCM/MeOH, 15:1, v/v (UV))

NMR Spectroscopy:

^{1}H NMR (300 MHz, CD_2Cl_2, 25 °C, δ): 8.38 (d, *J* = 7.4 Hz, 2H), 7.90 (d, *J* = 7.9 Hz, 2H), 7.86 (t, *J* = 7.3 Hz, 2H), 7.79 (t, *J* = 7.6 Hz, 2H), 7.55 (d, *J* = 2.8 Hz, 1H), 7.41 (dd, *J* = 9.4, 2.9 Hz, 1H), 7.12 (d, *J* = 9.4 Hz, 1H), 3.90 (s, 3H), 3.79 (s, 3H).

^{13}C {^{1}H} NMR (76 MHz, CD_2Cl_2, 25 °C, δ): 164.6, 163.1, 136.9, 135.5, 134.8, 134.0, 131.8, 131.0, 130.7, 123.1, 119.1, 115.1, 113.4, 57.1, 52.9.

^{19}F NMR (282 MHz, CD_2Cl_2, 25 °C, δ): –151.4 (bs), –151.5 (bs).

HRMS ESI (m/z) calc'd for $C_{21}H_{17}O_3S_2^+$ $[M–BF_4]^+$, 381.0614; found, 381.0612. Deviation: 0.4 ppm.

Fenbufen methyl ester-derived thianthrenium salt 19-BF$_4$

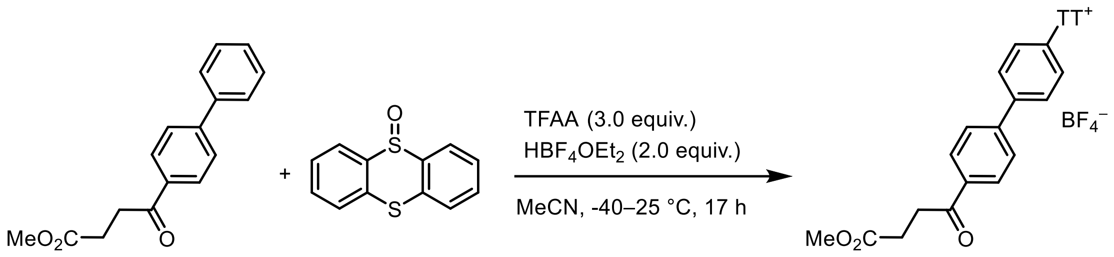

19-BF$_4$, 60%

The compound **19-BF$_4$** was prepared according to the modified versions of the reported procedures and the NMR spectra are in good accordance with reported data.[8] Under an ambient atmosphere, a 25 mL round-bottom flask equipped with a stir bar was charged with thianthrene-S-oxide (232 mg, 1.00 mmol, 1.00 equiv.), fenbufen methyl ester (268 mg, 1.00 mmol, 1.00 equiv.) and MeCN (10 mL, *c* = 0.1 M). After cooling to −40 C, TFAA (417 µL, 630 mg, 3.00 mmol, 3.00 equiv.) and then $HBF_4 \cdot OEt_2$ (272 µL,324 mg, 2.00 mmol, 2.00 equiv.) were subsequently added to the vial while stirring the reaction mixture. The reaction mixture was stirred at −40 C for 1 hour, and then stirred at 25 °C for 16 hours. The solvent was removed under reduced pressure by rotary evaporation and the residue was dissolved in DCM (10 mL). The resulting solution was washed with saturated aqueous $NaHCO_3$ solution (10 mL) and aqueous $NaBF_4$ solution (2 × 10 mL, 10% w/w). The organic phase was dried over Na_2SO_4, filtered, and the solvent was removed under reduced pressure. The residue was purified by chromatography on silica gel eluting with DCM/MeOH (50:1 to 20:1, v/v) to afford **19-BF$_4$** (340 mg, 60%) as a colorless solid.

R*f* = 0.30 (DCM/MeOH, 15:1, v/v (UV))

NMR Spectroscopy:

^{1}H NMR (300 MHz, $CDCl_3$, 25 °C, δ): 8.61 (d, *J* = 7.3 Hz, 2H), 8.00 (d, *J* = 8.7 Hz, 2H), 7.87 – 7.80 (m, 5H), 7.80 – 7.75 (m, 2H), 7.62 (d, *J* = 8.2 Hz, 2H), 7.54 (d, *J* = 8.2 Hz, 2H), 7.24 (d, *J* = 8.4 Hz, 2H), 3.69 (s, 3H), 3.30 (t, *J* = 7.0 Hz, 2H), 2.76 (t, *J* = 6.7 Hz, 2H).

^{13}C {^{1}H} NMR (76 MHz, $CDCl_3$, 25 °C, δ): 197.8, 173.6, 144.7, 142.9, 136.9, 136.8, 136.0, 135.3, 130.8, 130.6, 129.6, 129.2, 129.0, 127.8, 123.7, 119.1, 52.2, 33.8, 29.3.

^{19}F NMR (282 MHz, $CDCl_3$, 25 °C, δ): –150.6 (bs), –150.7 (bs).

HRMS ESI (m/z) calc'd for $C_{29}H_{23}O_3S_2^+$ $[M–BF_4]^+$, 483.1083; found, 483.1082. Deviation: 0.2 ppm.

1,2-Dimethoxybenzene-derived thianthrenium salt 20-BF$_4$

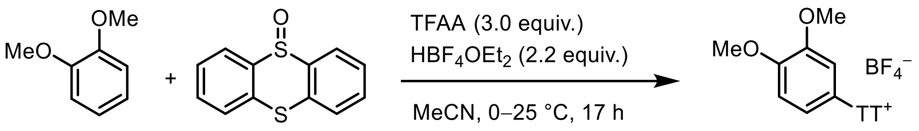

20-BF$_4$, 75%

The compound **20-BF$_4$** was prepared according to the modified versions of the reported procedures and the NMR spectra are in good accordance with reported data.[7] Under an ambient atmosphere, a 25 mL round-bottom flask equipped with a stir bar was charged with thianthrene-S-oxide (232 mg, 1.00 mmol, 1.00 equiv.), 1,2-dimethoxybenzene (128 µL, 138 mg, 1.00 mmol, 1.00 equiv.) and MeCN (10 mL, *c* = 0.1 M). After cooling to 0 C, TFAA (417 µL, 630 mg, 3.00 mmol, 3.00 equiv.) and then $HBF_4·OEt_2$ (299 µL, 356 mg, 2.20 mmol, 2.20 equiv.) were subsequently added to the vial while stirring the reaction mixture. The reaction mixture was stirred at 0 C for 1 hour, and then stirred at 25 °C for 16 hours. The solvent was removed under reduced pressure by rotary evaporation and the residue was dissolved in DCM (10 mL). The resulting solution was washed with saturated aqueous $NaHCO_3$ solution (10 mL) and aqueous $NaBF_4$ solution (2 × 10 mL, 10% w/w). The organic phase was dried over Na_2SO_4, filtered, and the solvent was removed under reduced pressure. The residue was purified by chromatography on silica gel eluting with DCM/MeOH (50:1 to 20:1, v/v) to afford **20-BF$_4$** (331 mg, 75%) as a grey solid.

R*f* = 0.40 (DCM/MeOH, 15:1, v/v (UV))

NMR Spectroscopy:

^{1}H NMR (300 MHz, DMSO-d$_6$, 25 °C, δ): 8.48 (dd, *J* = 7.8, 1.1 Hz, 2H), 8.06 (dd, *J* = 7.8, 1.3 Hz, 2H), 7.90 (td, *J* = 7.3, 1.2 Hz, 2H), 7.82 (td, *J* = 7.9, 1.3 Hz, 2H), 7.13 (d, *J* = 8.3 Hz, 1H), 7.01 (d, *J* = 2.4 Hz, 1H), 6.86 (dd, *J* = 8.9, 2.4 Hz, 1H), 3.78 (s, 3H), 3.64 (s, 3H).

^{13}C {^{1}H} NMR (76 MHz, DMSO-d$_6$, 25 °C, δ): 152.7, 150.0, 134.7, 134.6, 134.5, 130.0, 129.6, 122.7, 119.9, 114.3, 112.7, 111.3, 56.0, 56.0.

^{19}F NMR (282 MHz, DMSO-d$_6$, 25 °C, δ): –148.2 (bs), –148.3 (bs).

HRMS ESI (m/z) calc'd for $C_{20}H_{17}O_2S_2^+$ $[M–BF_4]^+$, 353.0665; found, 353.0662. Deviation: 0.7 ppm.

Fluorobenzene-derived thianthrenium salt 25-BF$_4$

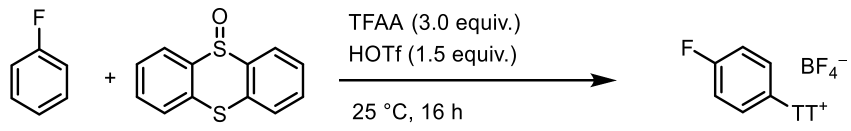

25-BF$_4$, 54%

The compound **25-BF$_4$** was prepared according to the modified versions of the reported procedures and the NMR spectra are in good accordance with reported data.[3] Under an ambient atmosphere, a 10 mL round-

bottom flask equipped with a stir bar was charged with thianthrene-S-oxide (232 mg, 1.00 mmol, 1.00 equiv.), and fluorobenzene (1.00 mL, 1.08 mg, 11.2 mmol, 11.2 equiv.). Trifluoromethanesulfonic acid (133 µL, 225 mg, 1.50 mmol, 1.50 equiv.) was added, and then TFAA (417 µL, 630 mg, 3.00 mmol, 3.00 equiv.) was subsequently added to the vial while stirring the reaction mixture. The reaction mixture was stirred at 25 °C for 16 hours. The solvent was removed under reduced pressure by rotary evaporation and the residue was dissolved in DCM (10 mL). The resulting solution was washed with saturated aqueous $NaHCO_3$ solution (10 mL) and aqueous $NaBF_4$ solution (2 × 10 mL, 10% w/w). The organic phase was dried over Na_2SO_4, filtered, and the solvent was removed under reduced pressure. The residue was purified by chromatography on silica gel eluting with DCM/MeOH (50:1 to 20:1, v/v) to afford **25-BF$_4$** (216 mg, 54%) as a grey solid.

R*f* = 0.40 (DCM/MeOH, 15:1, v/v (UV))

NMR Spectroscopy:

^{1}H NMR (300 MHz, DMSO-d$_6$, 25 °C, δ): 8.28 (d, *J* = 7.6 Hz, 2H), 8.07 (d, *J* = 7.9 Hz, 2H), 7.93 (t, *J* = 7.2 Hz, 2H), 7.86 (t, *J* = 7.4 Hz, 2H), 7.48 – 7.38 (m, 2H), 7.34 – 7.27 (m, 2H).

^{13}C {^{1}H} NMR (76 MHz, DMSO-d$_6$, 25 °C, δ): 164.2 (d, *J* = 253.0 Hz), 135.6, 135.3, 134.8, 131.1 (d, *J* = 9.7 Hz), 130.3, 129.6, 120.4 (d, *J* = 3.2 Hz), 119.5, 117.8 (d, *J* = 24.5 Hz).

^{19}F NMR (282 MHz, DMSO-d$_6$, 25 °C, δ): –106.3, –148.2 (bs), –148.3 (bs).

HRMS ESI (m/z) calc'd for $C_{18}H_{12}F_1S_2^+$ $[M–BF_4]^+$, 331.0359; found, 331.0357. Deviation: 0.7 ppm.

3,3-Diphenylpropan-1-ol -derived thianthrenium salt 26-BF$_4$

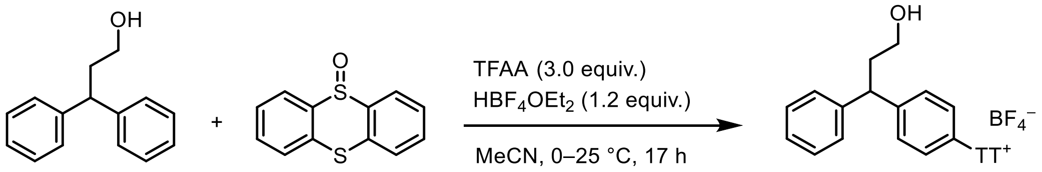

26-BF$_4$, 24%

The compound **26-BF$_4$** was prepared according to the modified versions of the reported procedures[3] and the NMR spectra are in good accordance with reported data.[3] Under an ambient atmosphere, a 25 mL round-bottom flask equipped with a stir bar was charged with thianthrene-S-oxide (230 mg, 1.00 mmol, 1.0 equiv.), 3,3-diphenylpropan-1-ol (212 mg, 1.0 mmol, 1.0 equiv.) and MeCN (10 mL, *c* = 0.1 M). After cooling to 0 °C, TFAA (417 µL, 630 mg, 1.20 mmol, 1.20 equiv.) was subsequently added to the vial while stirring the reaction mixture, followed by $HBF_4{\cdot}OEt_2$ (163 µL, 194 mg, 1.20 mmol, 1.20 equiv.). The reaction mixture was stirred at 0 °C for 1 hour, and then stirred at 25 °C for 16 hours. The reaction mixture was washed with saturated aqueous $NaHCO_3$ solution (10 mL) and aqueous $NaBF_4$ solution (2 × 10 mL, 10% w/w). The organic phase was dried over Na_2SO_4, filtered, and the solvent was removed under reduced pressure. The residue was purified by chromatography on silica gel eluting with DCM/MeOH (50:1 to 20:1, v/v) to afford **26-BF$_4$** (124 mg, 24%) as a colorless solid.

R*f* = 0.30 (DCM/MeOH, 15:1, v/v (UV))

NMR Spectroscopy:

^{1}H NMR (300 MHz, CD_3CN, 25 °C, δ): 8.32 (d, *J* = 8.0 Hz, 2H), 7.94 (d, *J* = 7.8 Hz, 2H), 7.87 (t, *J* = 8.3 Hz, 2H), 7.79 (t, *J* = 8.0 Hz, 2H), 7.41 (t, *J* = 8.3 Hz, 2H), 7.31 – 7.14 (m, 5H), 7.05 (d, *J* = 8.5 Hz, 2H), 4.18 (d, *J* = 7.8 Hz, 1H), 3.33 (d, *J* = 6.2 Hz, 2H), 2.67 (s, 1H), 2.19 – 2.09 (m, 2H).

^{13}C {^{1}H} NMR (76 MHz, CD_3CN, 25 °C, δ): 152.2, 144.3, 137.4, 136.0, 135.8, 131.6, 130.9, 130.8, 129.7, 129.1, 128.7, 127.6, 122.0, 119.44, 119.42, 60.0, 47.6, 38.3.

^{19}F NMR (282 MHz, CD_3CN, 25 °C, δ): –151.6 (bs), –151.7 (bs).

HRMS ESI (m/z) calc'd for $C_{27}H_{23}O_1S_2^+$ $[M–BF_4]^+$, 427.1185; found, 427.1183. Deviation: 0.4 ppm.

Ethylbenzene-derived thianthrenium salt 28-BF_4

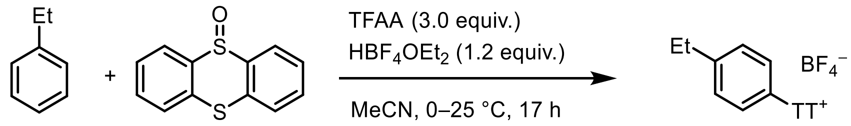

28-BF_4, 70%

The compound **28-BF_4** was prepared according to the modified versions of the reported procedures and the NMR spectra are in good accordance with reported data.[2, 13] Under an ambient atmosphere, a 25 mL round-bottom flask equipped with a stir bar was charged with thianthrene-S-oxide (232 mg, 1.00 mmol, 1.00 equiv.), ethylbenzene (123 µL, 106 mg, 1.00 mmol, 1.00 equiv.) and MeCN (10 mL, *c* = 0.1 M). After cooling to 0 C, TFAA (417 µL, 630 mg, 3.00 mmol, 3.00 equiv.) and then $HBF_4{\cdot}OEt_2$ (163 µL, 194 mg, 1.20 mmol, 1.20 equiv.) were subsequently added to the vial while stirring the reaction mixture. The reaction mixture was stirred at 0 C for 1 hour, and then stirred at 25 °C for 16 hours. The solvent was removed under reduced pressure by rotary evaporation and the residue was dissolved in DCM (10 mL). The resulting solution was washed with saturated aqueous $NaHCO_3$ solution (10 mL) and aqueous $NaBF_4$ solution (2 × 10 mL, 10% w/w). The organic phase was dried over Na_2SO_4, filtered, and the solvent was removed under reduced pressure. The residue was purified by chromatography on silica gel eluting with DCM/MeOH (50:1 to 20:1, v/v) to afford **28-BF_4** (284 mg, 70%) as a colorless solid.

R*f* = 0.30 (DCM/MeOH, 15:1, v/v (UV))

NMR Spectroscopy:

^{1}H NMR (300 MHz, $CDCl_3$, 25 °C, δ): 8.56 (d, *J* = 7.3 Hz, 2H), 7.89 – 7.74 (m, 6H), 7,27 (d, *J* = 8.4 Hz, 2H), 7.11 (d, *J* = 8.5 Hz, 2H), 2.65 (q, *J* = 7.6 Hz, 2H), 1.19 (t, *J* = 7.5 Hz, 3H).

^{13}C {^{1}H} NMR (76 MHz, $CDCl_3$, 25 °C, δ): 150.5, 136.6, 135.4, 135.0, 130.42, 130.39, 130.33, 128.2, 120.7, 119.1, 28.7, 14.9.

^{19}F NMR (282 MHz, $CDCl_3$, 25 °C, δ): –151.2 (bs), –151.3 (bs).

HRMS ESI (m/z) calc'd for $C_{20}H_{17}S_2^+$ [M–BF_4]$^+$, 321.0766; found, 321.0764. Deviation: 0.8 ppm.

Synthesis of aryl triflates

Estrone-derived aryl triflate 25a

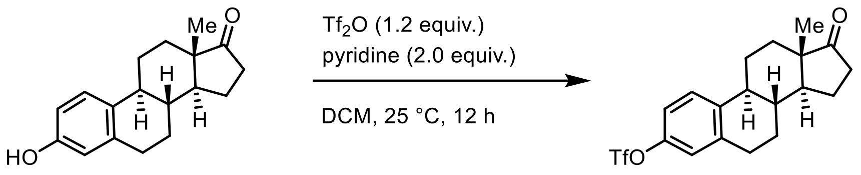

25a, 76%

The compound **26a** was prepared according to the modified versions of the reported procedures and the NMR spectra are in good accordance with reported data.[4] Under nitrogen atmosphere, a 25 mL round-bottom flask equipped with a stir bar was charged with estrone (406 mg, 1.50 mmol, 1.0 equiv.), pyridine (242 μL, 237 mg, 3.00 mmol, 2.00 equiv.) and DCM (8 mL, *c* = 0.2 M). After cooling to 0 °C, trifluoromethanesulfonic anhydride (302 μL, 508 mg, 1.80 mmol, 1.20 equiv.) was added dropwise to the flask while stirring the reaction mixture. The mixture was stirred at 25 °C for 12 h. Then the reaction was quenched with water (5 mL), the organic layer was collected and washed with brine (10 mL). The resulting organic phase was dried over Na_2SO_4, filtered, and the solvent was removed under reduced pressure. The residue was purified by chromatography on silica gel eluting with hexanes/ethyl acetate (50:1 to 3:1, v/v) to afford **25a** (460 mg, 76%) as a colorless solid.

R*f* = 0.50 (hexanes/ethyl acetate, 3:1, v/v (UV))

NMR Spectroscopy:

^{1}H NMR (300 MHz, $CDCl_3$, 25 °C, δ): 7.33 (d, *J* = 8.6 Hz, 1H), 7.06 – 6.97 (m, 2H), 2.97 – 2.91 (m, 2H), 2.57 – 2.47 (m, 1H), 2.46 – 2.36 (m, 1H), 2.35 – 2.23 (m, 1H), 2.23 – 2.01 (m, 3H), 2.01 – 1.93 (m, 1H), 1.74 – 1.40 (m, 6H), 0.91 (s, 3H).

^{13}C {^{1}H} NMR (76 MHz, $CDCl_3$, 25 °C, δ): 220.5, 147.7, 140.4, 139.4, 127.3, 121.4, 118.6 (q, *J* = 320.9 Hz), 118.4, 50.5, 48.0, 44.2, 37.9, 35.9, 31.6, 29.5, 26.2, 25.8, 21.7, 13.9.

^{19}F NMR (282 MHz, $CDCl_3$, 25 °C, δ): –73.0.

HRMS GC-EI (m/z) calc'd for $C_{19}H_{21}O_4S_1F_3^+$ [M]$^+$, 402.1107; found, 402.1111. Deviation: –1.1 ppm.

4-(3-Oxobutyl)phenyl trifluoromethanesulfonate 26a

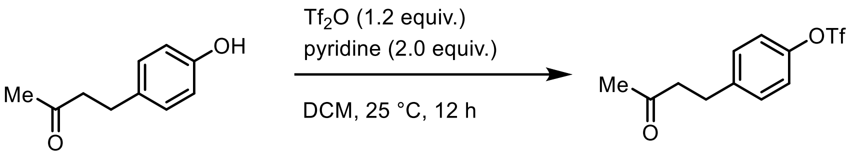

26a, 81%

The compound **26a** was prepared according to the modified versions of the reported procedures and the NMR spectra are in good accordance with reported data.[5] Under nitrogen atmosphere, a 10 mL round-bottom flask equipped with a stir bar was charged with 4-(4-hydroxyphenyl)-2-butanone (164 mg, 1.00 mmol, 1.00 equiv.), pyridine (162 µL, 158 mg, 2.00 mmol, 2.00 equiv.) and DCM (5 mL, *c* = 0.2 M). After cooling to 0 °C, trifluoromethanesulfonic anhydride (202 µL, 339 mg, 1.20 mmol, 1.20 equiv.) was added dropwise to the flask while stirring the reaction mixture. The mixture was stirred at 25 °C for 12 h. Then the reaction was quenched with water (5 mL), the organic layer was collected and washed with brine (10 mL). The resulting organic phase was dried over Na_2SO_4, filtered, and the solvent was removed under reduced pressure. The residue was purified by chromatography on silica gel eluting with hexanes/ethyl acetate (50:1 to 3:1, v/v) to afford **26a** (241 mg, 81%) as a colorless oil.

R_f = 0.40 (hexanes/ethyl acetate, 5:1, v/v (UV))

NMR Spectroscopy:

^{1}H NMR (300 MHz, $CDCl_3$, 25 °C, δ): 7.28 – 7.23 (m, 2H), 7.20 – 7.14 (m, 2H), 2.91 (t, *J* = 7.0 Hz, 2H), 2.76 (t, *J* = 7.6 Hz, 2H), 2.15 (s, 3H).

^{13}C {^{1}H} NMR (76 MHz, $CDCl_3$, 25 °C, δ): 207.2, 148.1, 141.9, 130.3, 121.4, 118.8 (q, *J* = 322.7 Hz), 44.8, 30.2, 29.0.

^{19}F NMR (282 MHz, $CDCl_3$, 25 °C, δ): –72.9.

HRMS GC-EI (m/z) calc'd for $C_{11}H_{11}O_4S_1F_3^+$ $[M]^+$, 296.0325; found, 296.0328. Deviation: –1.2 ppm.

MECHANISTIC STUDIES

NMR Monitoring

Competitive borylation of arylthianthrenium salt and aryl bromide

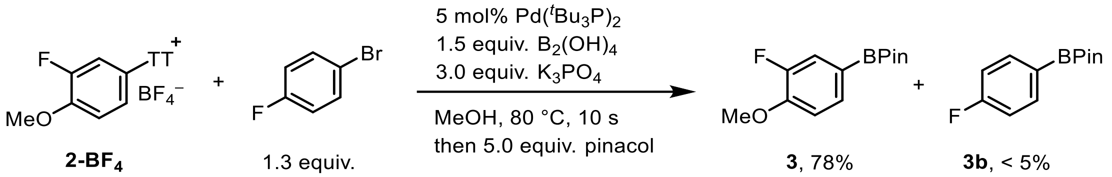

Under an ambient atmosphere, to a 4-mL borosilicate vial equipped with a stir bar was added 2-fluoroanisole-derived thianthrenium salt **2-BF$_4$** (21.4 mg, 50.0 μmol, 1.00 equiv.), $B_2(OH)_4$ (6.7 mg, 75 μmol, 1.5 equiv.), K_3PO_4 (31.8 mg, 0.150 mmol, 3.00 equiv.), *p*-bromofluorobenzene (8.0 μL, 11 mg, 65 μmol, 1.3 equiv.), followed by addition of MeOH (0.5 mL). After all the materials were fully dissolved, Pd(^{t}Bu$_3$P)$_2$ (1.3 mg, 2.5 μmol, 5.0 mol%) was added. The vial was then capped with a septum cap. Then, the reaction mixture was stirred vigorously at 80 °C for 10 s on a heating block. The reaction mixture was cooled down using an ice bath. Then, the reaction mixture was quenched with MeCN (0.2 mL), followed by addition of pinacol (29.5 mg, 0.25 mmol, 5.00 equiv.). After that, 2-fluorotoluene was added as an internal standard. An aliquot (ca. 0.1 mL) of the mixture was taken and diluted with $CDCl_3$ (0.5 mL), and pinacol (5.9 mg, 50 μmol, 1.0 equiv.) was additionally added into the mixture. The yield of products were determined by ^{19}F NMR integration of the aromatic C-F of products relative to that of peak of 2-fluorotoluene at –118.6 ppm (aromatic C–F, 1H).

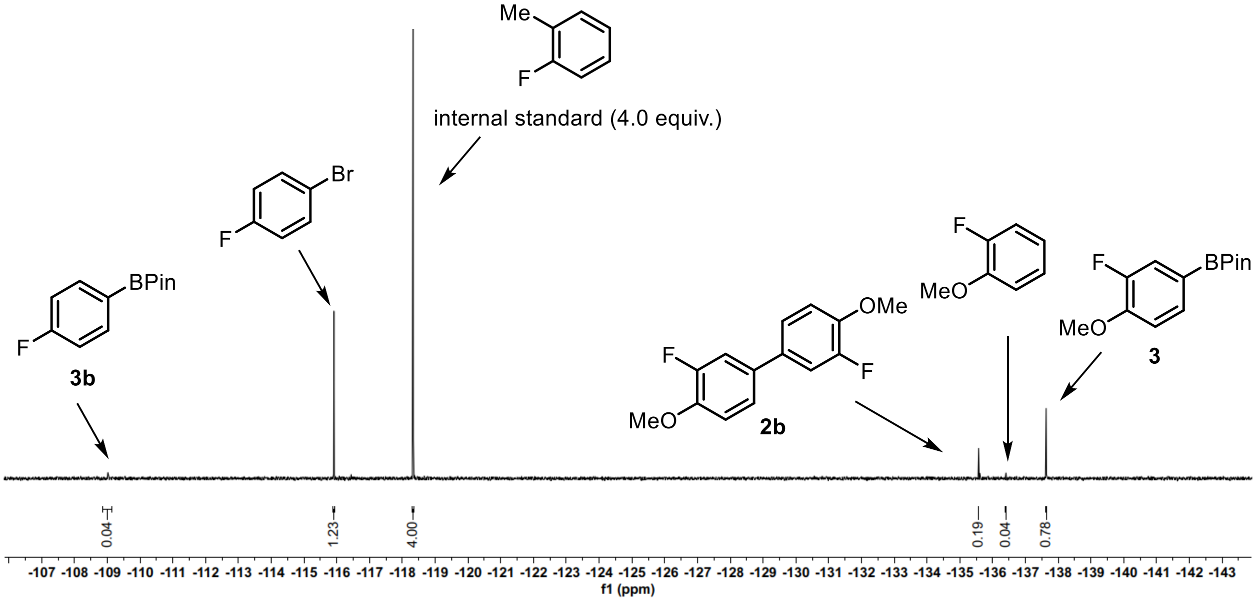

Figure S3. The ^{19}F NMR spectrum in $CDCl_3$

Competitive borylation of arylthianthrenium salt and aryl iodide

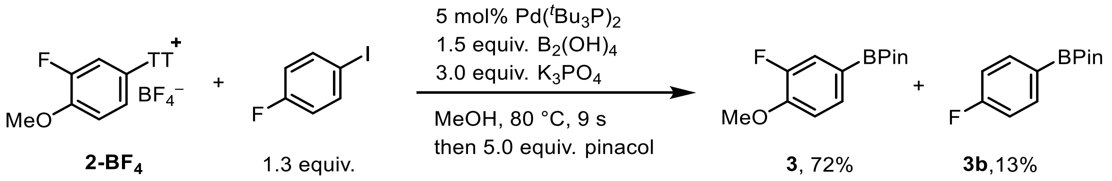

Under an ambient atmosphere, to a 4-mL borosilicate vial equipped with a stir bar was added 2-fluoroanisole-derived thianthrenium salt **2-BF$_4$** (21.4 mg, 50.0 µmol, 1.00 equiv.), $B_2(OH)_4$ (6.7 mg, 75 µmol, 1.5 equiv.), K_3PO_4 (31.8 mg, 0.150 mmol, 3.00 equiv.), *p*-iodofluorobenzene (14.4 mg, 65 µmol, 1.3 equiv.), followed by addition of MeOH (0.5 mL). After all the materials were fully dissolved, Pd(^{t}Bu$_3$P)$_2$ (1.3 mg, 2.5 µmol, 5.0 mol%) was added. The vial was then capped with a septum cap. Then, the reaction mixture was stirred vigorously at 80 °C for 10 s on a heating block. The reaction mixture was cooled down using an ice bath. Then, the reaction mixture was quenched with MeCN (0.2 mL), followed by addition of pinacol (29.5 mg, 0.25 mmol, 5.00 equiv.). After that, 2-fluorotoluene was added as an internal standard. An aliquot (ca. 0.1 mL) of the mixture was taken and diluted with $CDCl_3$ (0.5 mL), and pinacol (5.9 mg, 50 µmol, 1.0 equiv.) was additionally added into the mixture. The yield of products were determined by ^{19}F NMR integration of the aromatic C-F of products relative to that of peak of 2-fluorotoluene at –118.6 ppm (aromatic C–F, 1H).

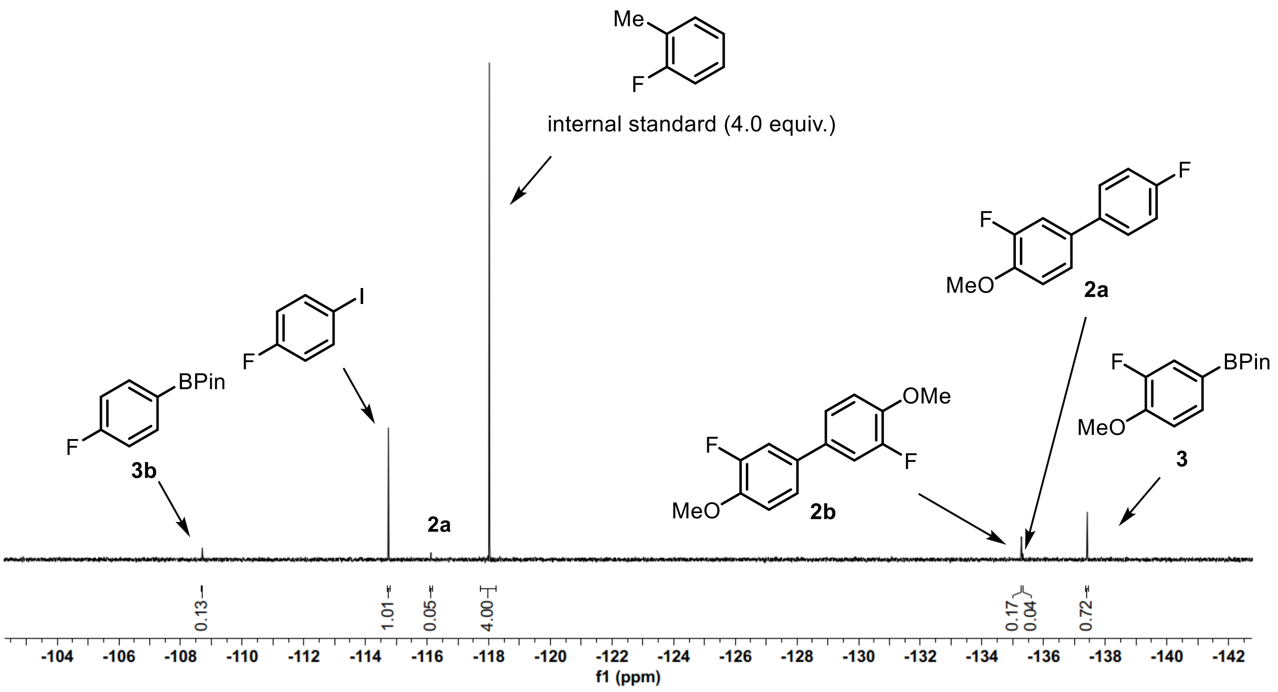

Figure S4. The ^{19}F NMR spectrum in $CDCl_3$

Competitive borylation of arylthianthrenium salt and aryl triflate

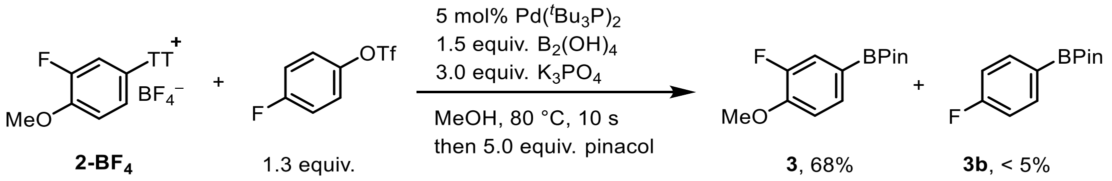

Under an ambient atmosphere, to a 4-mL borosilicate vial equipped with a stir bar was added 2-fluoroanisole-derived thianthrenium salt **2-BF₄** (21.4 mg, 50.0 μmol, 1.00 equiv.), $B_2(OH)_4$ (6.7 mg, 75 μmol, 1.5 equiv.), K_3PO_4 (31.8 mg, 0.150 mmol, 3.00 equiv.), 4-fluorophenyl triflate (15.9 mg, 0.130 mmol, 1.30 equiv.), followed by addition of MeOH (0.5 mL). After all the materials were fully dissolved, Pd(^{t}Bu$_3$P)$_2$ (1.3 mg, 2.5 μmol, 5.0 mol%) was added. The vial was then capped with a septum cap. Then, the reaction mixture was stirred vigorously at 80 °C for 10 s on a heating block. The reaction mixture was cooled down using an ice bath. Then, the reaction mixture was quenched with MeCN (0.2 mL), followed by addition of pinacol (29.5 mg, 0.25 mmol, 5.00 equiv.). After that, 2-fluorotoluene was added as an internal standard. An aliquot (ca. 0.1 mL) of the mixture was taken and diluted with $CDCl_3$ (0.5 mL), and pinacol (5.9 mg, 50 μmol, 1.0 equiv.) was additionally added into the mixture. The yield of products were determined by ^{19}F NMR integration of the aromatic C-F of products relative to that of peak of 2-fluorotoluene at –118.6 ppm (aromatic C–F, 1H).

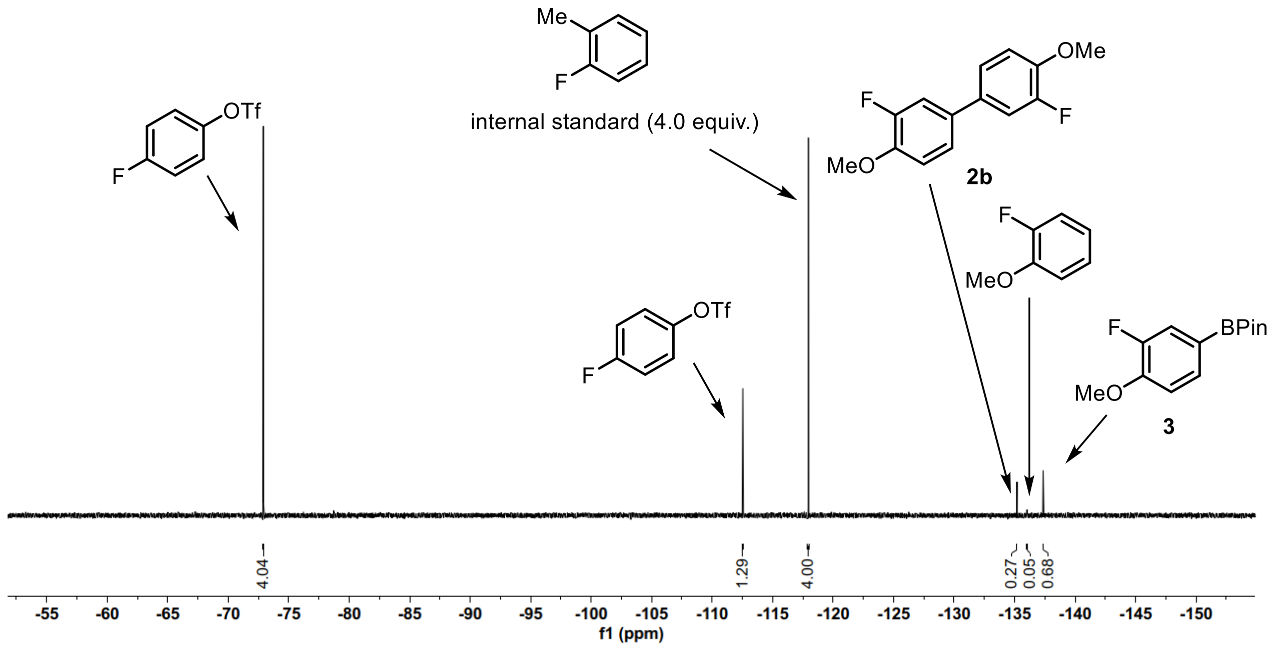

Figure S5. The ^{19}F NMR spectrum in $CDCl_3$

Reaction profile of Pd-catalyzed formal cross electrophile coupling

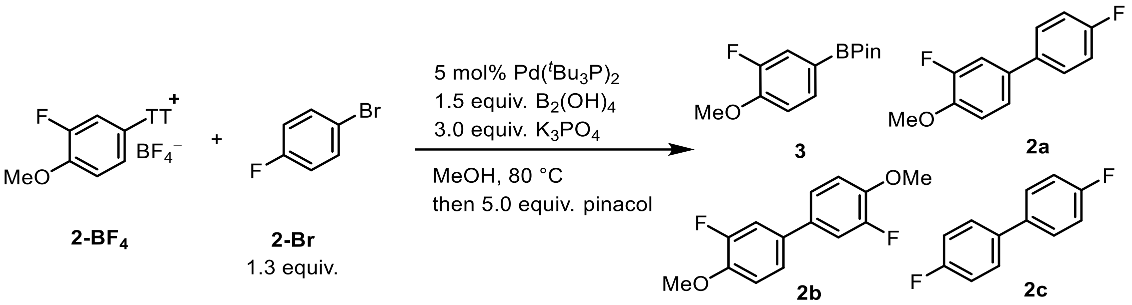

Under an ambient atmosphere, to a 4-mL borosilicate vial equipped with a stir bar was added 2-fluoroanisole-derived thianthrenium salt **2-BF4** (21.4 mg, 50.0 μmol, 1.00 equiv.), $B_2(OH)_4$ (6.7 mg, 75 μmol, 1.5 equiv.), K_3PO_4 (31.8 mg, 0.150 mmol, 3.00 equiv.), *p*-bromofluorobenzene (8.0 μL, 11 mg, 65 μmol, 1.3 equiv.), followed by addition of MeOH (0.5 mL, 0.1 M). After all the materials were fully dissolved, Pd(^{t}Bu$_3$P)$_2$ (1.3 mg, 2.5 μmol, 5.0 mol%) was added. The vial was then capped with a septum cap. Then, the reaction mixture was stirred vigorously at 80 °C for 10 s on a heating block. The reaction mixture was cooled down using an ice bath. Then, the reaction mixture was quenched with MeCN (0.2 mL), followed by addition of pinacol (29.5 mg, 0.25 mmol, 5.00 equiv.). After that, 2-fluorotoluene was added as an internal standard. An aliquot (ca. 0.1 mL) of the mixture was taken and diluted with $CDCl_3$ (0.5 mL), and pinacol (5.9 mg, 50 μmol, 1.0 equiv.) was additionally added into the mixture. The yield of products were determined by ^{19}F NMR integration of the aromatic C-F of products relative to that of peak of 2-fluorotoluene at –118.6 ppm (aromatic C–F, 1H).

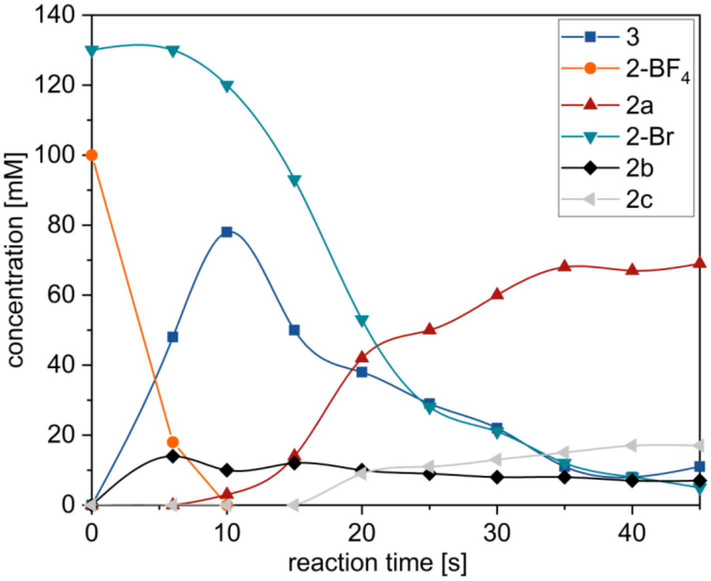

Figure S6. Reaction profile of formal CEC reaction.

Competition Kinetics

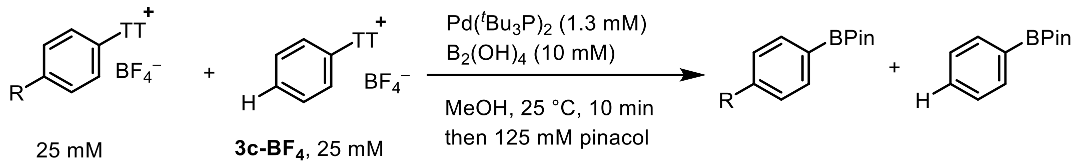

To a 4-mL borosilicate vial equipped with a stir bar was added para substituted benzene-derived arylthianthrenium salt (50.0 μmol, *c* = 25 mM, 1.00 equiv), benzene-derived arylthianthrenium salt **3c-BF$_4$** (20.5 mg, 50.0 μmol, *c* = 25 mM, 1.00 equiv.), $B_2(OH)_4$ (1.8 mg, 20 μmol, *c* = 10 mM, 0.40 equiv.). The vial was transferred into a N_2-filled glovebox, followed by addition of MeOH (2 mL). After all the materials were fully dissolved, Pd(^{t}Bu$_3$P)$_2$ (1.3 mg, 2.5 μmol, *c* = 1.3 mM, 5.0 mol%) was added. The vial was then capped with a septum cap, transferred out of the glovebox. The reaction mixture was stirred vigorously at 25 °C for 10 min. After that, the reaction vessel was opened to air, and the resulting mixture was concentrated by rotary evaporation. The residue was loaded on a silica column, eluting with ca. 100 mL of a hexanes/ethyl acetate mixture (1:1, v:v), the eluate was collected, and the solvent was removed. After that, CH_2Br_2 was added as an internal standard. An aliquot (ca. 0.1 mL) of the mixture was taken and diluted with MeOD-*d*$_4$ (0.5 mL), and the yield of products were determined by ^{1}H NMR integration relative to the internal standard.

Table S4. Reaction rate ratio for competition reactions (X: yield for borylation of para-substituted arylthianthrenium salt; H: yield for borylation of benzene-derived arylthianthrenium salt)

Entry	Para-substituent (X)	Yield ratio for X/H	Ratio of rates X/H
1	OMe	0.95	0.95
2	F	1.00	1.00
3	H	1.00	1.00
4	CO_2Me	1.12	1.12
5	NO_2	1.33	1.33

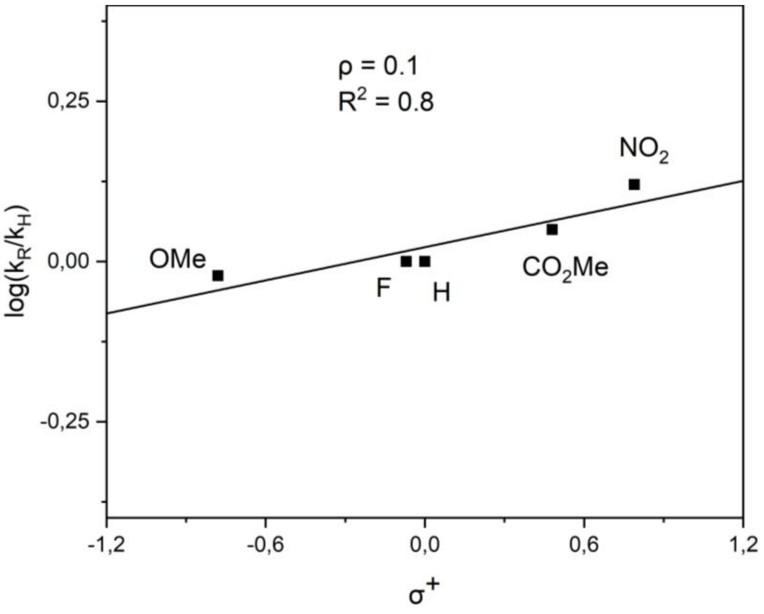

Figure S7. Hammett-plot for the borylation of arylthianthrenium salts, electrophilic substituent constants by *Brown* and *Okamoto*.[6]

Radical clock cyclization experiment

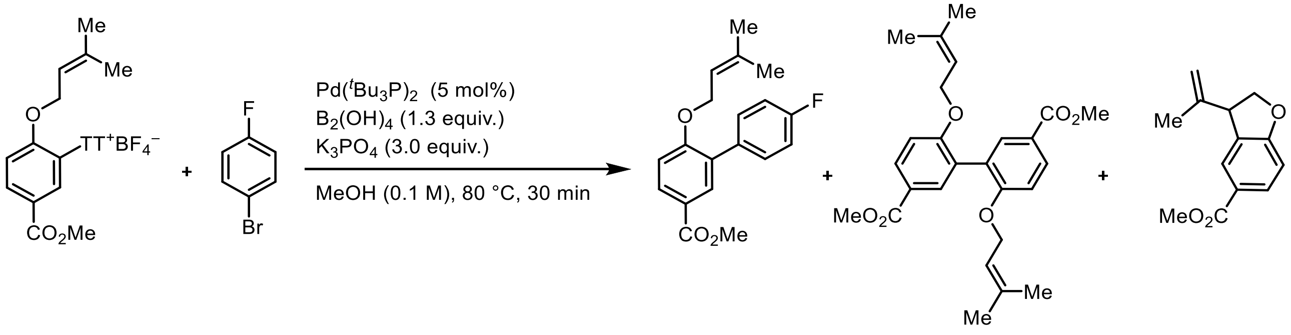

Under an inert atmosphere, thianthrenium salt **4-BF4** (26.1 mg, 50.0 μmol, 1.00 equiv.), $B_2(OH)_4$ (11.4 mg, 0.130 mmol, 1.30 equiv.), K_3PO_4 (31.4 mg, 0.150 mmol, 3.00 equiv.), *p*-bromofluorobenzene (8.0 μL, 11 mg, 65 μmol, 1.3 equiv.), Pd(^{t}Bu$_3$P)$_2$ (1.3 mg, 2.5 μmol, 5.0 mol%) were added to a 4-mL vial containing a magnetic stir bar, followed by MeOH (0.5 mL, *c* = 0.1 M). The vial was sealed with a septum cap, and the reaction mixture was stirred vigorously at 80 °C for 30 min. After the indicated time, the reaction mixture was allowed to cool to 23 °C. Then, the reaction vial was opened to air, and the resulting mixture was concentrated by rotary evaporation. The residue was purified by chromatography on silica gel eluting with hexanes/ethyl acetate (100:1 to 20:1, v/v) to afford 3.0 mg (19% yield) of product **4a** as a colorless oil and 6.8 mg (31% yield) of **4b** as a yellow oil. No cyclized product was observed.

Compound 4a R*f* = 0.2 (hexanes/EtOAc, 10:1, v/v (UV))

Compound 4b R*f* = 0.3 (hexanes/EtOAc, 10:1, v/v (UV))

NMR Spectroscopy for compound 4a:

^{1}H NMR (500 MHz, $CDCl_3$, 25 °C, δ): 8.01 – 7.97 (m, 2H), 7.54 – 7.47 (m, 2H), 7.12 – 7.07 (m, 2H), 6.98 (d, *J* = 9.0 Hz, 1H), 5.39 (tt, *J* = 6.9, 1.9 Hz, 1H), 4.60 (d, *J* = 7.1 Hz, 2H), 3.89 (s, 3H), 1.78 (s, 3H), 1.70 (s, 3H).

^{13}C NMR (126 MHz, $CDCl_3$, 25 °C, δ): 166.9, 159.6, 138.0, 133.7 (d, *J* = 3.7 Hz), 132.5, 131.3 (d, *J* = 8.9 Hz), 130.8, 129.9, 122.7, 119.4, 115.1, 114.9, 112.2, 65.8, 52.1, 25.9, 18.5.

^{19}F NMR (471 MHz, $CDCl_3$, 25 °C, δ): –115.4.

HRMS GC-EI (m/z) calc'd for $C_{19}H_{19}O_3F^+$ $[M]^+$, 314.1312; found, 314.1314. Deviation: –0.3 ppm.

NMR Spectroscopy for compound 4b:

^{1}H NMR (500 MHz, $CDCl_3$, 25 °C, δ): 8.01 (dd, *J* = 8.7, 2.2 Hz, 2H), 7.93 (d, *J* = 2.2 Hz, 2H), 6.94 (d, *J* = 8.7 Hz, 2H), 5.33 – 5.24 (m, 2H), 4.56 (d, *J* = 6.3 Hz, 4H), 3.87 (s, 6H), 1.71 (s, 6H), 1.67 (s, 6H).

^{13}C NMR (126 MHz, $CDCl_3$, 25 °C, δ): 167.1, 160.4, 137.1, 133.2, 131.1, 127.6, 122.2, 119.9, 111.7, 65.7, 52.0, 25.8, 18.4.

HRMS ESIpos (m/z) calc'd for $C_{26}H_{30}O_6Na^+$ $[M+Na]^+$, 461.1934; found, 461.1933. Deviation: +0.2 ppm.

SPECTROSCOPIC DATA

^{1}H NMR of 1

CDCl$_3$, 500 MHz, 25 °C

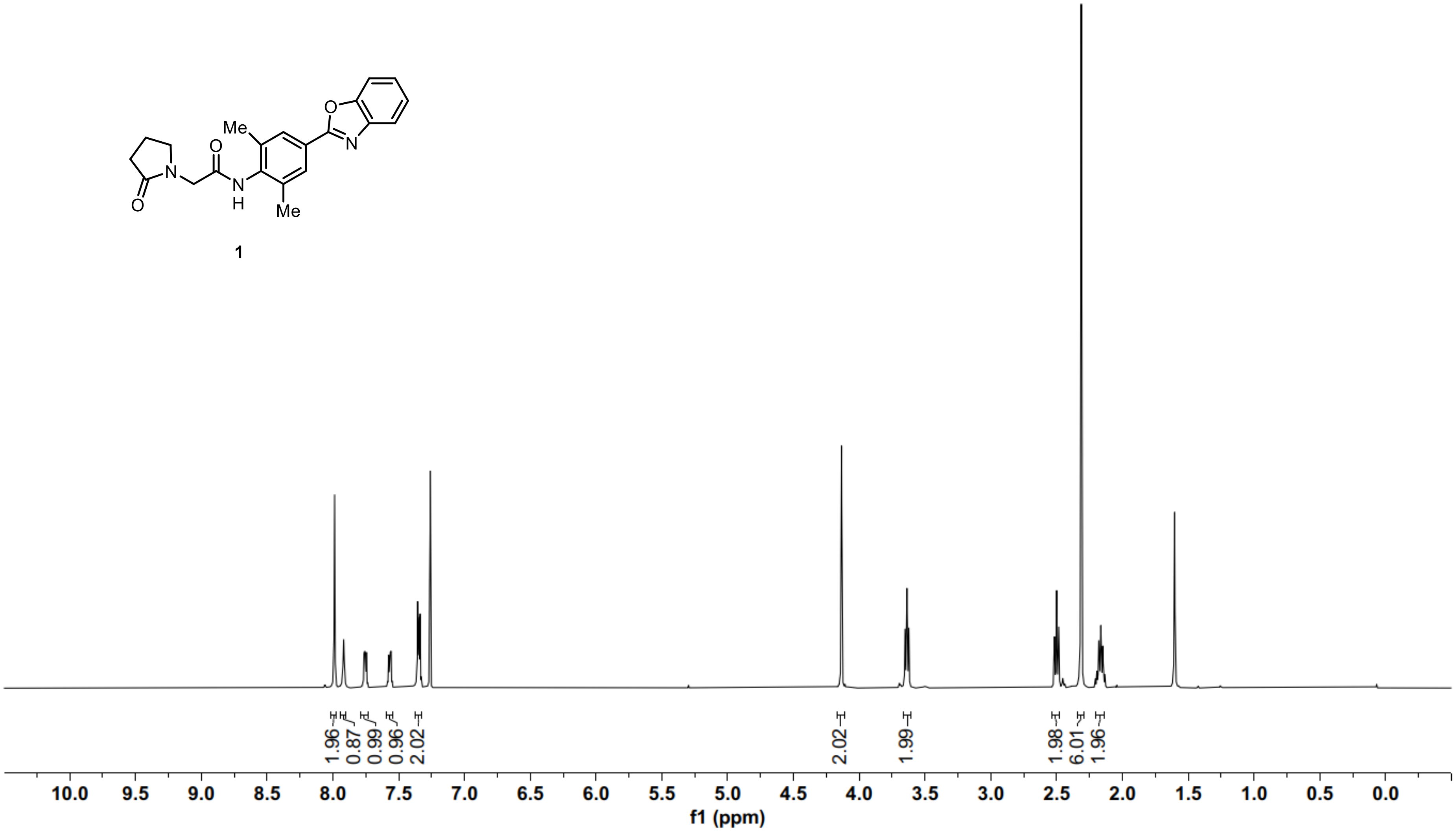

^{13}C NMR of 1

CDCl$_3$, 126 MHz, 25 °C

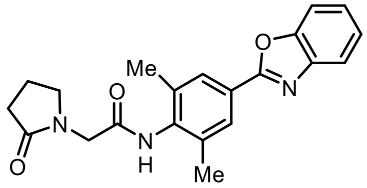

1

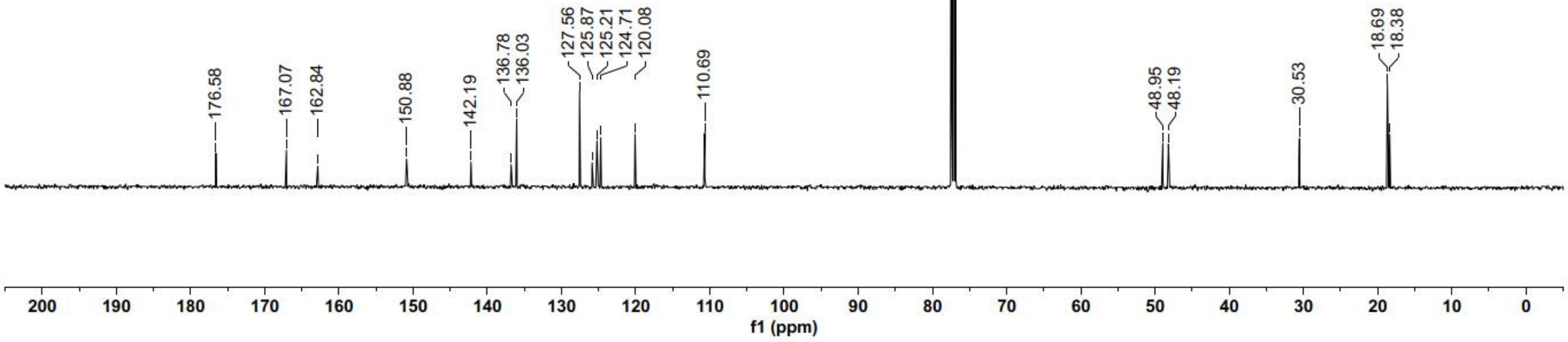

^{1}H NMR of 2

CDCl$_3$, 500 MHz, 25 °C

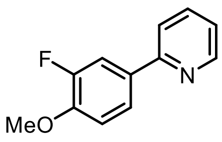

2

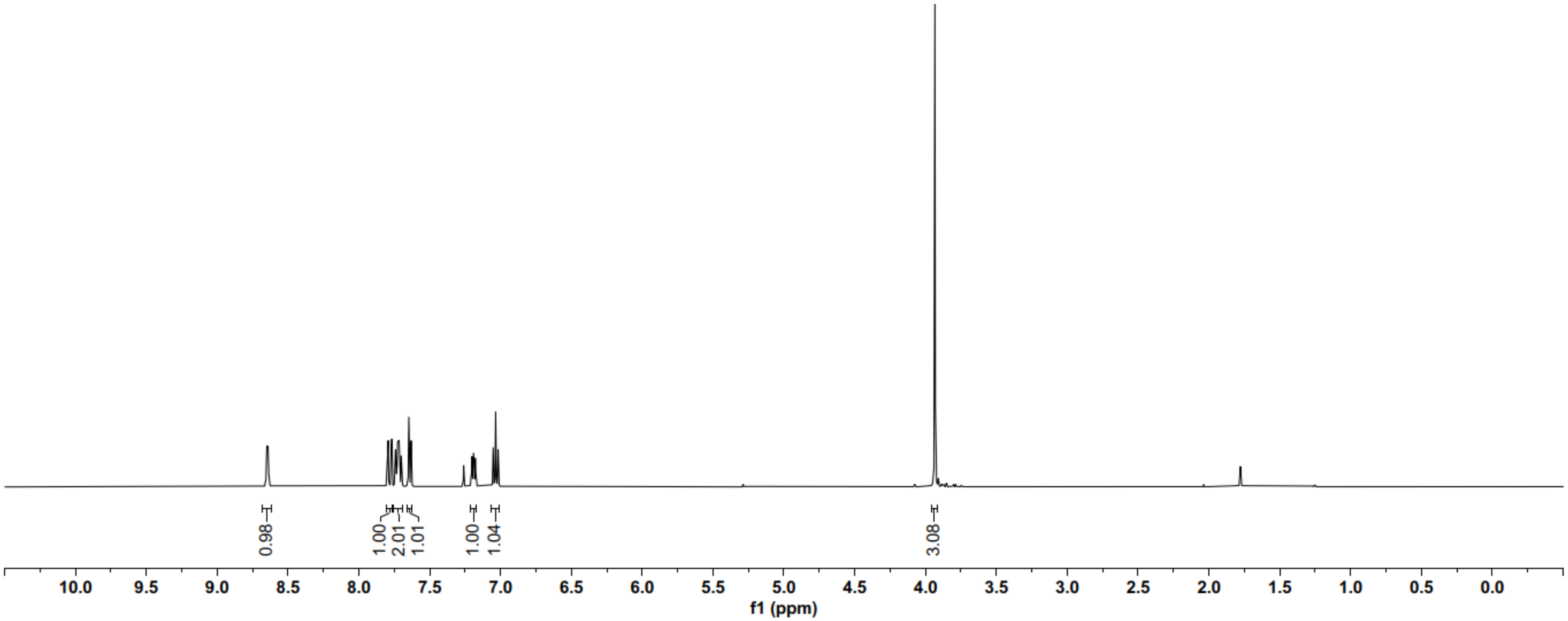

^{13}C NMR of 2

CDCl$_3$, 126 MHz, 25 °C

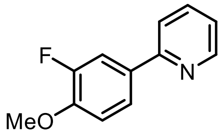

2

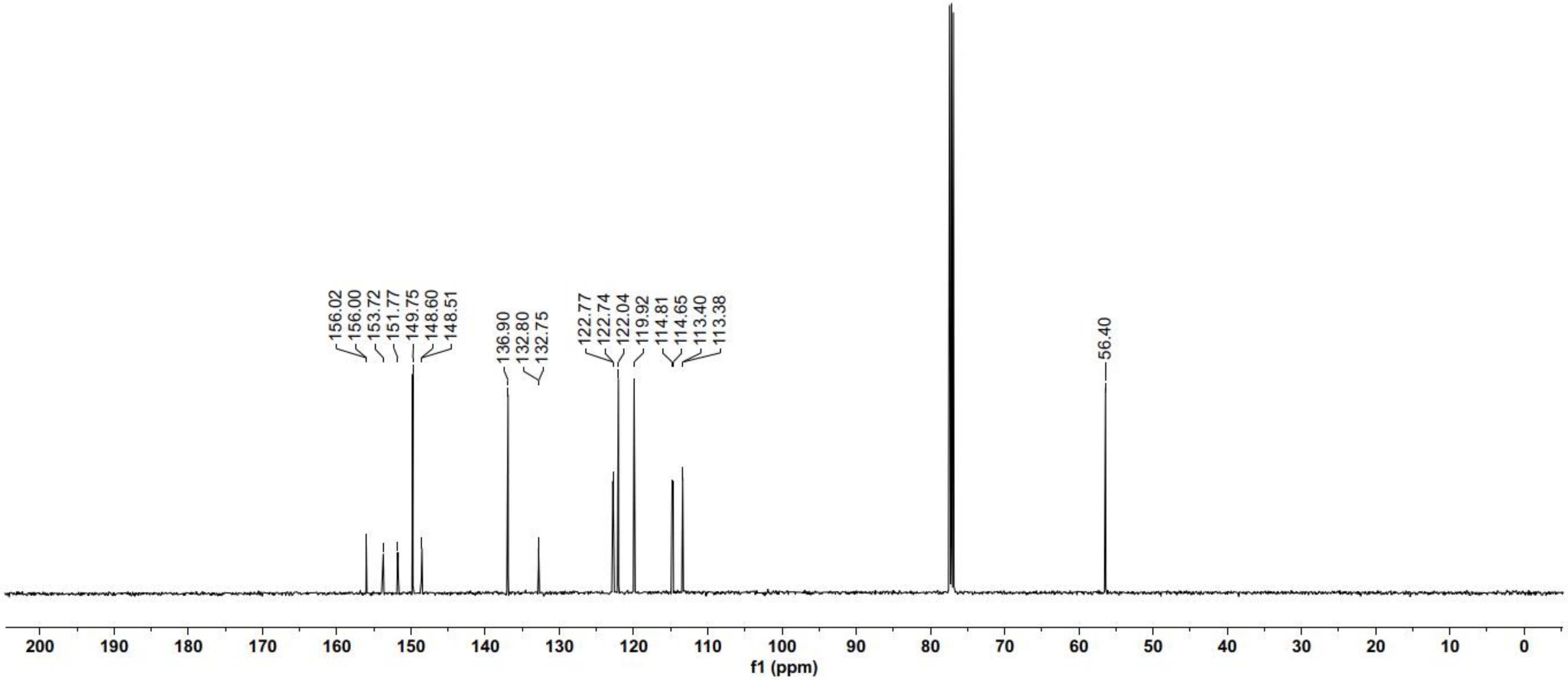

^{19}F NMR of 2

$CDCl_3$, 471 MHz, 25 °C

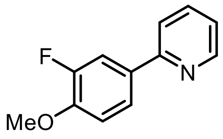

2

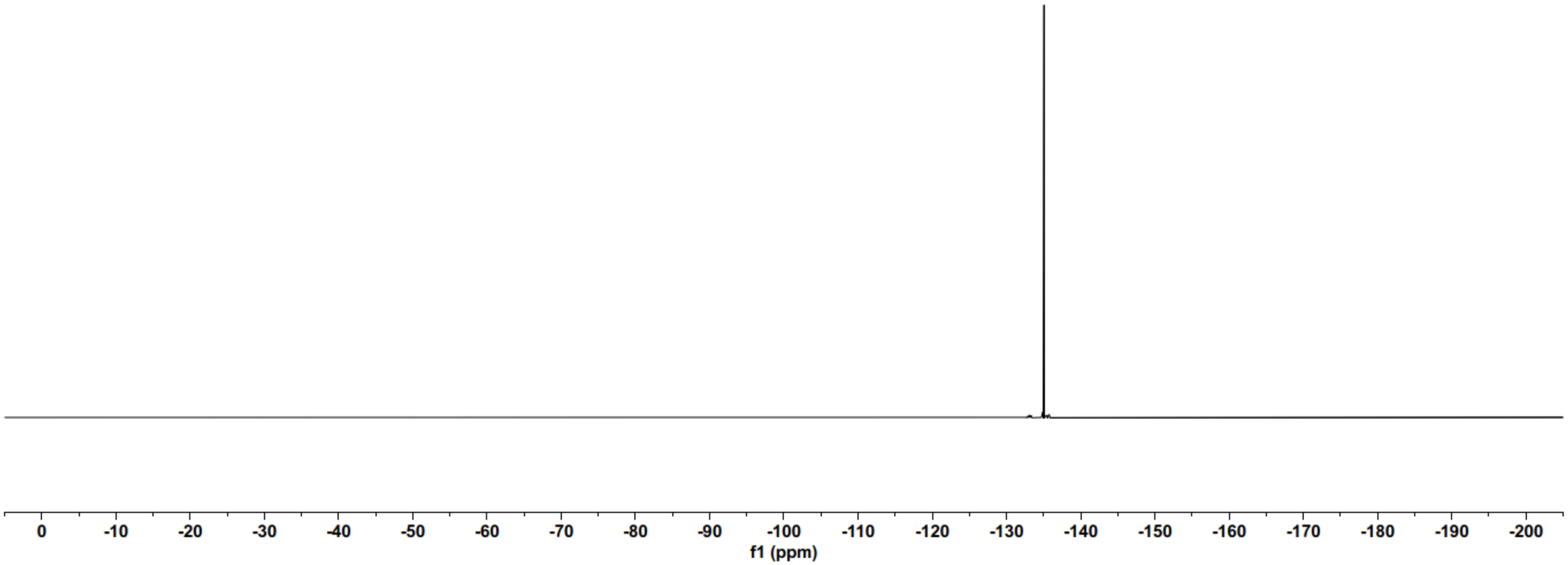

^{1}H NMR of 2a

CDCl$_3$, 500 MHz, 25 °C

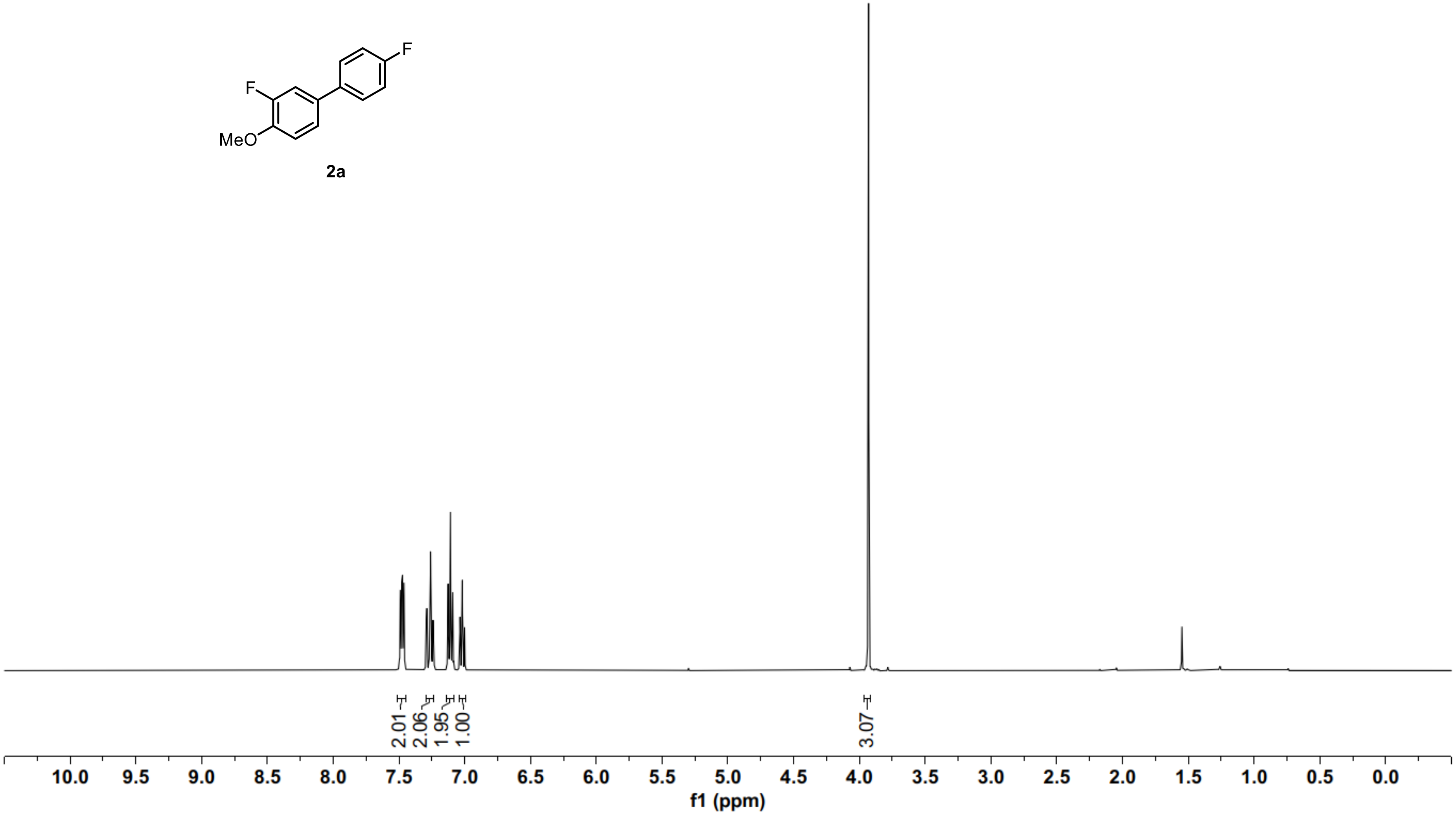

^{13}C NMR of 2a

CDCl₃, 126 MHz, 25 °C

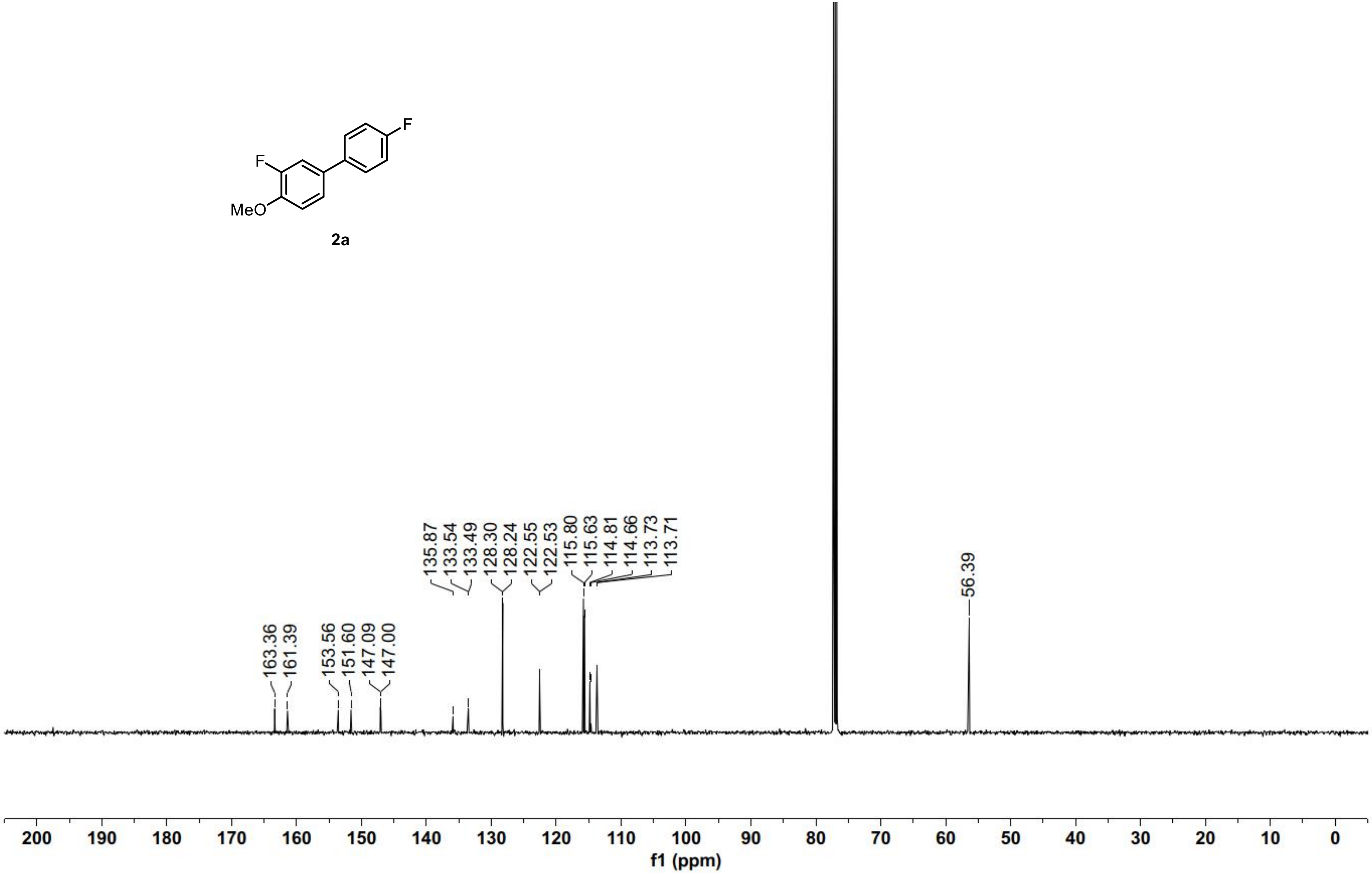

^{19}F NMR of 2a

$CDCl_3$, 471 MHz, 25 °C

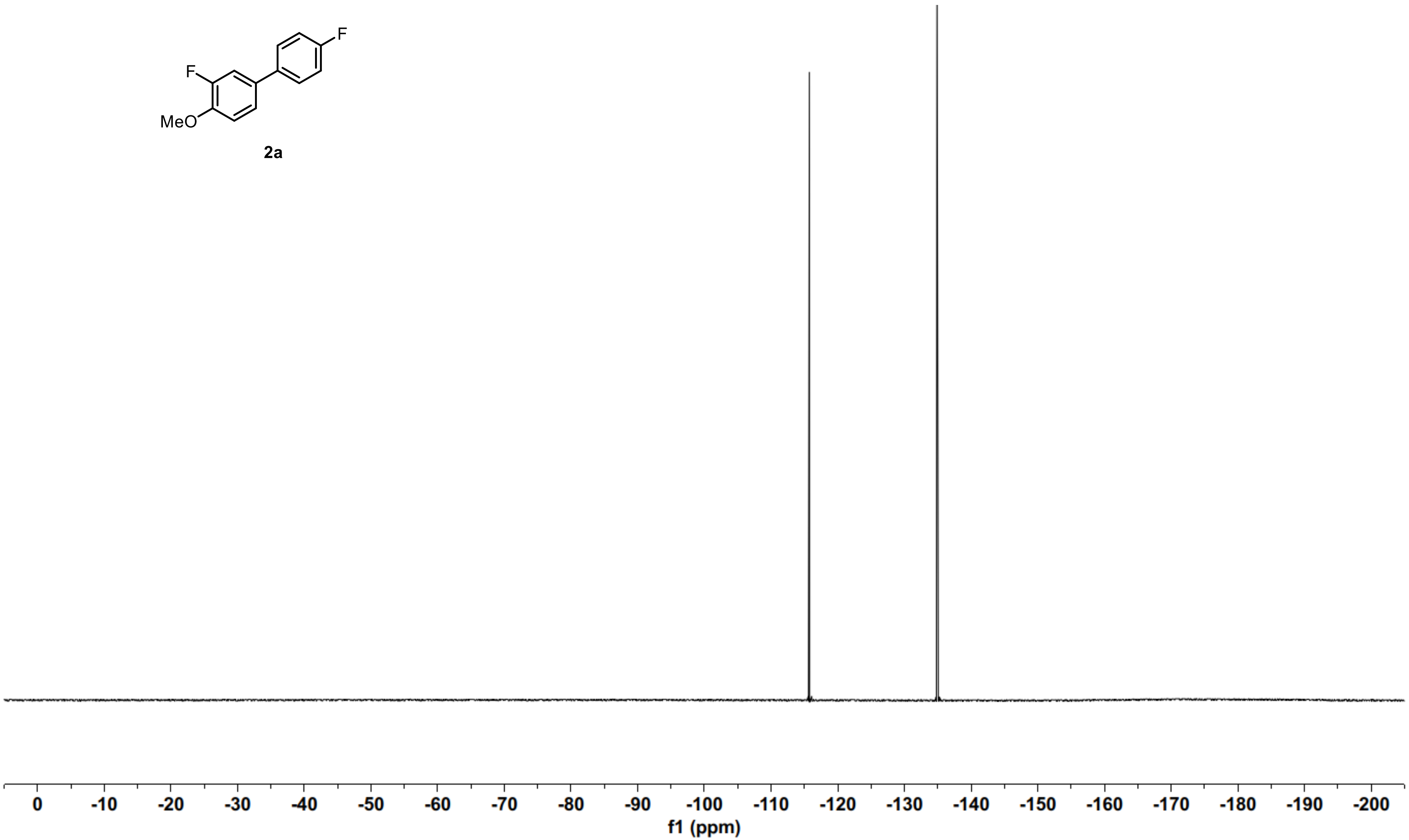

^{1}H NMR of 2b

CDCl$_3$, 500 MHz, 25 °C

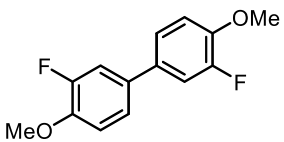

2b

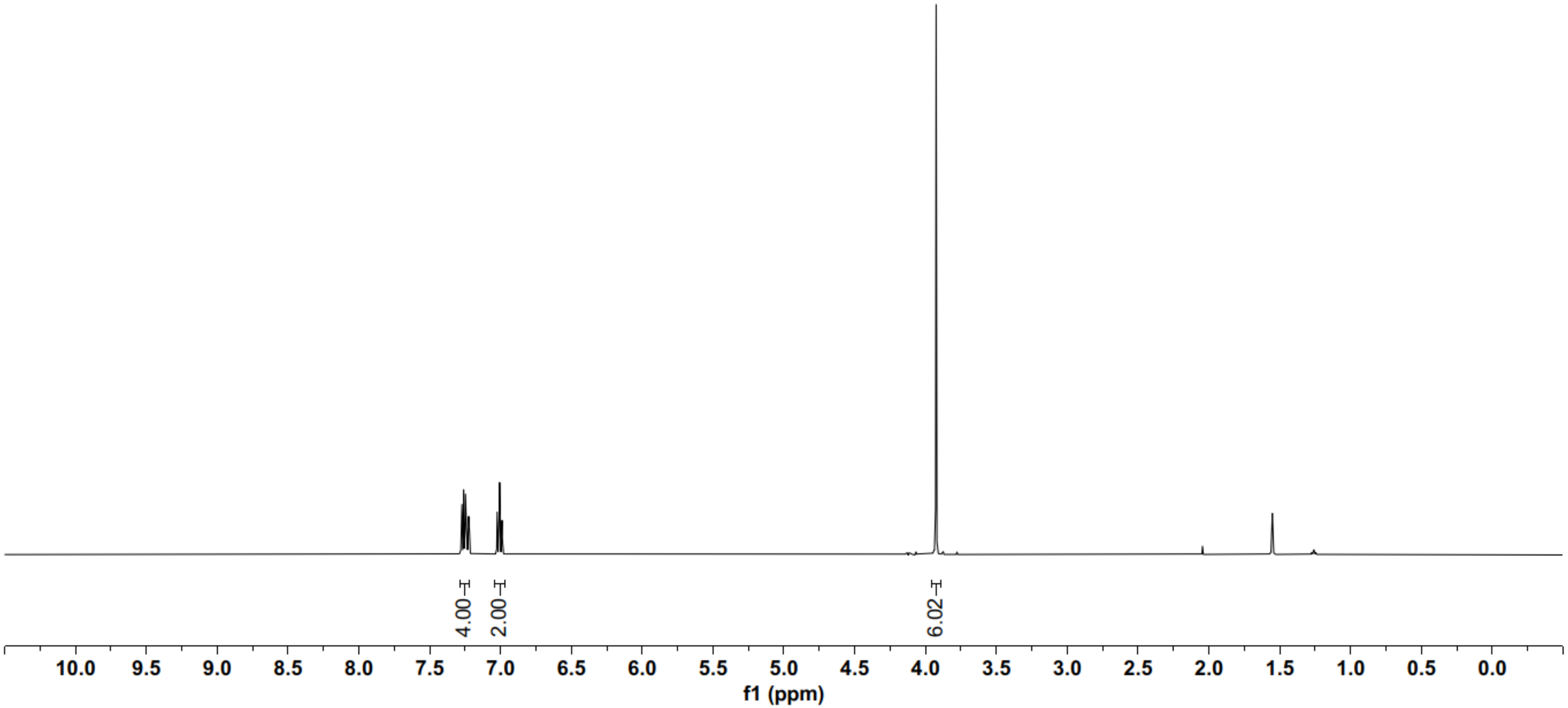

^{13}C NMR of 2b

CDCl$_3$, 126 MHz, 25 °C

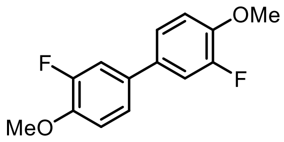

2b

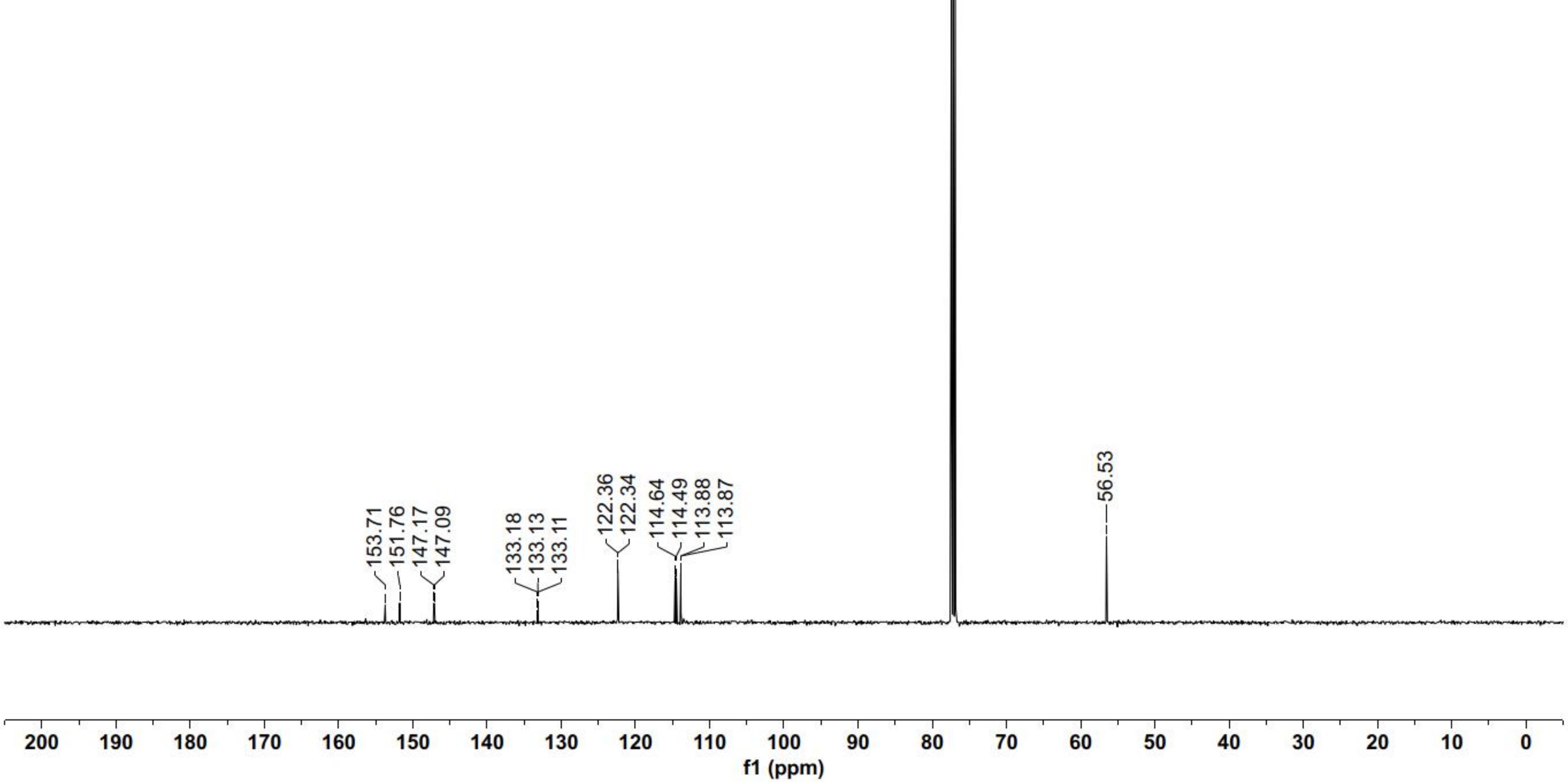

^{19}F NMR of 2b

$CDCl_3$, 471 MHz, 25 °C

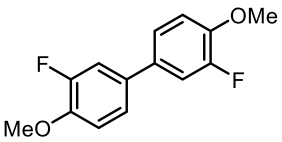

2b

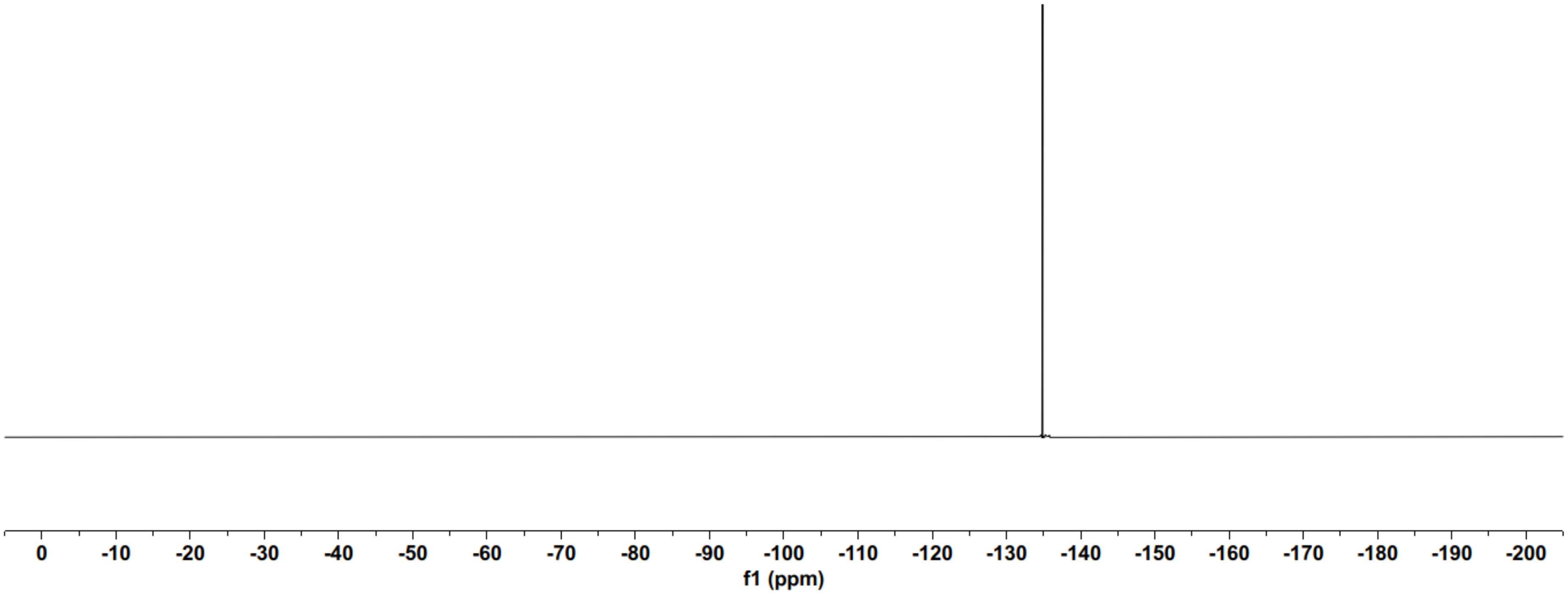

^{1}H NMR of 2d

CDCl$_3$, 500 MHz, 25 °C

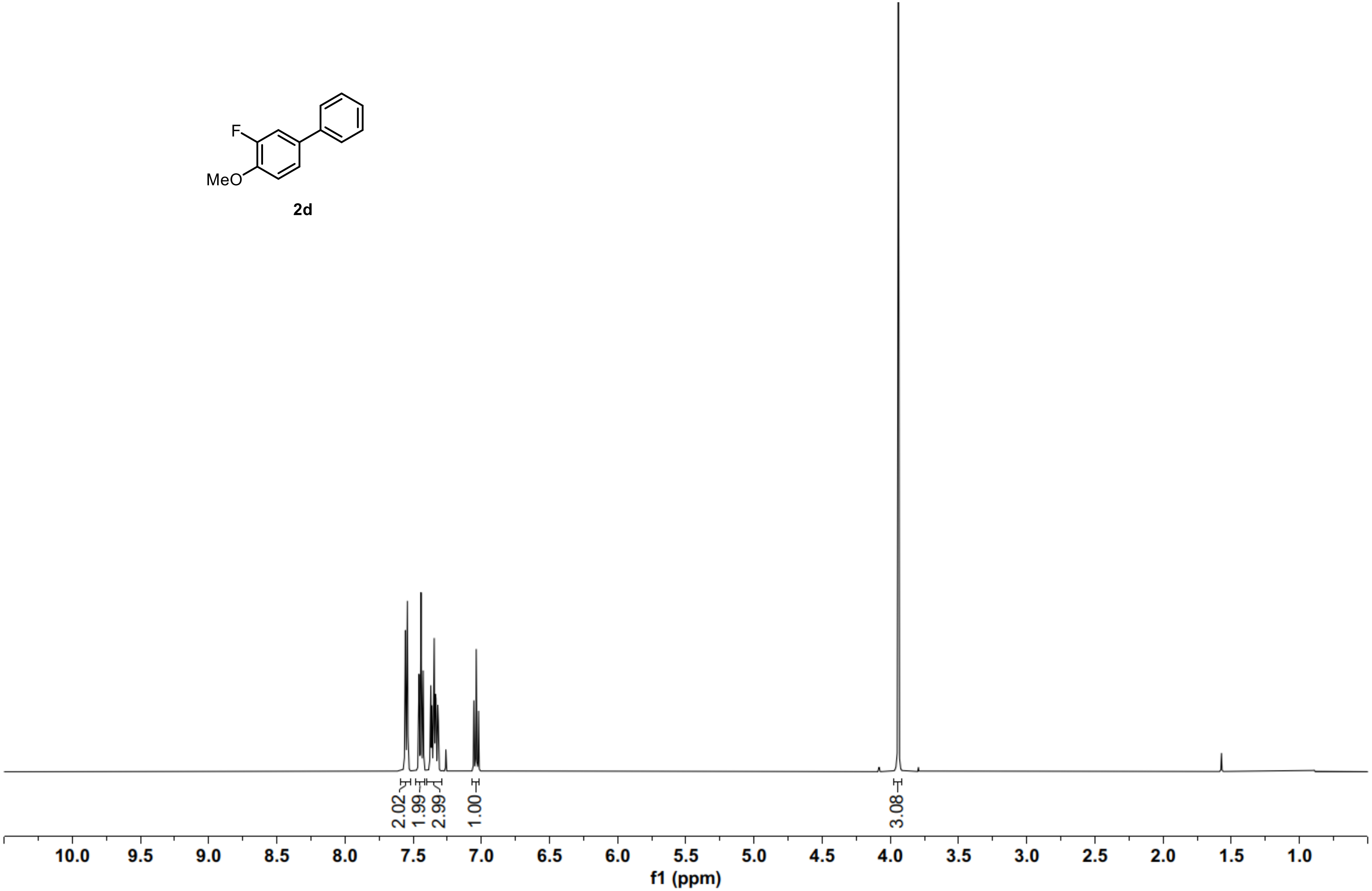

^{13}C NMR of 2d

$CDCl_3$, 126 MHz, 25 °C

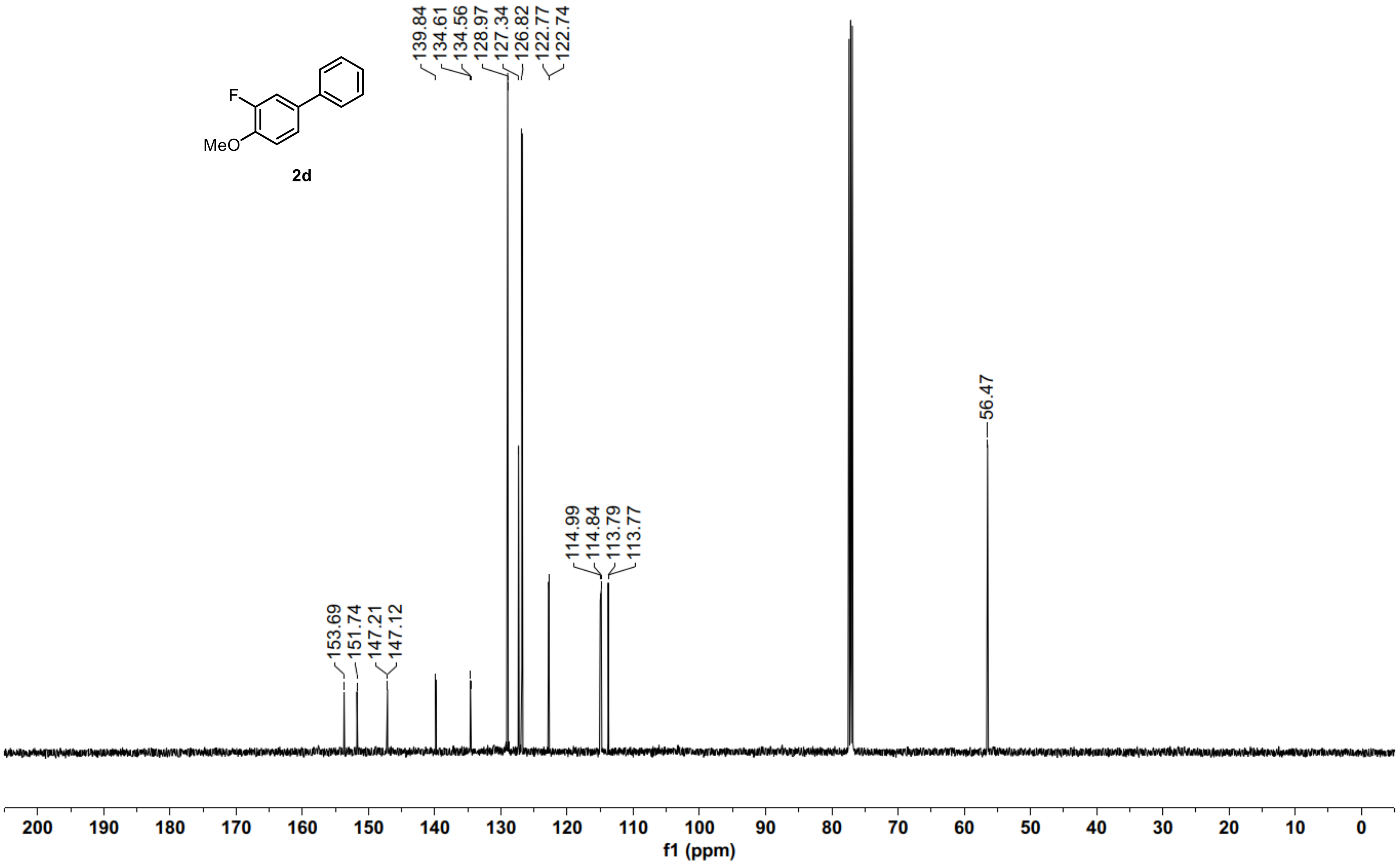

^{19}F NMR of 2d

CDCl$_3$, 471 MHz, 25 °C

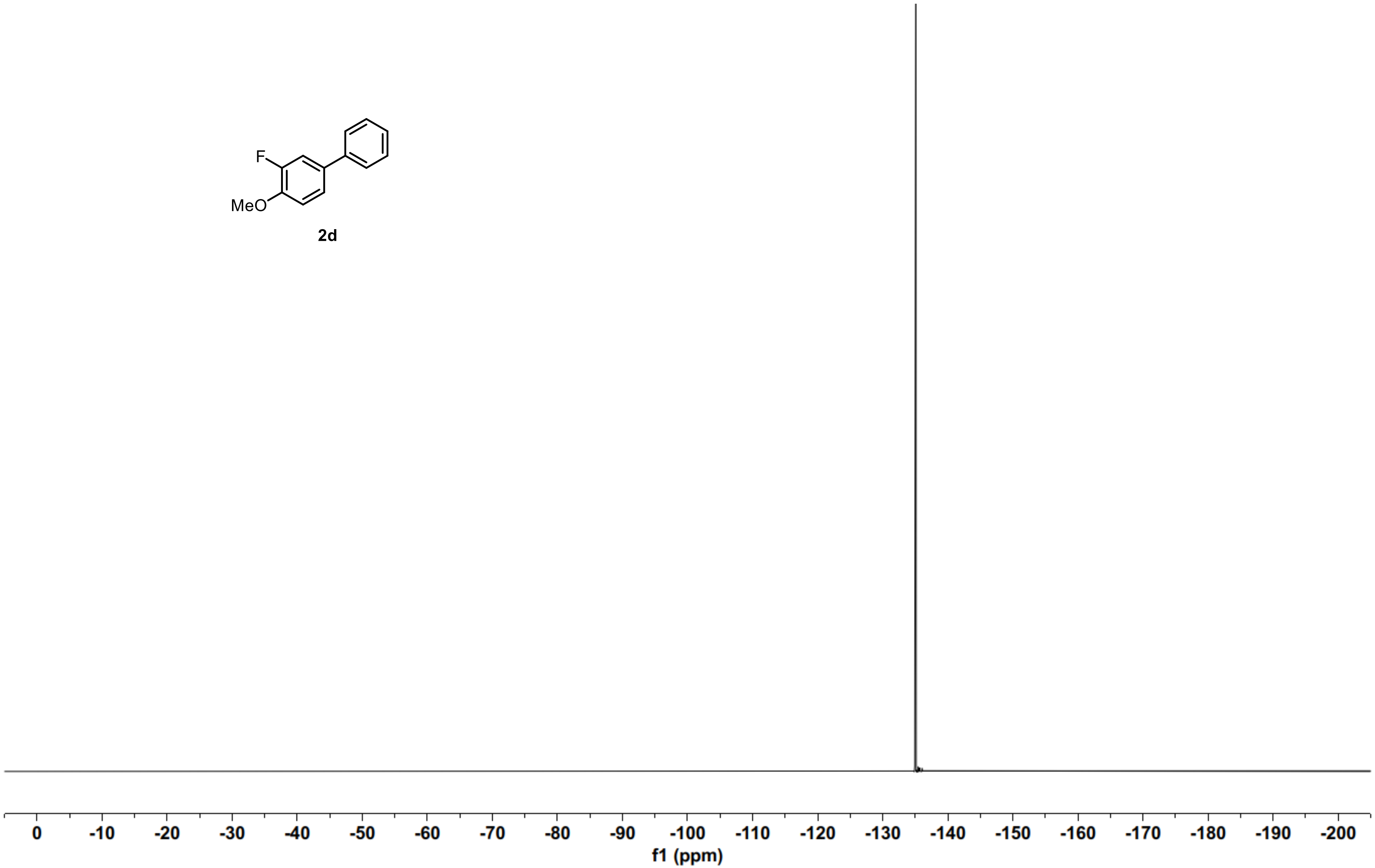

^{1}H NMR of 4a

CDCl$_3$, 500 MHz, 25 °C

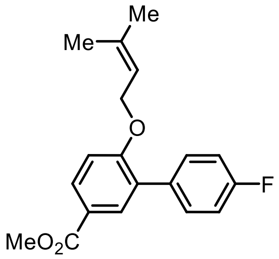

4a

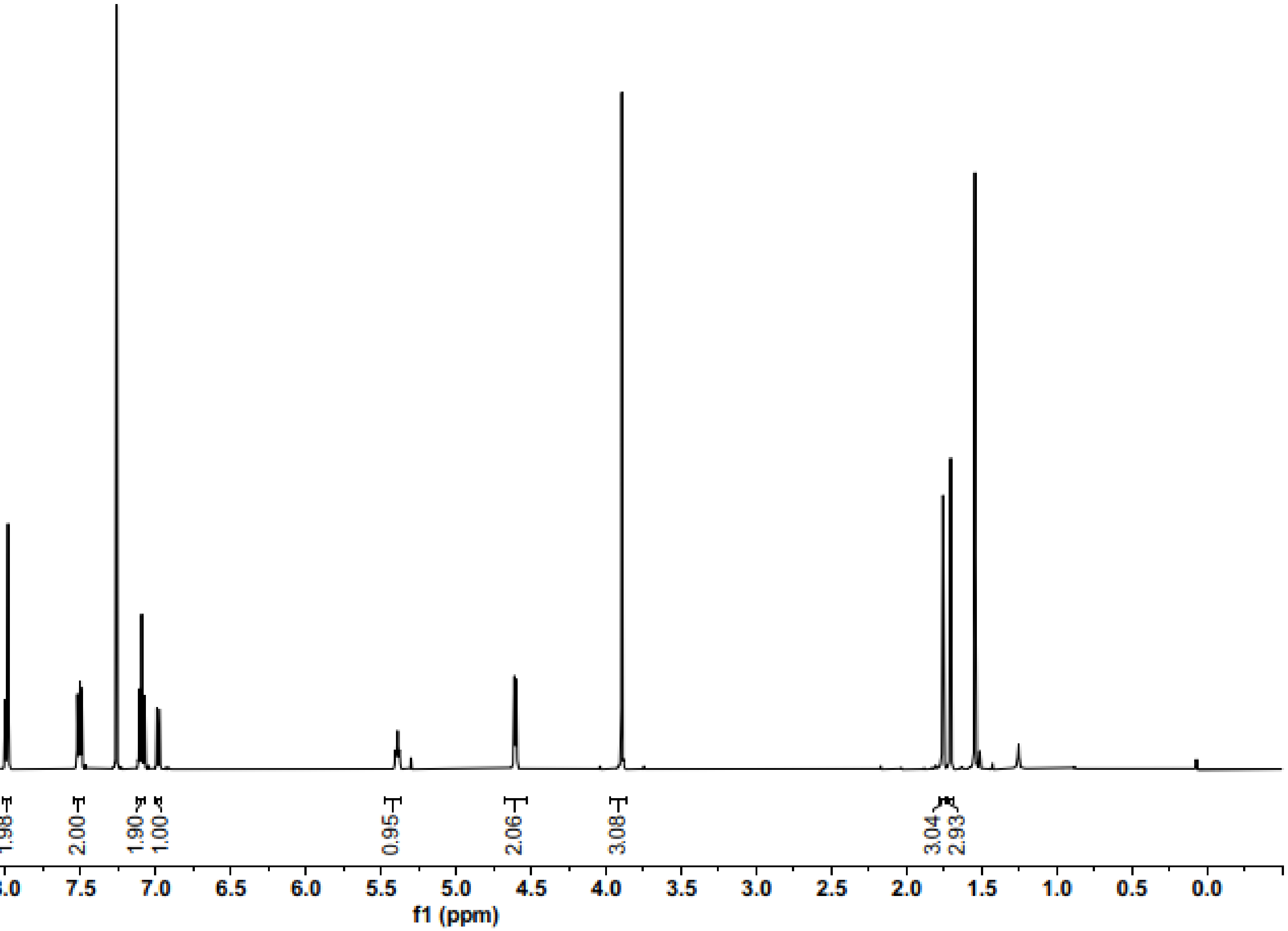

^{13}C NMR of 4a

$CDCl_3$, 126 MHz, 25 °C

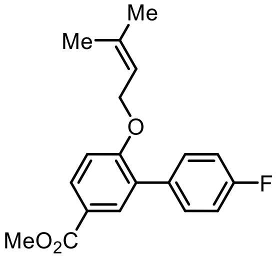

4a

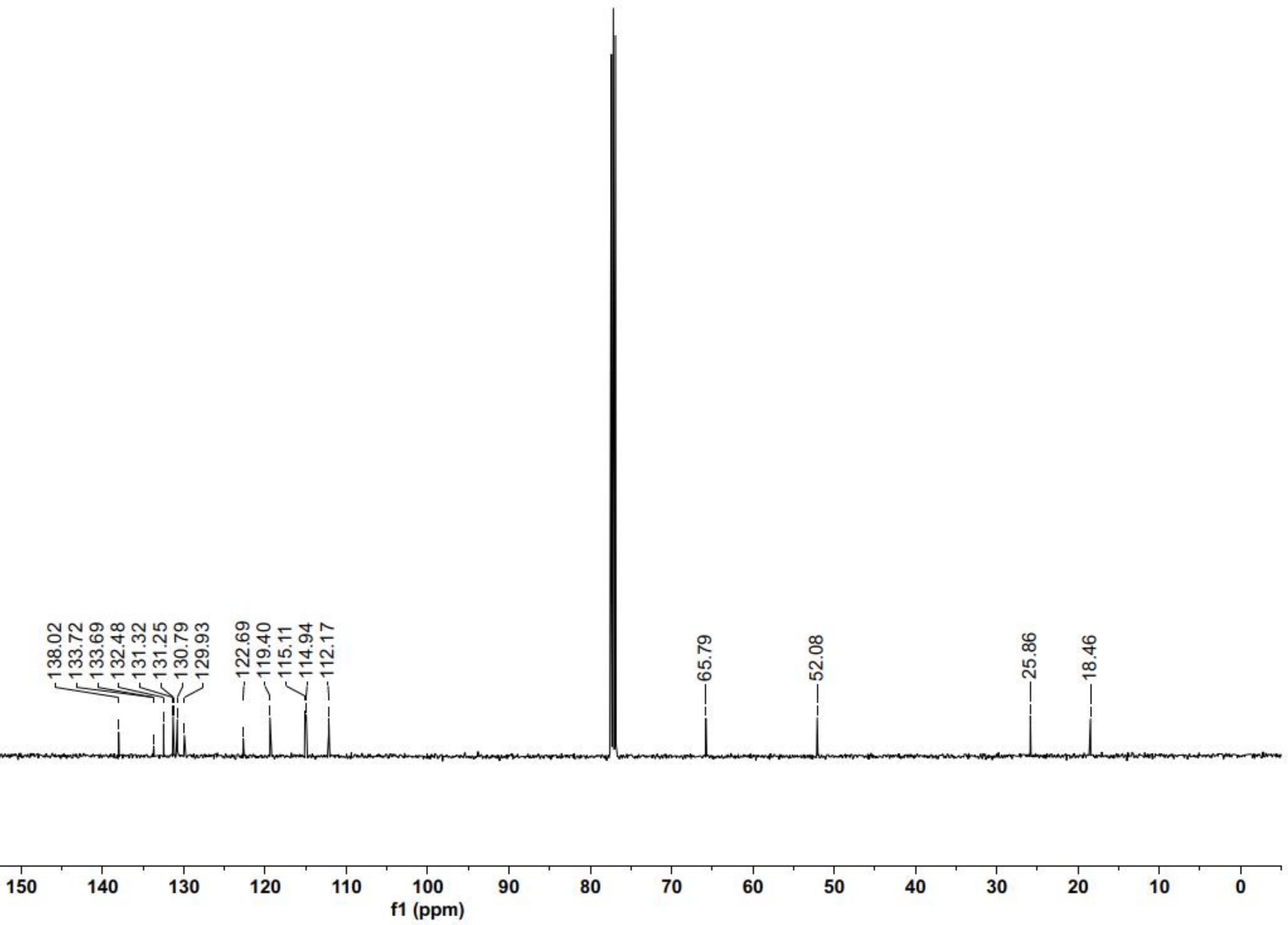

^{19}F NMR of 4a

$CDCl_3$, 471 MHz, 25 °C

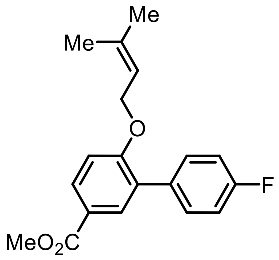

4a

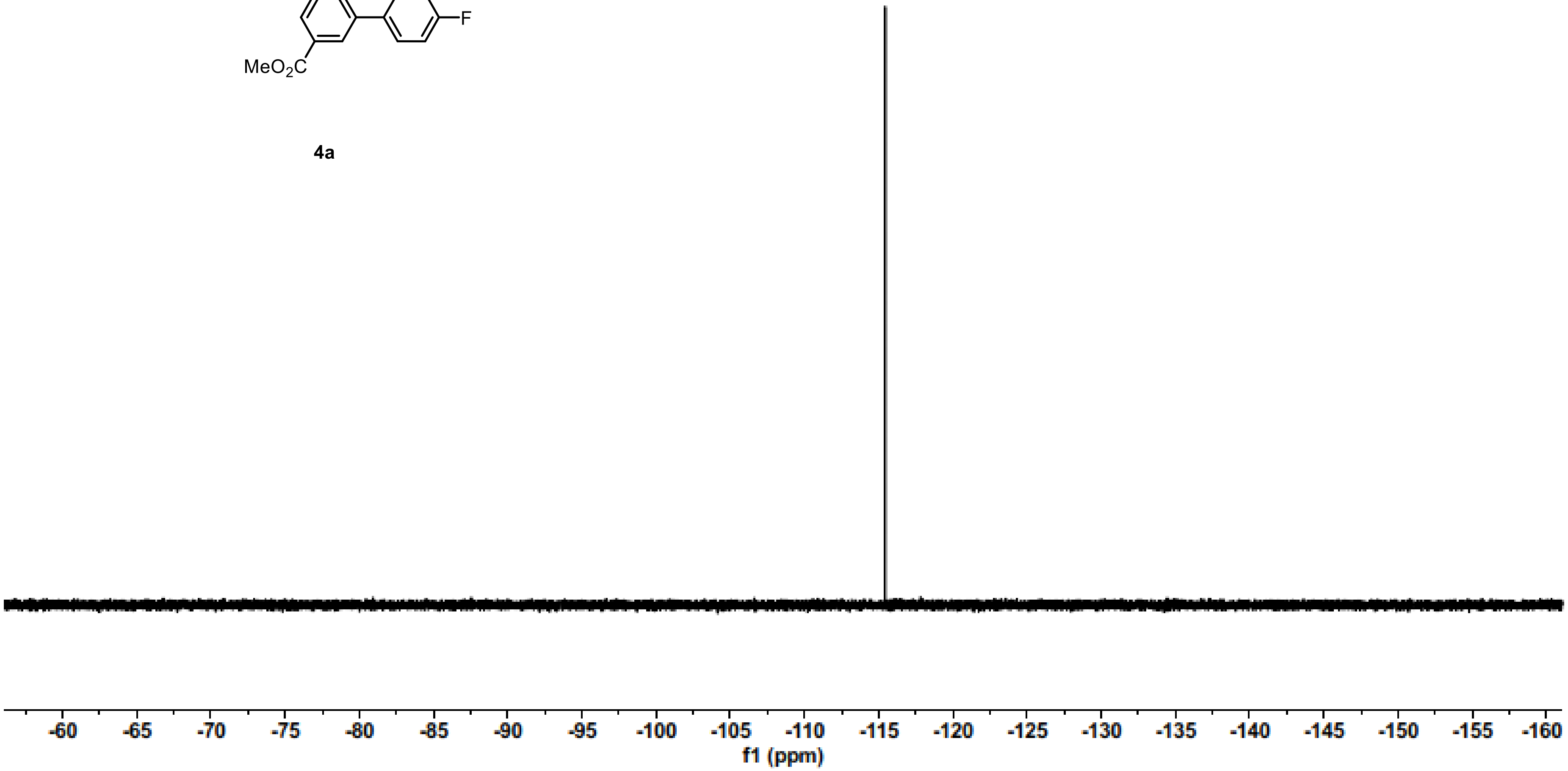

^{1}H NMR of 4b

CDCl3, 500 MHz, 25 °C

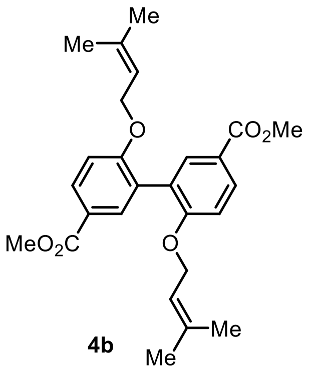

4b

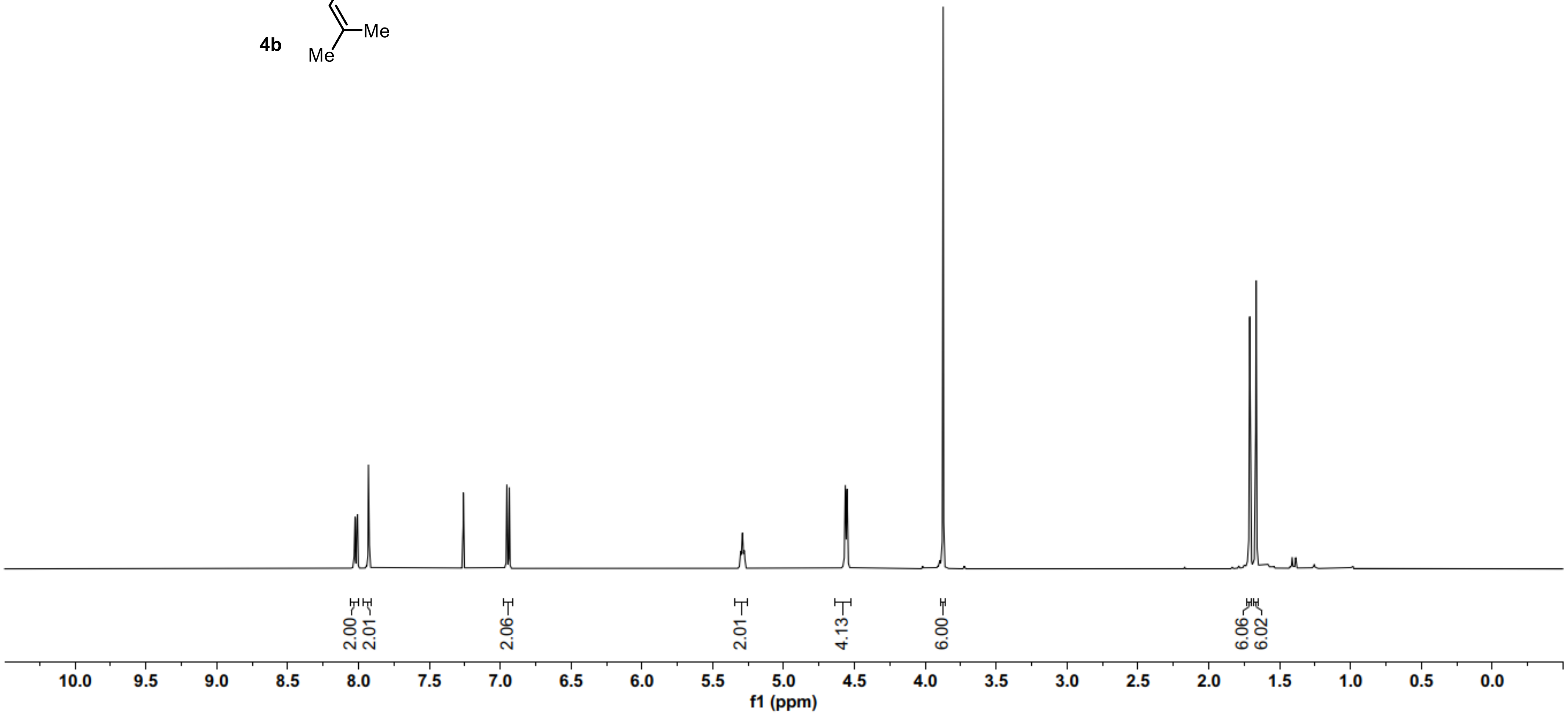

^{13}C NMR of 4b

$CDCl_3$, 126 MHz, 25 °C

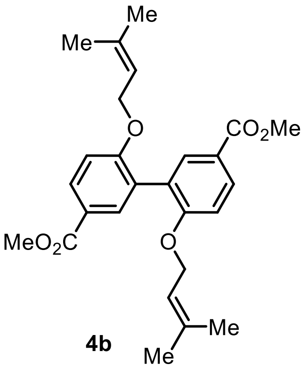

4b

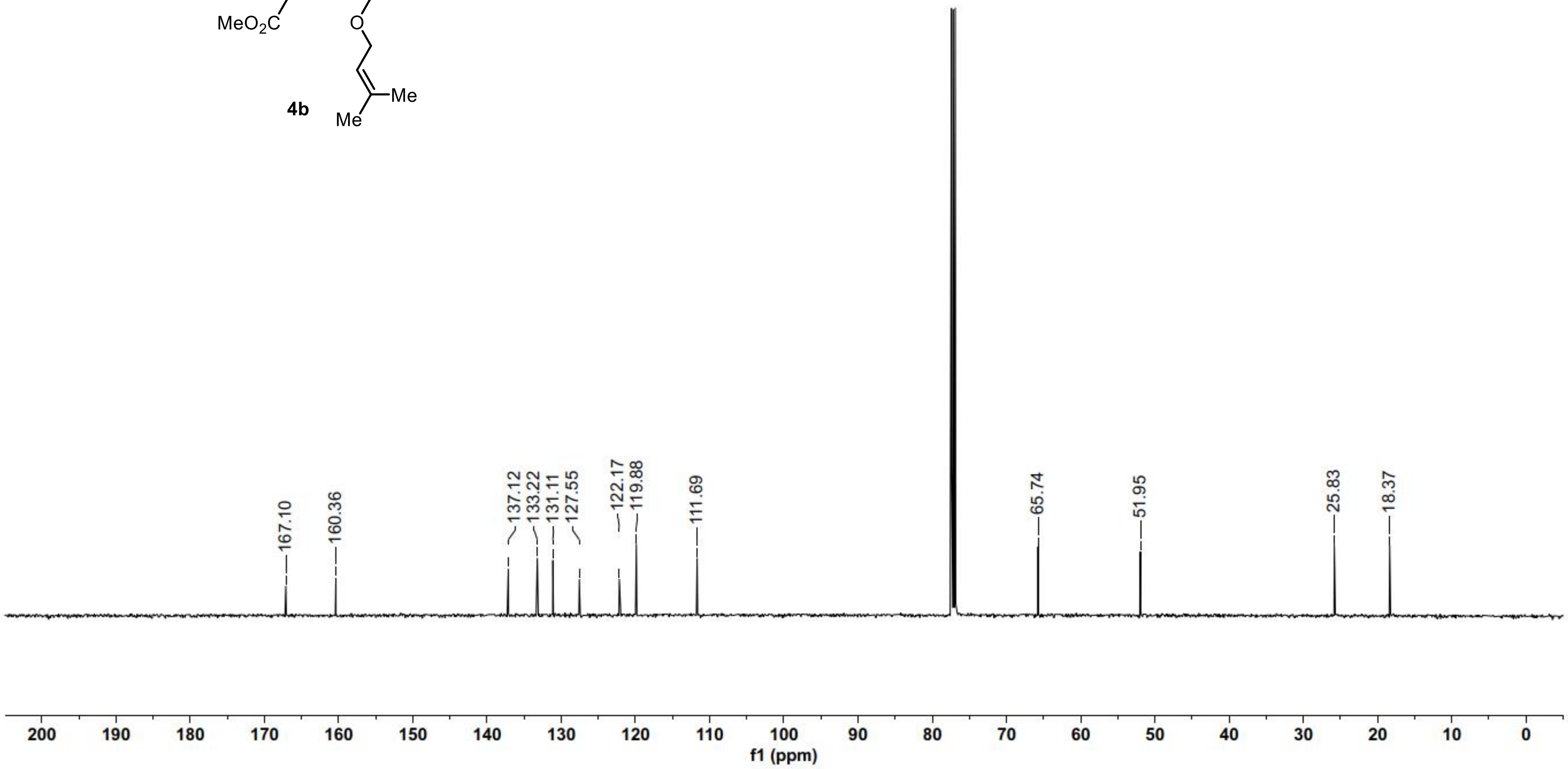

^{1}H NMR of 5

CDCl3, 500 MHz, 25 °C

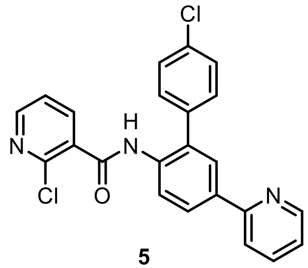

5

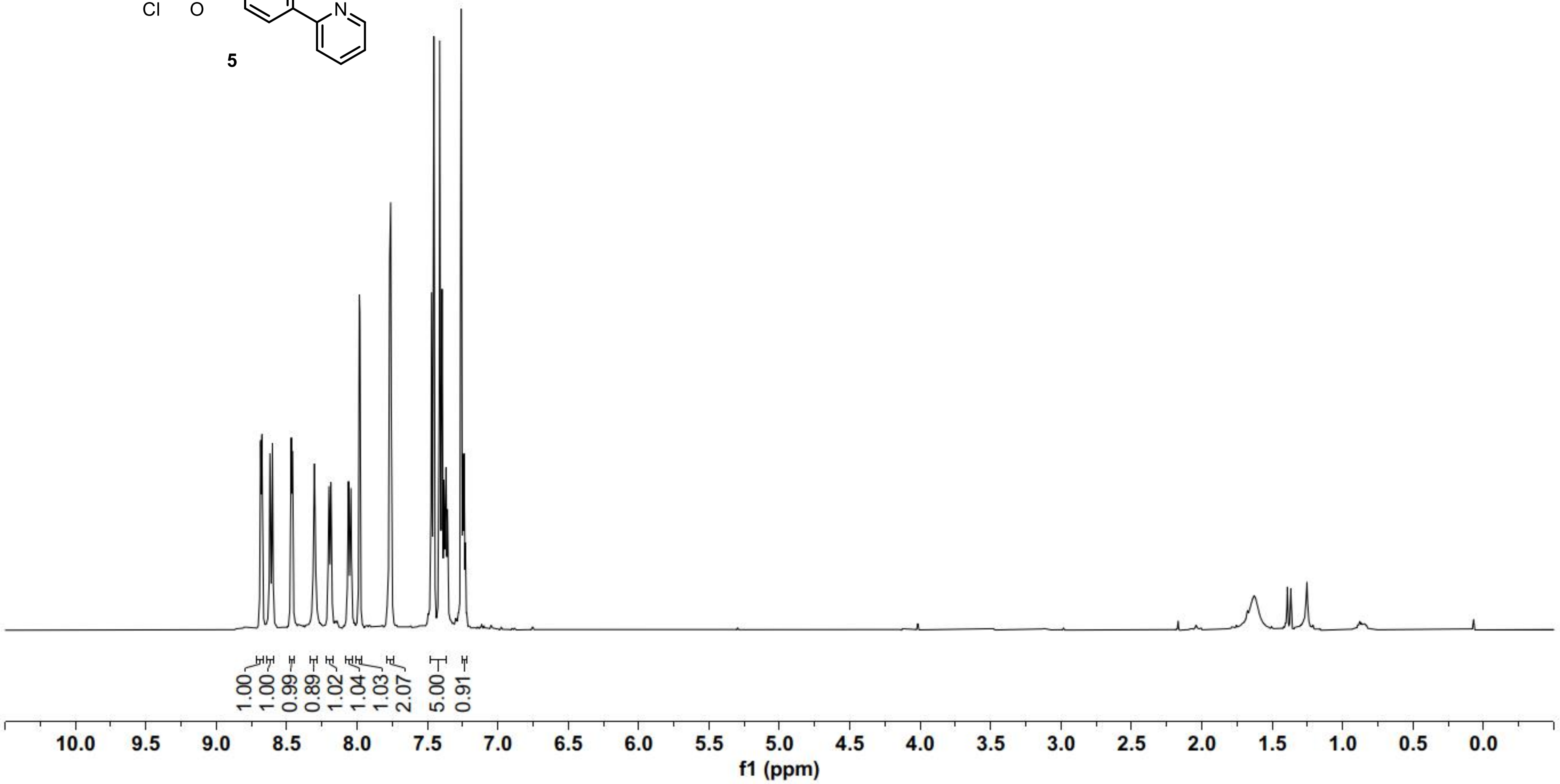

^{13}C NMR of 5

$CDCl_3$, 126 MHz, 25 °C

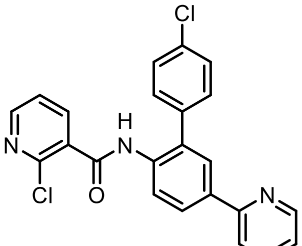

5

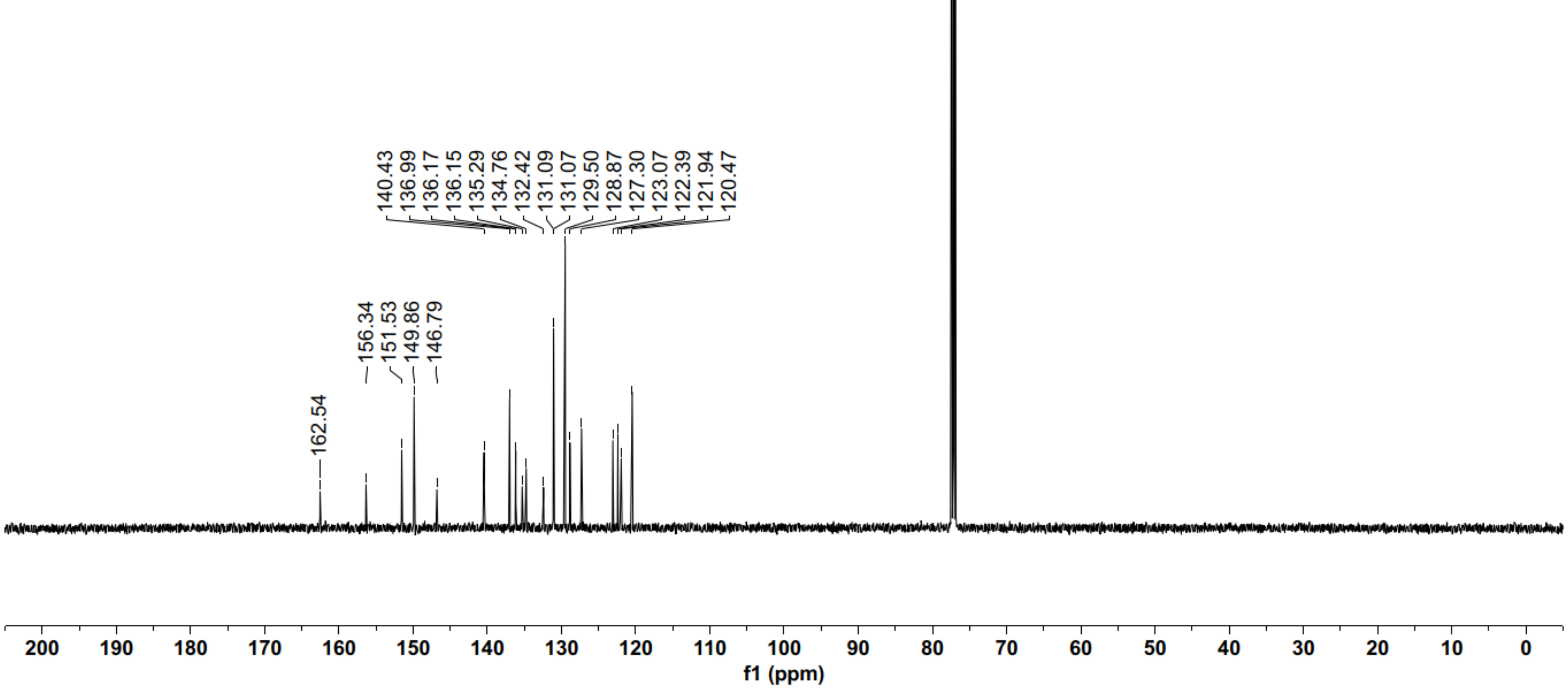

^{1}H NMR of 6

CDCl$_3$, 500 MHz, 25 °C

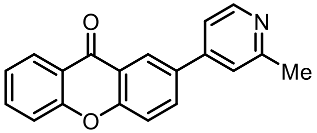

6

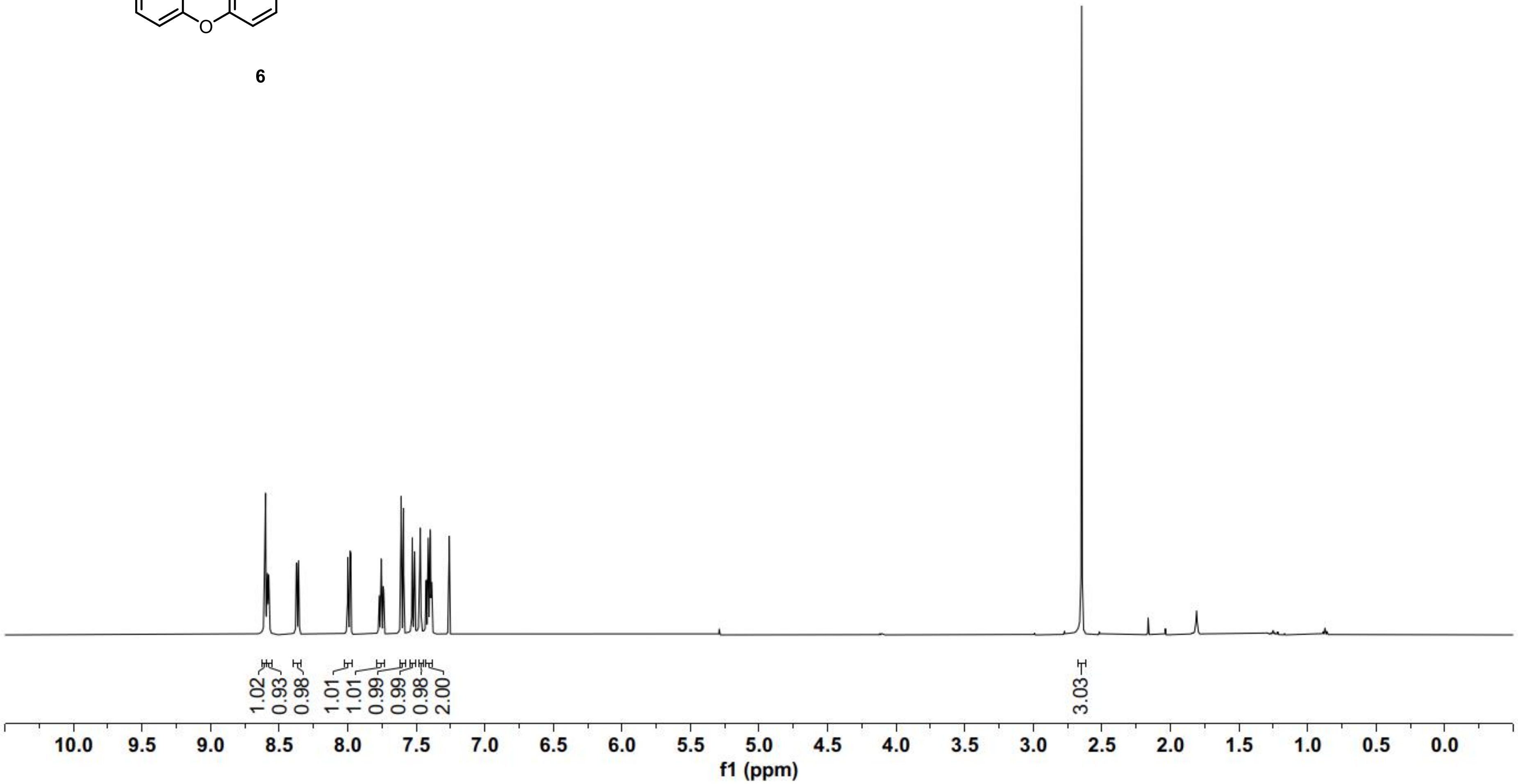

^{13}C NMR of 6

$CDCl_3$, 126 MHz, 25 °C

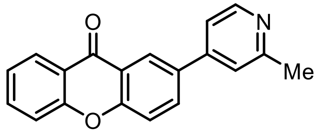

6

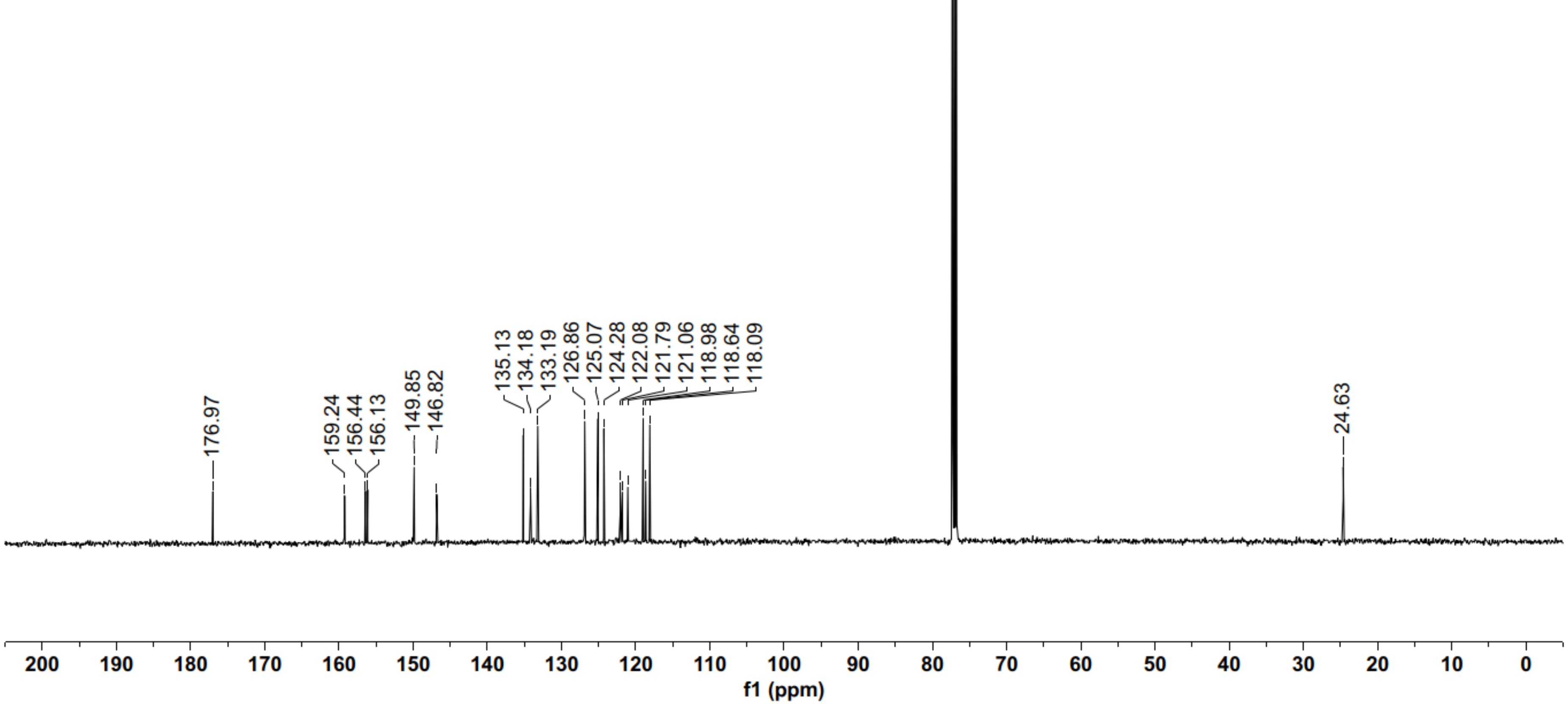

^{1}H NMR of 7

$CDCl_3$, 500 MHz, 25 °C

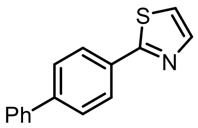

7

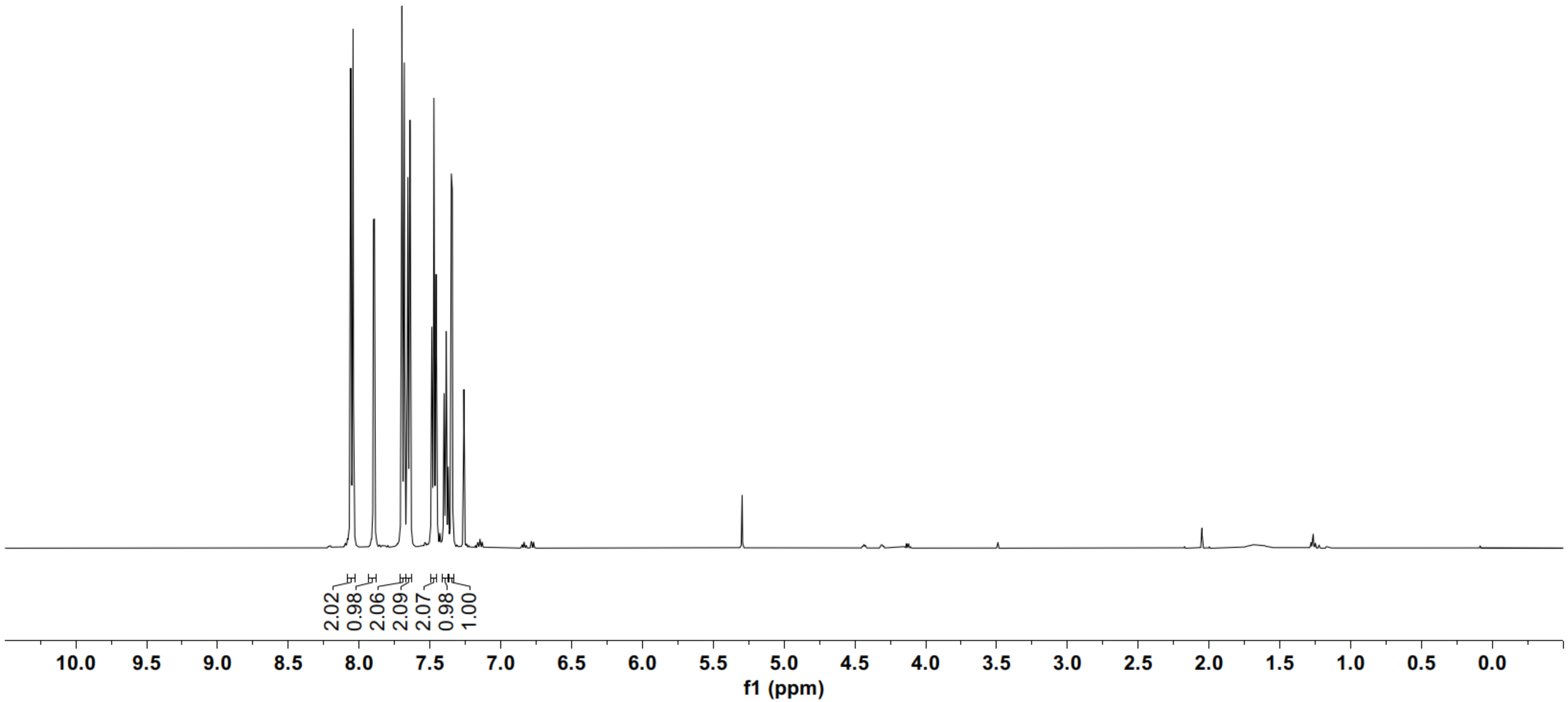

^{13}C NMR of 7

CDCl$_3$, 126 MHz, 25 °C

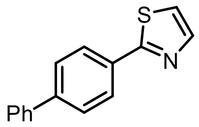

7

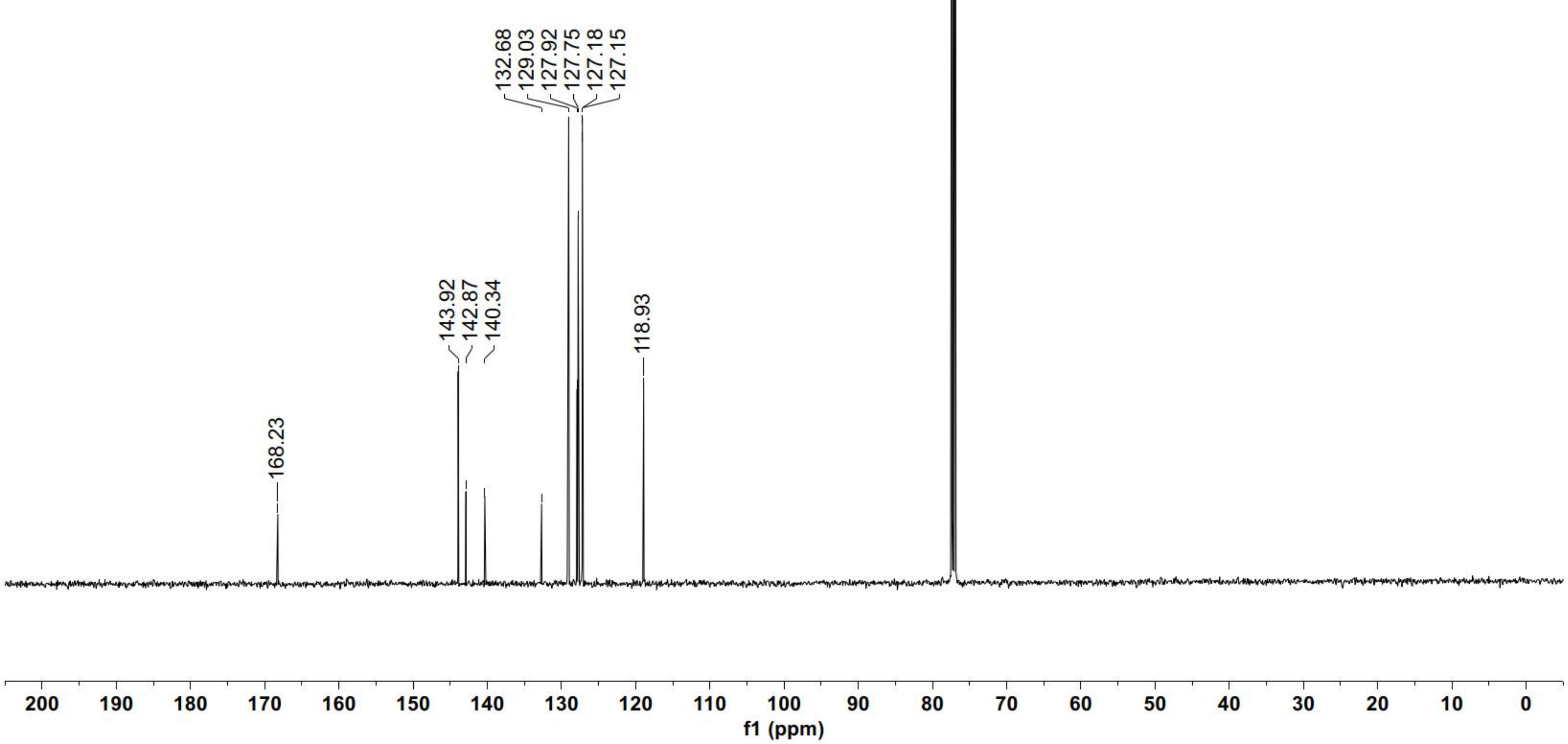

^{1}H NMR of 8

CDCl$_3$, 500 MHz, 25 °C

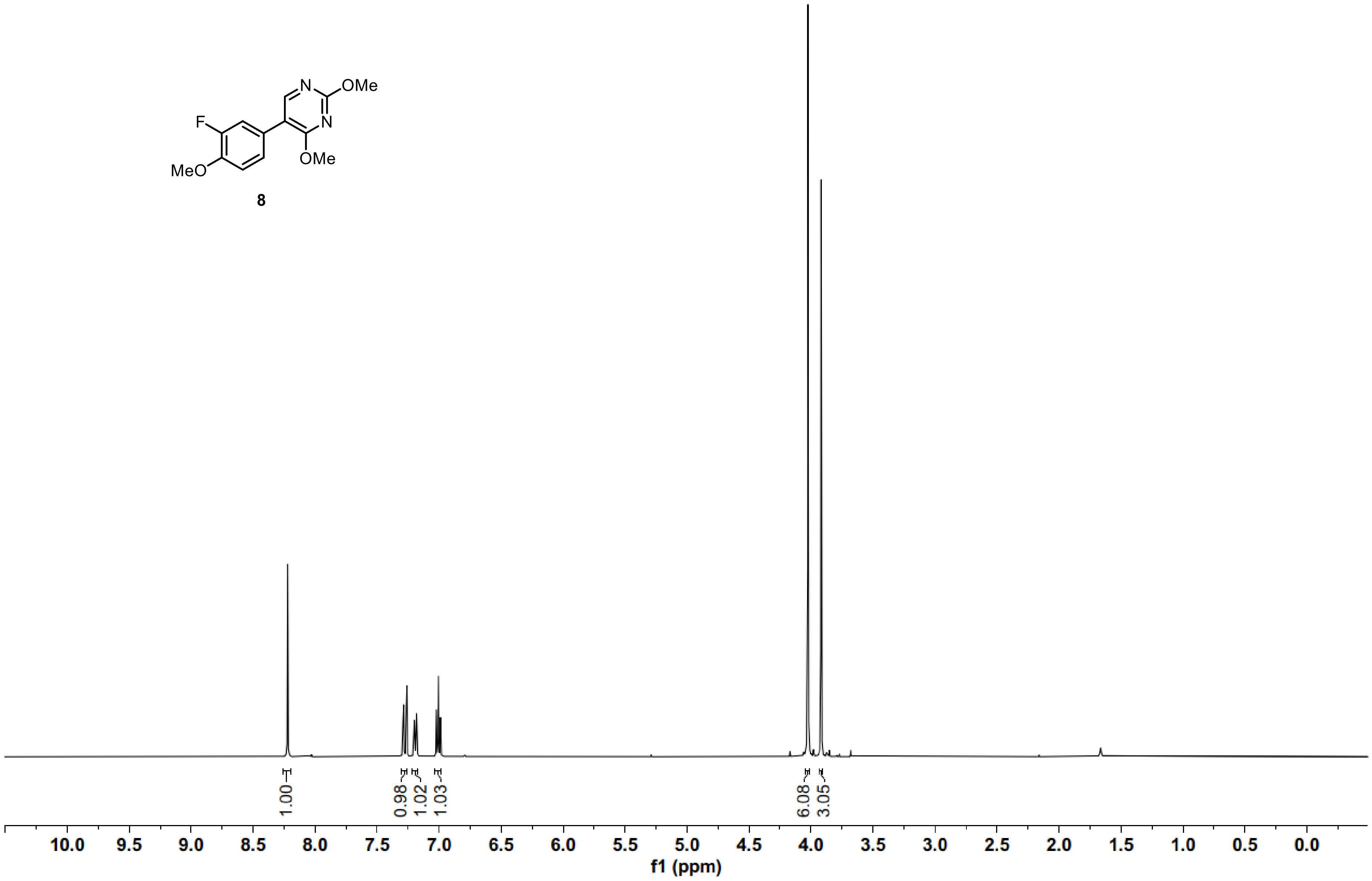

^{13}C NMR of 8

$CDCl_3$, 126 MHz, 25 °C

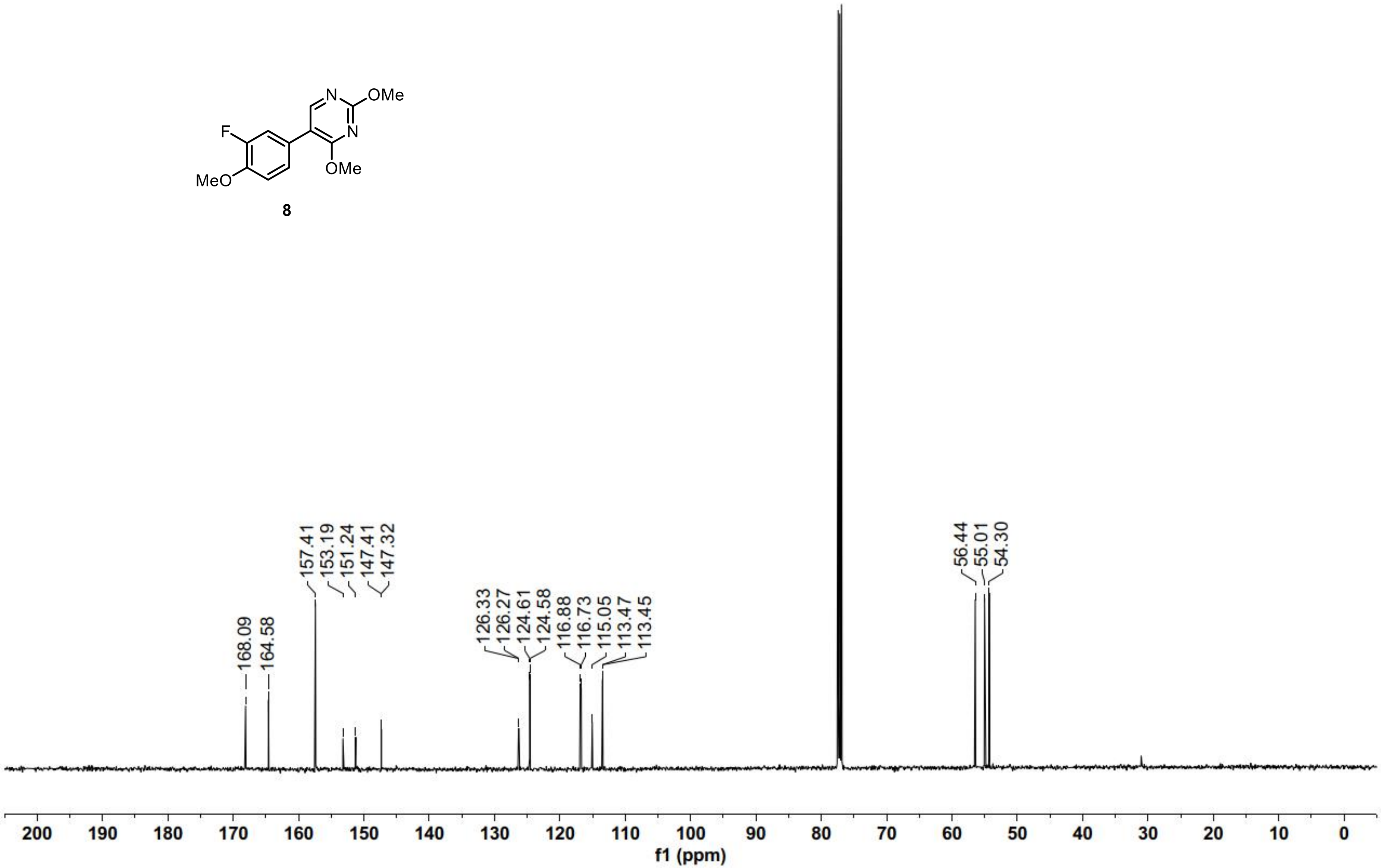

^{19}F NMR of 8

CDCl3, 471 MHz, 25 °C

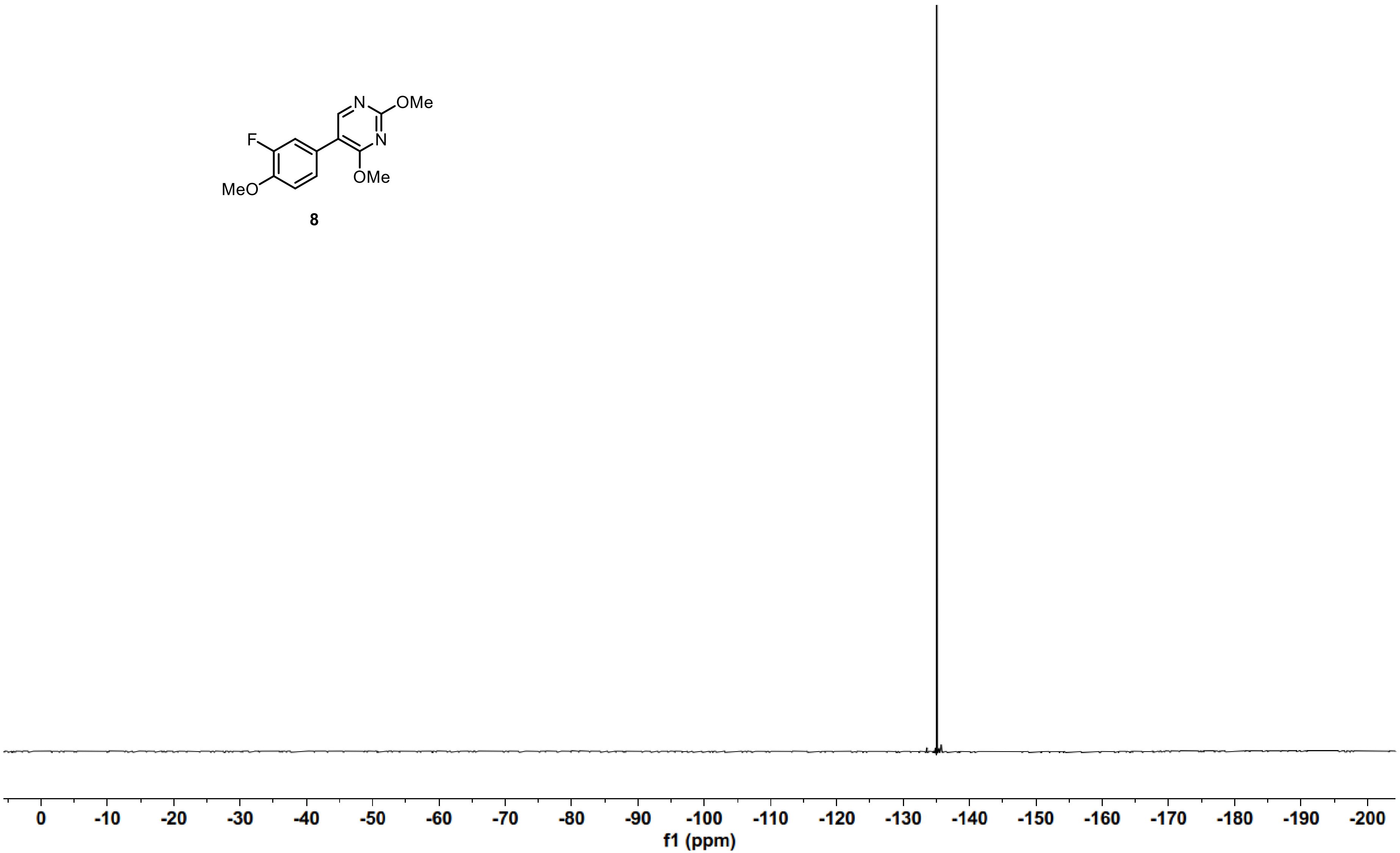

^{1}H NMR of 9

CDCl$_3$, 500 MHz, 25 °C

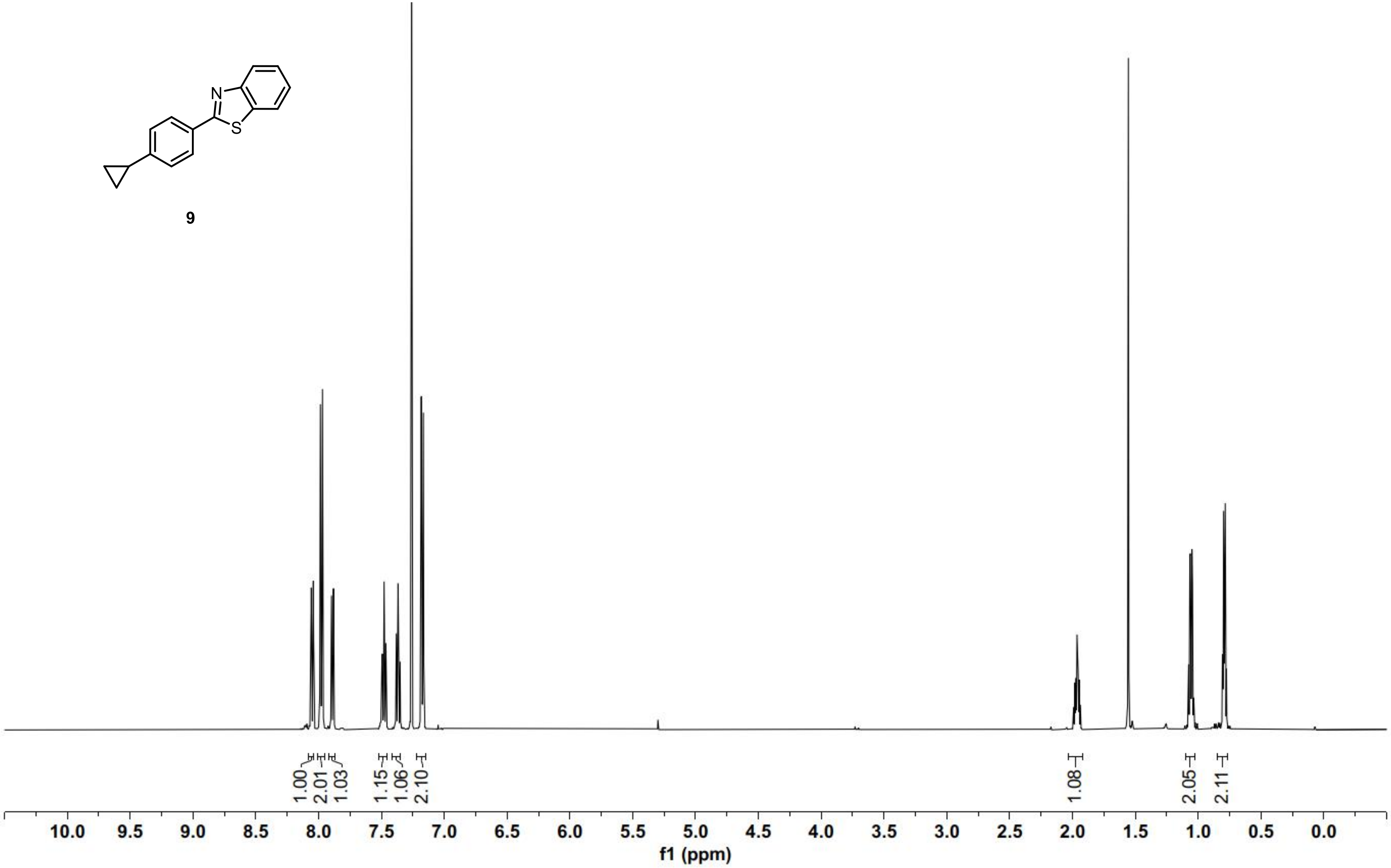

^{13}C NMR of 9

$CDCl_3$, 126 MHz, 25 °C

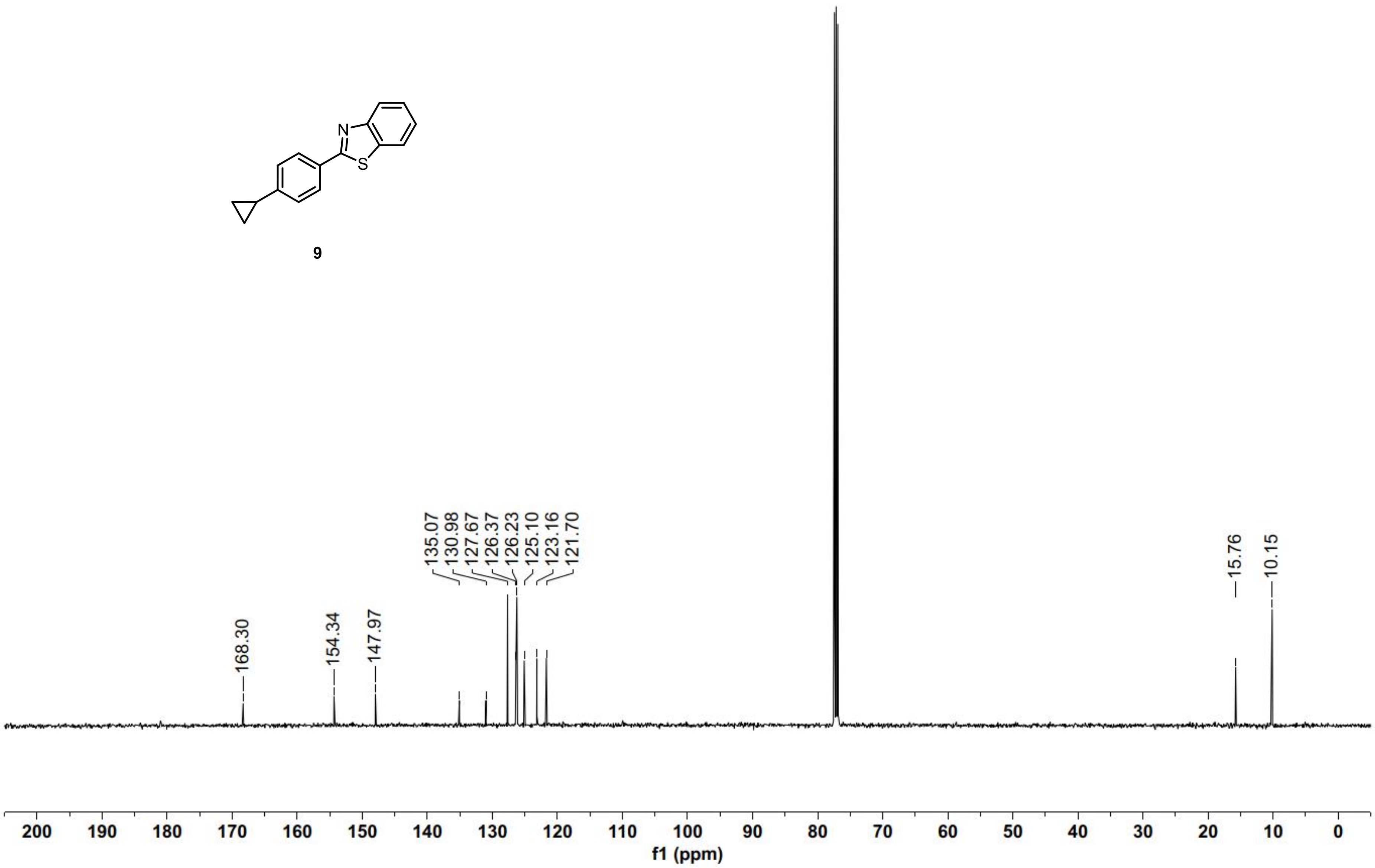

^{1}H NMR of 10

$CDCl_3$, 500 MHz, 25 °C

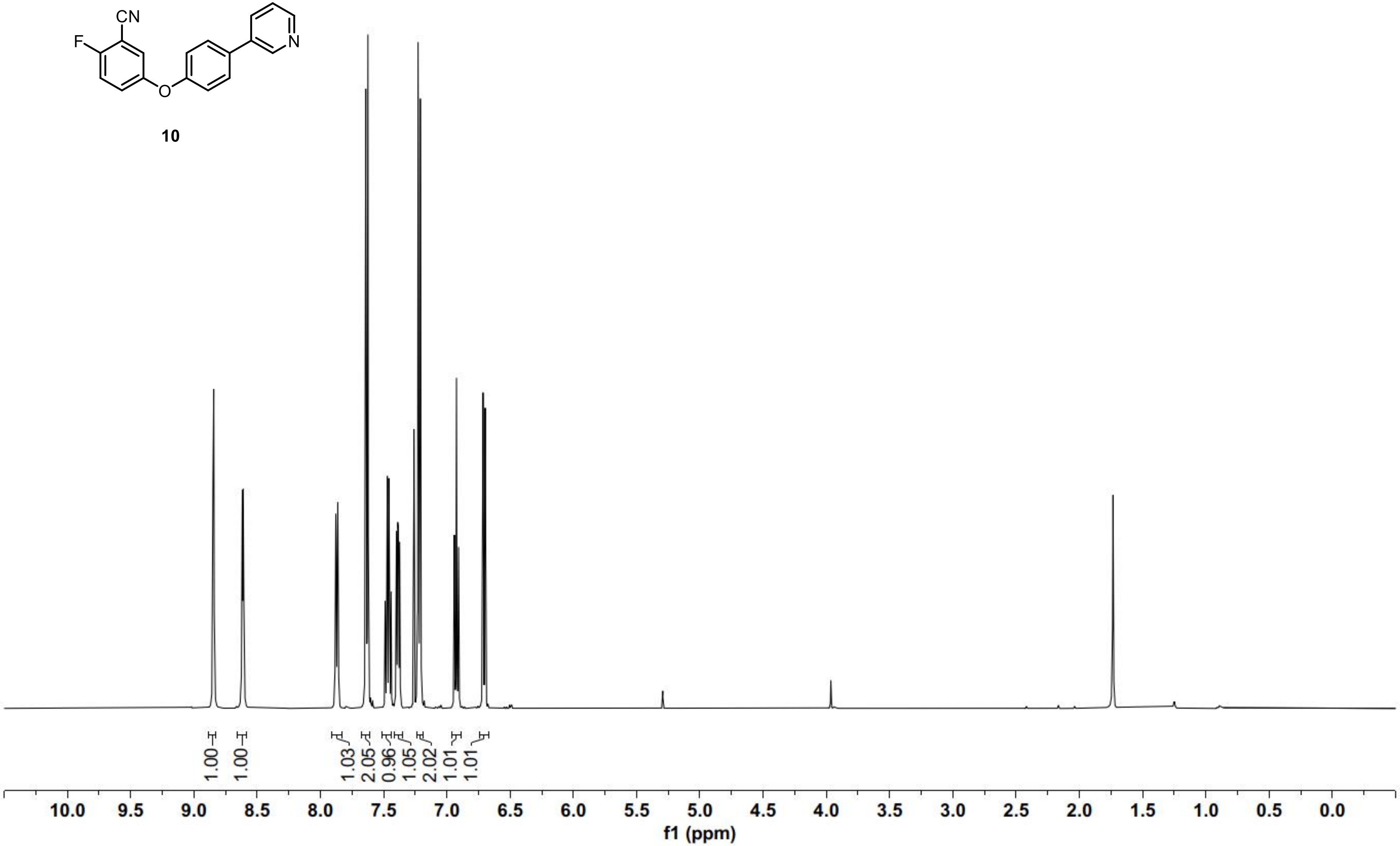

^{13}C NMR of 10

$CDCl_3$, 126 MHz, 25 °C

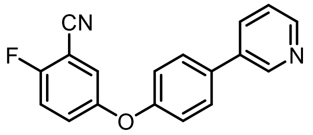

10

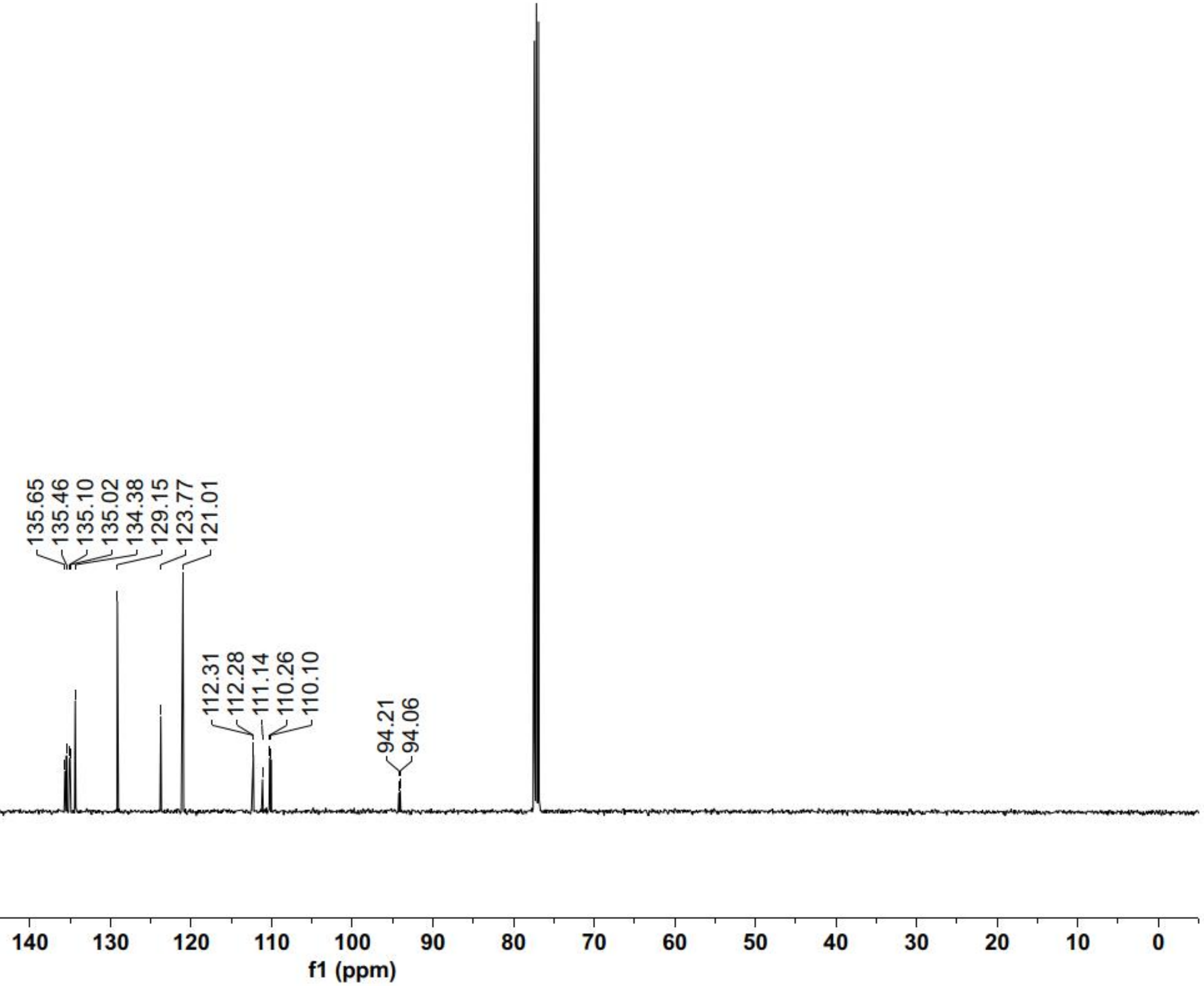

^{19}F NMR of 10

$CDCl_3$, 471 MHz, 25 °C

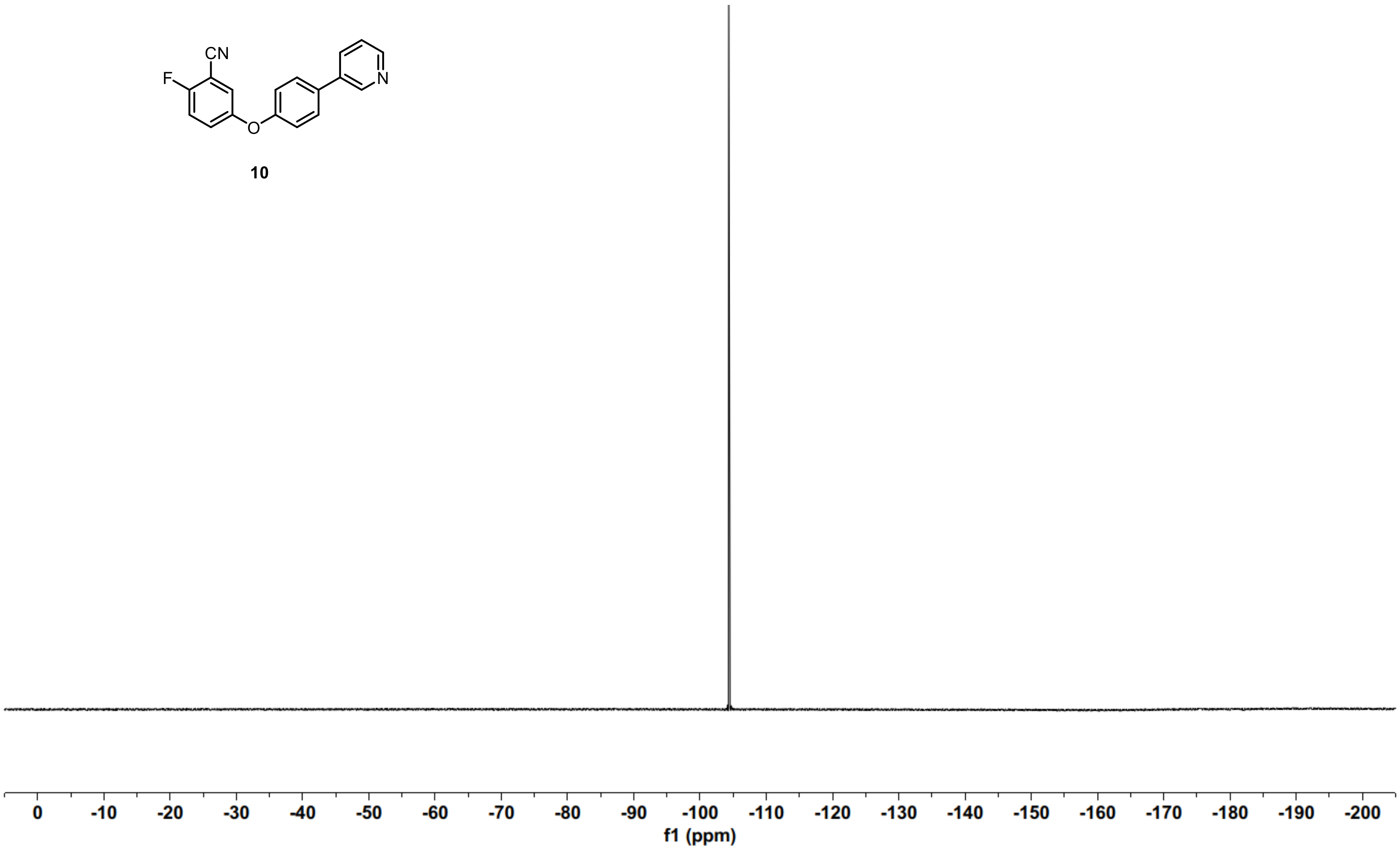

10

^{1}H NMR of 11

$CDCl_3$, 500 MHz, 25 °C

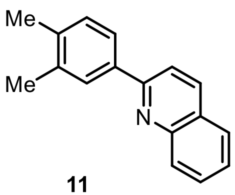

11

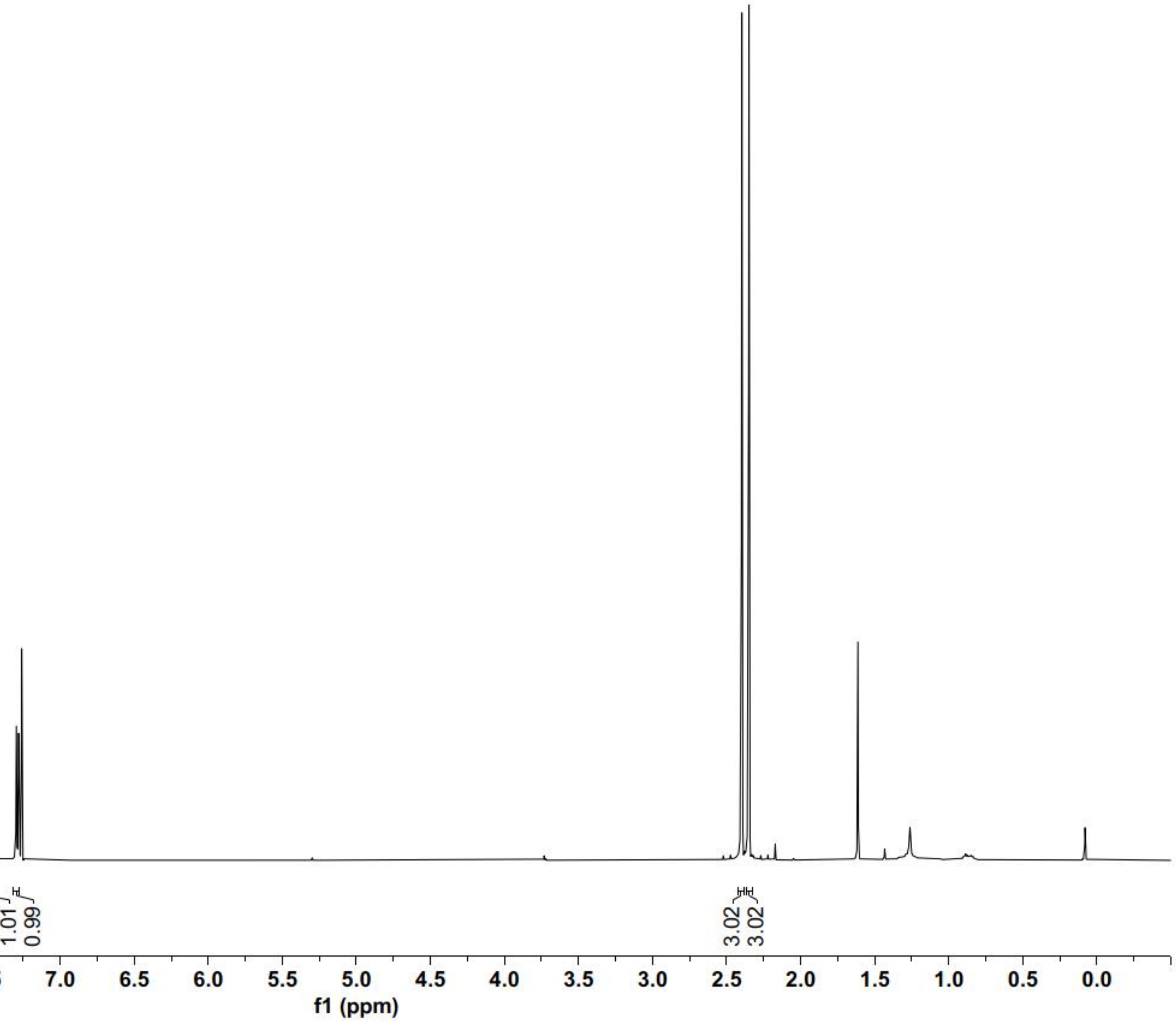

^{13}C NMR of 11

CDCl$_3$, 126 MHz, 25 °C

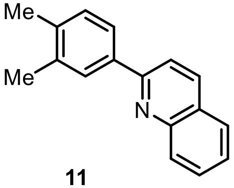

11

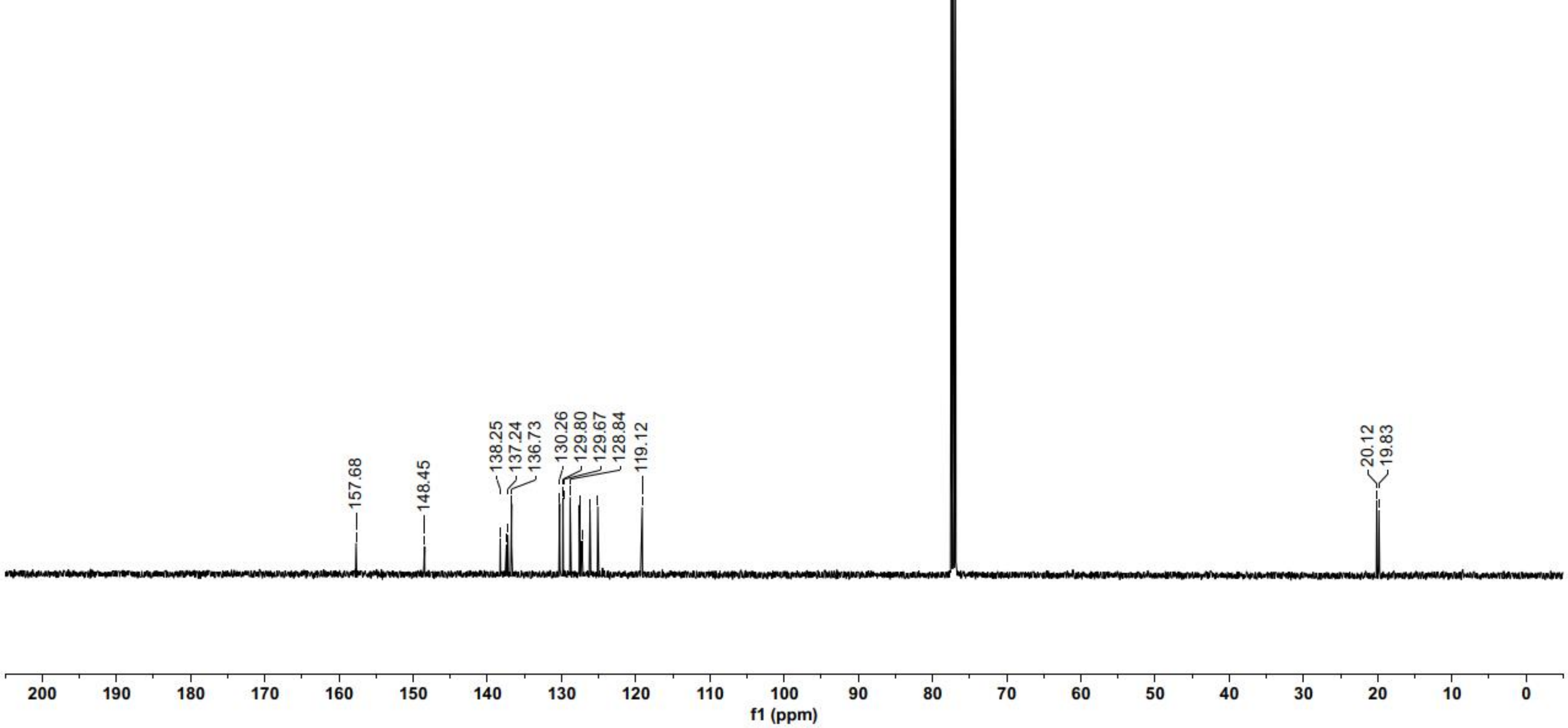

^{1}H NMR of 12

CDCl3, 500 MHz, 25 °C

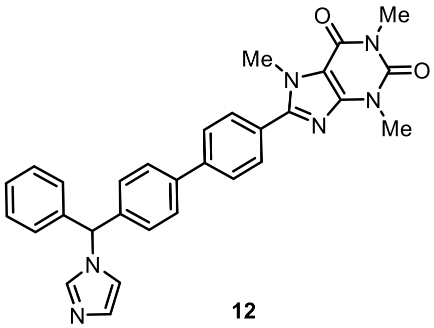

12

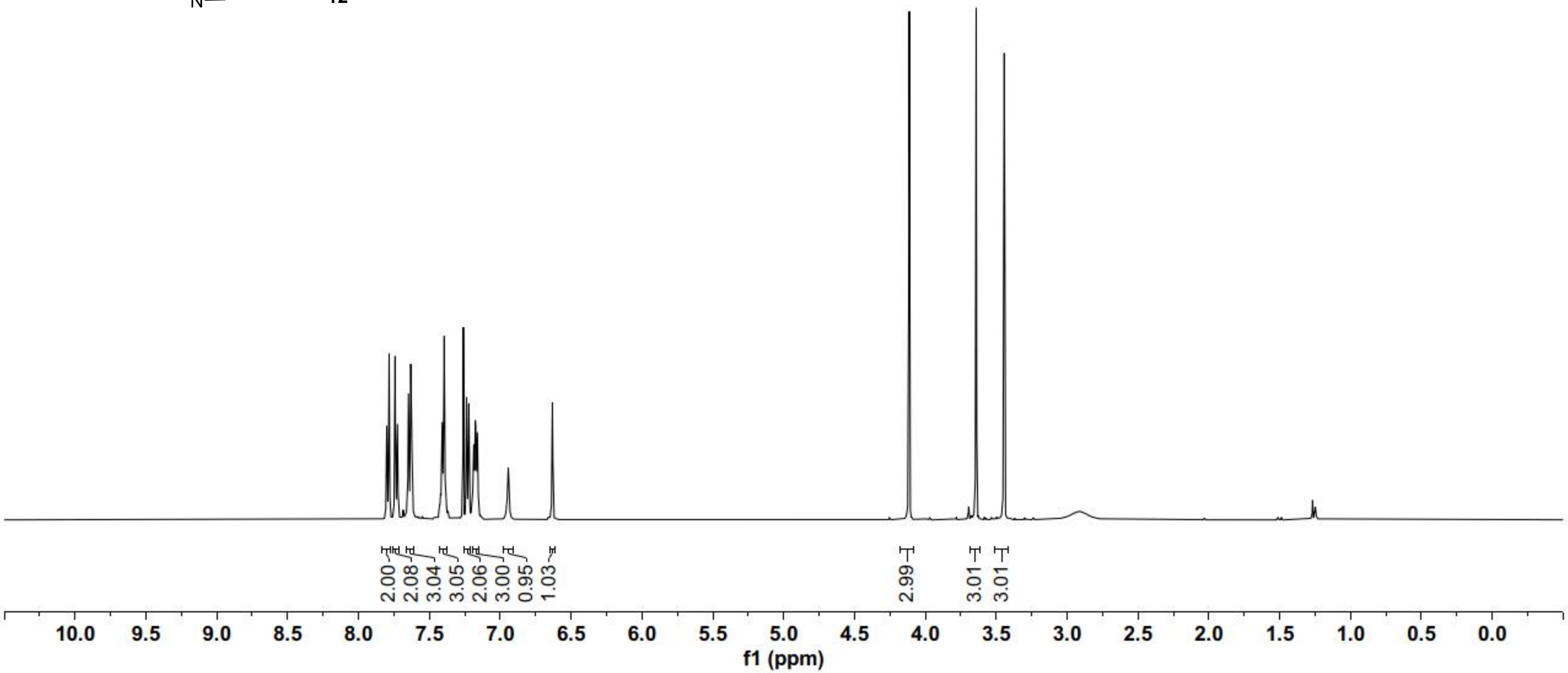

^{13}C NMR of 12

$CDCl_3$, 126 MHz, 25 °C

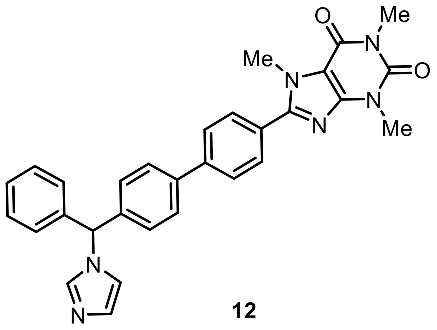

12

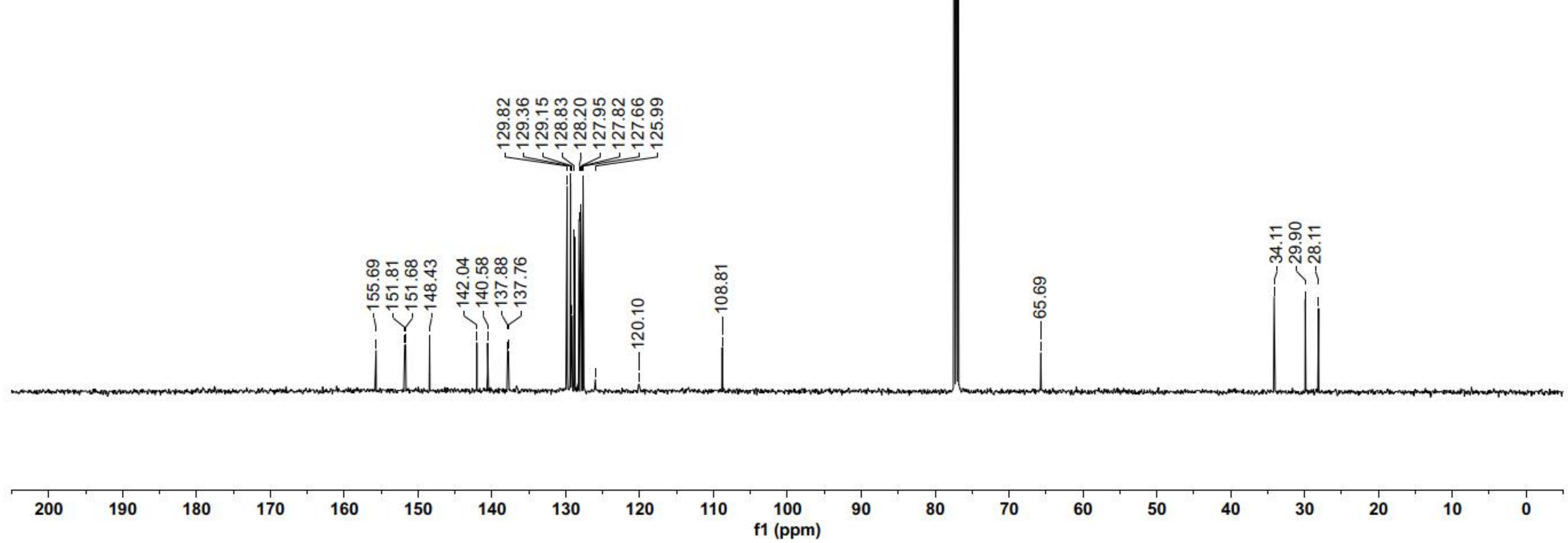

^{1}H NMR of 13

CDCl$_3$, 500 MHz, 25 °C

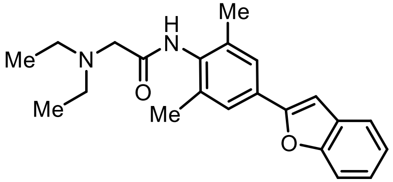

13

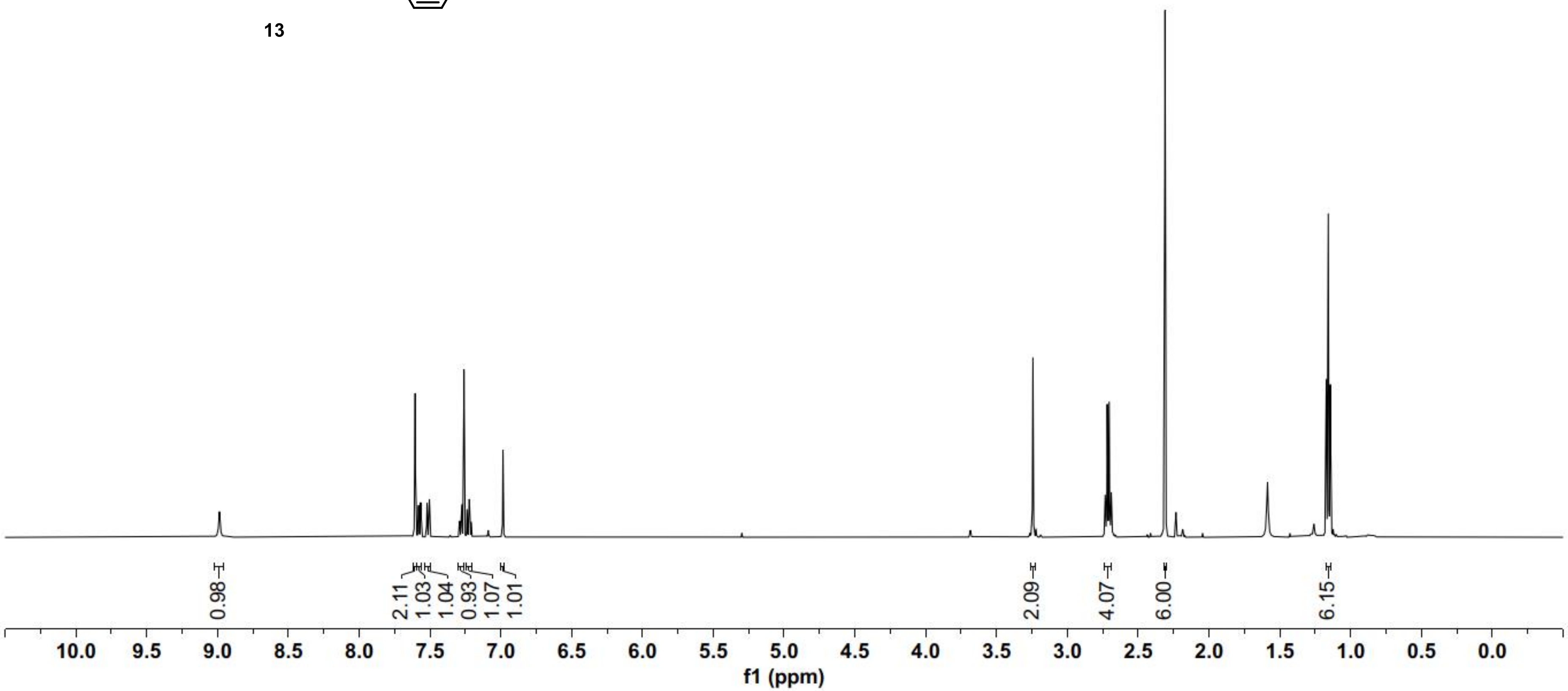

^{13}C NMR of 13

$CDCl_3$, 126 MHz, 25 °C

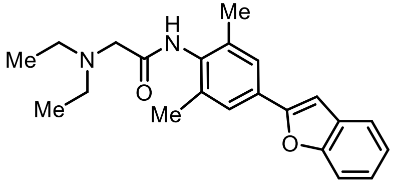

13

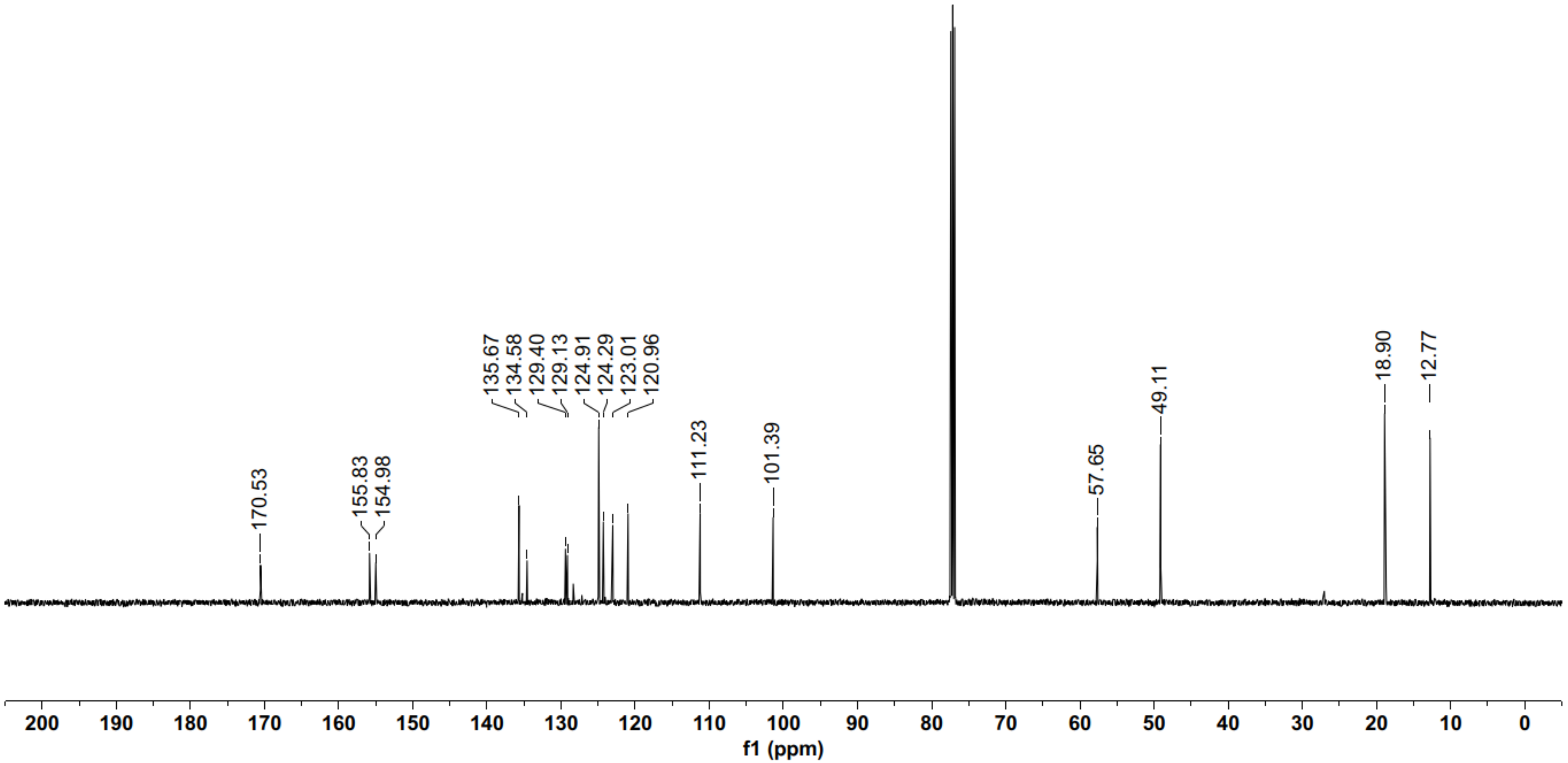

^{1}H NMR of 14

$CDCl_3$, 500 MHz, 25 °C

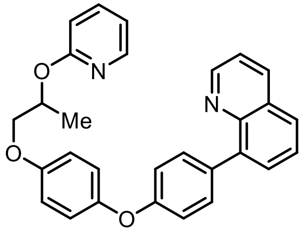

14

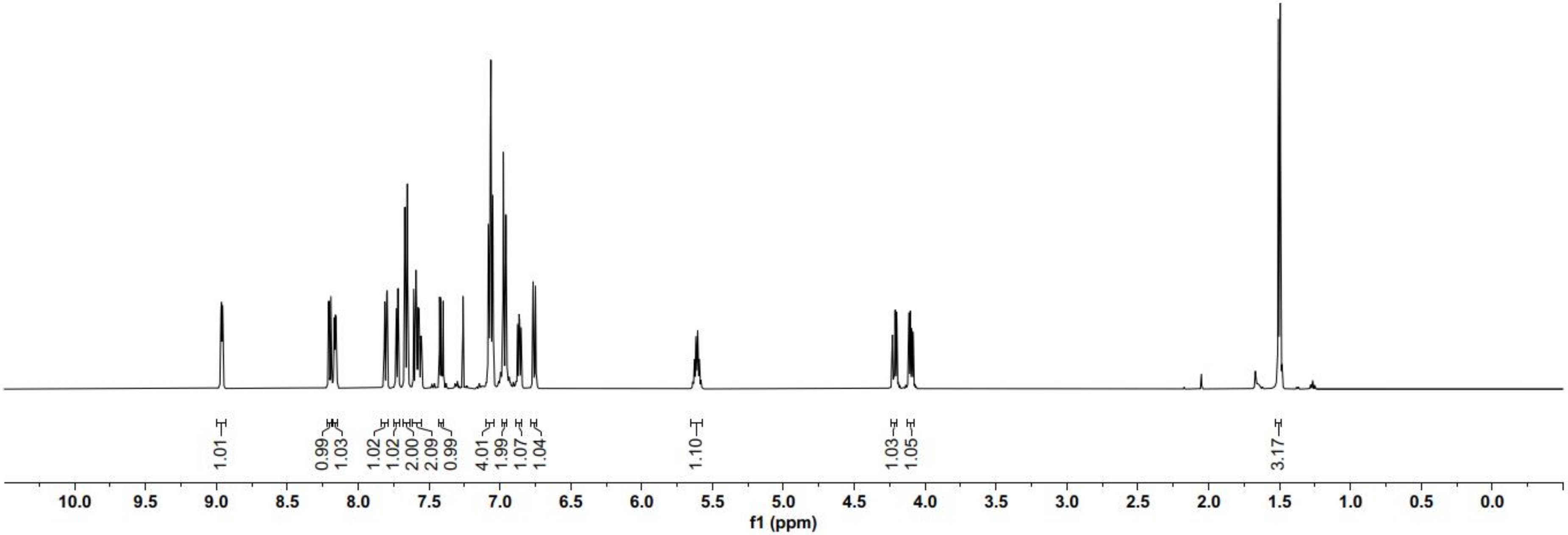

^{13}C NMR of 14

$CDCl_3$, 126 MHz, 25 °C

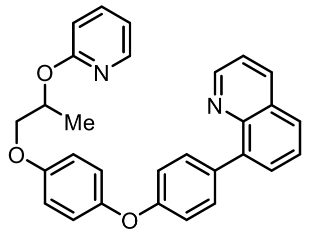
14

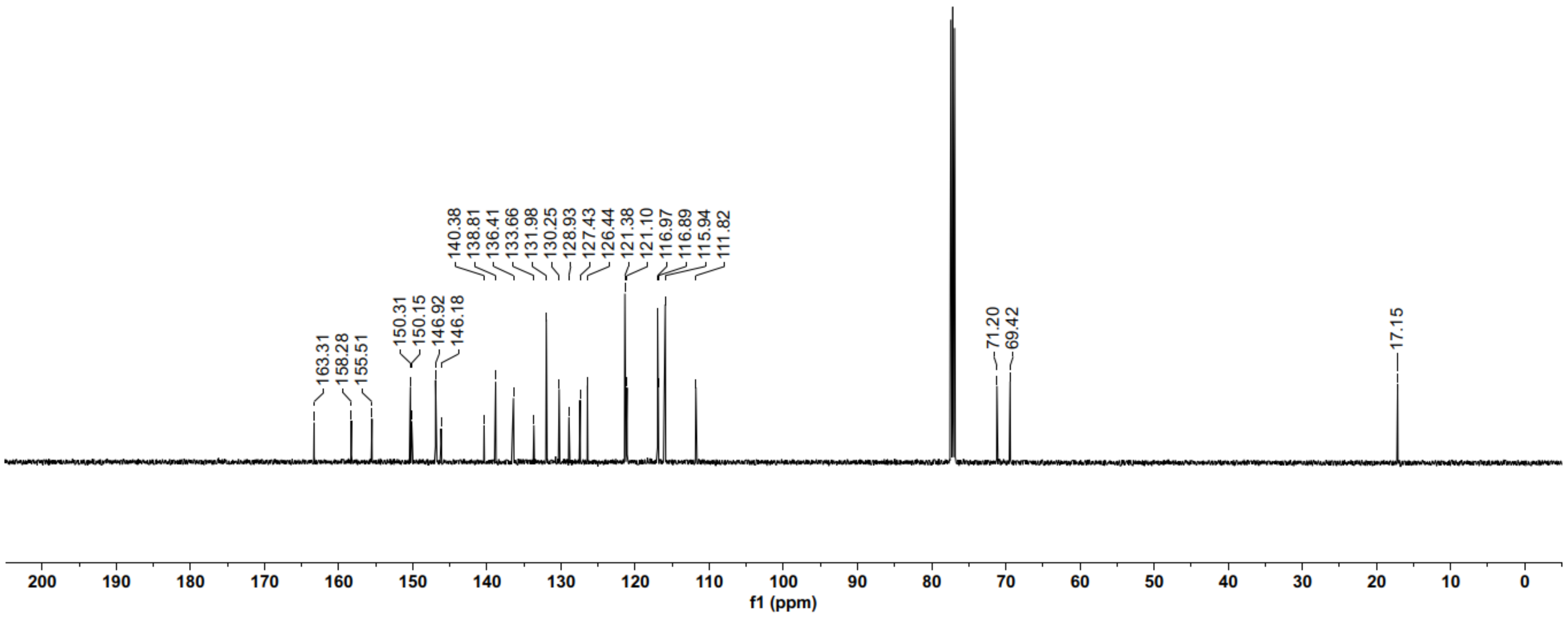

^{1}H NMR of 15

$CDCl_3$, 500 MHz, 25 °C

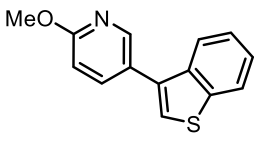

15

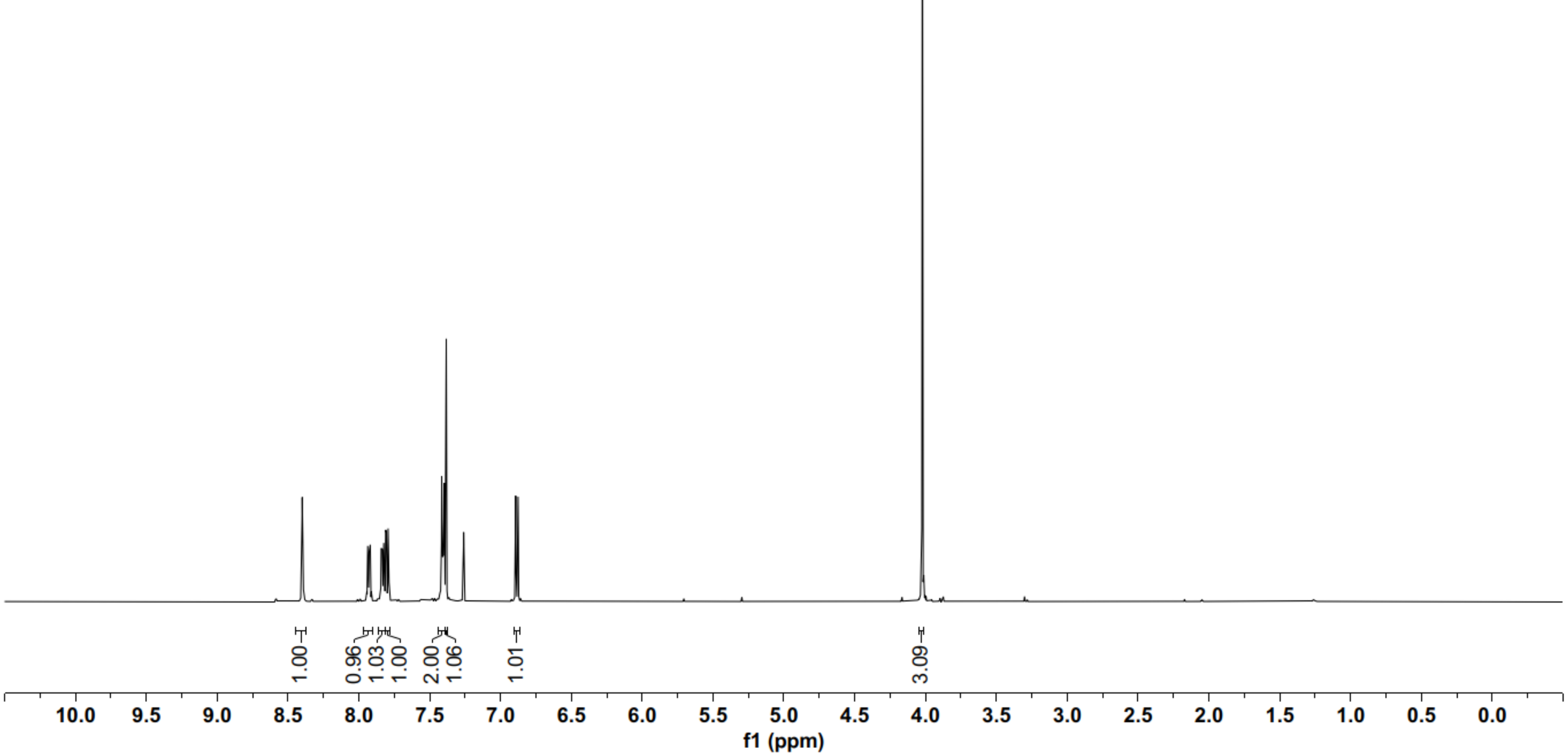

^{13}C NMR of 15

$CDCl_3$, 126 MHz, 25 °C

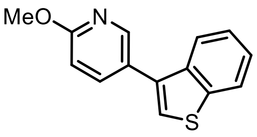

15

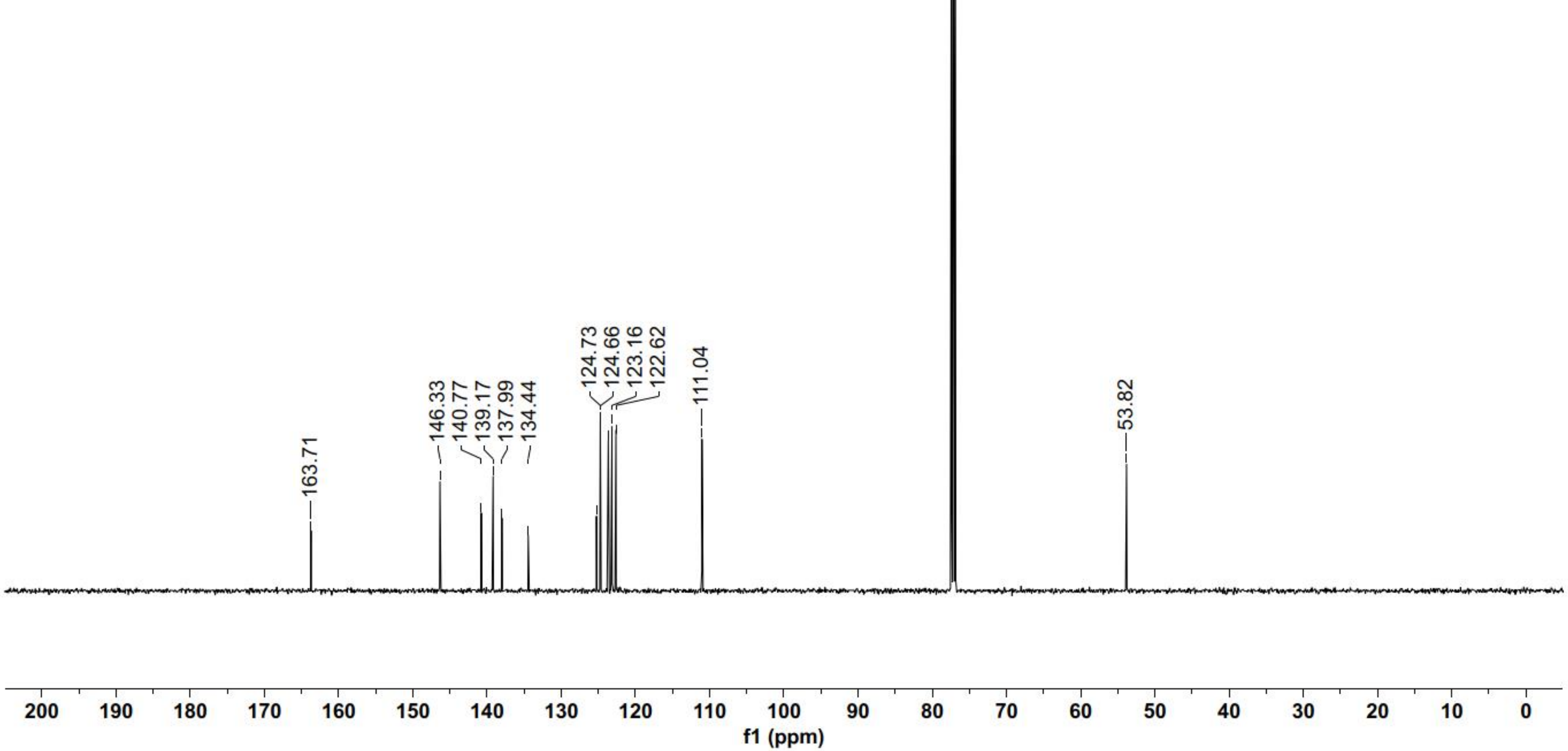

^{1}H NMR of 16

$CDCl_3$, 500 MHz, 25 °C

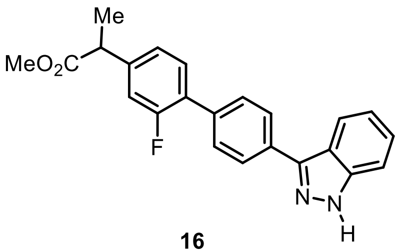

16

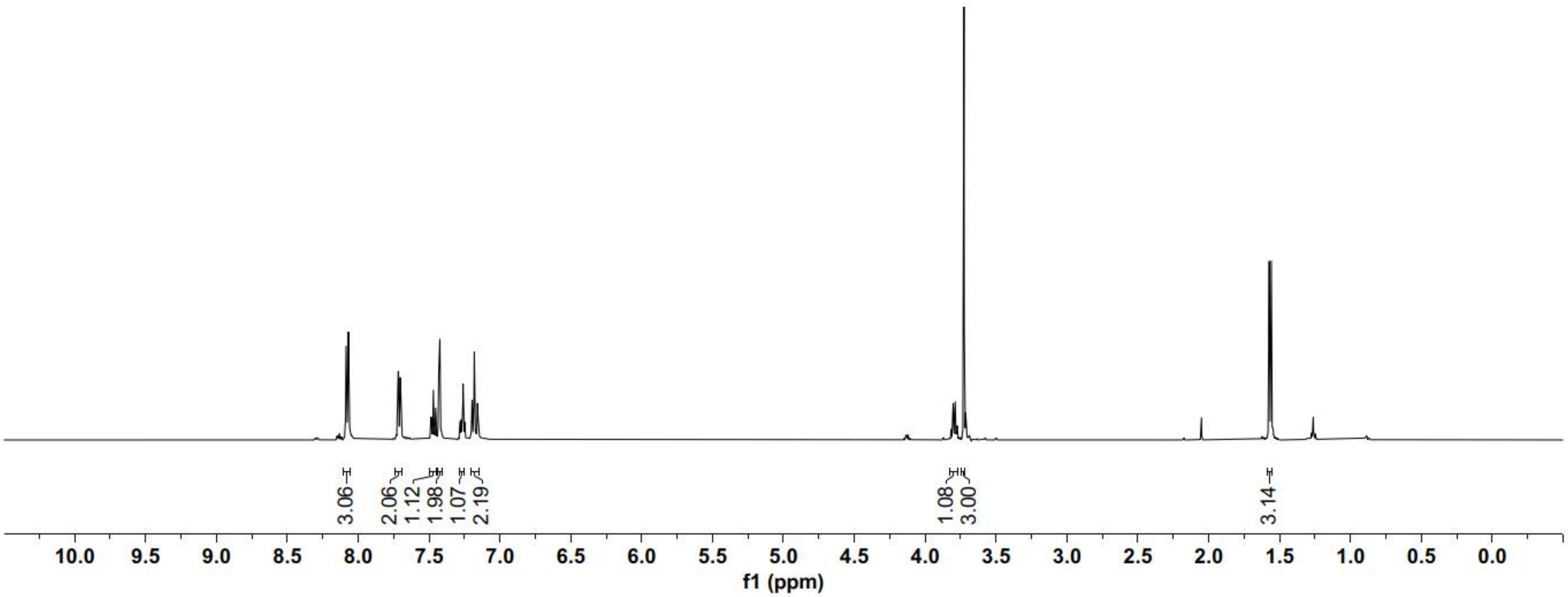

^{13}C NMR of 16

$CDCl_3$, 126 MHz, 25 °C

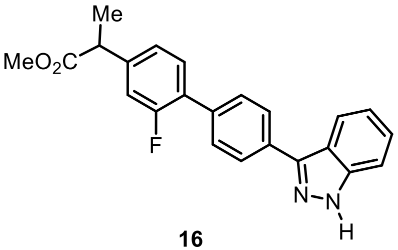

16

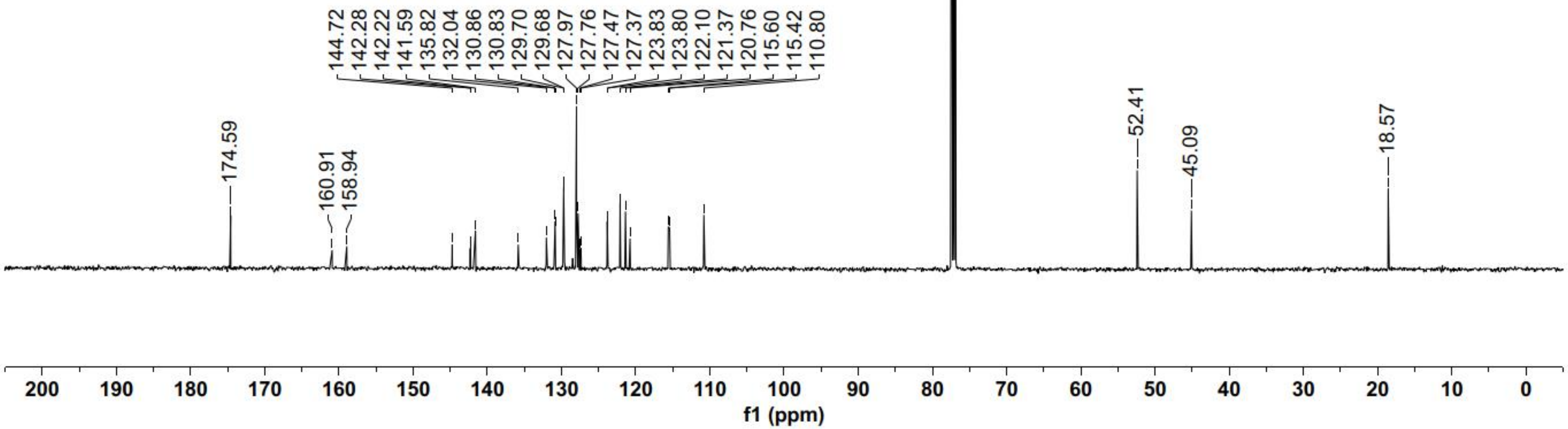

^{19}F NMR of 16

$CDCl_3$, 471 MHz, 25 °C

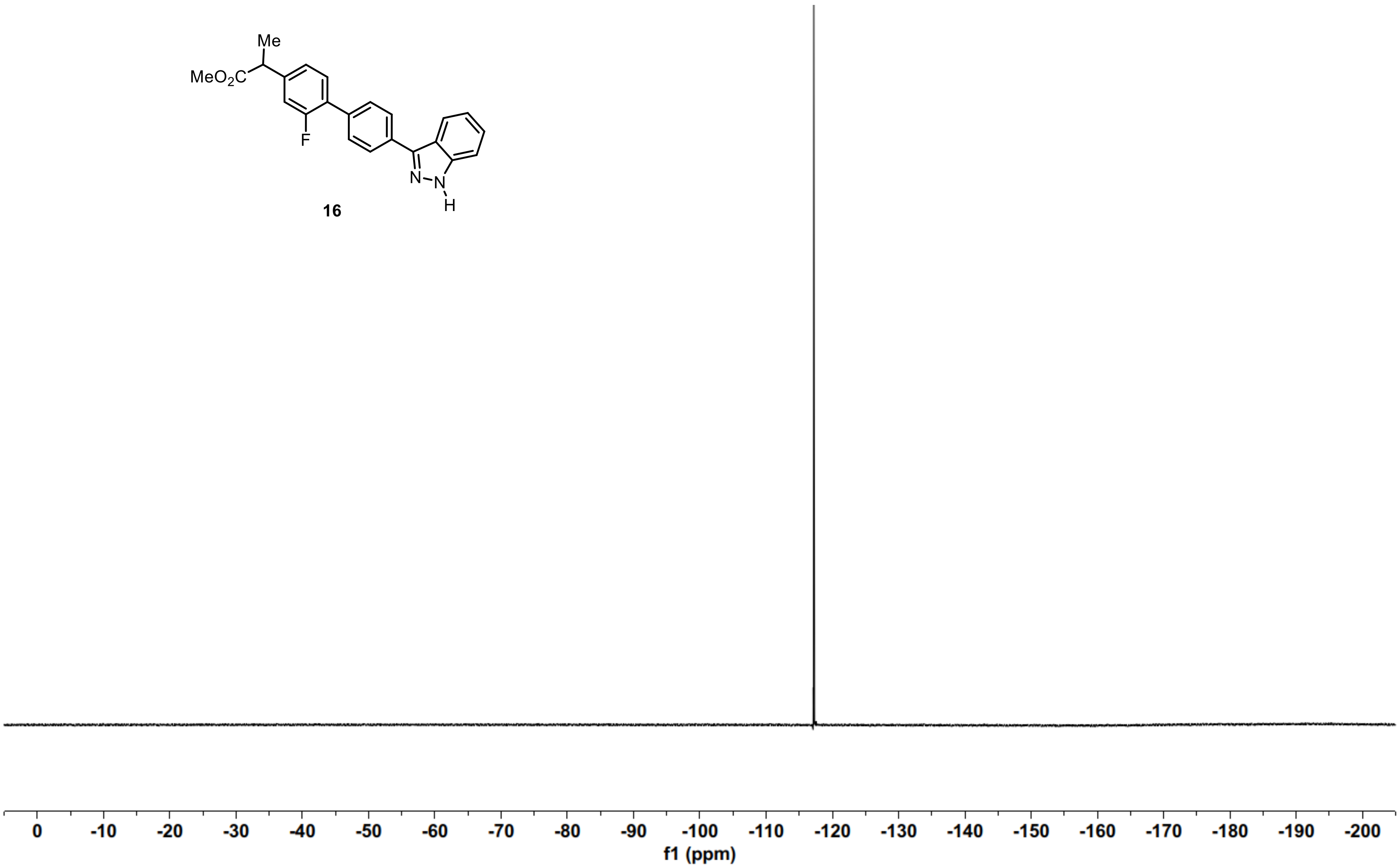

^{1}H NMR of 17

CDCl$_3$, 500 MHz, 25 °C

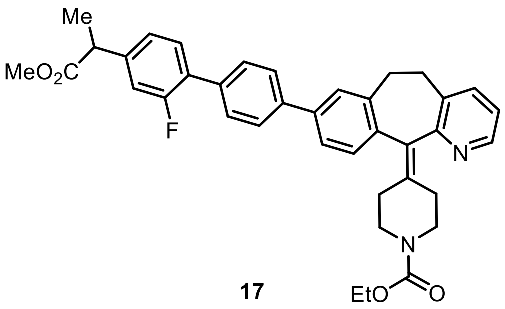

17

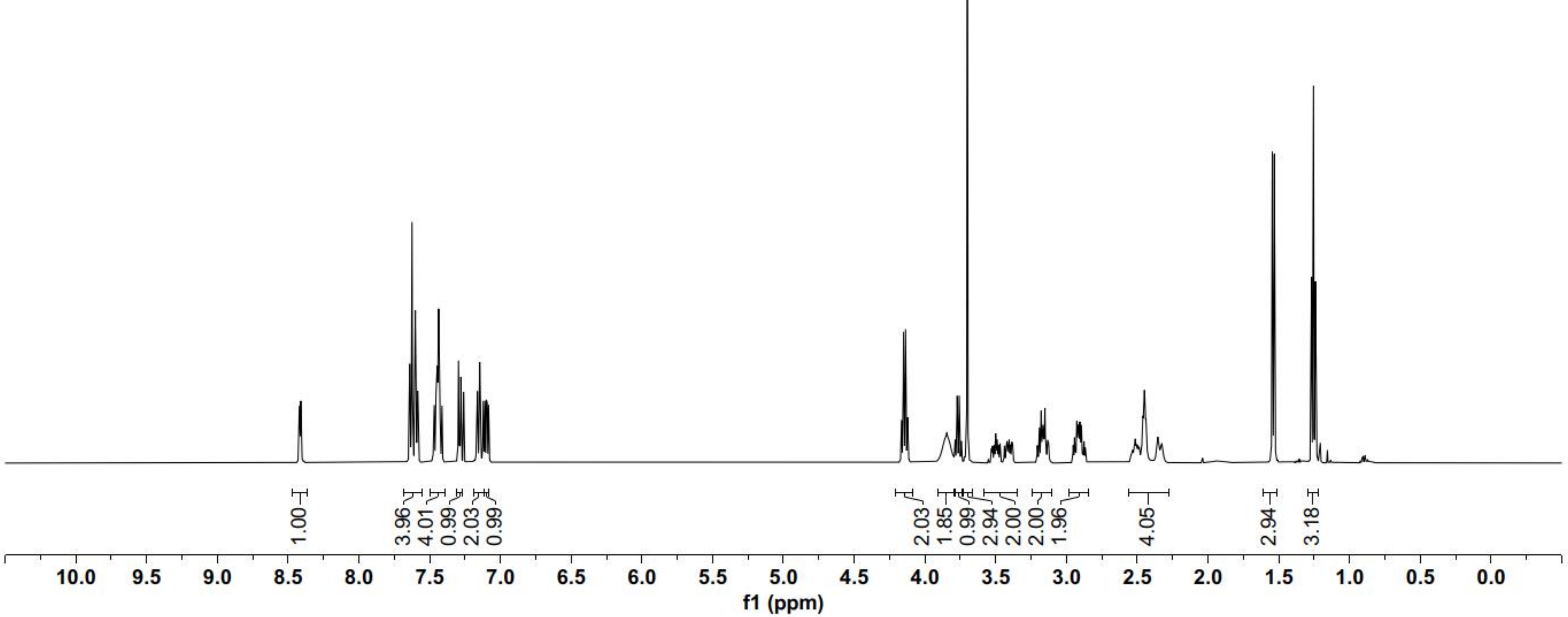

^{13}C NMR of 17

CDCl$_3$, 126 MHz, 25 °C

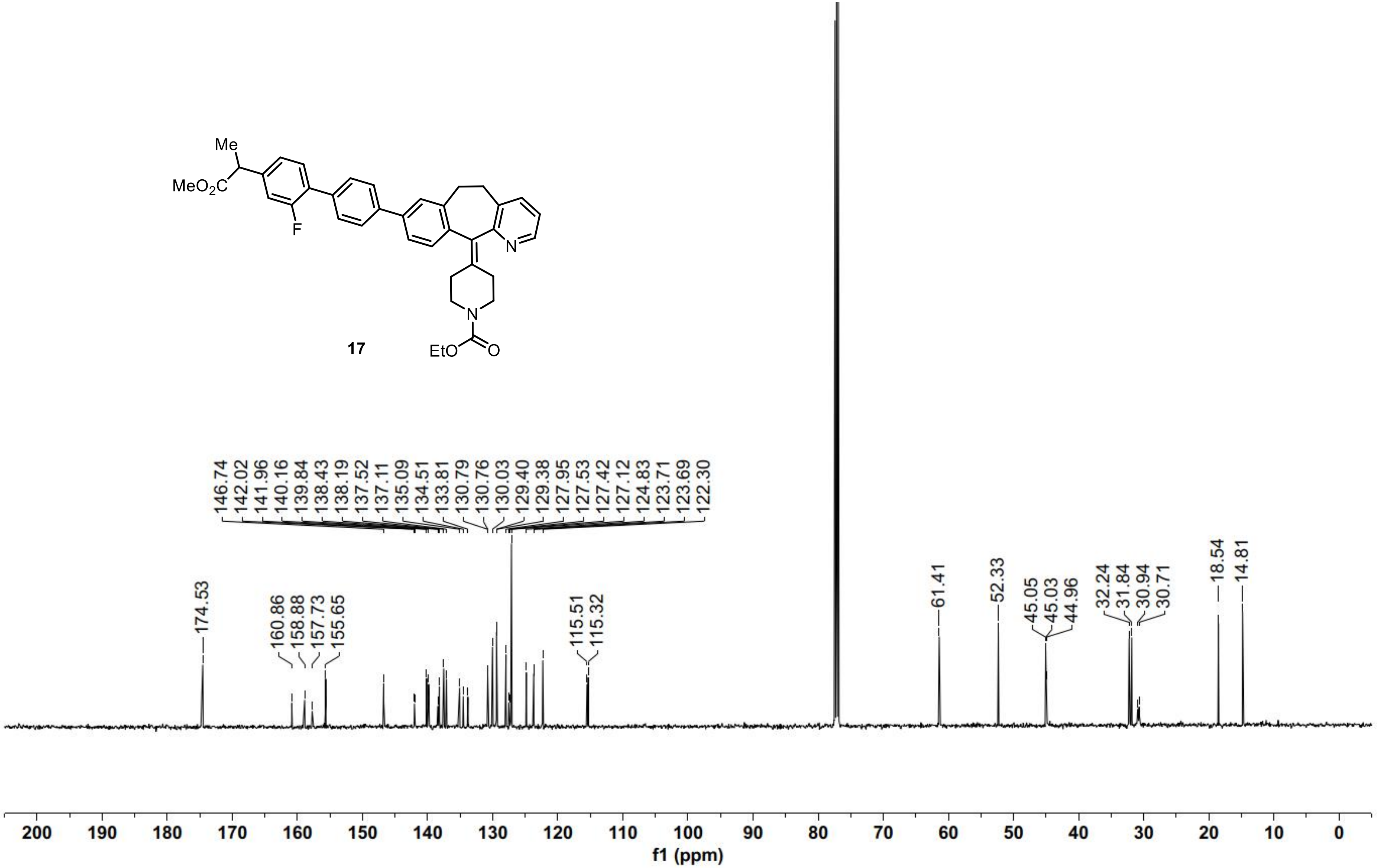

^{19}F NMR of 17

$CDCl_3$, 471 MHz, 25 °C

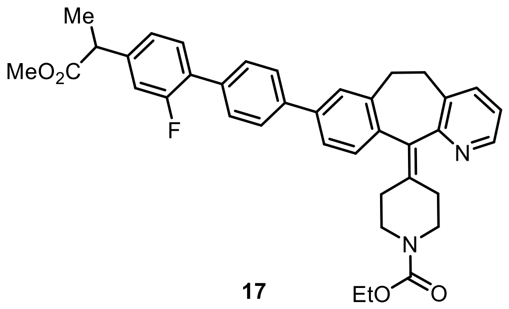

17

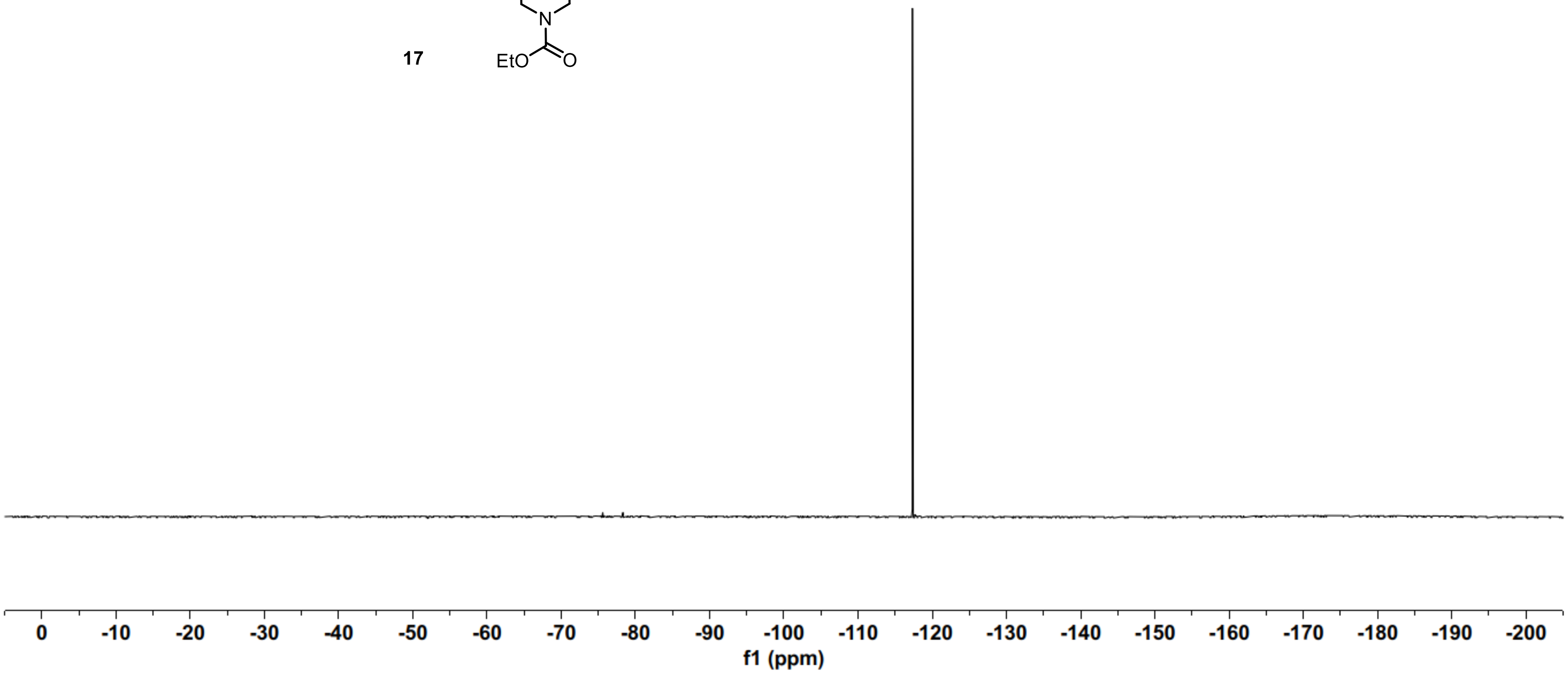

^{1}H NMR of 18

CDCl$_3$, 500 MHz, 25 °C

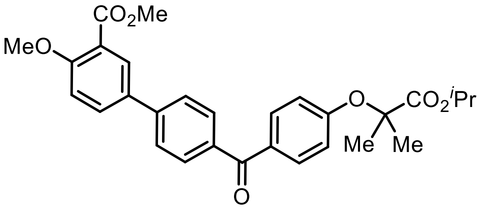

18

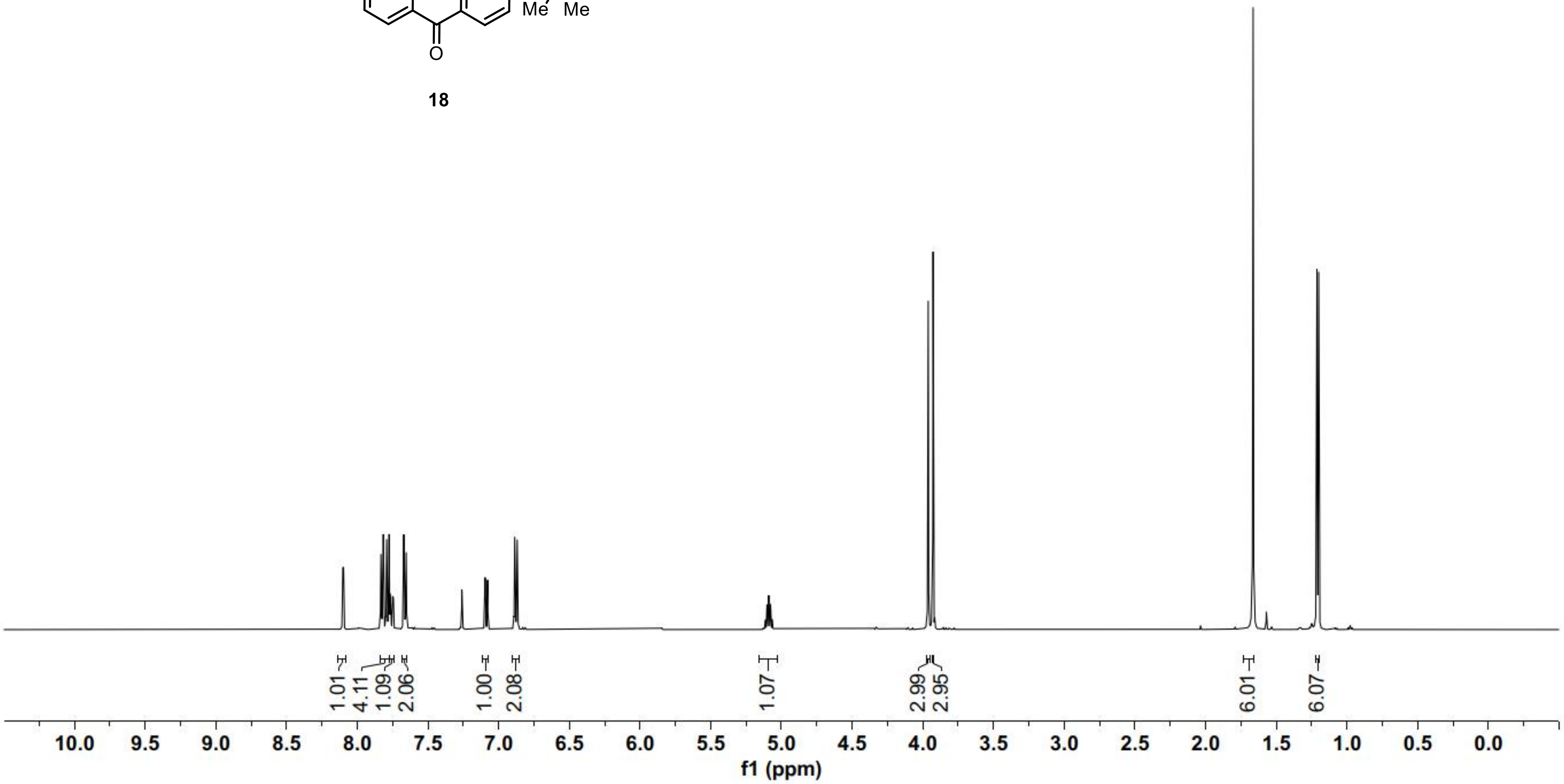

^{13}C NMR of 18

$CDCl_3$, 126 MHz, 25 °C

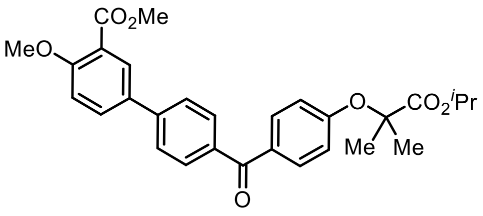

18

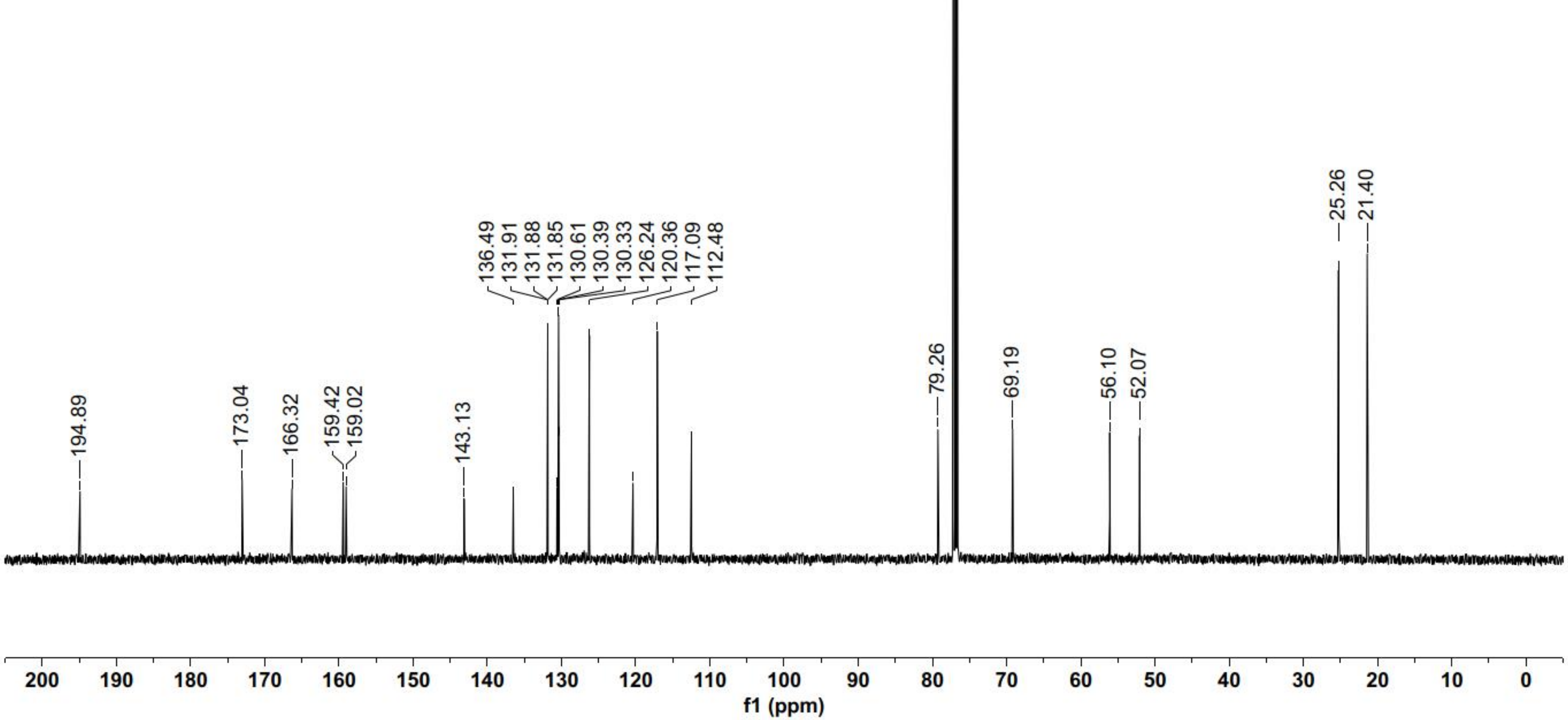

^{1}H NMR of 19

CDCl₃, 500 MHz, 25 °C

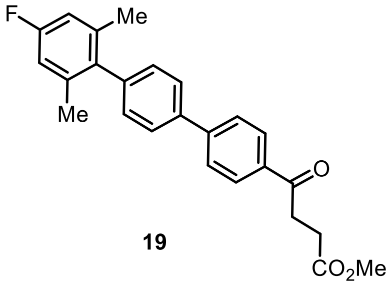

19

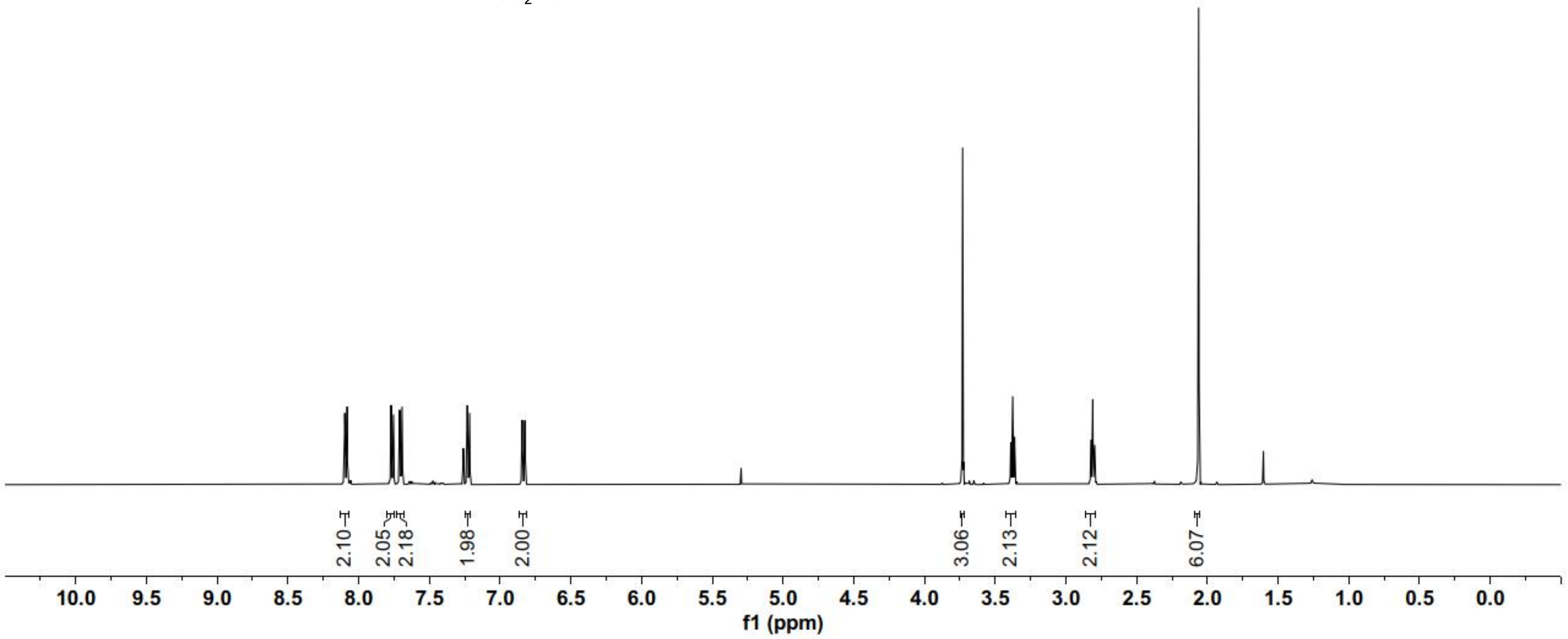

^{13}C NMR of 19

$CDCl_3$, 126 MHz, 25 °C

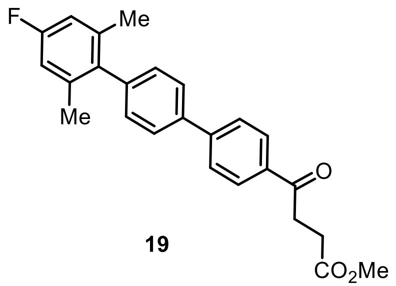

19

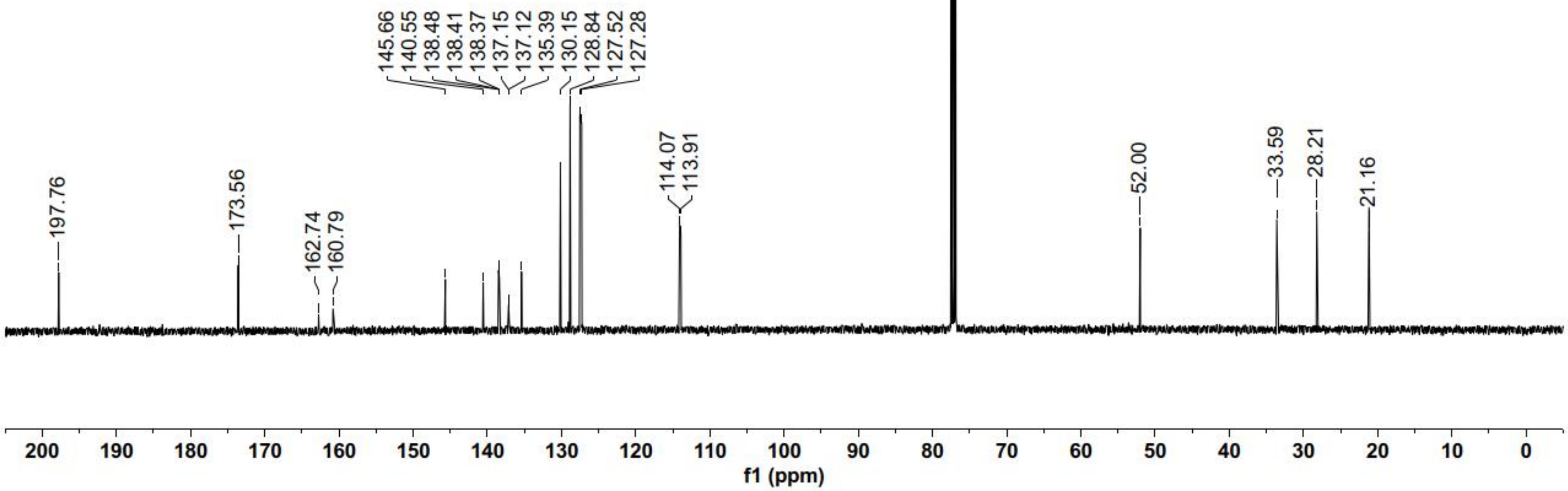

^{19}F NMR of 19

$CDCl_3$, 471 MHz, 25 °C

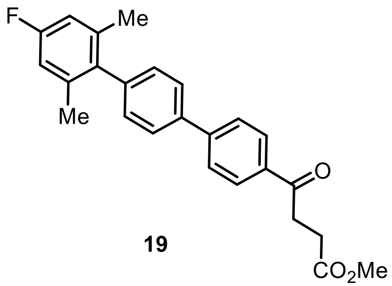

19

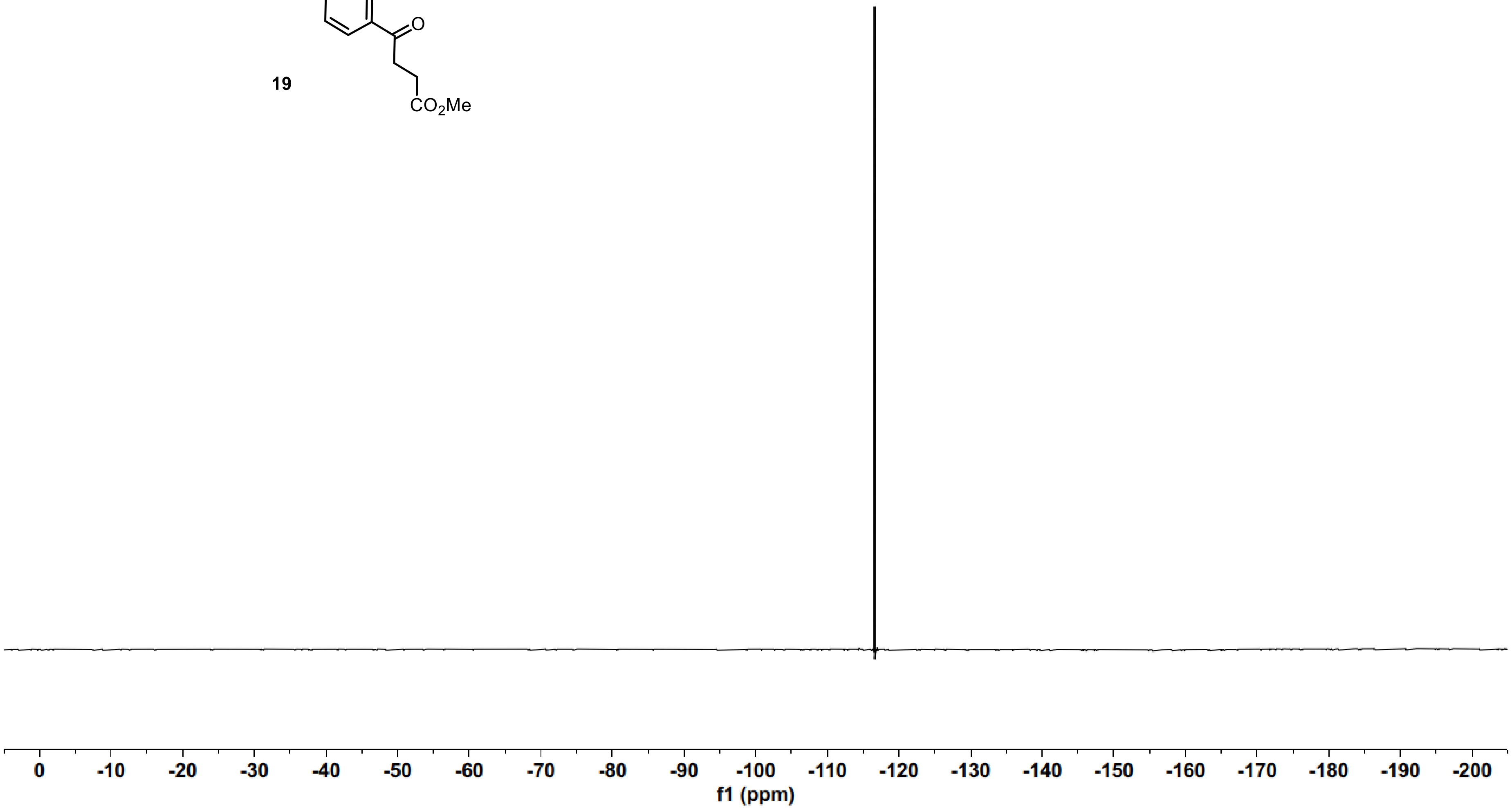

^{1}H NMR of 20

CDCl$_3$, 500 MHz, 25 °C

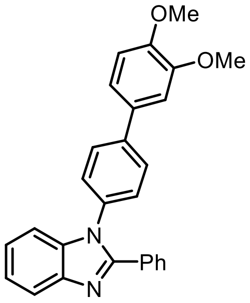

20

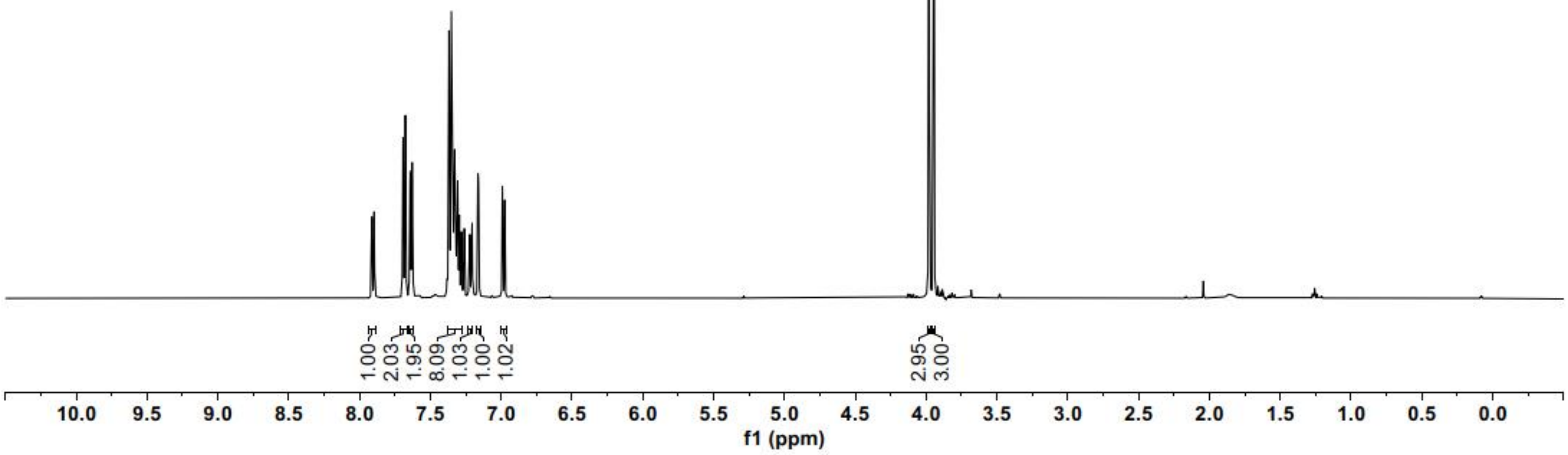

^{13}C NMR of 20

$CDCl_3$, 126 MHz, 25 °C

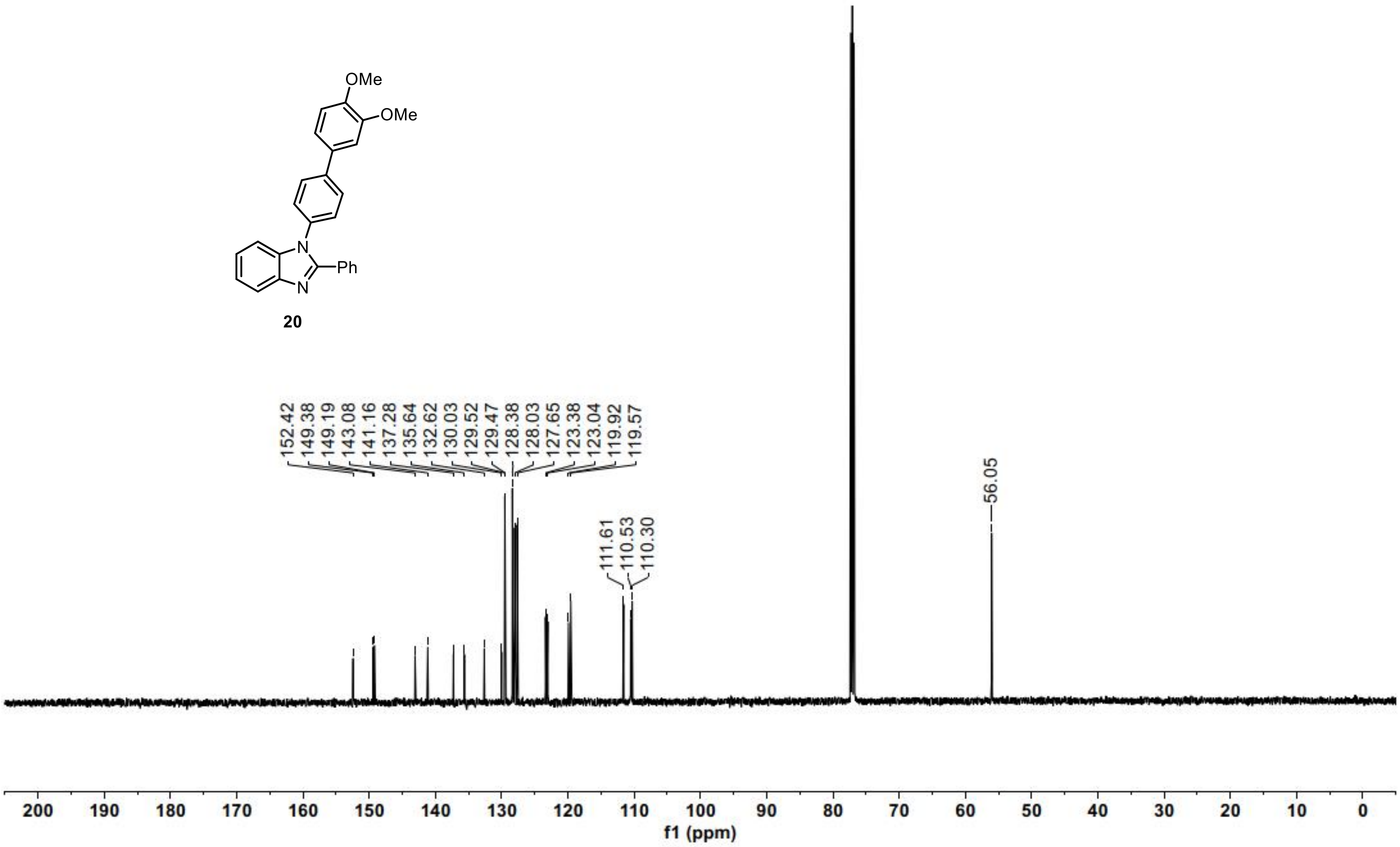

^{1}H NMR of 21

$CDCl_3$, 500 MHz, 25 °C

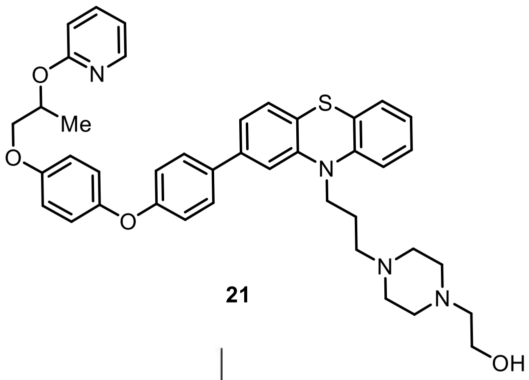

21

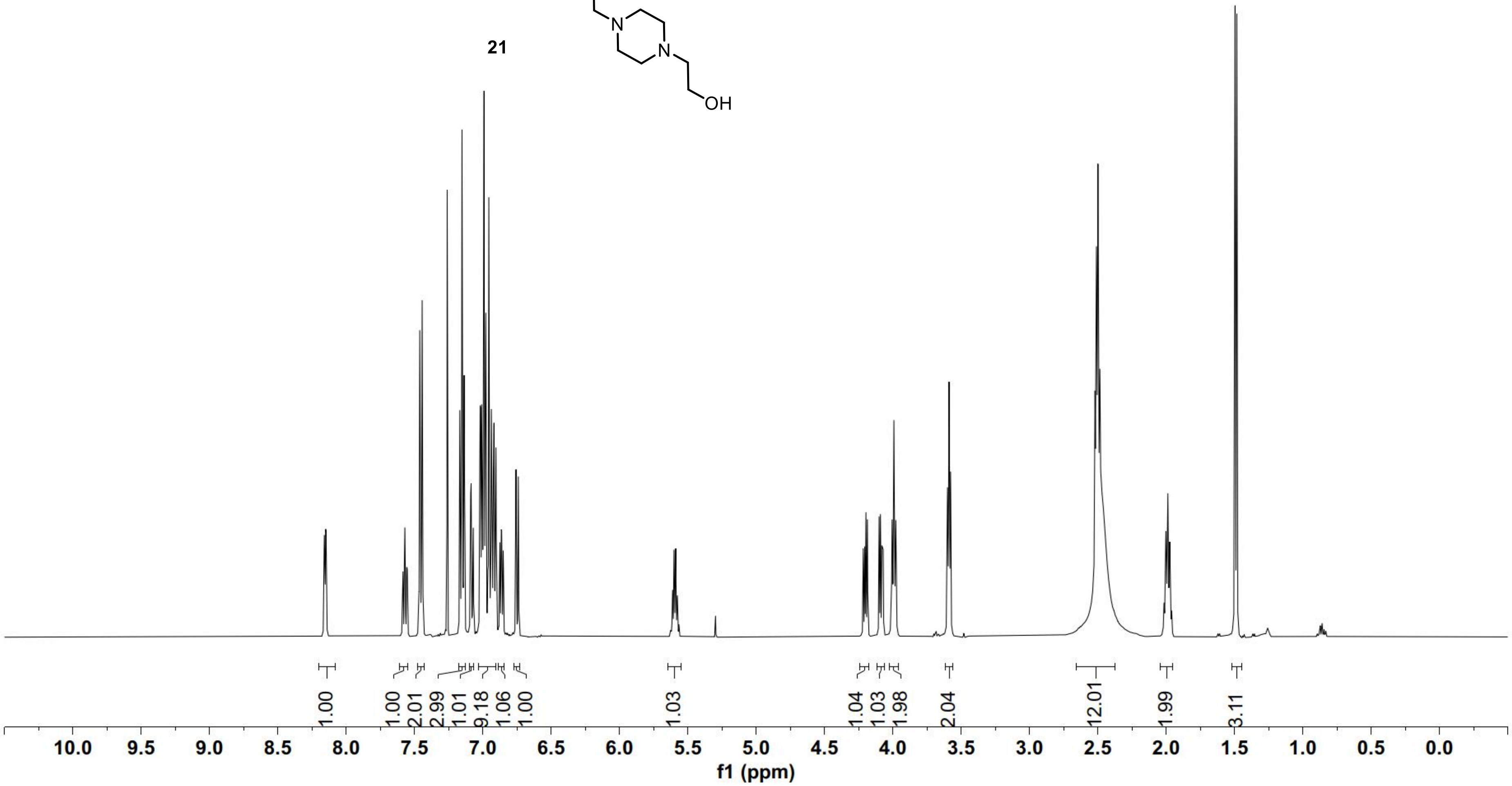

^{13}C NMR of 21

$CDCl_3$, 126 MHz, 25 °C

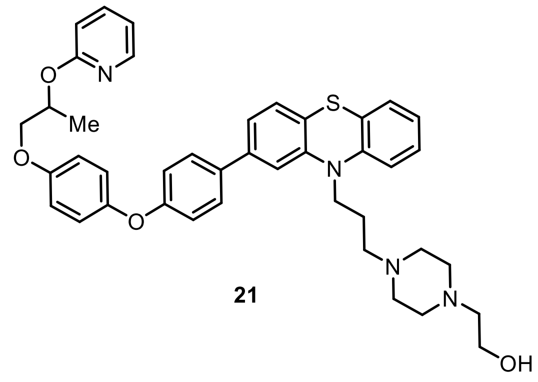

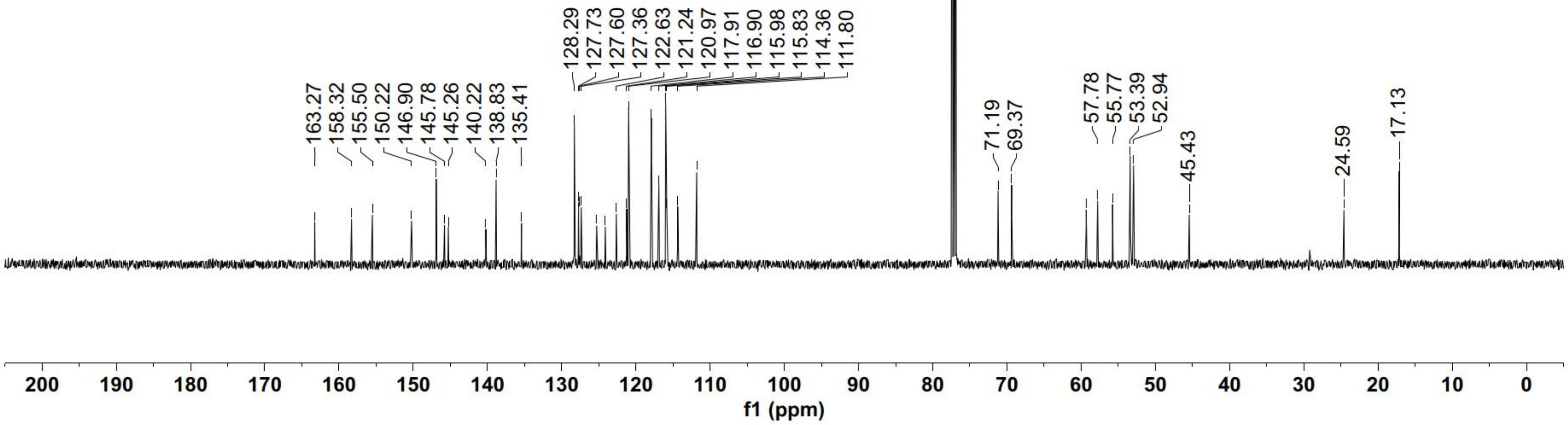

^{1}H NMR of 22

CDCl$_3$, 500 MHz, 25 °C

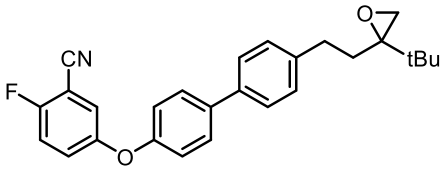

22

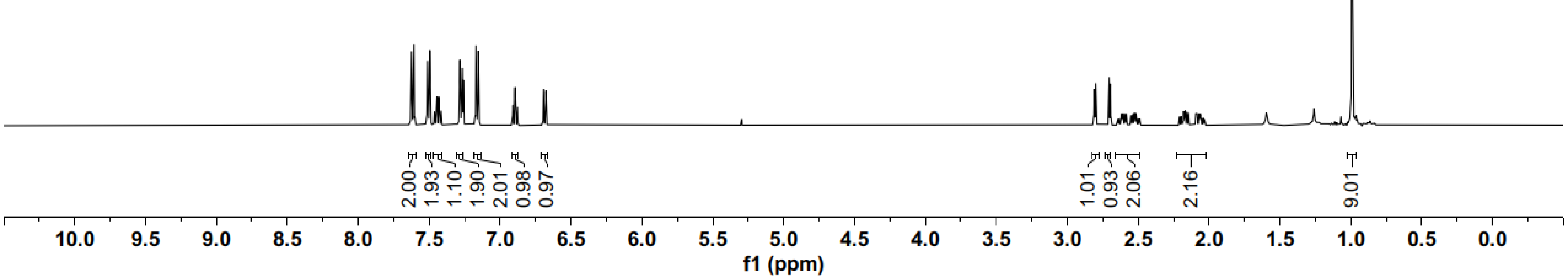

^{13}C NMR of 22

$CDCl_3$, 126 MHz, 25 °C

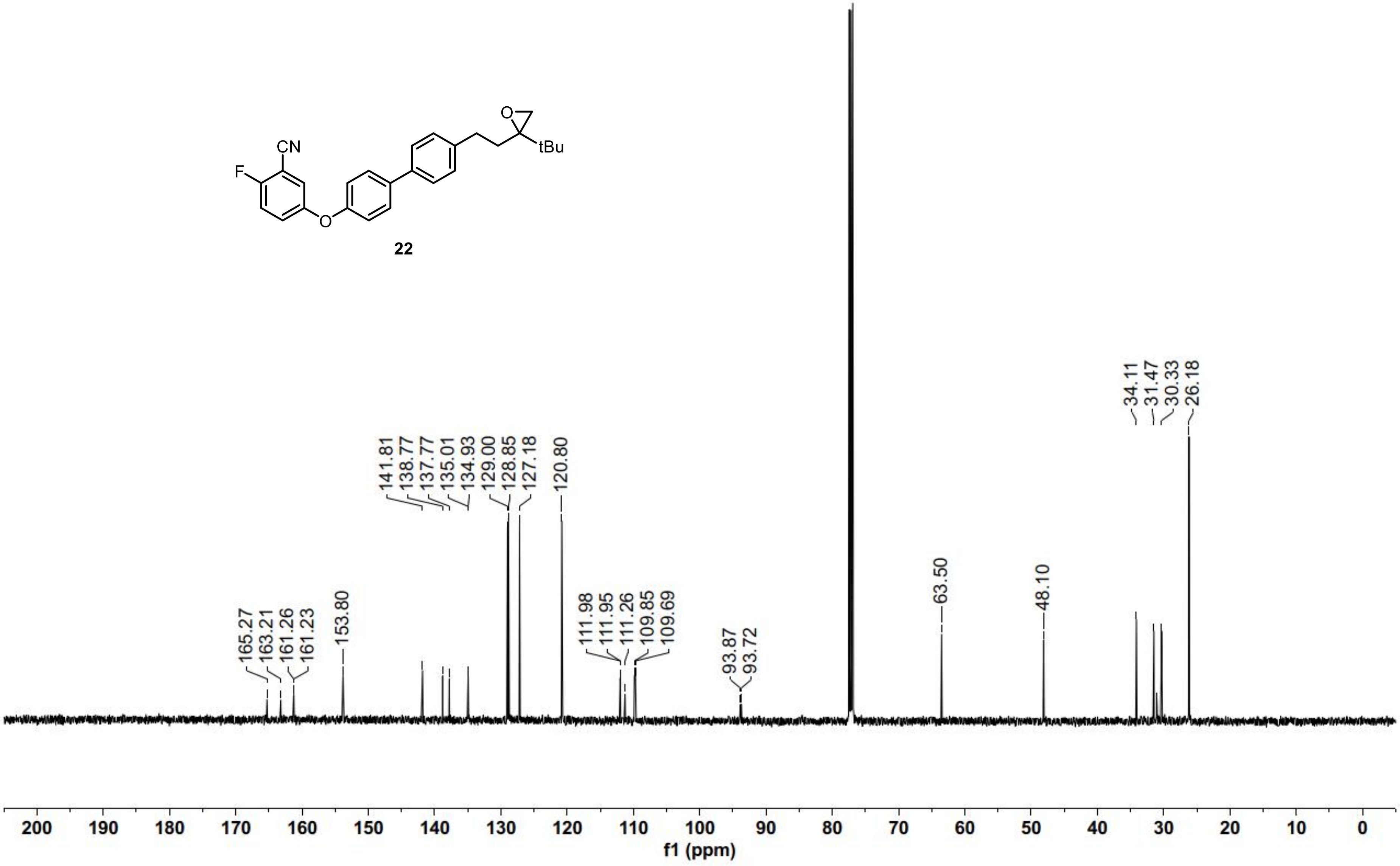

^{19}F NMR of 22

$CDCl_3$, 471 MHz, 25 °C

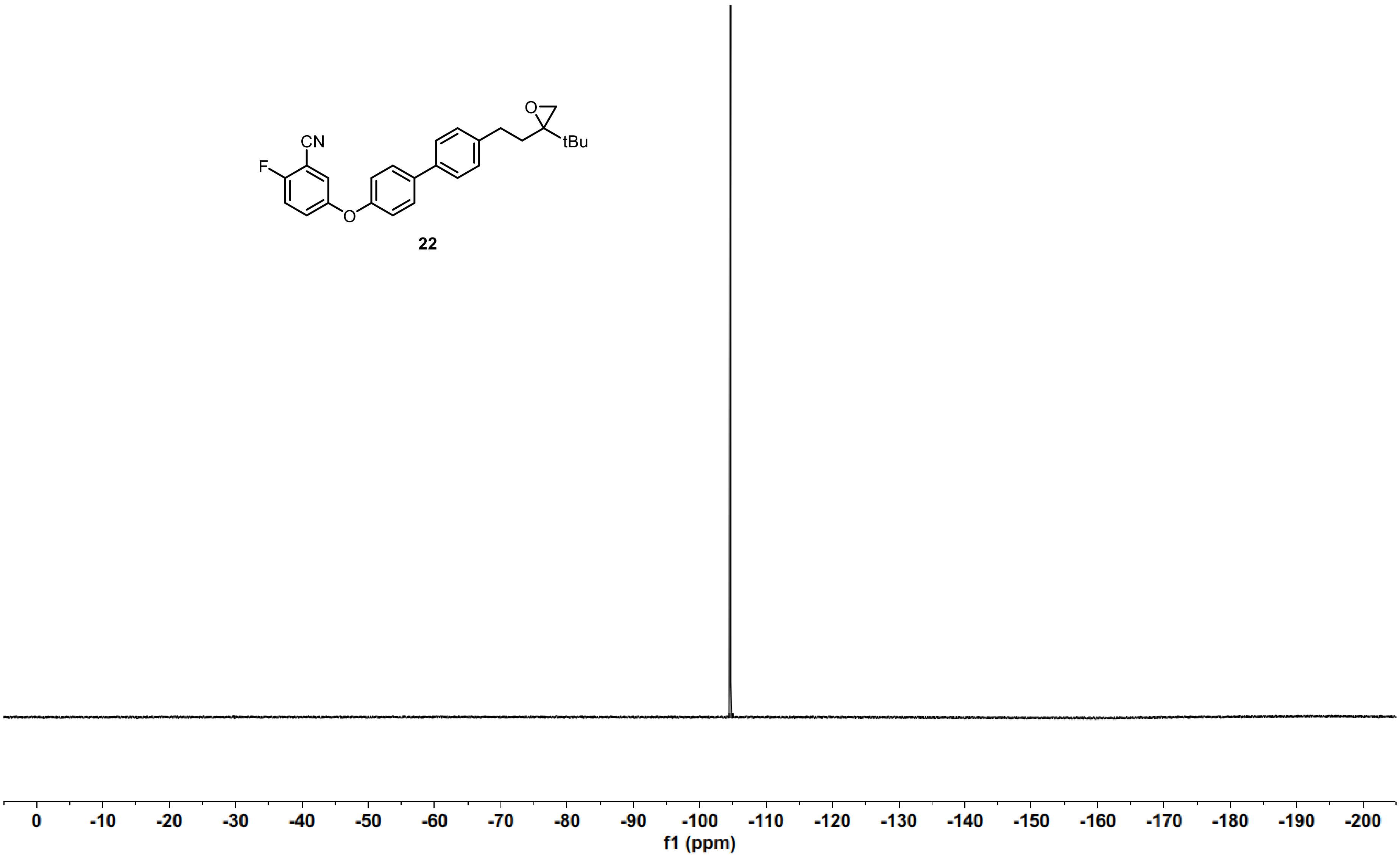

^{1}H NMR of 23

$CDCl_3$, 500 MHz, 25 °C

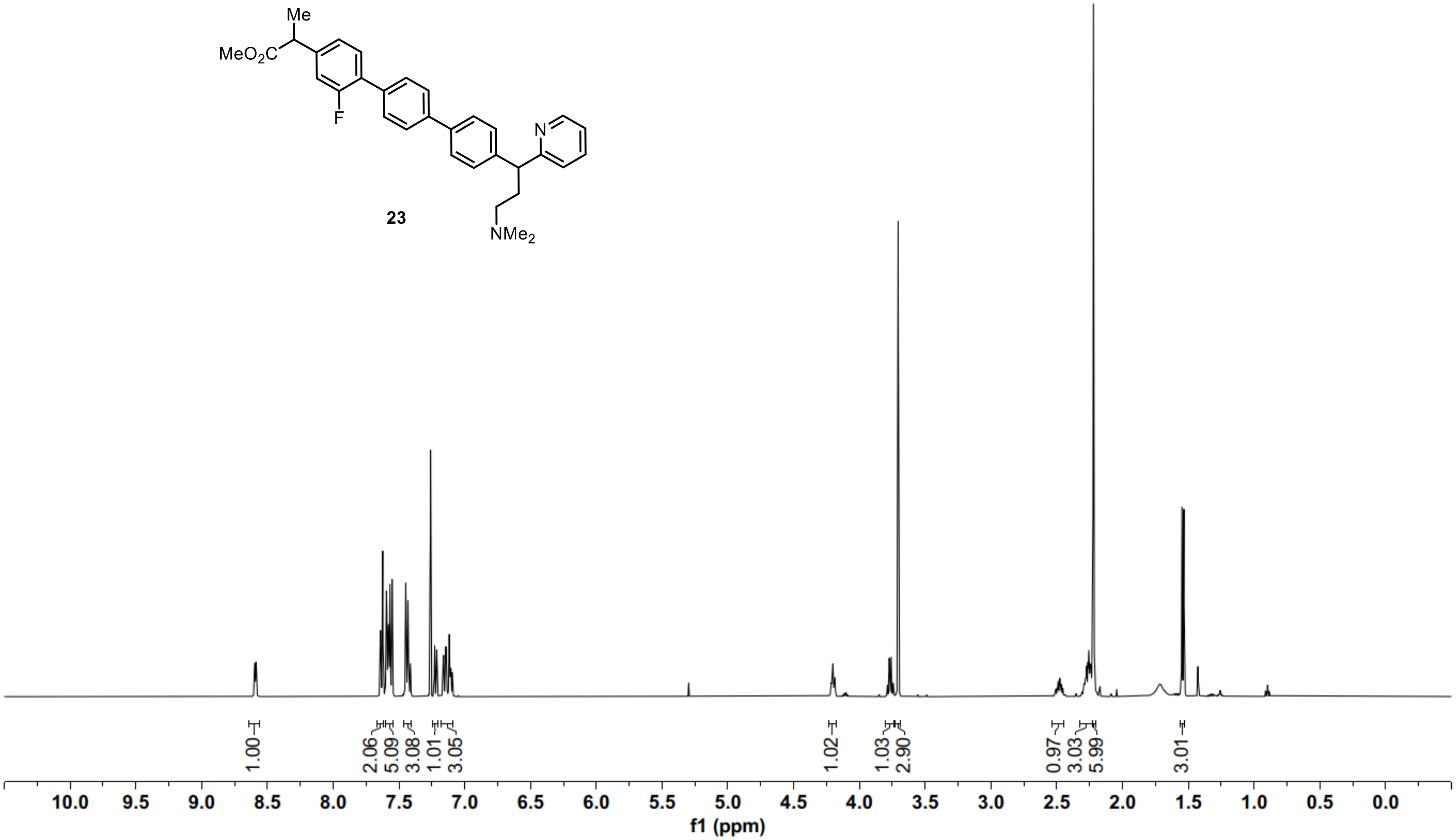

^{13}C NMR of 23

$CDCl_3$, 126 MHz, 25 °C

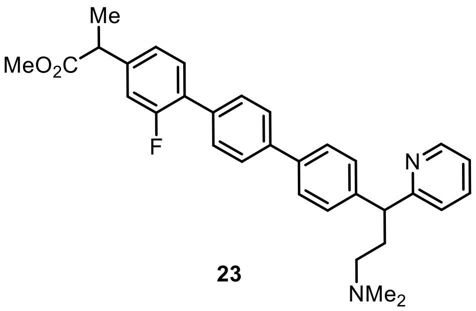

23

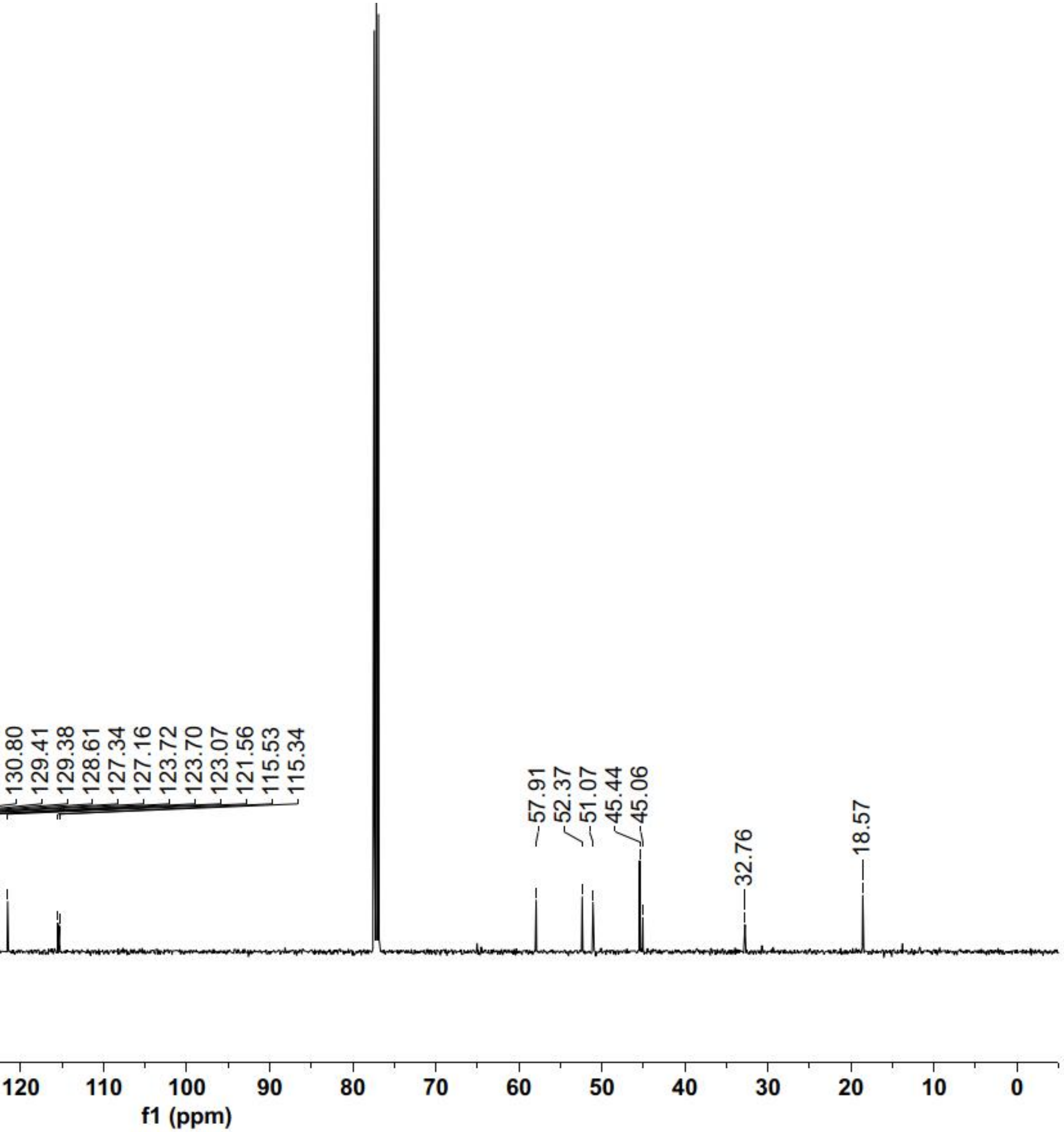

^{19}F NMR of 23

$CDCl_3$, 471 MHz, 25 °C

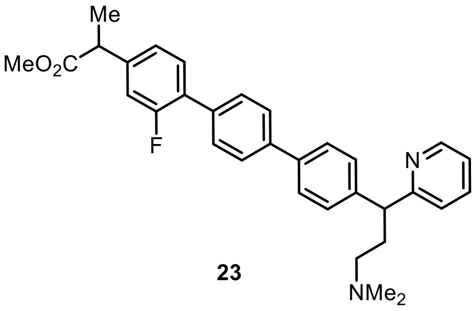

23

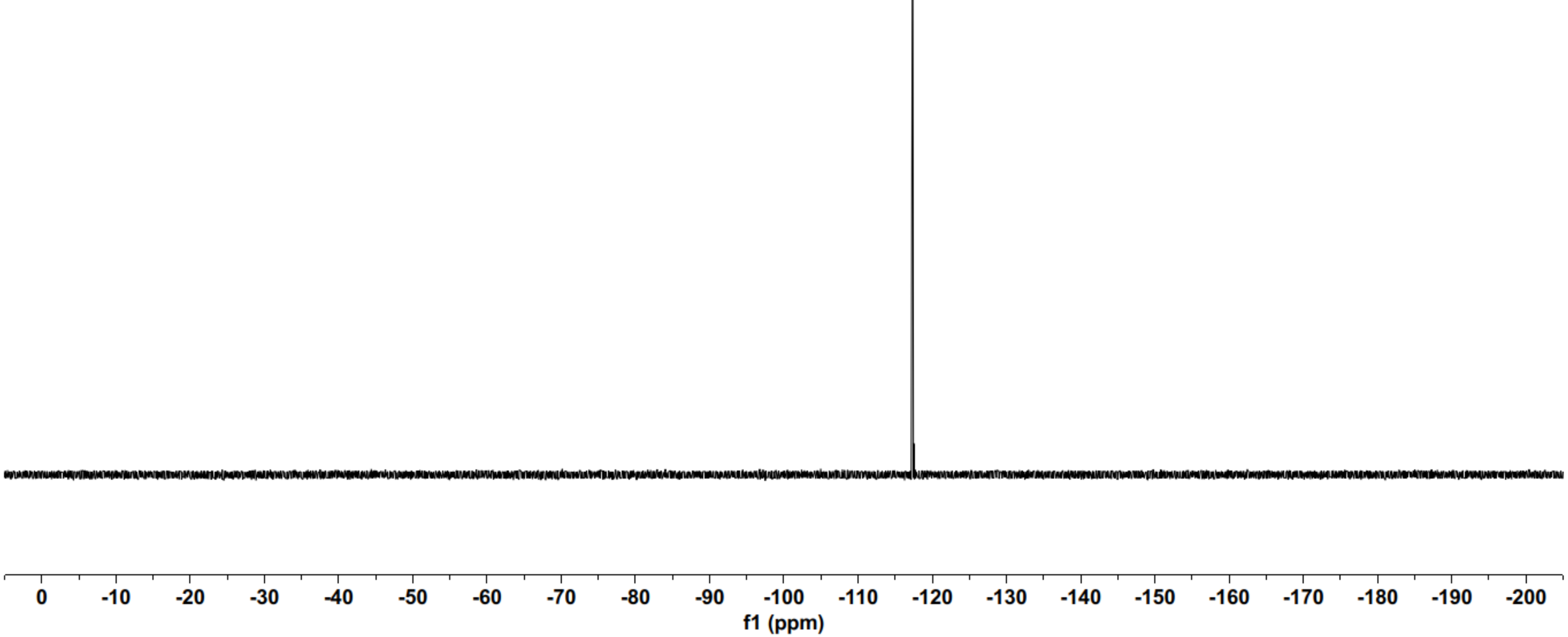

^{1}H NMR of 24

$CDCl_3$, 500 MHz, 25 °C

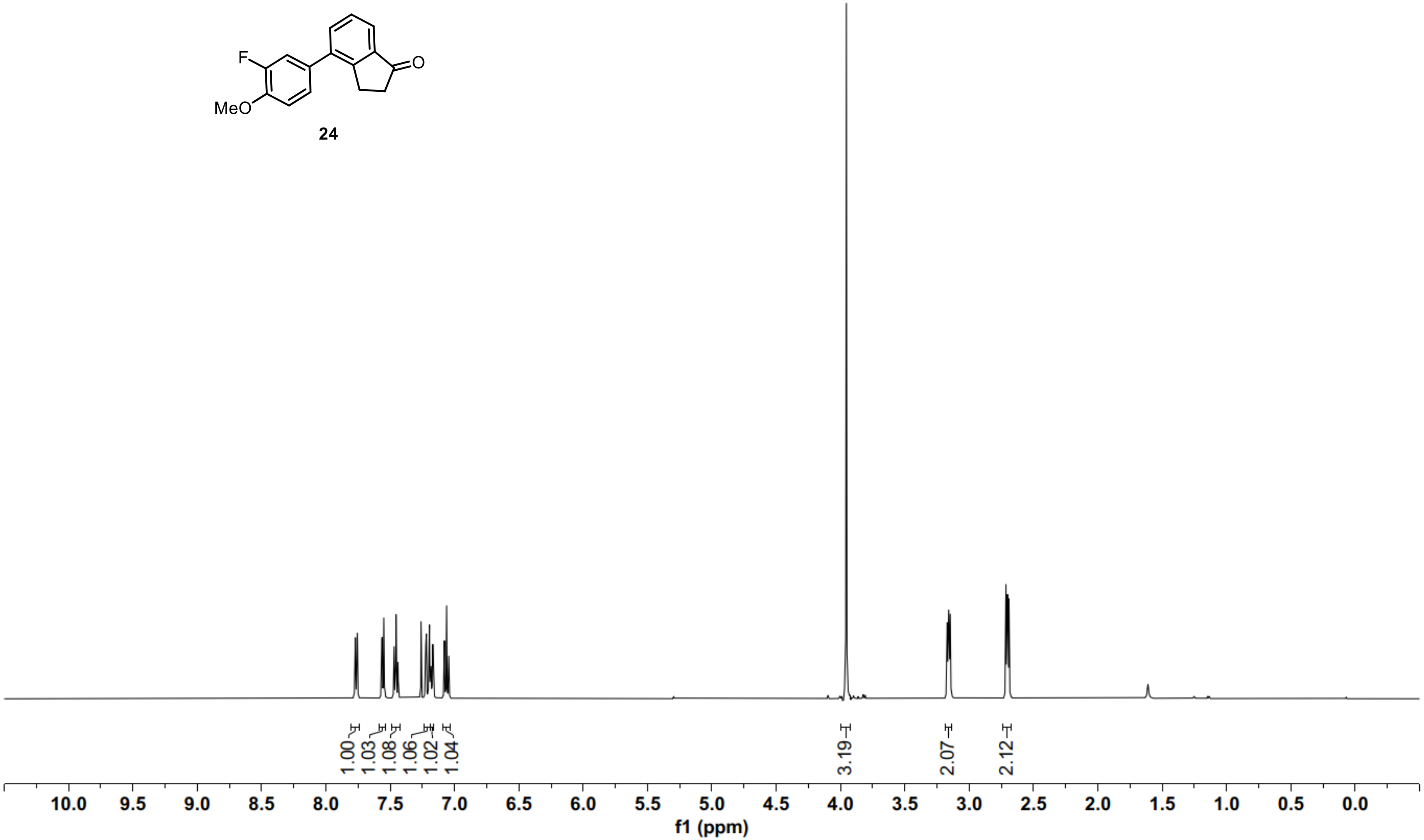

^{13}C NMR of 24

CDCl$_3$, 126 MHz, 25 °C

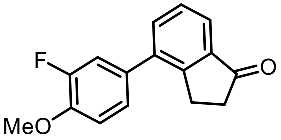

24

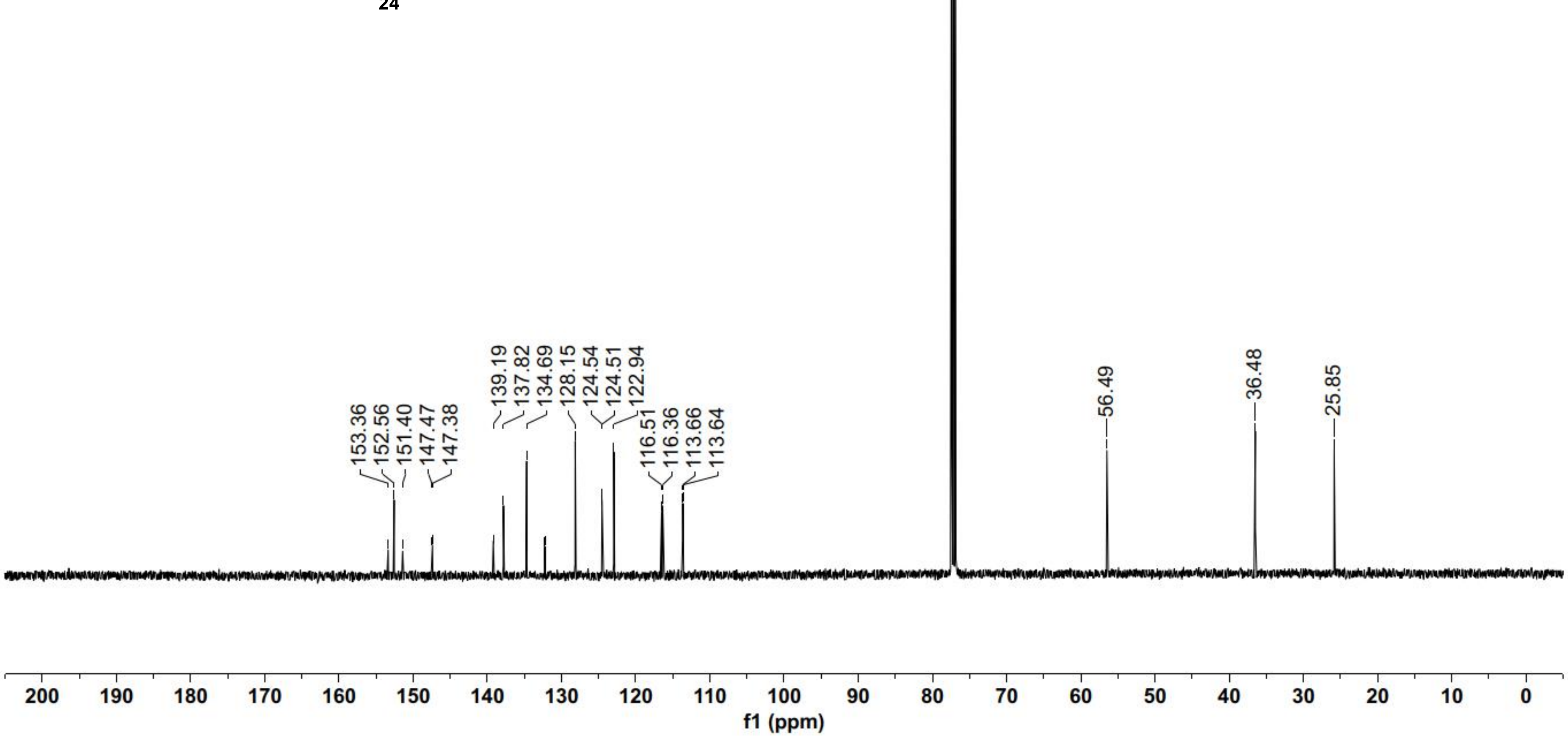

^{19}F NMR of 24

$CDCl_3$, 471 MHz, 25 °C

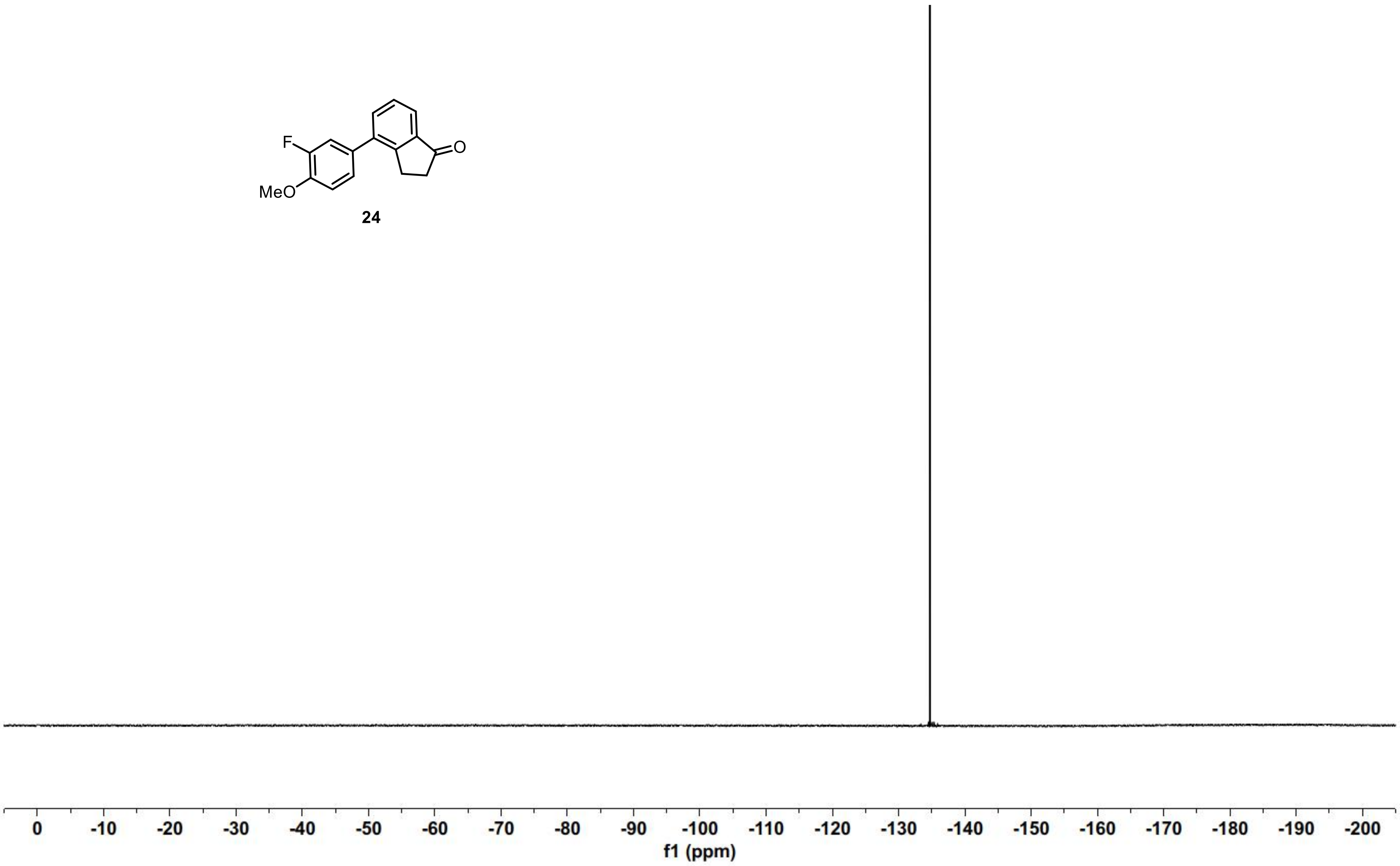

^{1}H NMR of 25

$CDCl_3$, 500 MHz, 25 °C

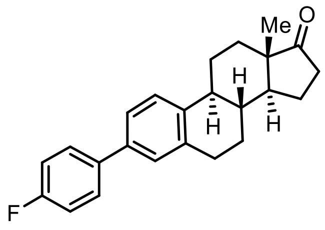

25

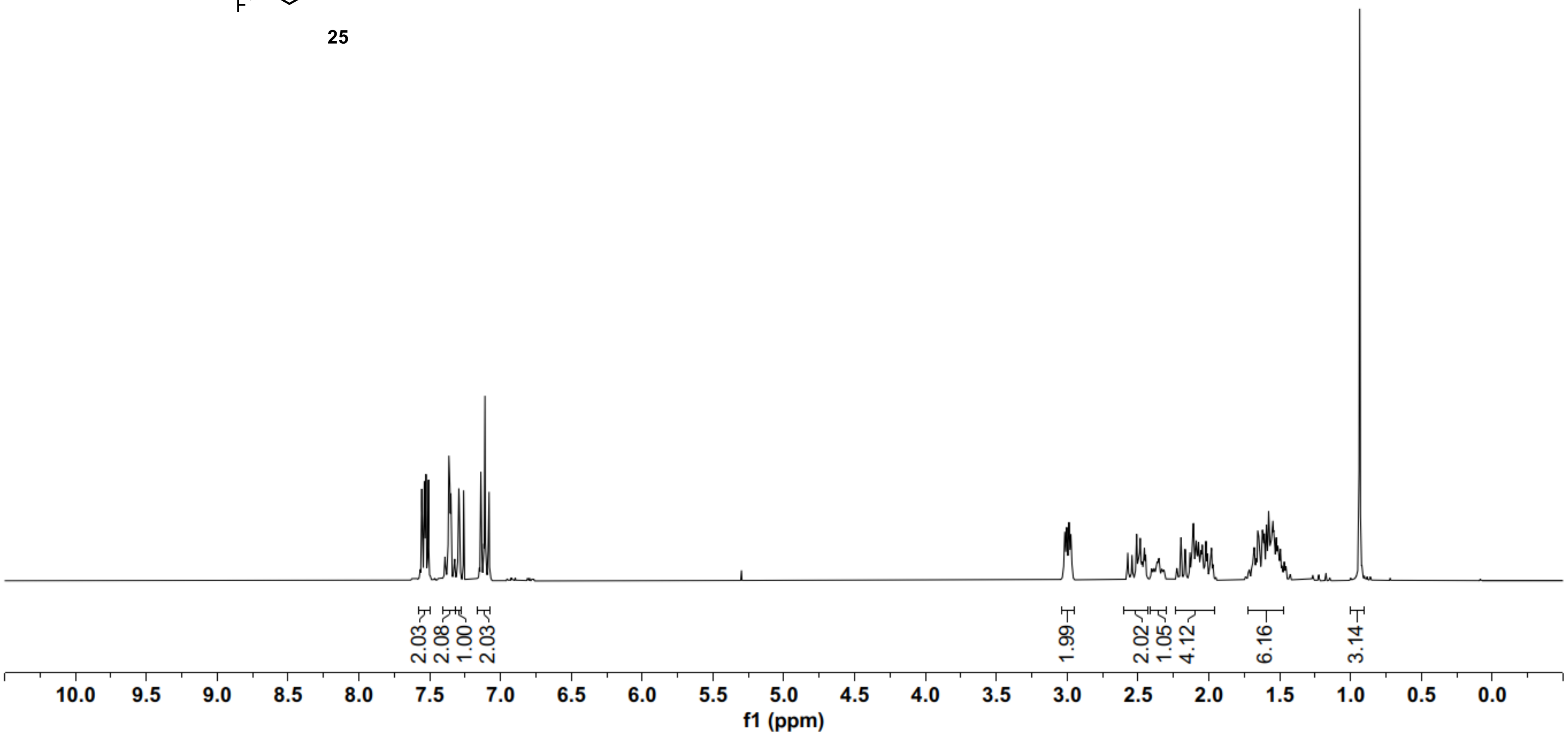

^{13}C NMR of 25

$CDCl_3$, 126 MHz, 25 °C

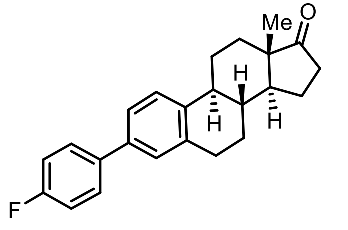

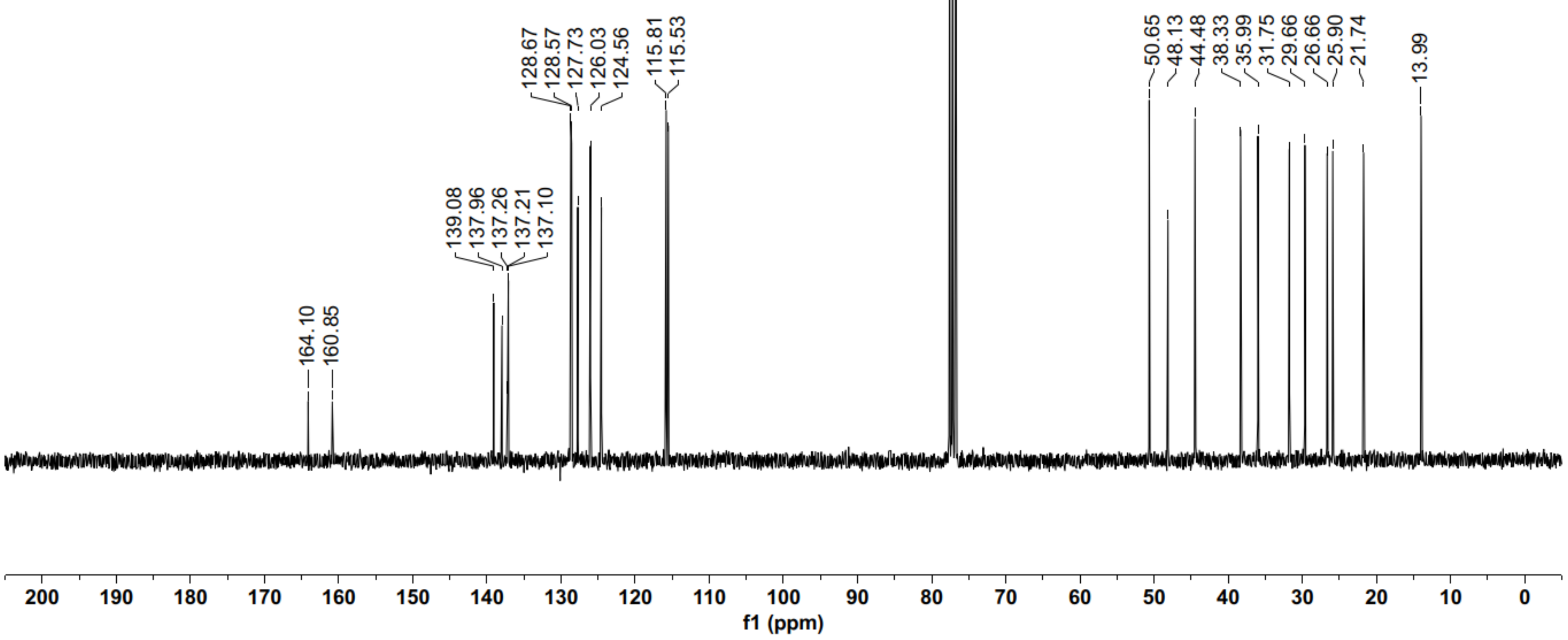

^{19}F NMR of 25

$CDCl_3$, 471 MHz, 25 °C

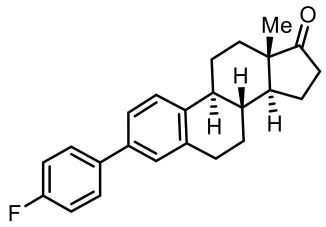

25

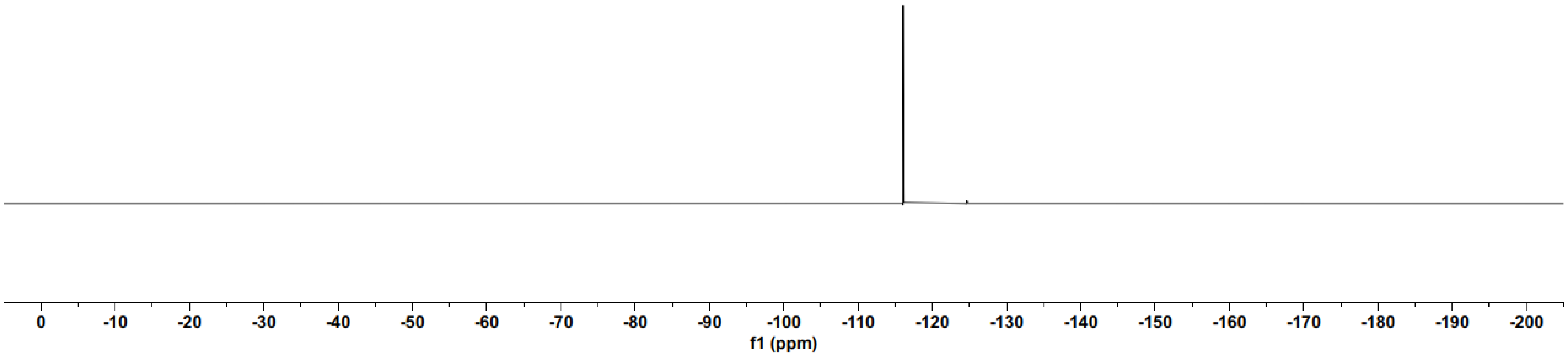

^{1}H NMR of 26

$CDCl_3$, 500 MHz, 25 °C

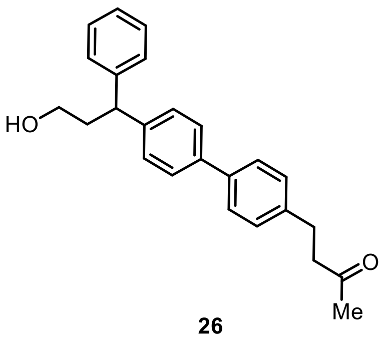

26

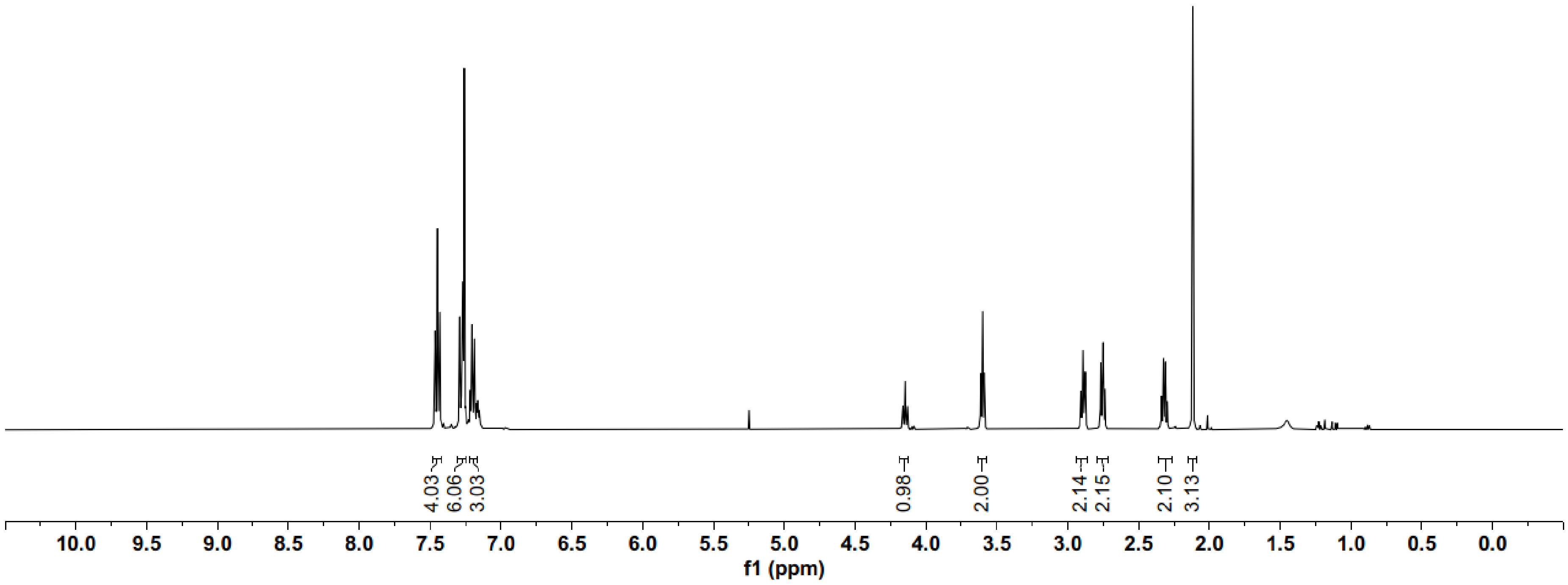

^{13}C NMR of 26

$CDCl_3$, 126 MHz, 25 °C

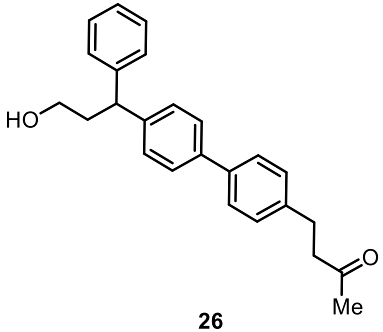

26

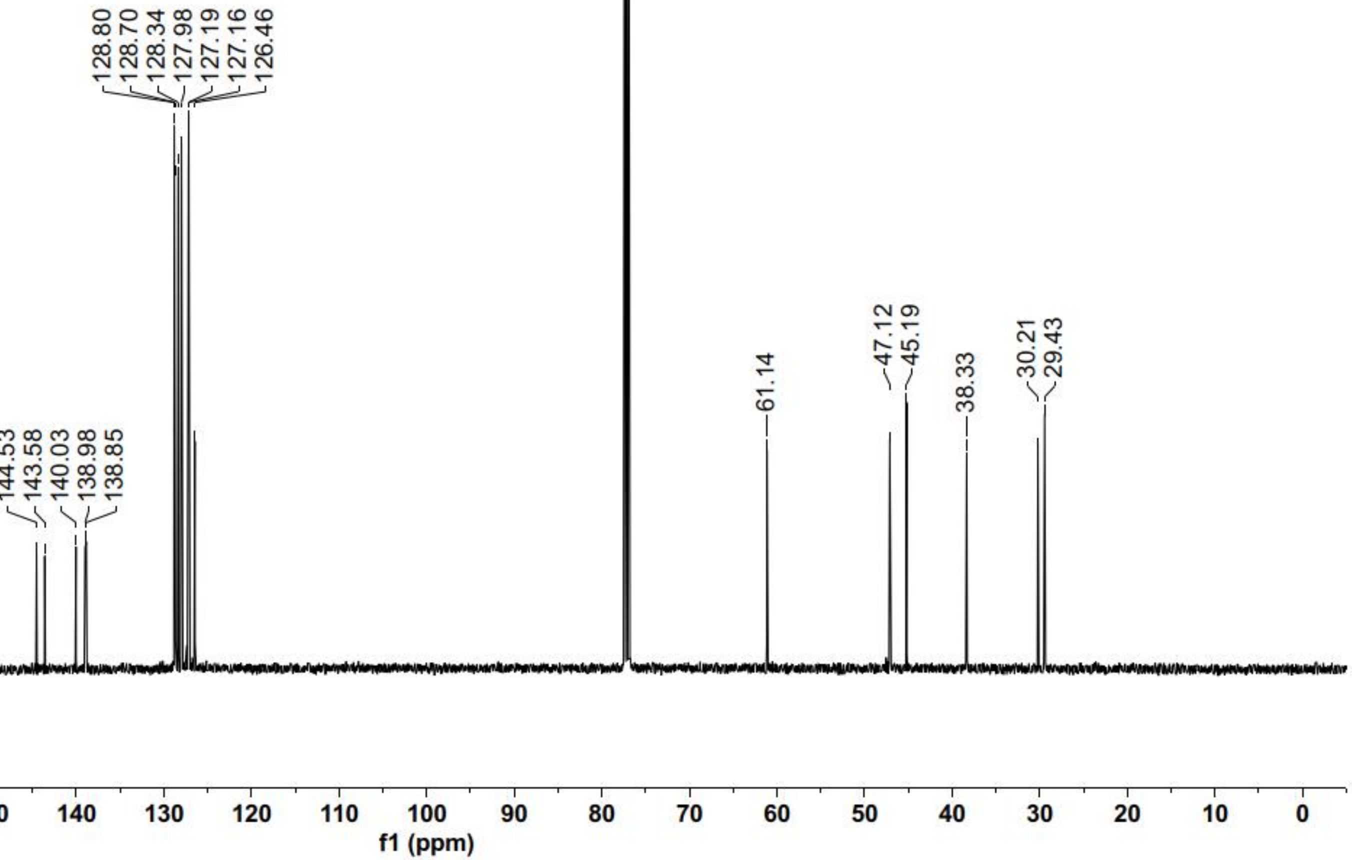

^{1}H NMR of 27

CDCl₃, 500 MHz, 25 °C

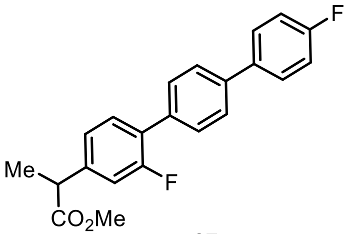

27

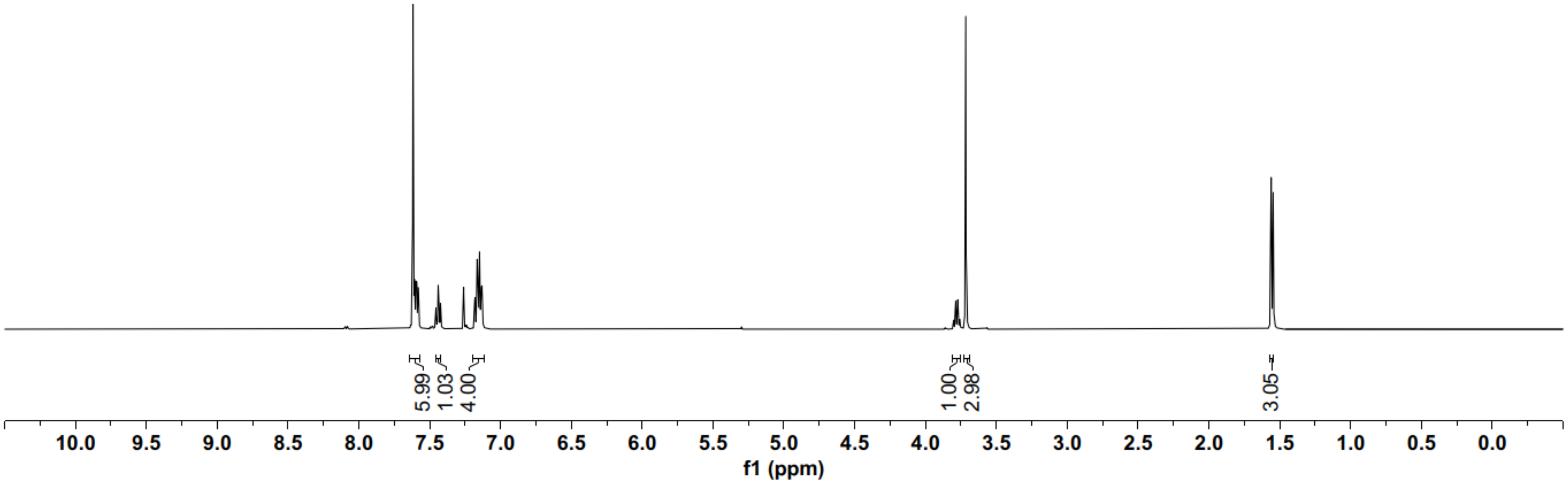

^{13}C NMR of 27

$CDCl_3$, 126 MHz, 25 °C

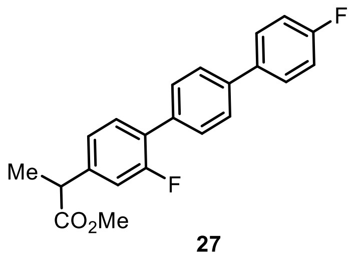

27

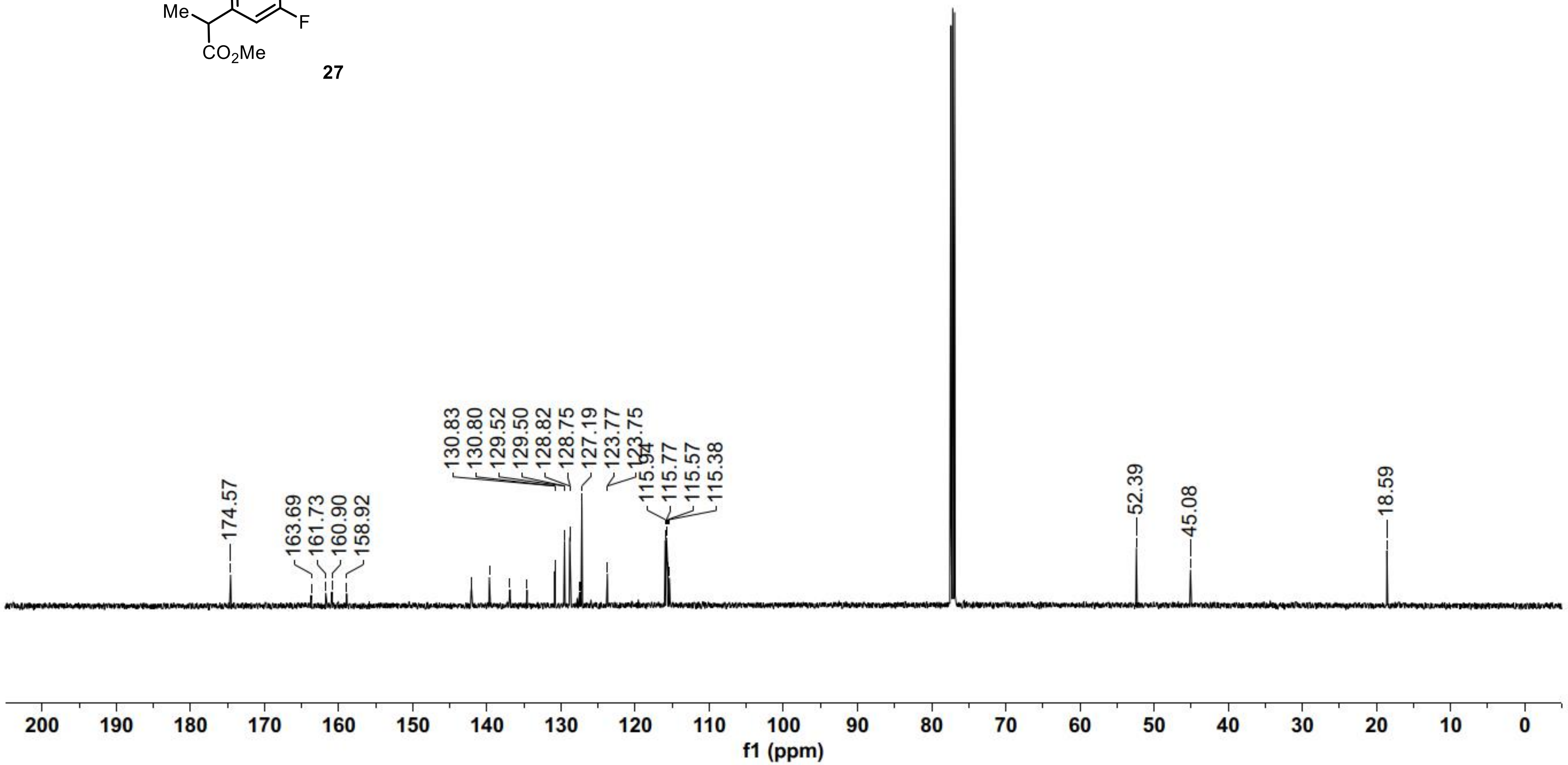

^{19}F NMR of 27

$CDCl_3$, 471 MHz, 25 °C

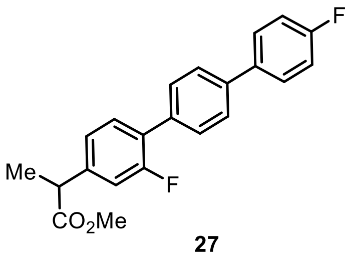

27

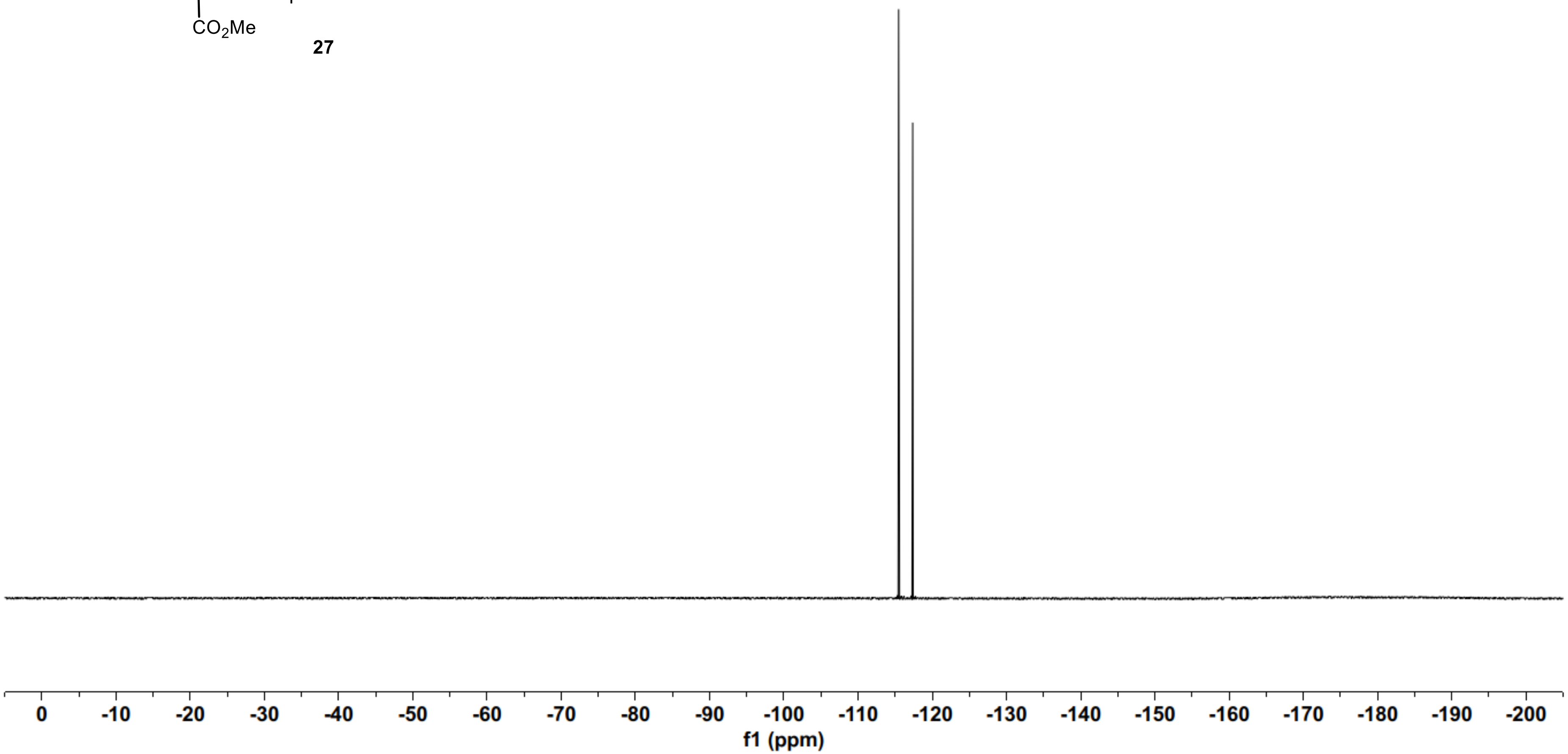

^{1}H NMR of 28

$CDCl_3$, 500 MHz, 25 °C

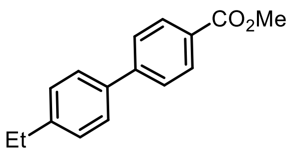

28

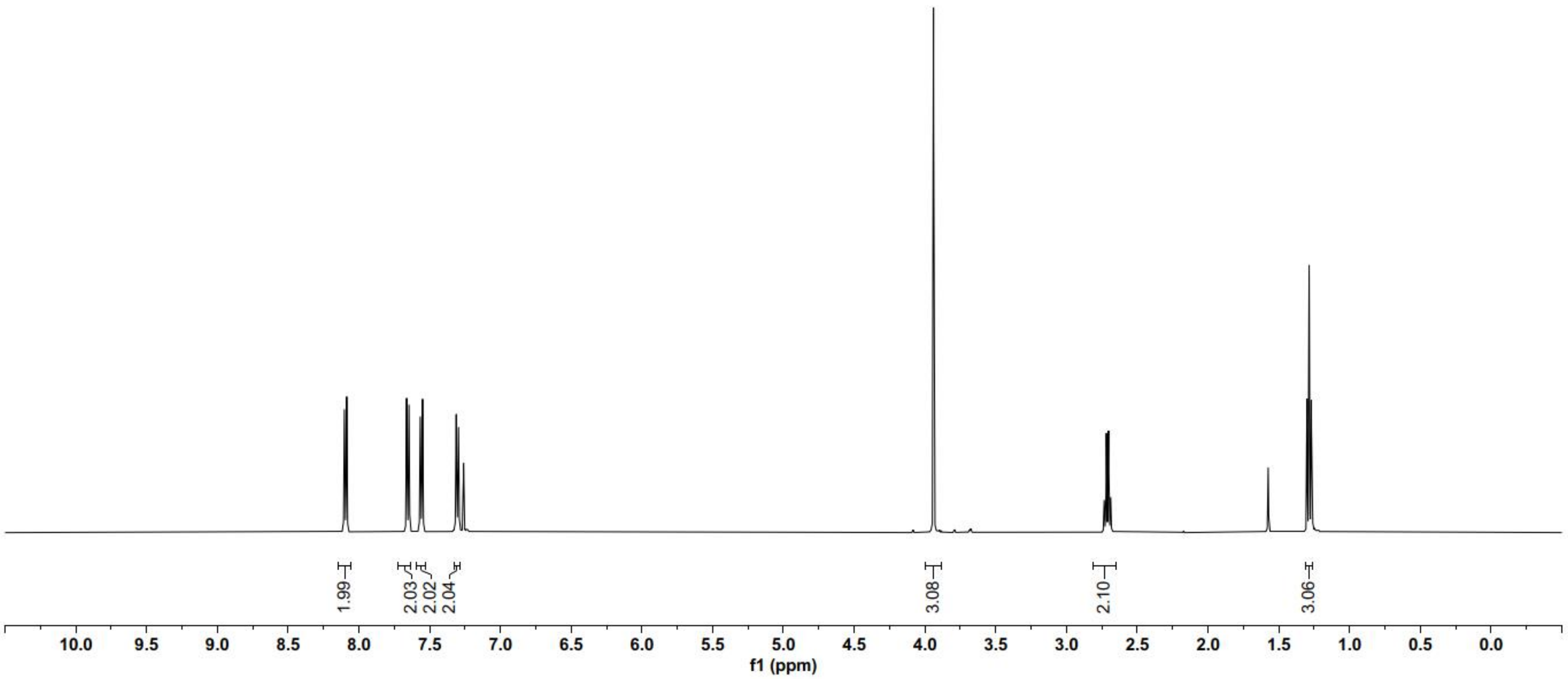

^{13}C NMR of 28

$CDCl_3$, 126 MHz, 25 °C

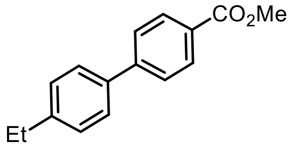

28

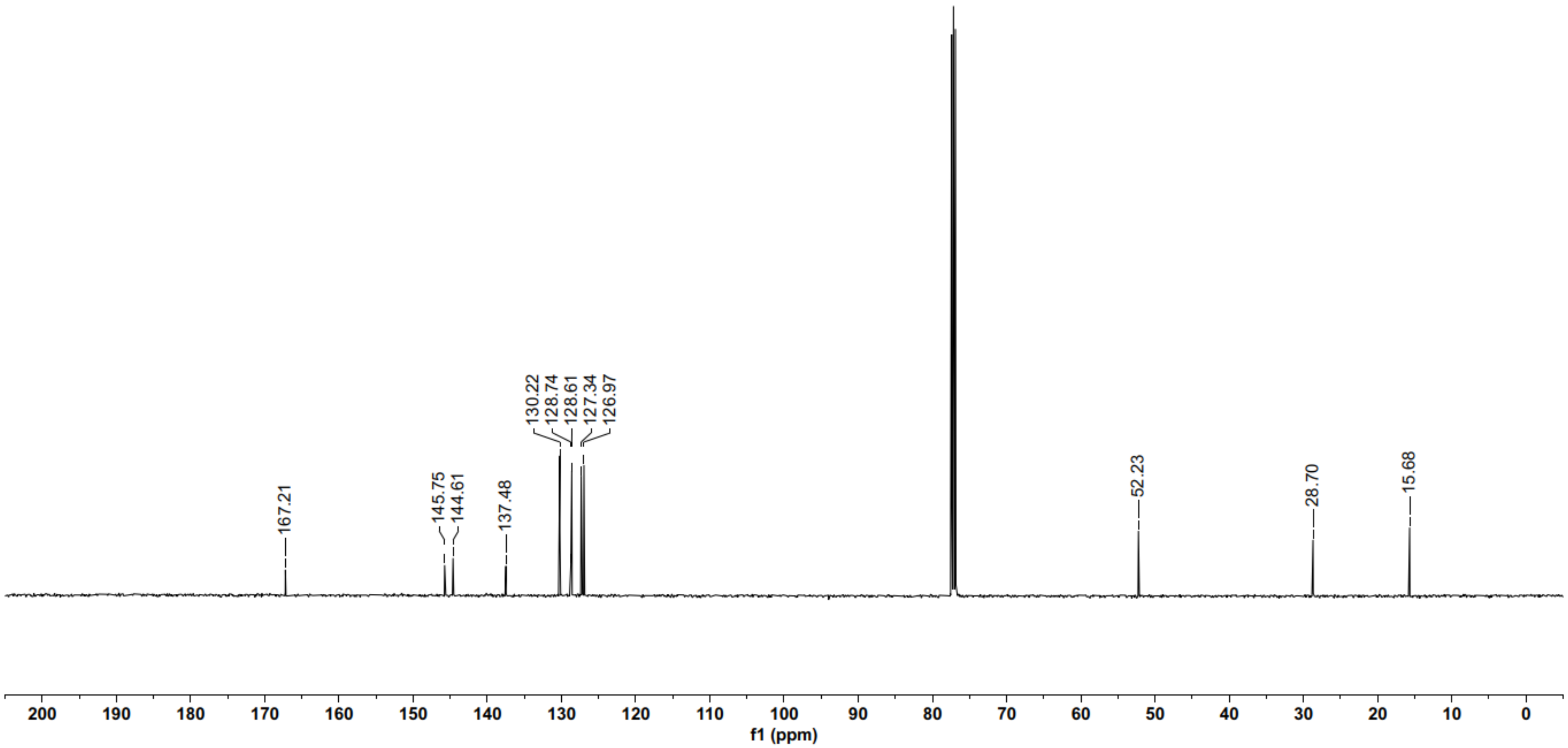

^{1}H NMR of 3

CDCl$_3$, 500 MHz, 25 °C

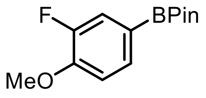

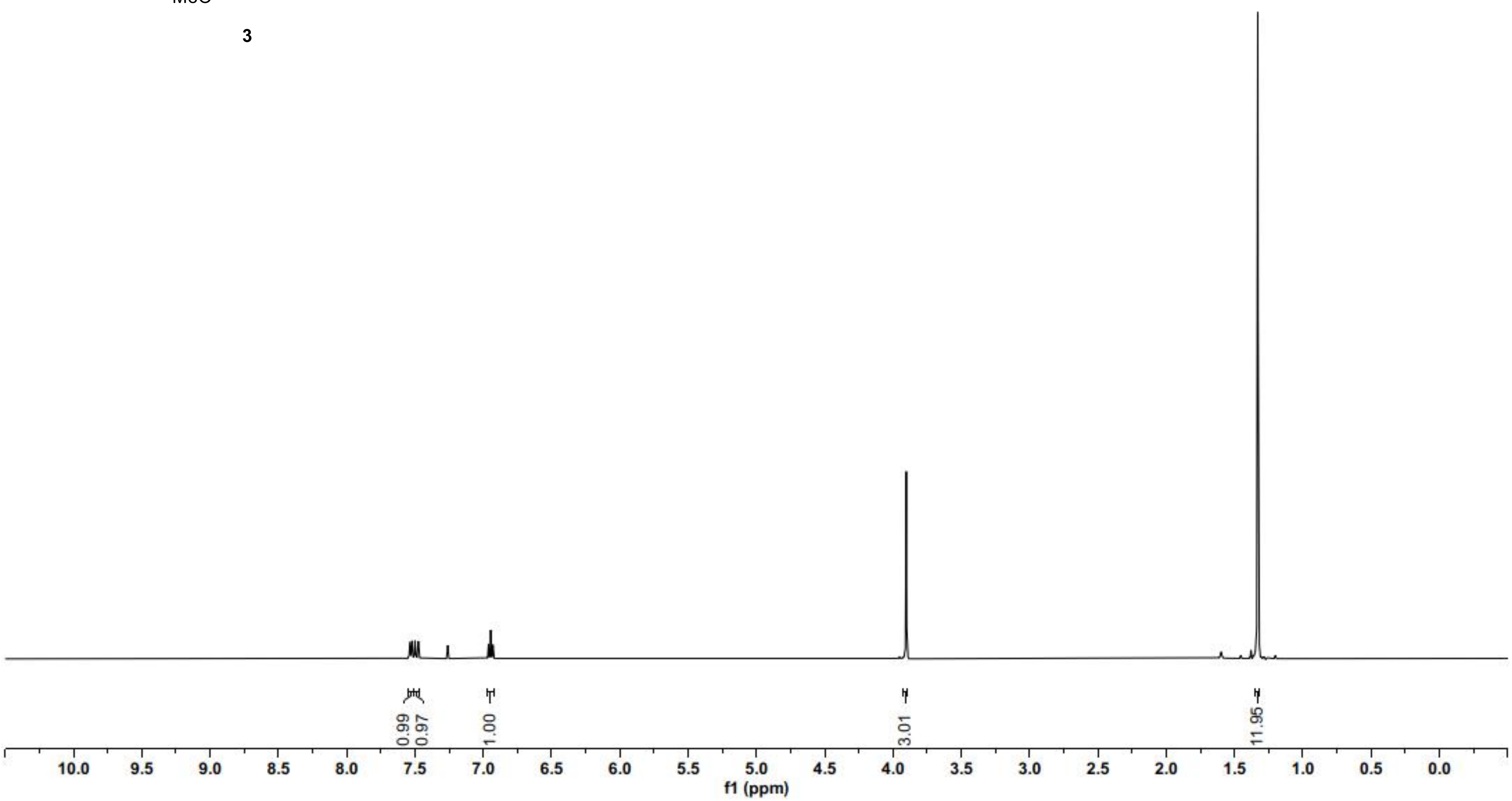

^{13}C NMR of 3

$CDCl_3$, 126 MHz, 25 °C

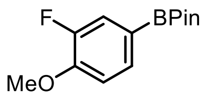

3

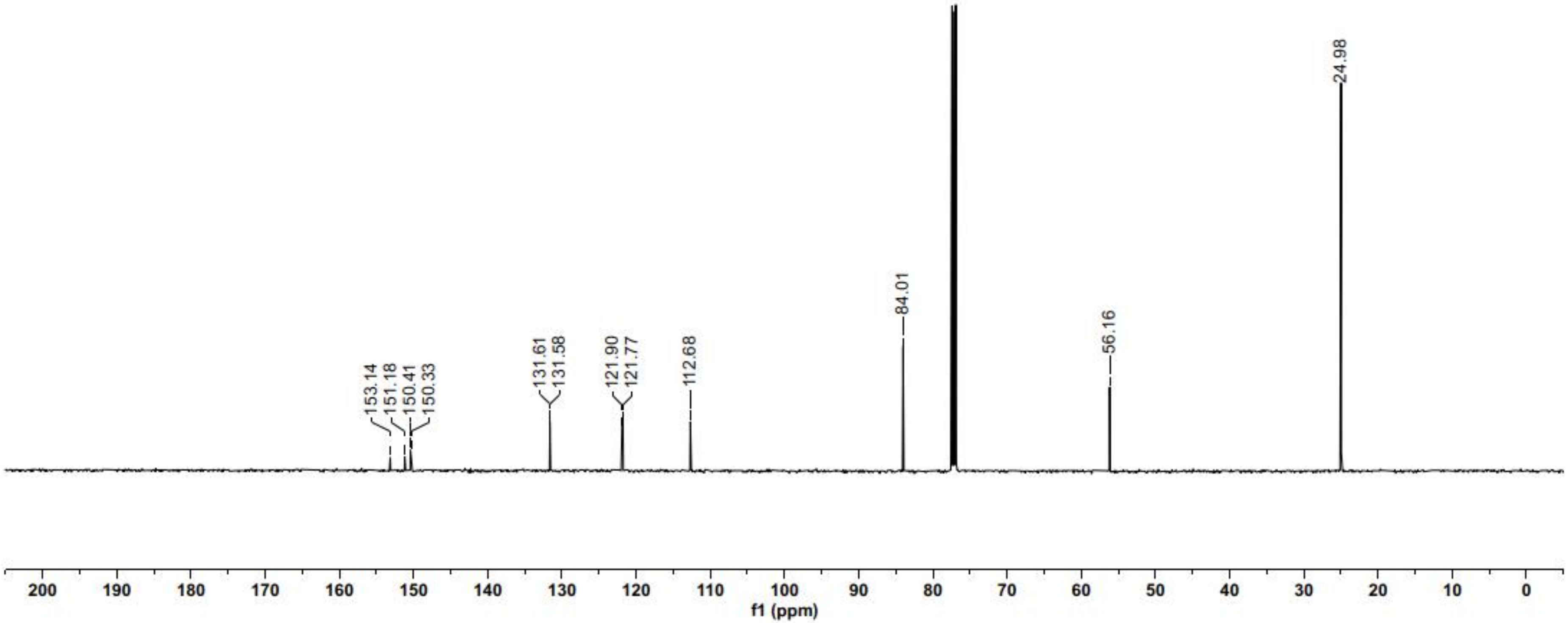

^{19}F NMR of 3

$CDCl_3$, 471 MHz, 25 °C

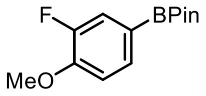

3

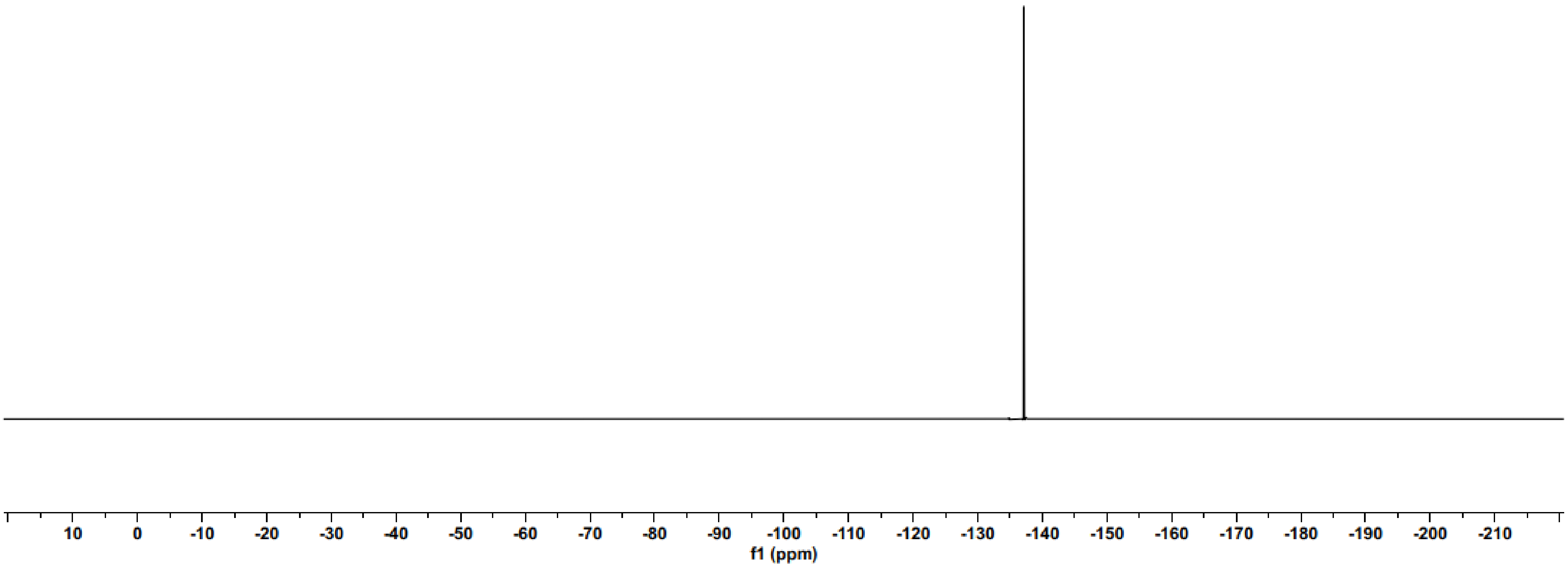

^{11}B NMR of 3

$CDCl_3$, 96 MHz, 25 °C

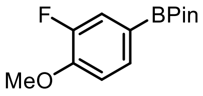

3

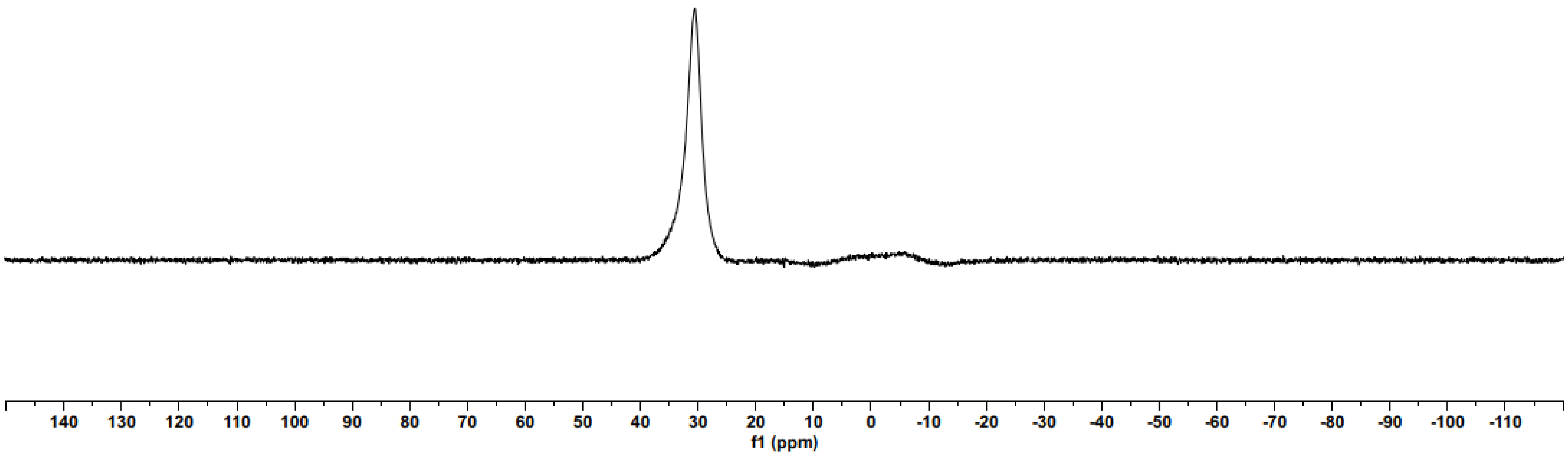

^{1}H NMR of 3a

CDCl$_3$, 500 MHz, 25 °C

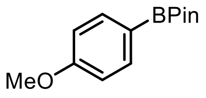

3a

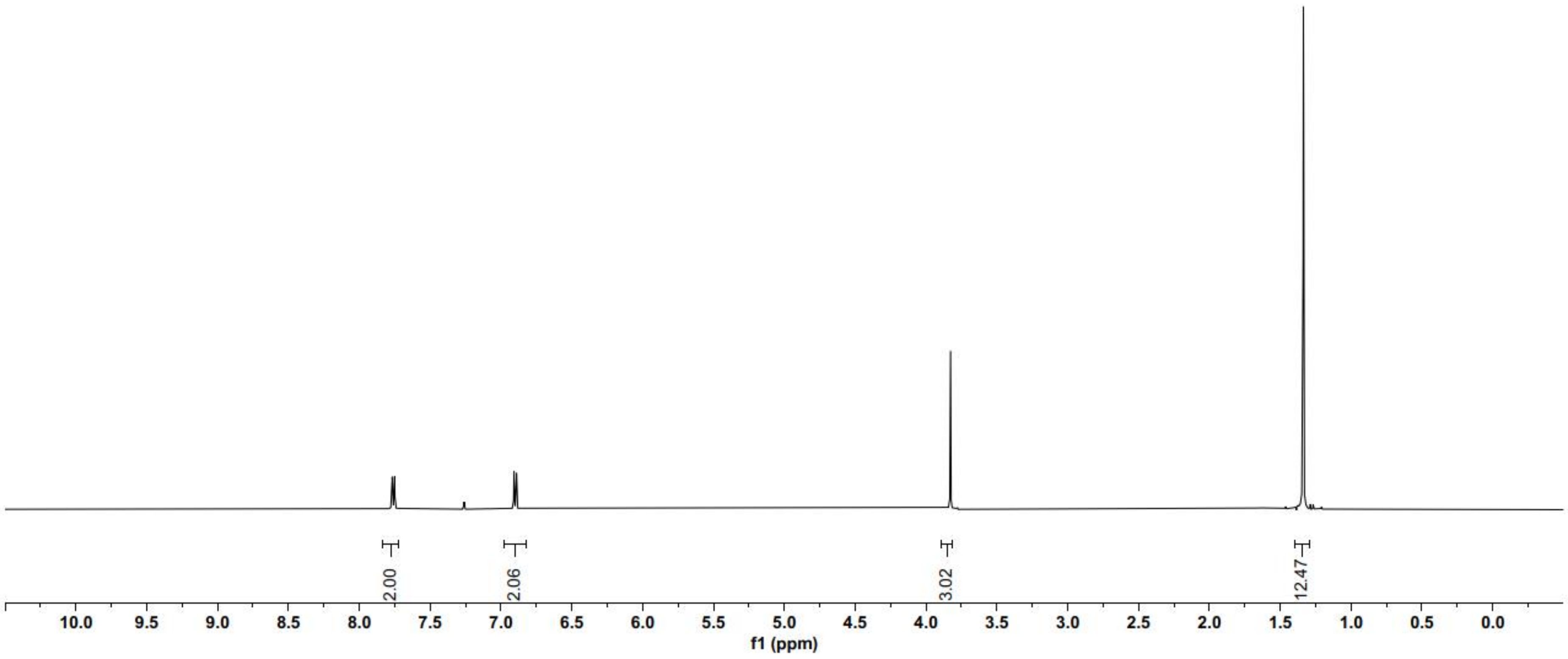

^{13}**C NMR of 3a**

$CDCl_3$, 126 MHz, 25 °C

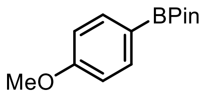

3a

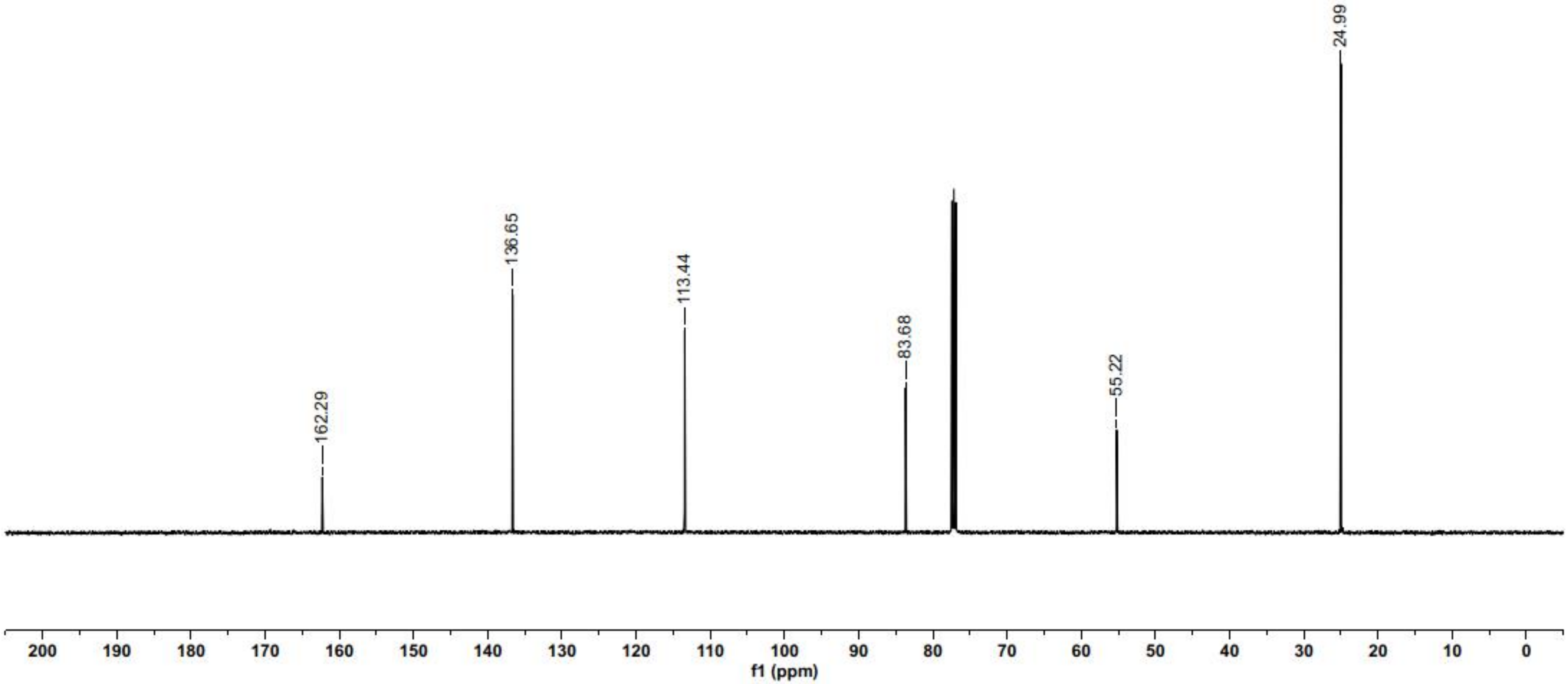

^{11}B NMR of 3a

$CDCl_3$, 96 MHz, 25 °C

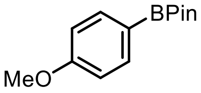

3a

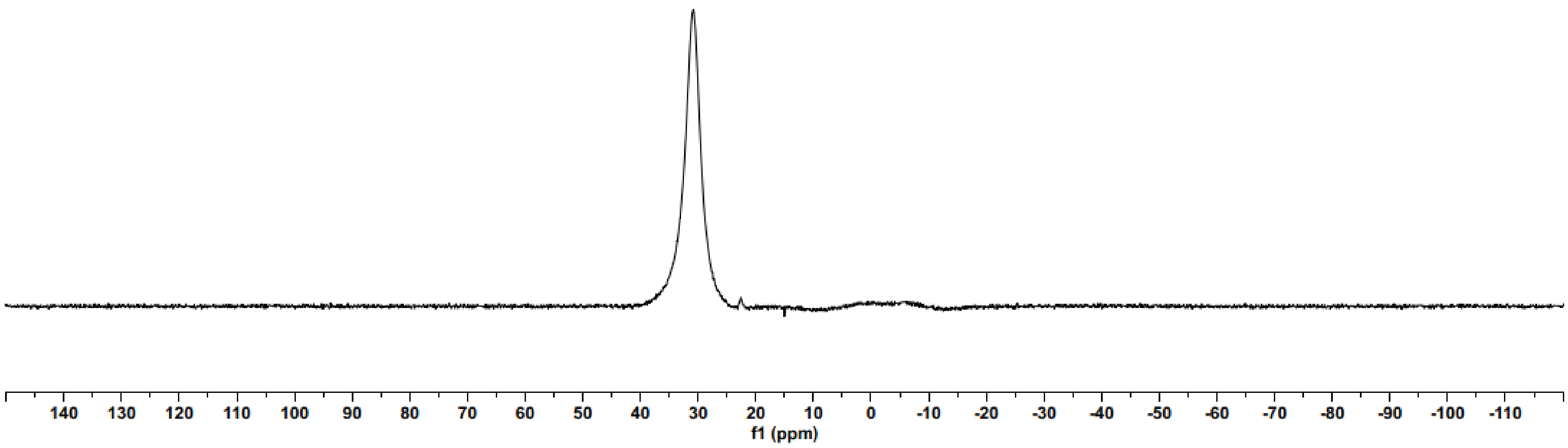

^{1}H NMR of 3b

CDCl$_3$, 500 MHz, 25 °C

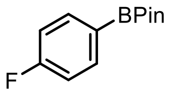

3b

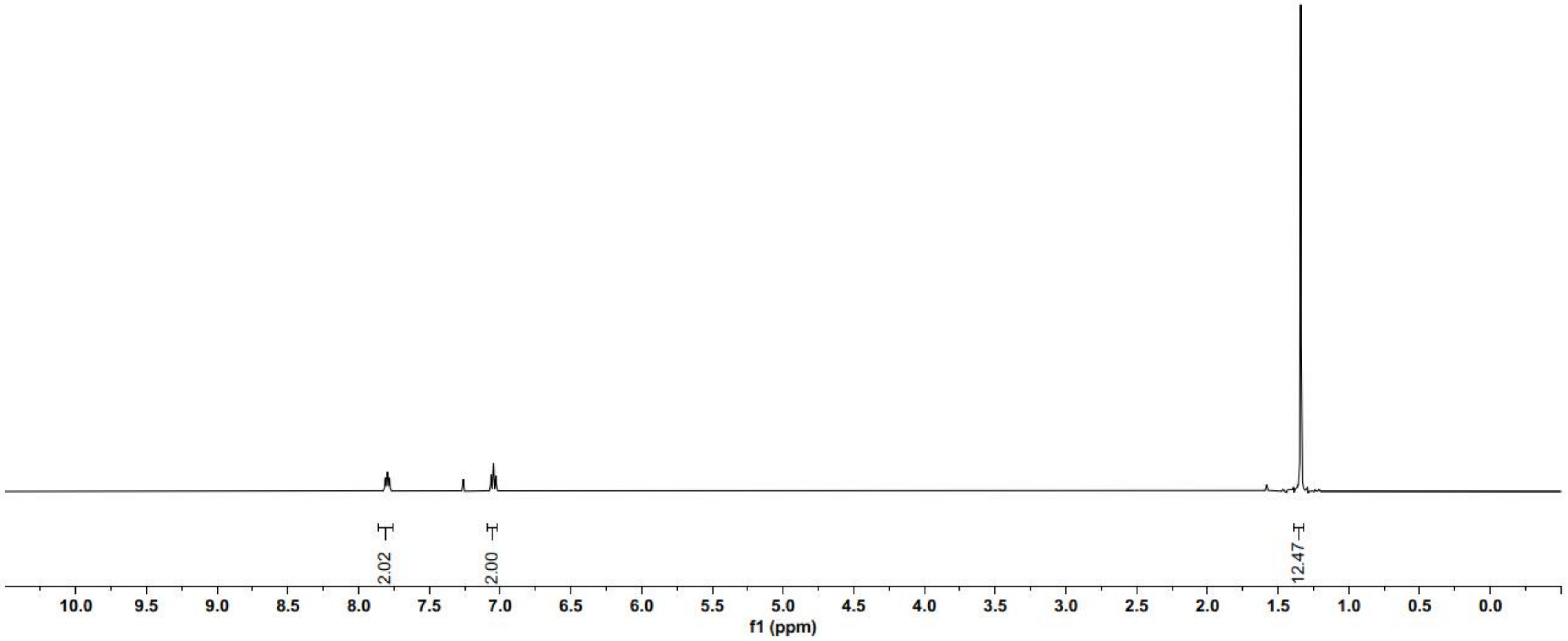

^{13}C NMR of 3b

$CDCl_3$, 126 MHz, 25 °C

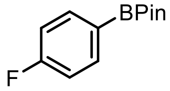

3b

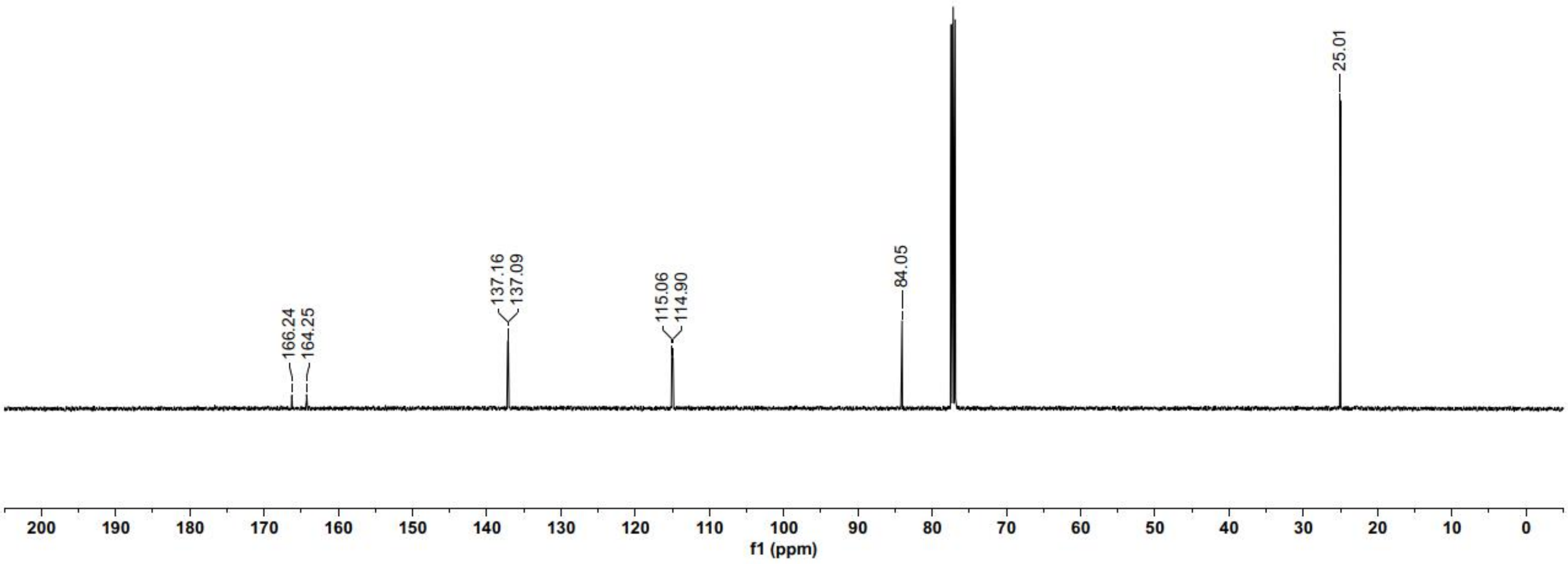

^{19}F NMR of 3b

$CDCl_3$, 471 MHz, 25 °C

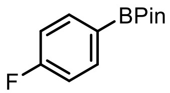

3b

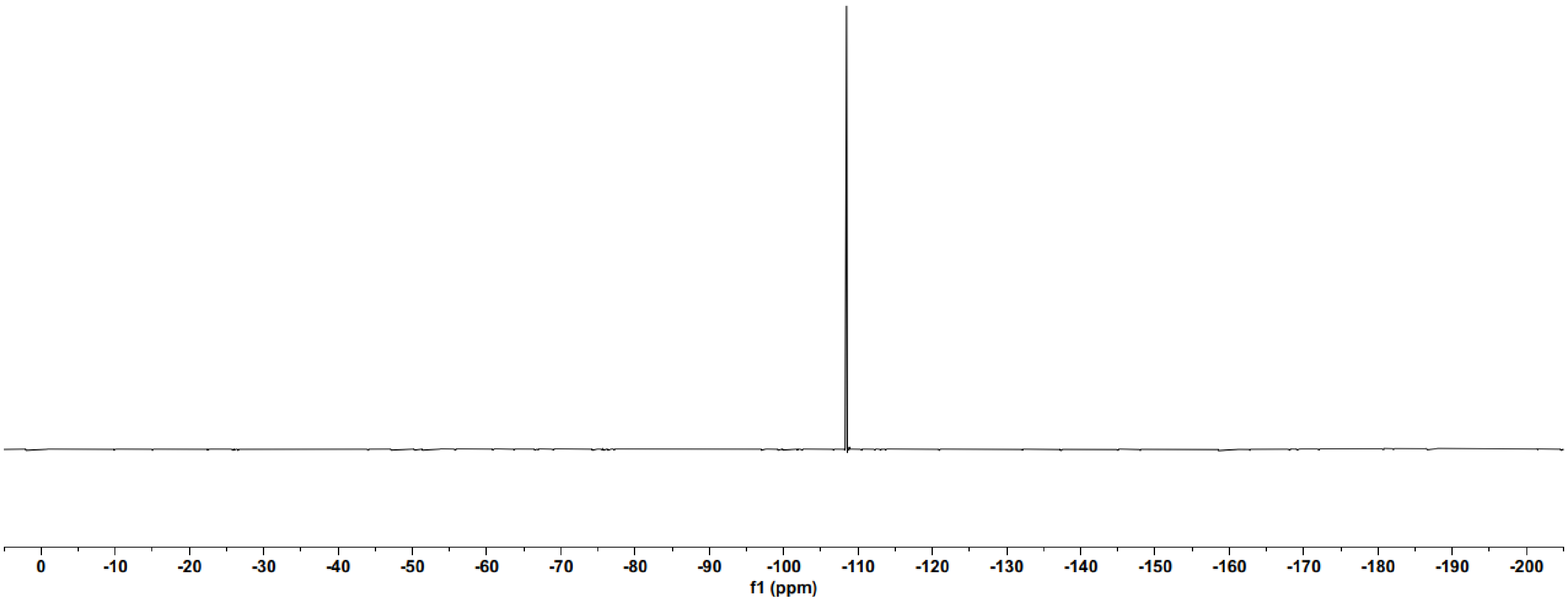

^{11}B NMR of 3b

$CDCl_3$, 96 MHz, 25 °C

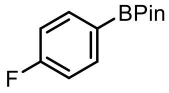

3b

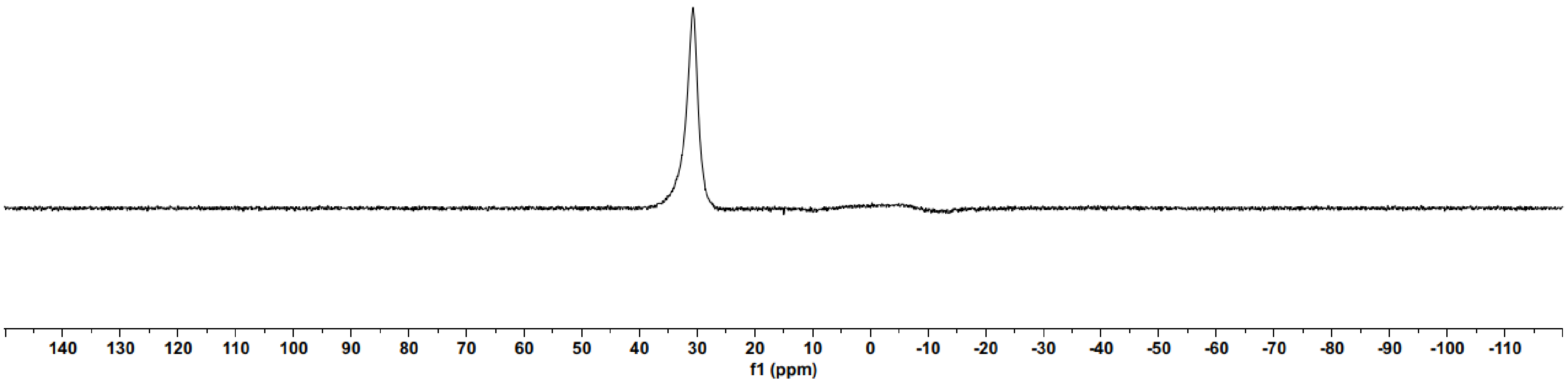

^{1}H NMR of 3c

CDCl$_3$, 500 MHz, 25 °C

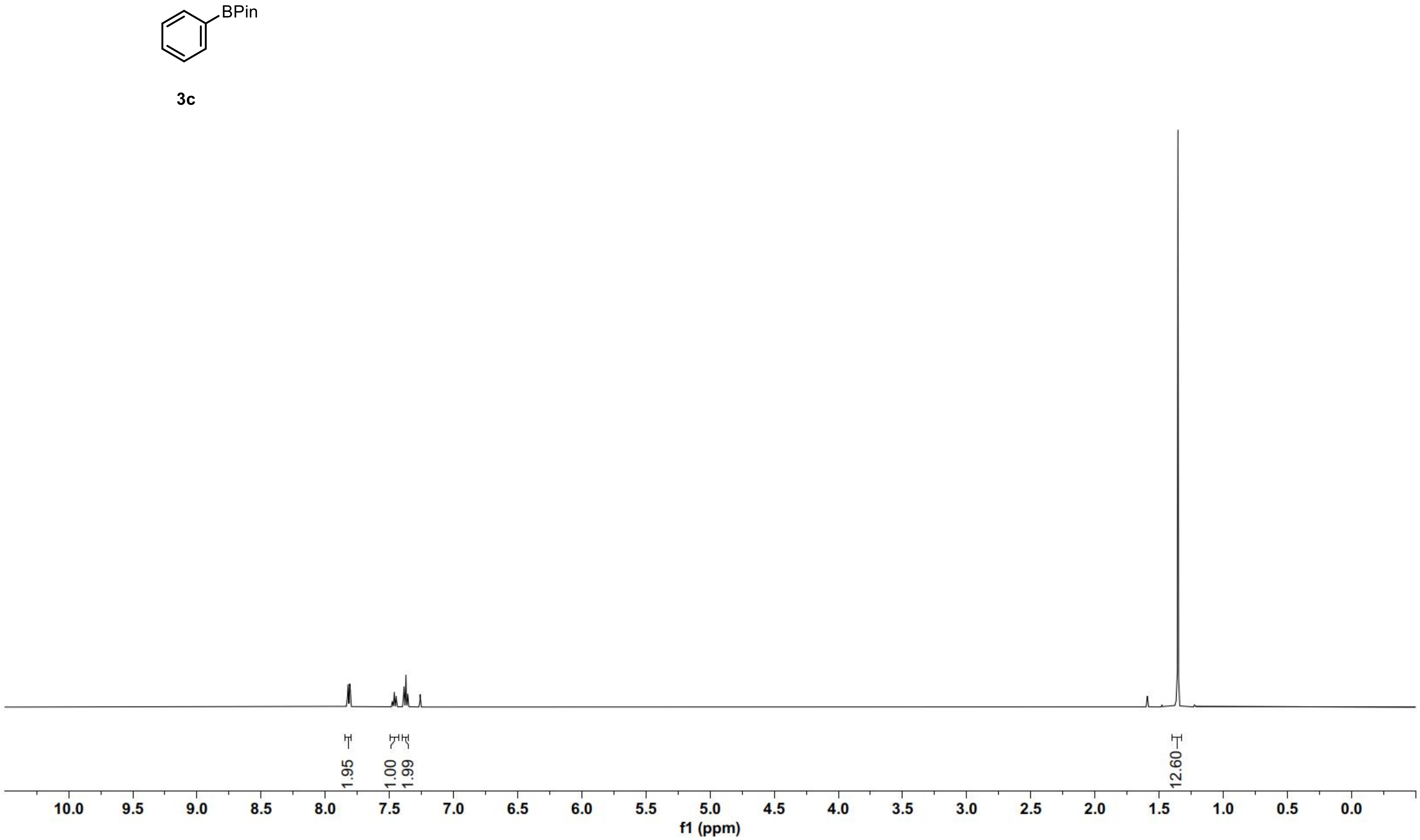

3c

^{13}C NMR of 3c

$CDCl_3$, 126 MHz, 25 °C

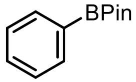

3c

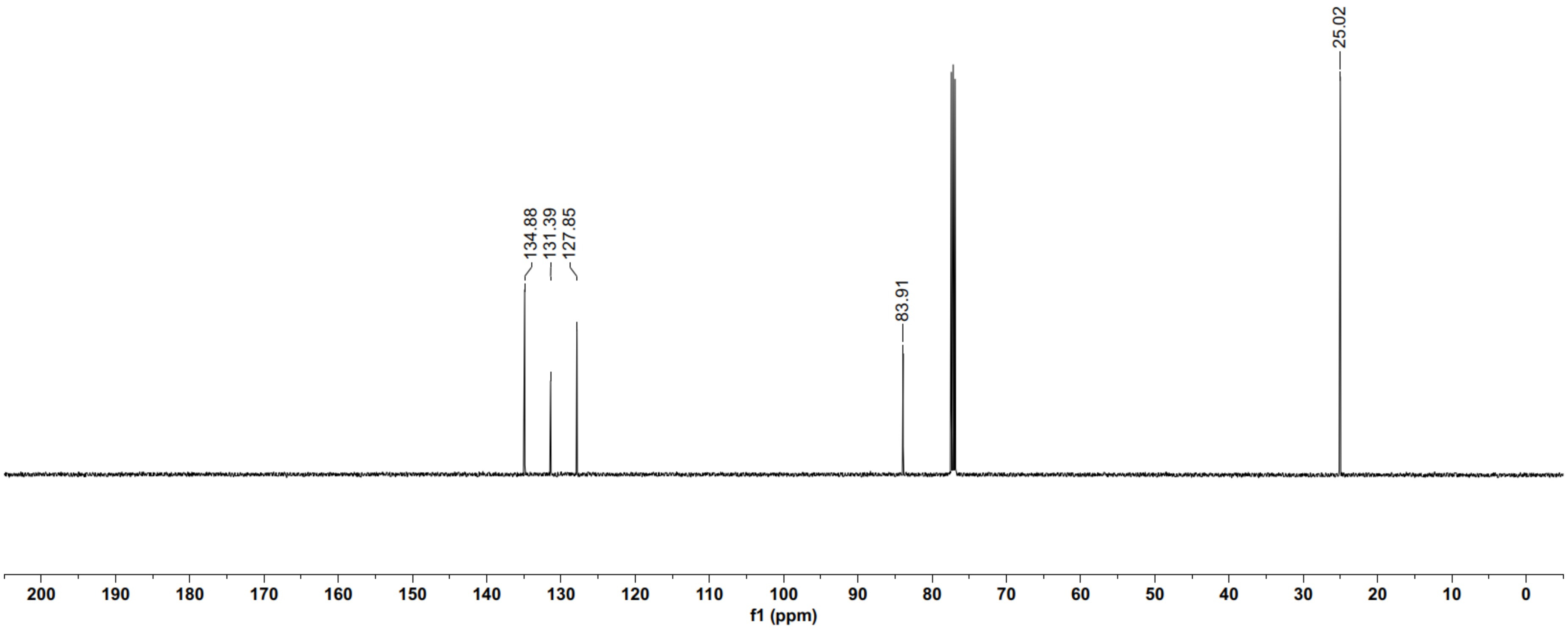

^{11}B NMR of 3c

$CDCl_3$, 96 MHz, 25 °C

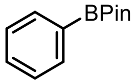

3c

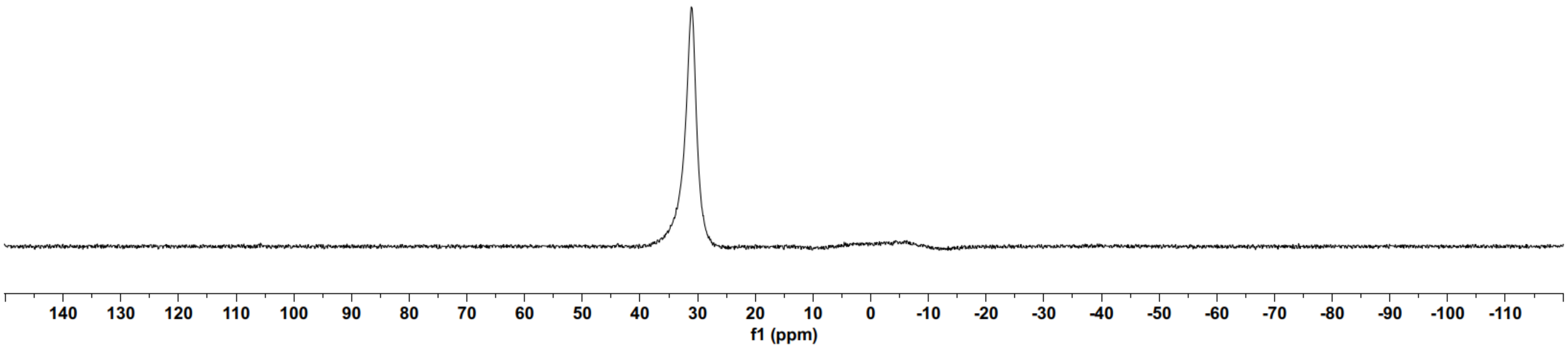

^{1}H NMR of 3d

$CDCl_3$, 500 MHz, 25 °C

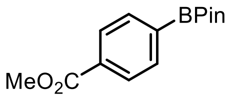

3d

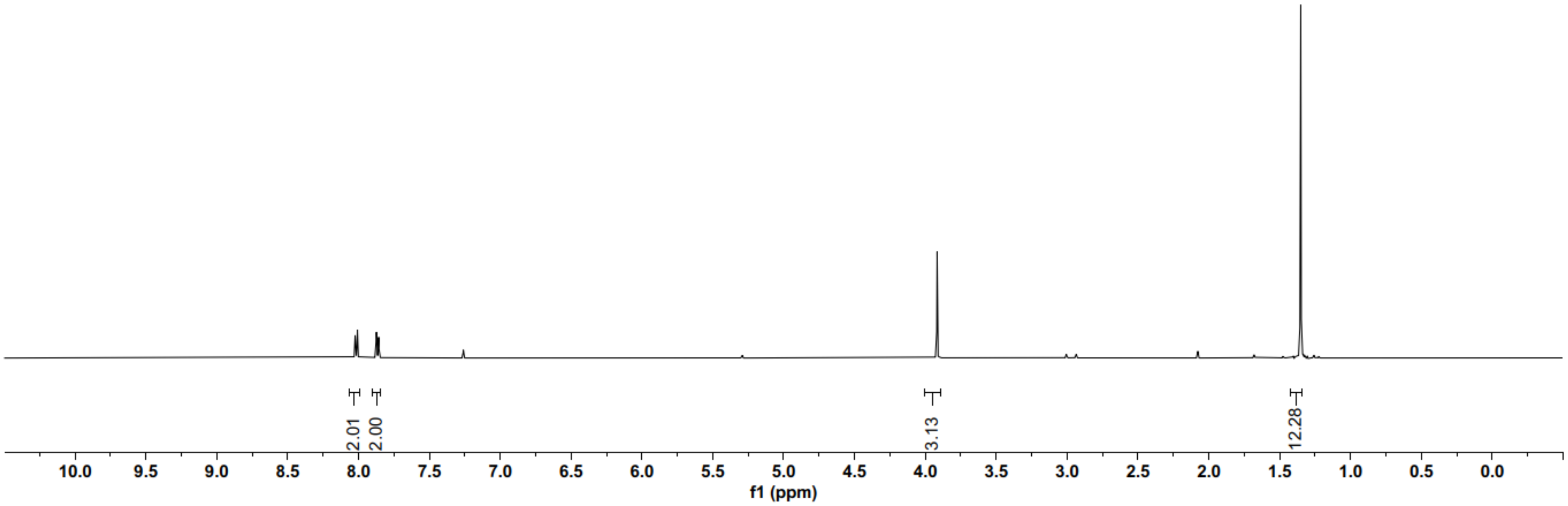

^{13}C NMR of 3d

$CDCl_3$, 126 MHz, 25 °C

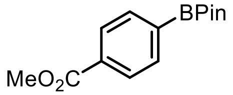

3d

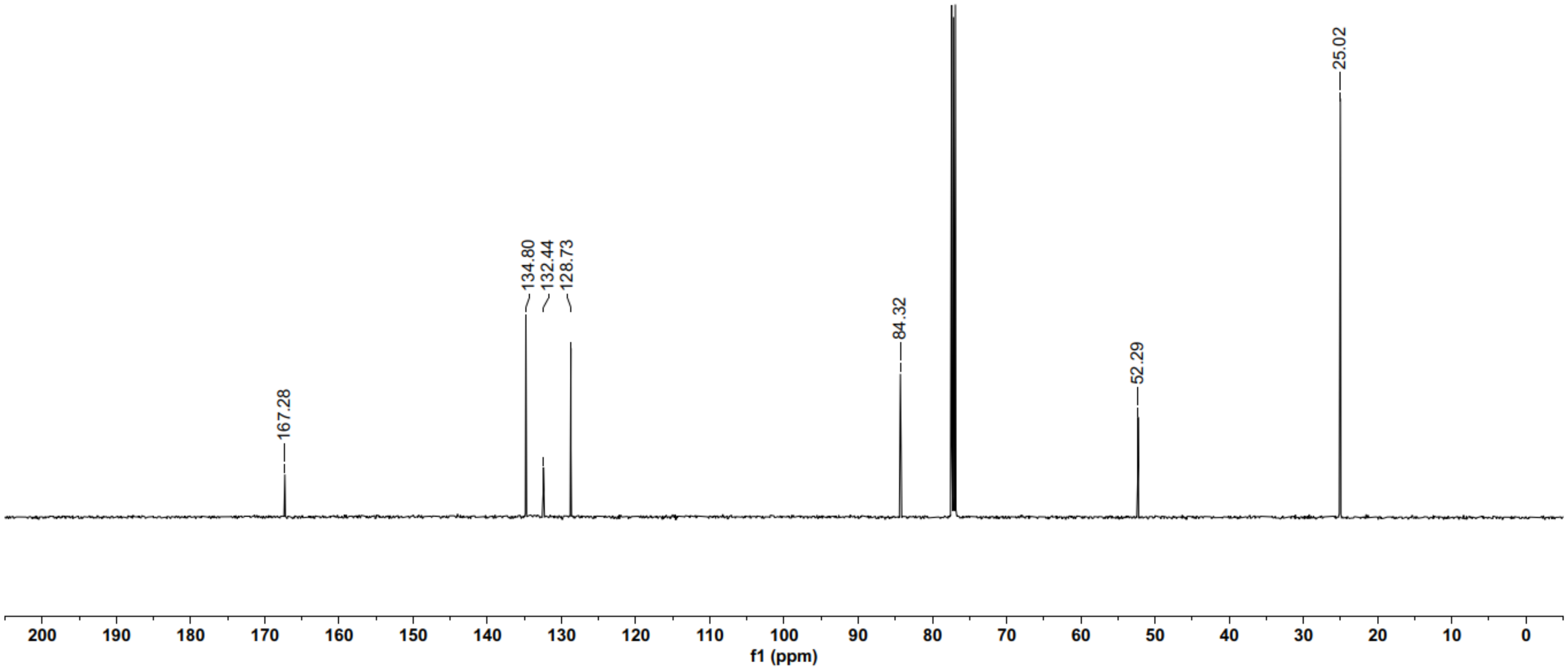

^{11}B NMR of 3d

$CDCl_3$, 96 MHz, 25 °C

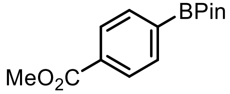

3d

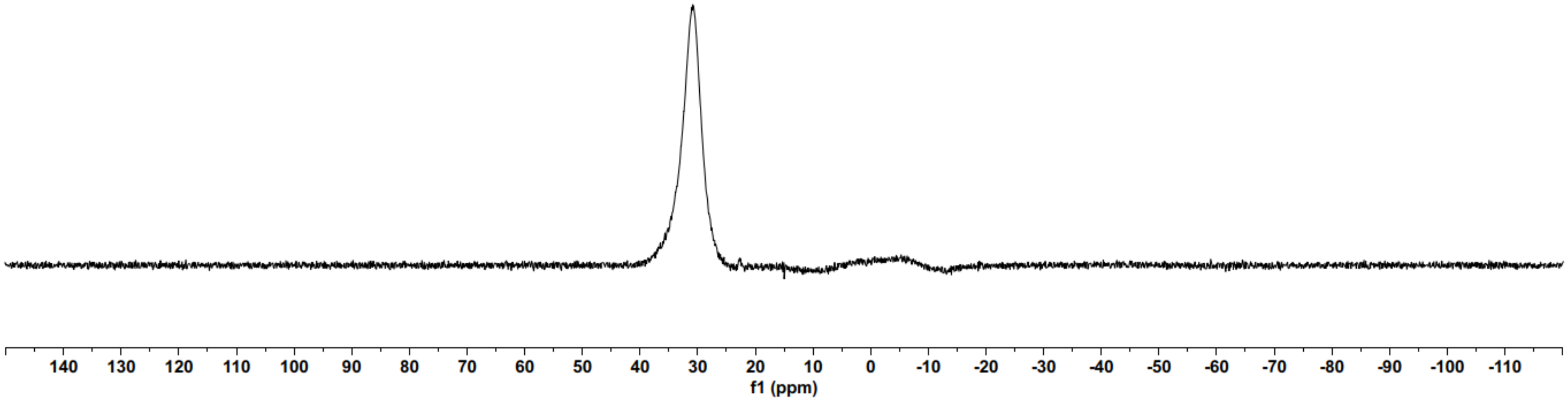

^{1}H NMR of 3e

$CDCl_3$, 500 MHz, 25 °C

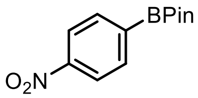

3e

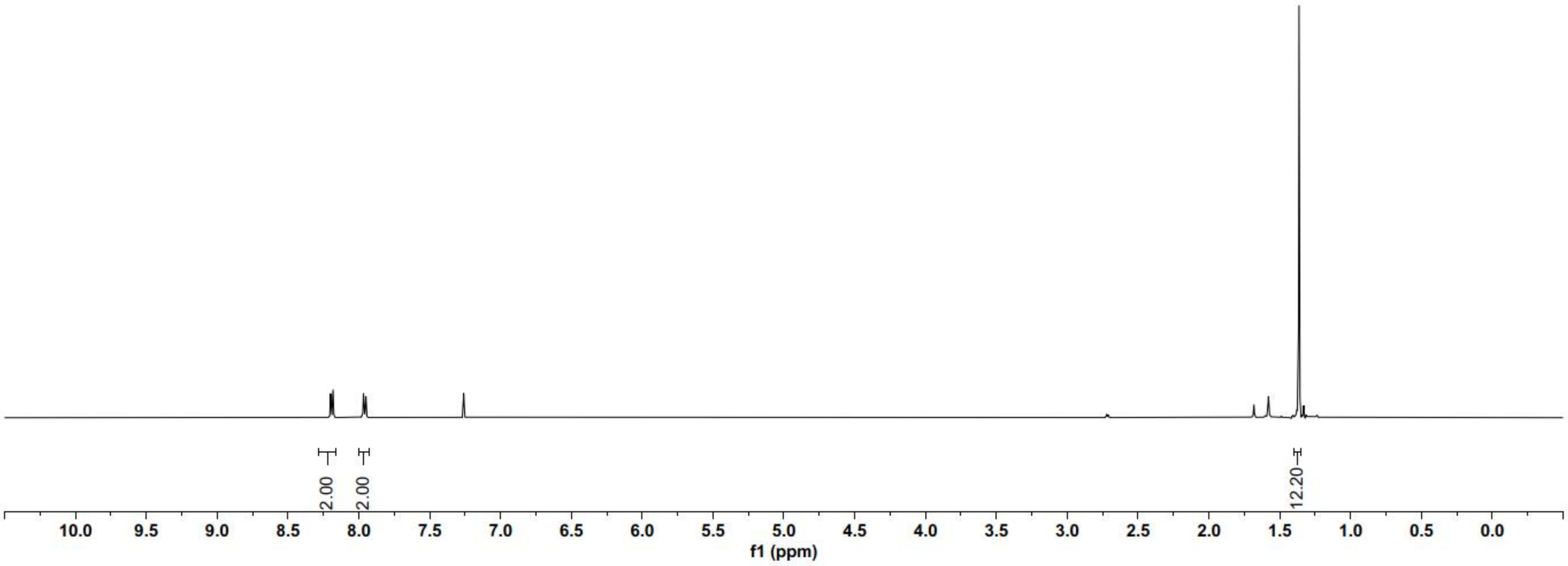

^{13}C NMR of 3e

$CDCl_3$, 126 MHz, 25 °C

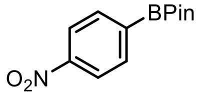

3e

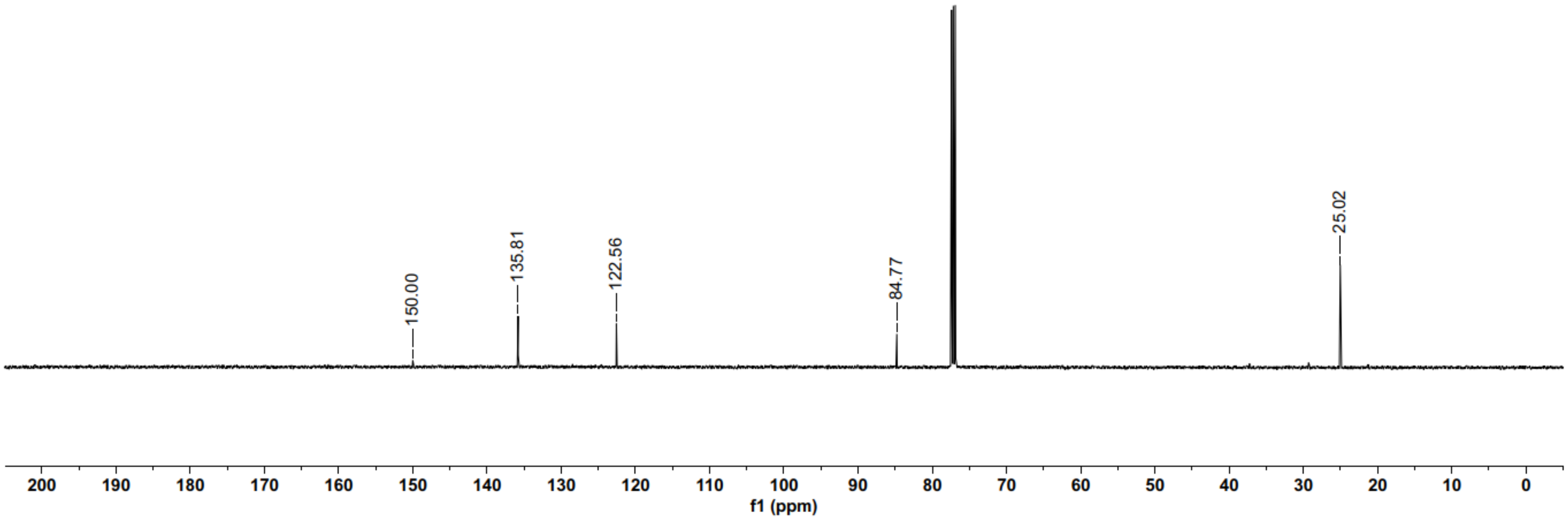

^{11}B NMR of 3e

$CDCl_3$, 96 MHz, 25 °C

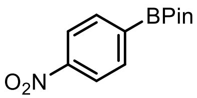

3e

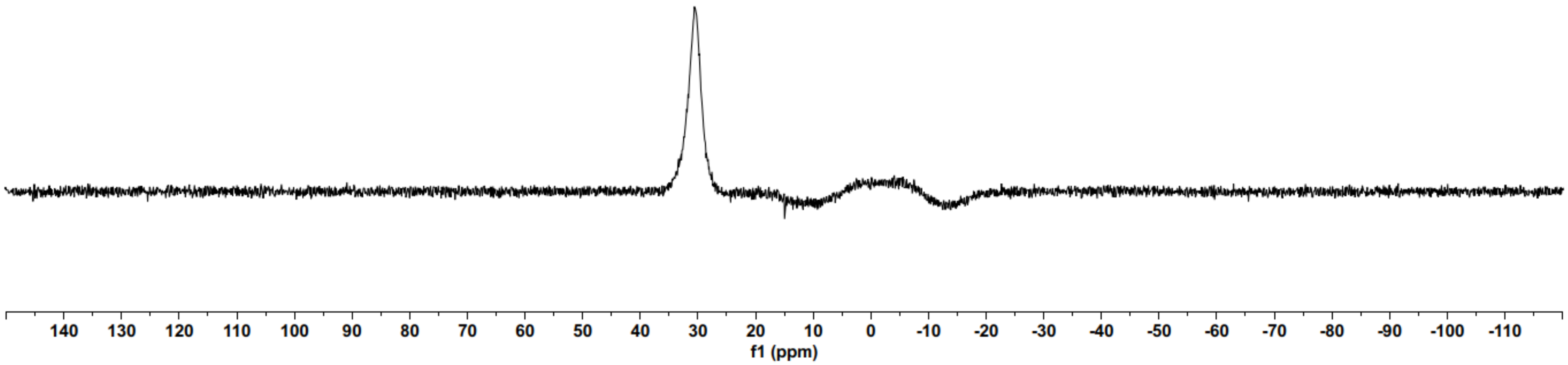

^{1}H NMR of 1-BF_4

CD_3CN, 500 MHz, 25 °C

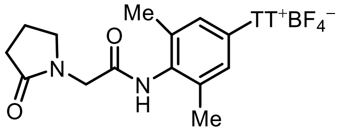

1-BF_4

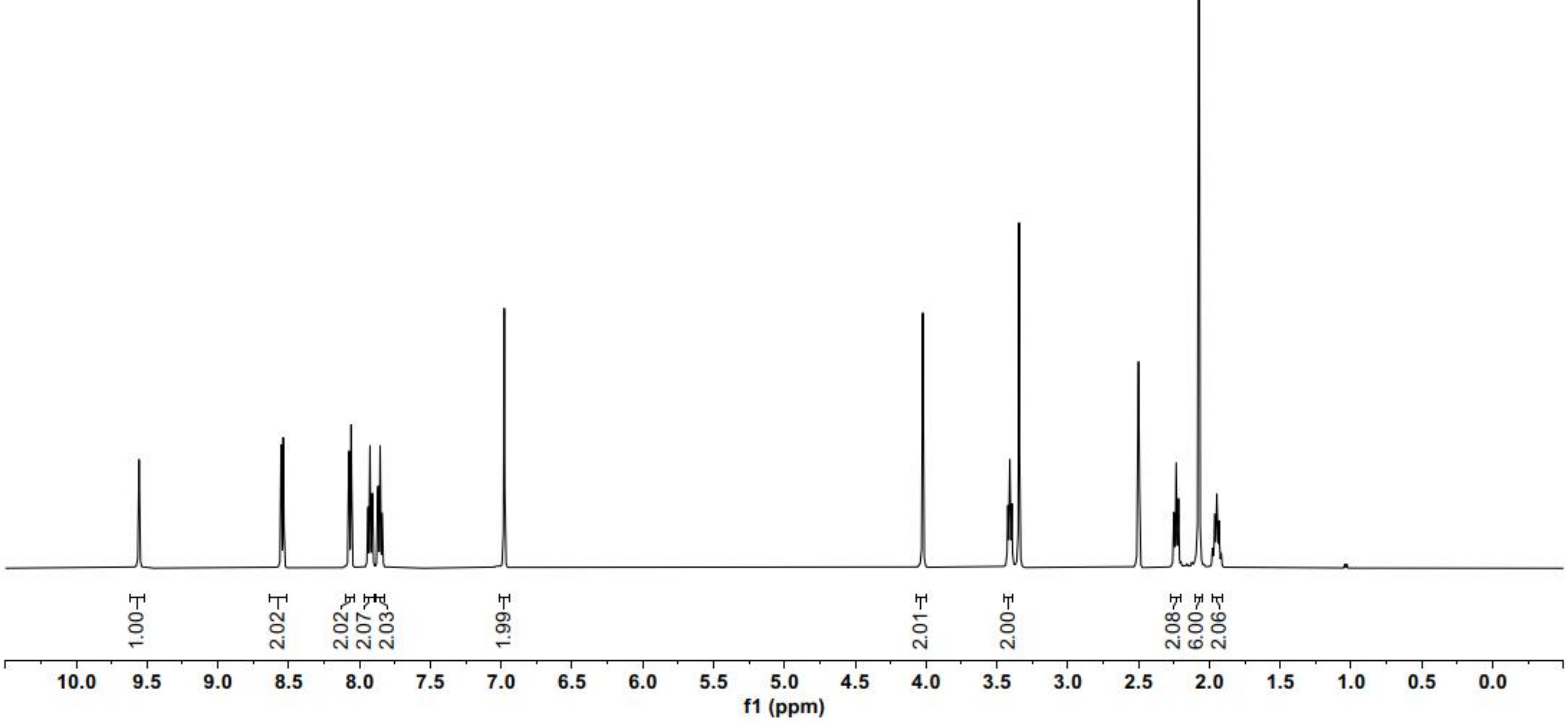

^{13}C NMR of 1-BF_4

CD_3CN, 126 MHz, 25 °C

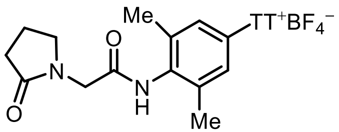

1-BF_4

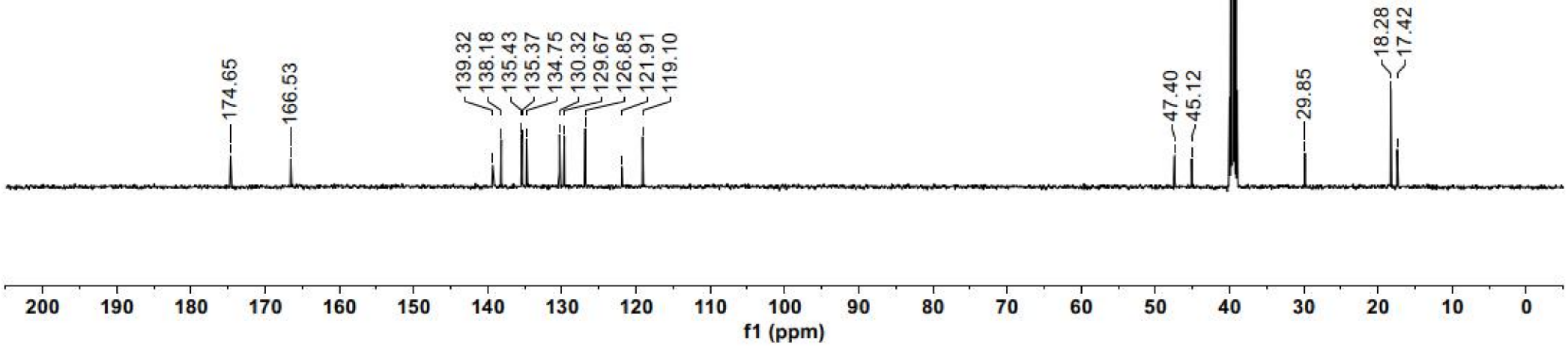

^{19}F NMR of 1-BF_4

CD_3CN, 471 MHz, 25 °C

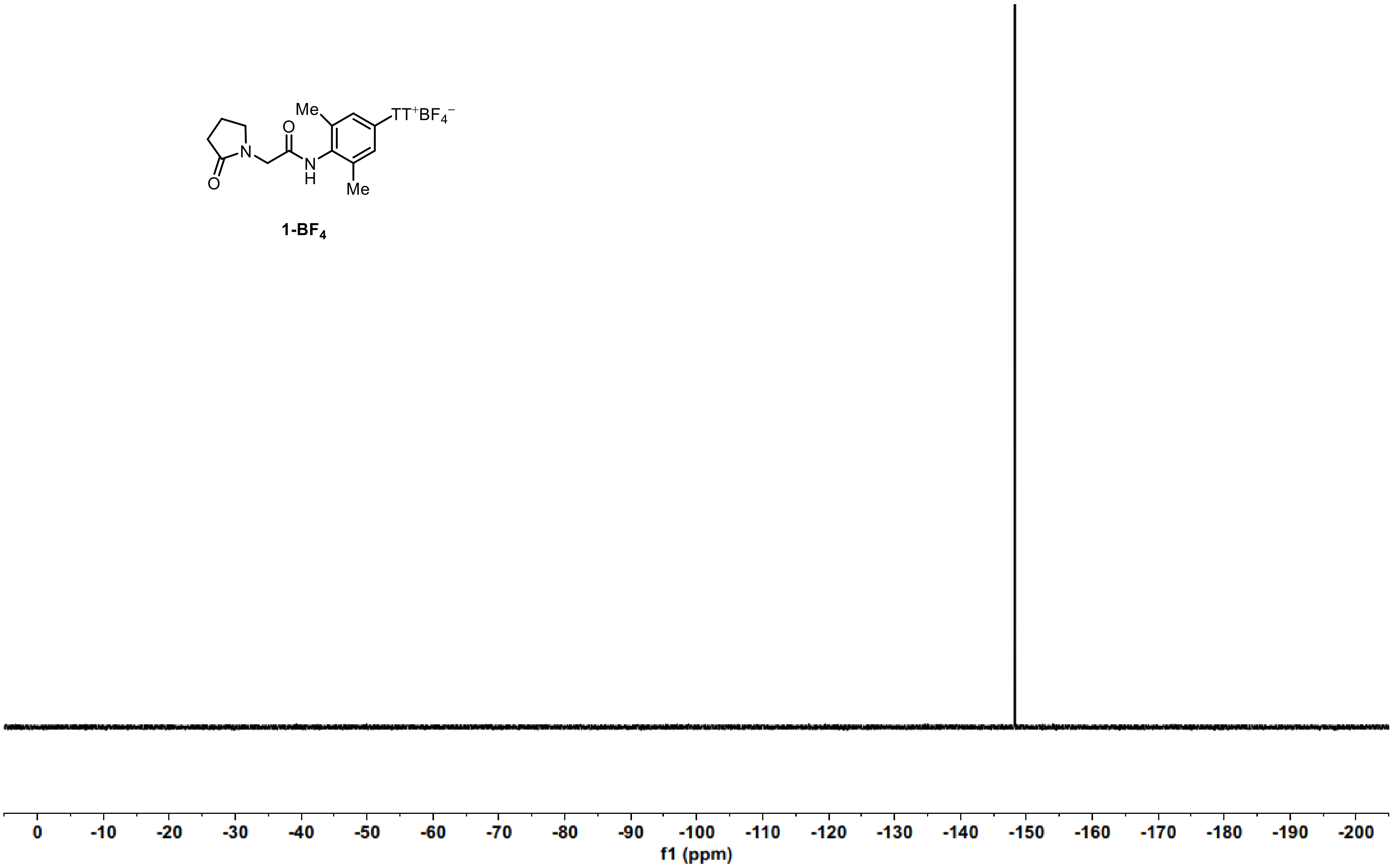

^{1}H NMR of 3d-BF_4

CD_3CN, 500 MHz, 25 °C

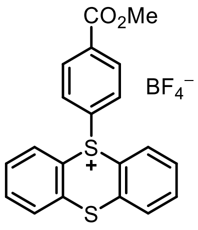

3d-BF_4

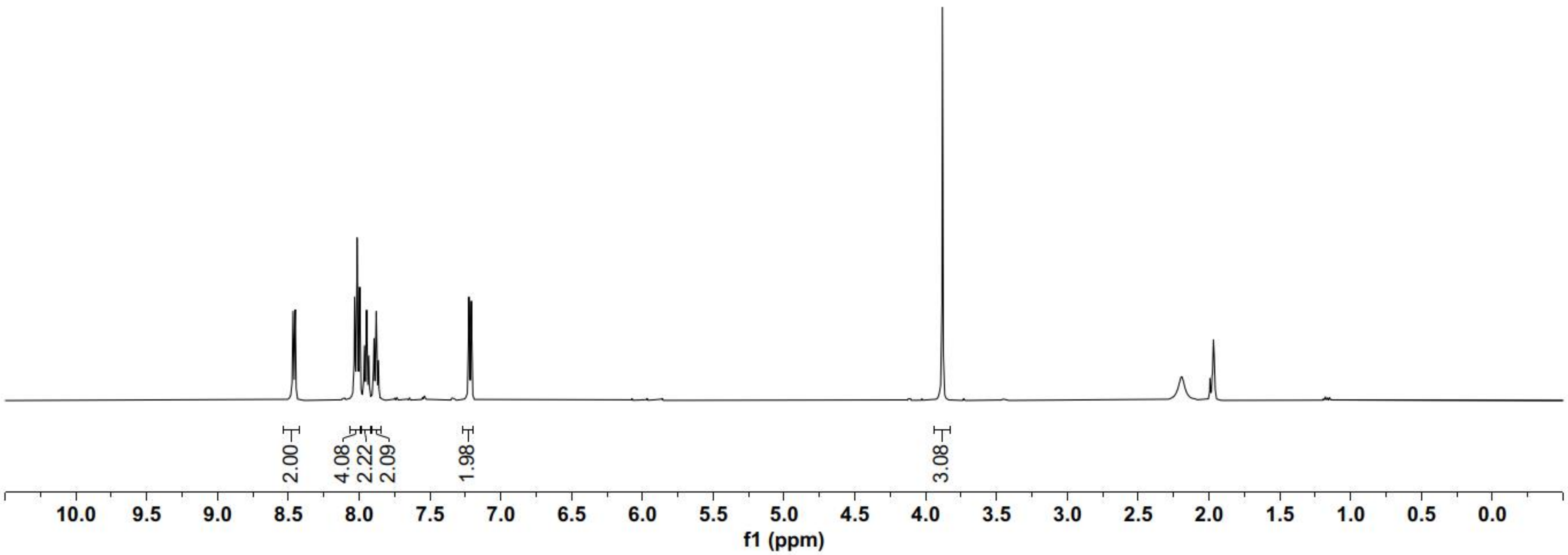

^{13}C NMR of 3d-BF_4

CD_3CN, 126 MHz, 25 °C

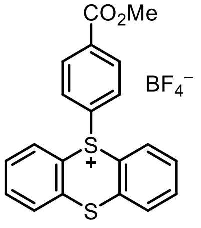

3d-BF_4

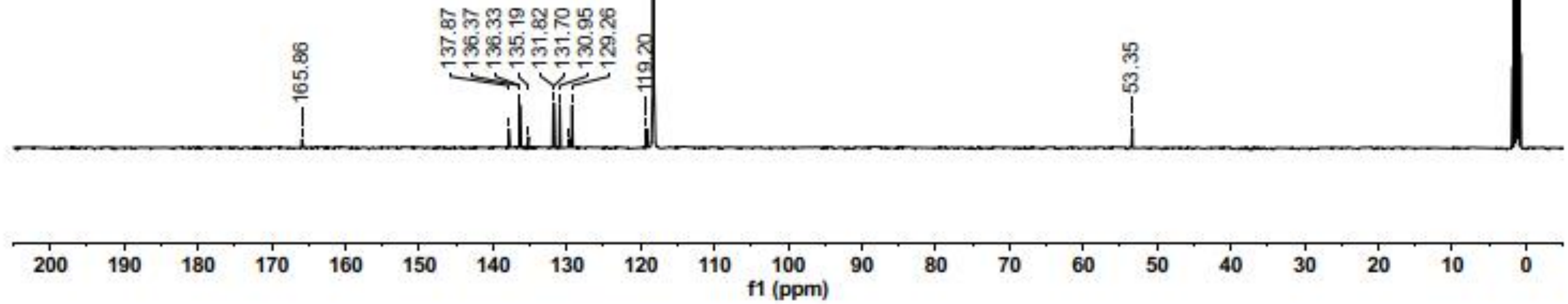

^{19}F NMR of 3d-BF_4

CD_3CN, 471 MHz, 25 °C

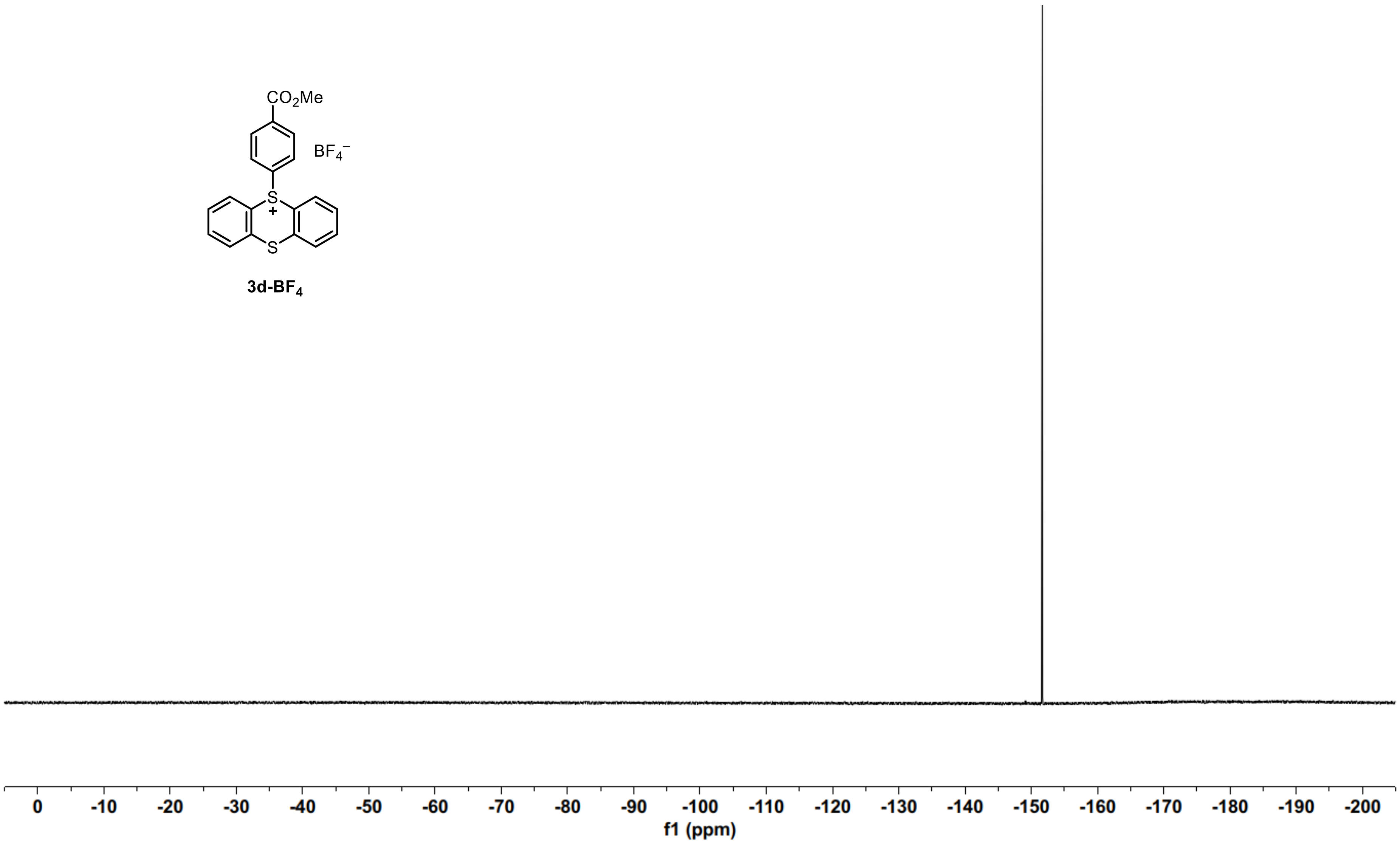

^{1}H NMR of 5-BF_4

CD_3CN, 300 MHz, 25 °C

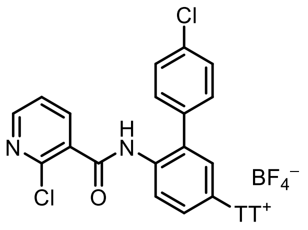

5-BF_4

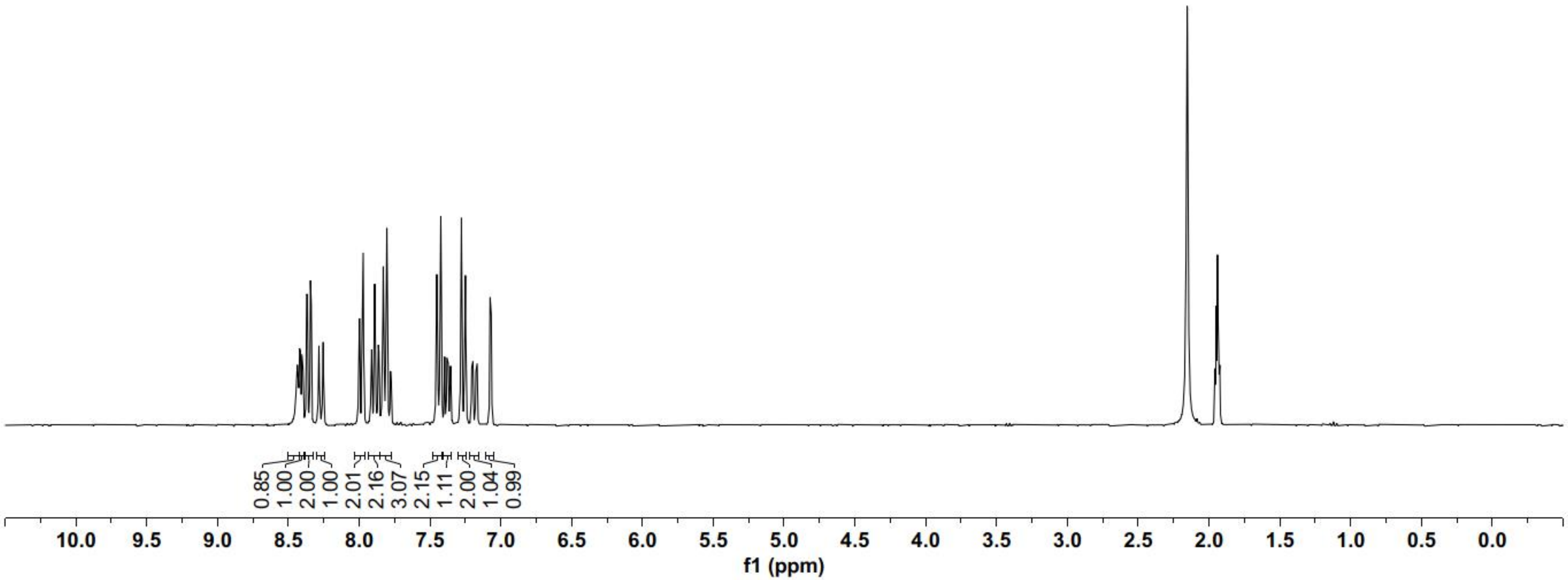

^{13}C NMR of 5-BF_4

CD_3CN, 126 MHz, 25 °C

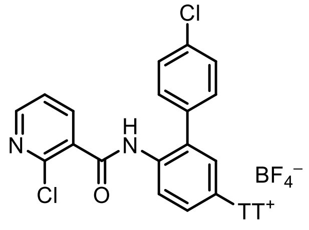

5-BF_4

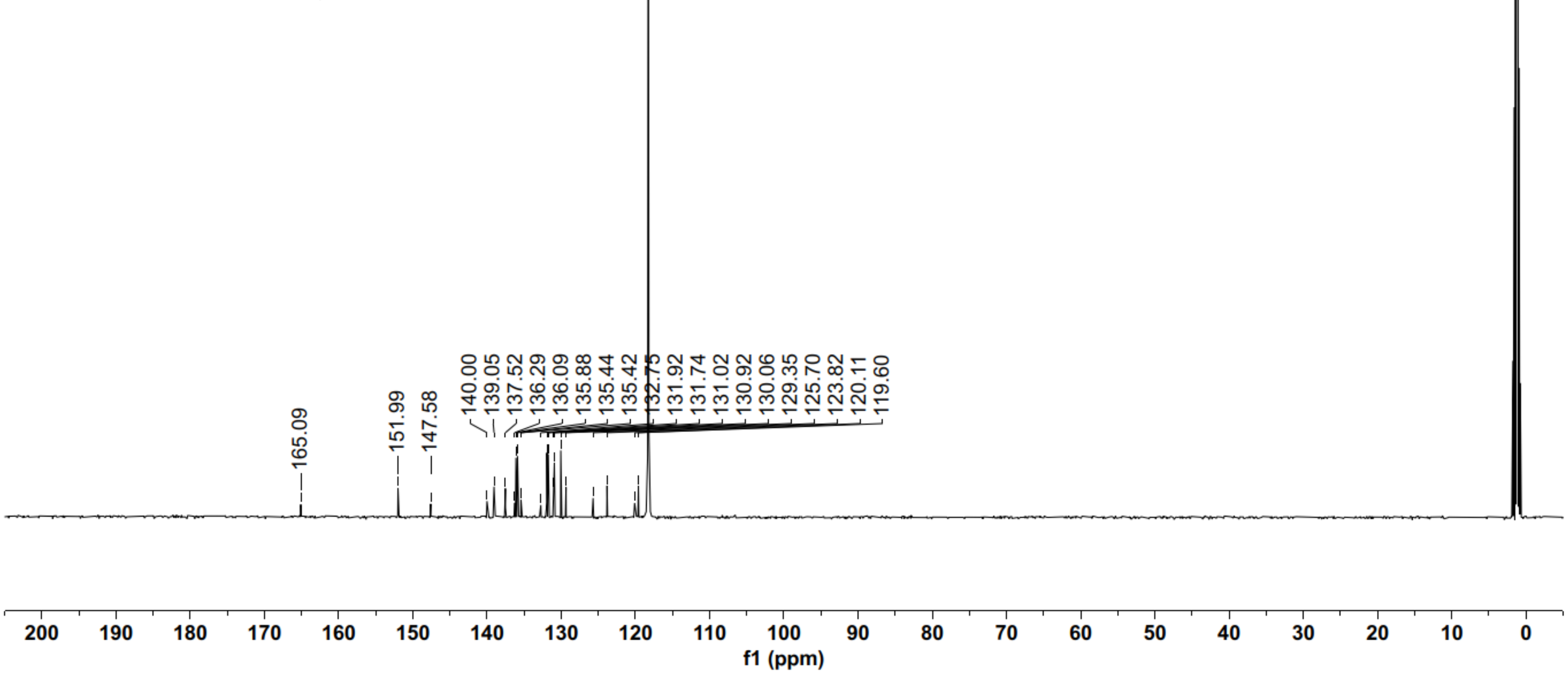

^{19}F NMR of 5-BF_4

CD_3CN, 282 MHz, 25 °C

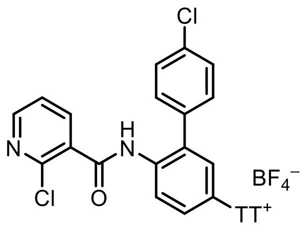

5-BF_4

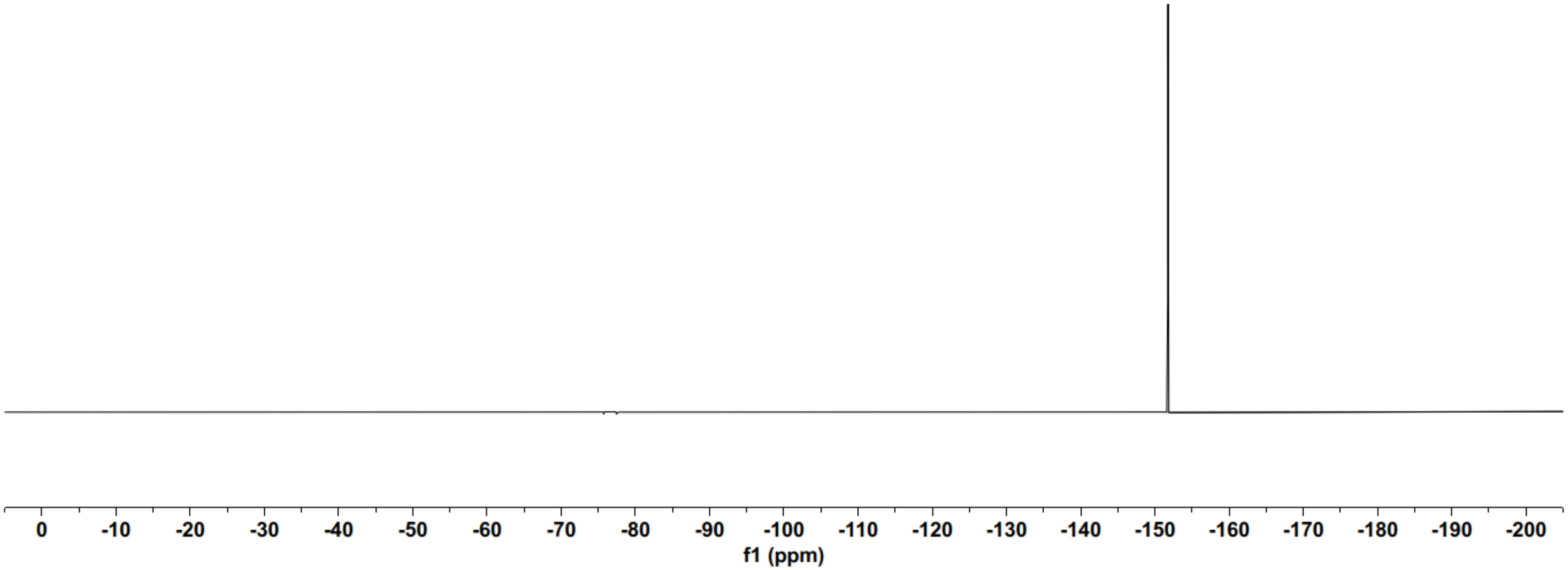

^{1}H NMR of 25-BF_4

DMSO-d6, 300 MHz, 25 °C

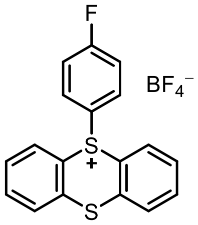

25-BF_4

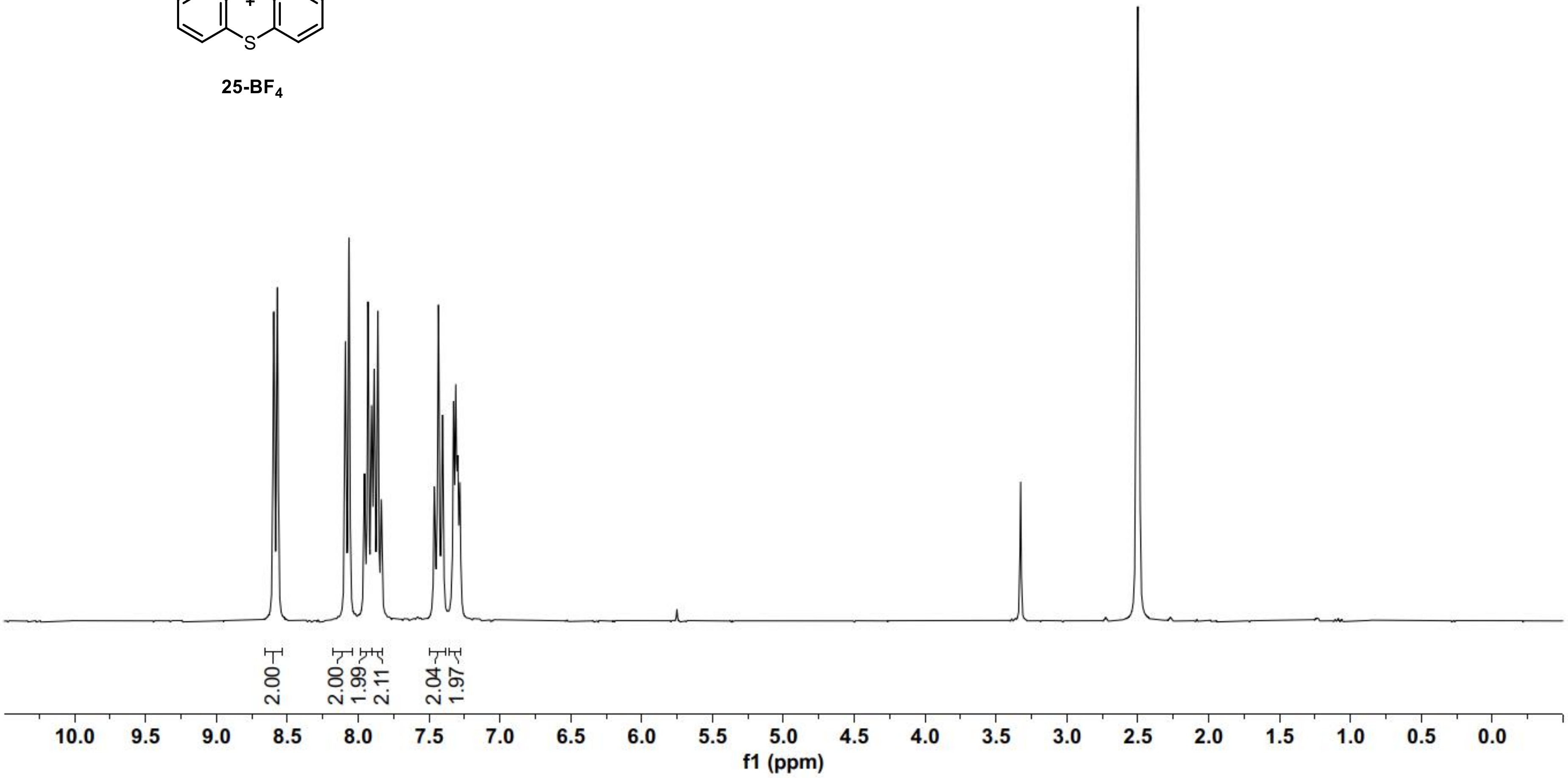

^{13}C NMR of 25-BF_4

DMSO-d_6, 76 MHz, 25 °C

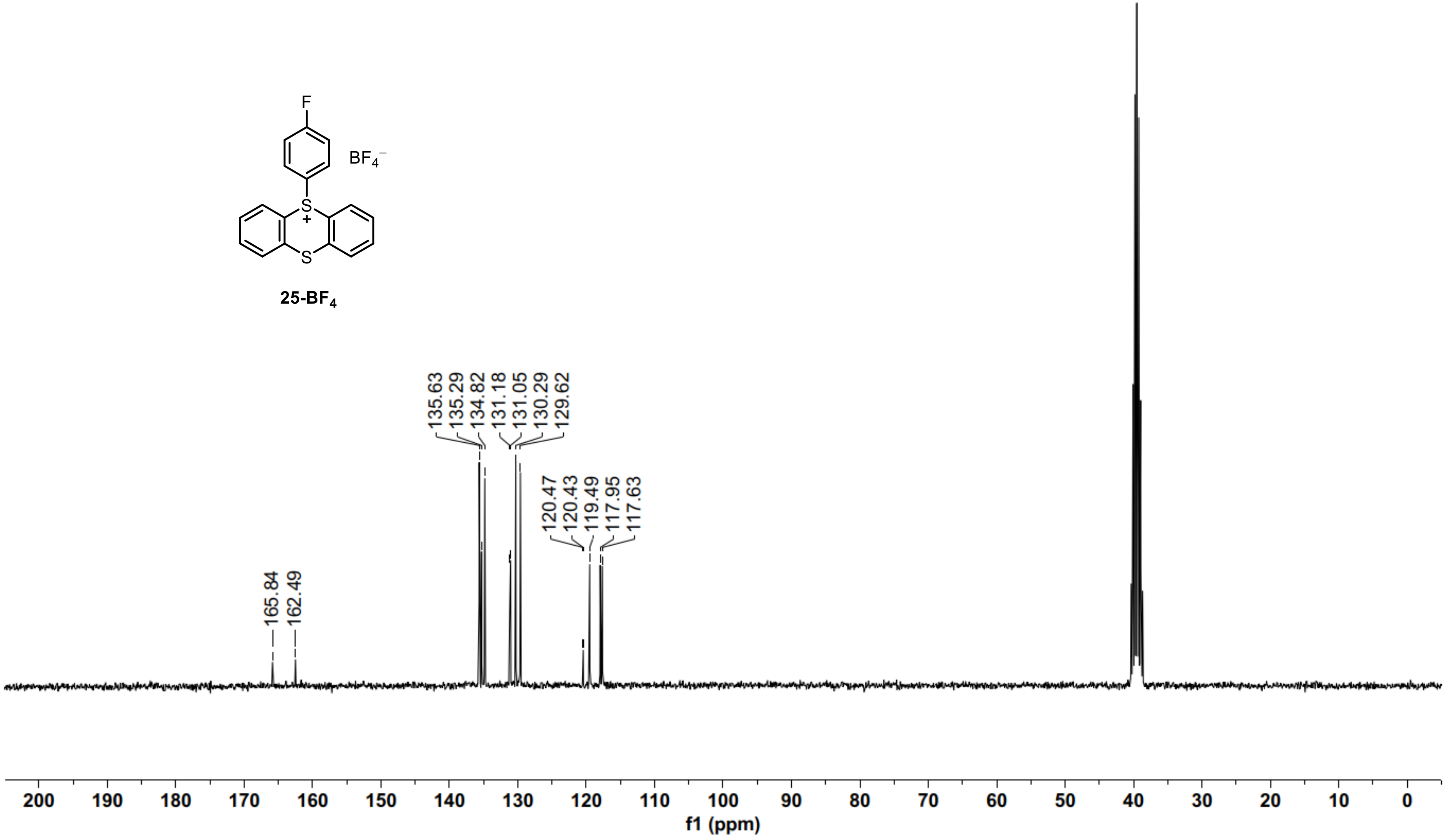

25-BF_4

^{19}F NMR of 25-BF_4

DMSO-d_6, 282 MHz, 25 °C

25-BF_4

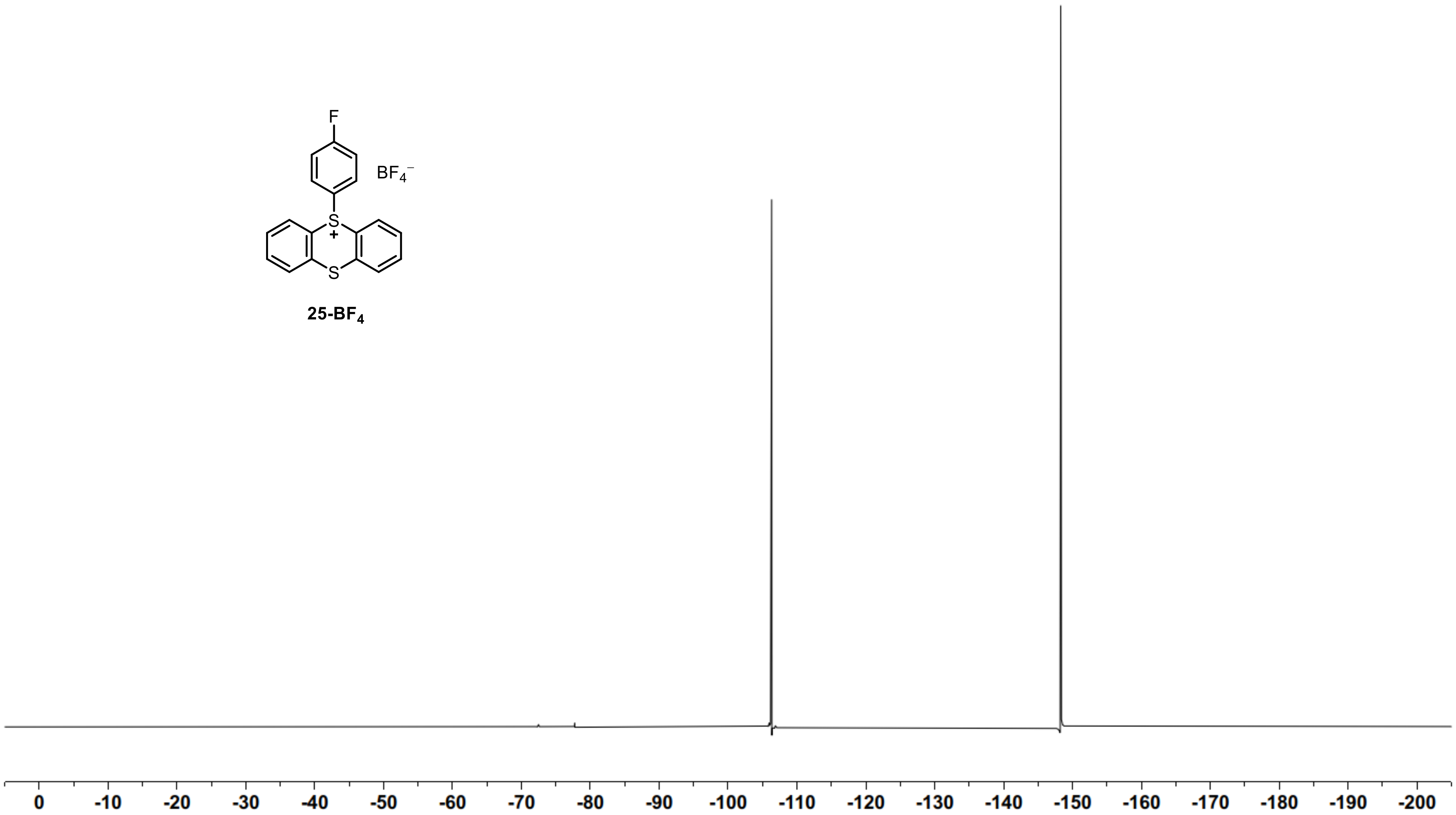

f1 (ppm)

REFERENCES

1. G. R. Fulmer, A. J. M. Miller, N. H. Sherden, H. E. Gottlieb, A. Nudelman, B. M. Stolz, J. E. Bercaw, K. I. Goldberg, *Organometallics* **2010**, *29*, 2176–2179.
2. F. Berger, M. B. Plutschack, J. Riegger, W. Yu, S. Speicher, M. Ho, N. Frank, T. Ritter, *Nature* **2019**, *567*, 223–228.
3. J. Li, J. Chen, R. Sang, W. Ham, M. B. Plutschack, F. Berger, S. Chabbra, A. Schnegg, C. Genicot, T. Ritter, *Nat. Chem.* **2020**, *12*, 56–62.
4. P. Cooper, G. E. M. Crisenza, L. J. Feron, J. F. Bower, *Angew. Chem. Int. Ed*. **2018**, *57*, 14198–14202.
5. S. Wang, X. Chen, Q. Ao, H. Wang, H. Zhai, *Chem. Commun.* **2016**, *52*, 9454–9457.
6. H. C. Brown, Y. Okamoto, *J. Am. Chem. Soc.* **1958**, *80*, 4978–4987.
7. Y. Zhao, C. Yu, W. Liang, F. W. Patureau, *Org. Lett.* **2021**, *23*, 6232–6236.
8. P. S. Engl, A. P. Häring, F. Berger, G. Berger, A. Pérez-Bitrián, T. Ritter, *J. Am. Chem. Soc.* **2019**, *141*, 13346–13351.
9. Q. Wang, W. Xiao, H. He, J. Guo, M. Wang, M. Ma, B. Zhao, *J. Org. Chem.* **2023**, *88*, 10818–10827.
10. F. Ye, F. Berger, H. Jia, J. Ford, A. Wortman, J. Börgel, C. Genicot, T. Ritter, *Angew. Chem. Int. Ed*. **2019**, *58*, 14615–14619.
11. E. M. Alvarez, M. B. Plutschack, F. Berger, T. Ritter, *Org. Lett.* **2020**, *22*, 4593–4596.
12. L. Zhang, Y. Xie, Z. Bai, T. Ritter, *Nat. Synth*. **2024**, *3*, 1490–1497.
13. G. Zhang, Z. Luo, C. Guan, X. Zhang, C. Ding, *J. Org. Chem.* **2023**, *88*, 9249–9256.
14. X.-Y. Chen, Y.-N. Li, Y. Wu, J. Bai, Y. Guo, P. Wang, *J. Am. Chem. Soc.* **2023**, *145*, 10431–10440.
15. X. Tan, W. Xiong, B. Zhu, H. Liu, W. Wu, H. Jiang, *Adv. Synth. Catal.* **2023**, *365*, 2165–2170.
